# Management of impacted fetal head at cesarean birth: A systematic review and meta‐analysis

**DOI:** 10.1111/aogs.14873

**Published:** 2024-05-24

**Authors:** Katie Cornthwaite, Jan W. van der Scheer, Sarah Kelly, Mia Schmidt‐Hansen, Jenni Burt, Mary Dixon‐Woods, Tim Draycott, Rachna Bahl

**Affiliations:** ^1^ Royal College of Obstetricians & Gynaecologists London UK; ^2^ University Hospitals Bristol and Weston Bristol UK; ^3^ THIS Institute (The Healthcare Improvement Studies Institute), Department of Public Health and Primary Care University of Cambridge, Strangeways Research Laboratory Cambridge UK; ^4^ National Institute for Health and Care Excellence London UK; ^5^ North Bristol NHS Trust Bristol UK

**Keywords:** cesarean, disimpaction, impacted fetal head, meta‐analysis, Patwardhan technique, reverse breech extraction, systematic review, vaginal push‐up

## Abstract

**Introduction:**

Despite increasing incidence of impacted fetal head at cesarean birth and associated injury, it is unclear which techniques are most effective for prevention and management. A high quality evidence review in accordance with international reporting standards is currently lacking. To address this gap, we aimed to identify, assess, and synthesize studies comparing techniques to prevent or manage impacted fetal head at cesarean birth prior to or at full cervical dilatation.

**Material and Methods:**

We searched MEDLINE, Emcare, Embase and Cochrane databases up to 1 January 2023 (PROSPERO: CRD420212750016). Included were randomized controlled trials (any size) and non‐randomized comparative studies (*n* ≥ 30 in each arm) comparing techniques or adjunctive measures to prevent or manage impacted fetal head at cesarean birth. Following screening and data extraction, we assessed risk of bias for individual studies using RoB2 and ROBINS‐I, and certainty of evidence using GRADE. We synthesized data using meta‐analysis where appropriate, including sensitivity analyses excluding data published in potential predatory journals or at risk of retraction.

**Results:**

We identified 24 eligible studies (11 randomized and 13 non‐randomized) including 3558 women, that compared vaginal disimpaction, reverse breech extraction, the Patwardhan method and/or the Fetal Pillow®. GRADE certainty of evidence was low or very low for all 96 outcomes across seven reported comparisons. Pooled analysis mostly showed no or equivocal differences in outcomes across comparisons of techniques. Although some maternal outcomes suggested differences between techniques (eg risk ratio of 3.41 [95% CI: 2.50–4.66] for uterine incision extension with vaginal disimpaction vs. reverse breech extraction), these were based on unreliable pooled estimates given very low GRADE certainty and, in some cases, additional risk of bias introduced by data published in potential predatory journals or at risk of retraction.

**Conclusions:**

The current weaknesses in the evidence base mean that no firm recommendations can be made about the superiority of any one impacted fetal head technique over another, indicating that high quality training is needed across the range of techniques. Future studies to improve the evidence base are urgently required, using a standard definition of impacted fetal head, agreed maternal and neonatal outcome sets for impacted fetal head, and internationally recommended reporting standards.

## Graphical abstract

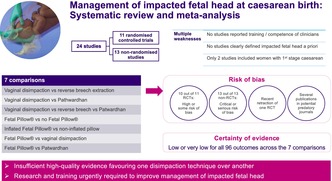

**Abbreviations**
GRADE: Grading of Recommendations Assessment, Development and Evaluation
IFH: impacted fetal head
RCT: randomized controlled trial

**Key message**

Current studies comparing the safety and effectiveness of techniques to manage impacted fetal head during cesarean birth demonstrate multiple weaknesses. Given the limited evidence for superiority of any one technique, high quality training is needed across the range of techniques.

## 1 INTRODUCTION

Difficulty in disimpacting the fetal head at cesarean birth increases maternal risks (eg postpartum hemorrhage, maternal visceral injury) and neonatal risks (eg hypoxic brain injury, intracranial hemorrhage, skull fracture).^1^, ^2^, ^3^, ^4^ Reports indicate that neonatal injuries involving an impacted fetal head (IFH) have risen in recent years, with corresponding increases in litigation claims, nationally and internationally.^4^, ^5^, ^6^, ^7^, ^8^, ^9^ IFH complicates as many as one in ten emergency cesarean births (1.5% of all births) in the United Kingdom (UK),^1^, ^4^, ^10^ making this obstetric emergency an important challenge for clinical practice.

To date, no clear consensus has emerged on the safest and most effective techniques to support disimpaction of the fetal head prior to or at cesarean birth, particularly in relation to neonatal outcomes.^2^, ^3^, ^9^, ^11^, ^12^, ^13^, ^14^ A range of techniques may be employed to prevent and manage IFH at cesarean birth (Table 1).^2^, ^9^, ^11^, ^13^, ^15^, ^16^, ^17^, ^18^, ^19^, ^20^, ^21^, ^22^, ^23^ A device used to elevate the fetal head prior to cesarean birth, known as the Fetal Pillow®,^13^, ^20^ has been reported to improve outcomes,^14^, ^24^ but most recently these have been the subject of scientific integrity concerns.^25^ Other techniques and adjunctive measures currently used to manage IFH include tocolysis, vaginal disimpaction (“push‐up”), reverse breech extraction, and the Patwardhan method,^2^, ^9^, ^11^, ^15^, ^16^, ^17^, ^18^, ^19^, ^20^, ^21^, ^22^, ^23^ with some births requiring use of several techniques employed sequentially.^1^

**TABLE 1:** The range of techniques that may be employed to prevent and manage impacted fetal head (IFH) at cesarean birth.^2^, ^9^, ^11^, ^13^, ^15^, ^16^, ^17^, ^18^, ^19^, ^20^, ^21^, ^22^, ^23^

Techniques for prevention of IFH (prior to starting cesarean birth)	
Manual vaginal disimpaction (“push‐up”)	Introducing a hand into the vagina to move the head up into the abdomen prior to making a uterine incision to reduce likelihood of IFH
Fetal pillow	Using an inflatable device in the vagina to move the head up into the abdomen prior to making a uterine incision to reduce likelihood of IFH

Techniques for management (when IFH encountered during cesarean birth)	
Manual abdominal cephalic disimpaction	Using the dominant or non‐dominant hand to flex and lift the baby's head upwards into the maternal abdomen to deliver the head
Manual vaginal disimpaction (“push‐up”)	Introducing a hand into the vagina to move the head up into the abdomen when IFH encountered at cesarean birth
Reverse breech extraction	A hand is introduced in the upper aspect of the uterus, the baby's feet are grasped and the baby is delivered feet first (breech). Once the baby's shoulders are delivered, the head is lifted out of the pelvis
Patwardhan method	A modification of reverse breech extraction, whereby the arms are delivered first.
Tocolysis	Administration of medicine (tocolysis) to relax the uterus and facilitate advanced disimpaction techniques

Though several reviews on IFH have been published,^2^, ^3^, ^11^, ^12^, ^24^ they have important limitations. For example, previous reviews have not assessed risk of publication bias of studies, nor have they been revisited following the retraction of an influential Fetal Pillow® trial.^25^ Moreover, the reviews have not been conducted or reported in accordance with international standards, such as applying Grading of Recommendations Assessment, Development, and Evaluation (GRADE) to determine the level of certainty in effect estimates of each outcome.^26^, ^27^ These scientific uncertainties, combined with lack of standardized and evidence‐based training, are implicated in poor confidence among maternity professionals in managing IFH,^20^, ^21^, ^22^, ^28^ variable practice,^13^, ^20^, ^29^ and potentially avoidable harmful care in some circumstances.^4^

A high quality evidence review is much needed. To address this need, we conducted a systematic review and meta‐analysis to identify, assess, and synthesize studies comparing techniques or adjunctive measures to prevent and manage IFH at emergency cesarean birth prior to (first stage) or at full cervical dilatation (second stage).

## 2 MATERIAL AND METHODS

We conducted a systematic review and meta‐analysis in accordance with internationally recommended guidance.^27^, ^30^, ^31^, ^32^, ^33^ It builds and expands on a preliminary search and analysis presented in a Scientific Impact Paper published by the Royal College of Obstetricians and Gynecologists.^23^

### 2.1 Eligibility criteria

We searched for full‐text articles published in English from 1980 up to 1 January 2023 using the following criteria (see Appendix S1 for full details):

#### 2.1.1 Population

Studies in women undergoing emergency cesarean birth, either prior to (first stage) or at full cervical dilatation (second stage), who were at risk of IFH (prevention) or who experienced an IFH at cesarean birth (management). Planned cesarean birth (not in labor) or non‐cephalic presentation (i.e., breech, transverse presentation or unstable lie) were excluded.

#### 2.1.2 Intervention/exposure

Techniques or adjunctive measures to prevent IFH (including inflated Fetal Pillow® and vaginal disimpaction) and/or manage IFH (including vaginal disimpaction, reverse breech extraction, Patwardhan method, and tocolysis).^2^, ^11^, ^15^, ^16^, ^17^, ^18^, ^19^, ^23^, ^24^

#### 2.1.3 Comparators

Eligible comparator groups included no intervention/exposure, non‐inflated Fetal Pillow®, or any of the eligible techniques/measures listed above.

#### 2.1.4 Outcomes

Maternal outcomes included uterine incision extension on lower segment, angle extensions into broad ligaments, operative blood loss or postpartum hemorrhage, operative time, visceral injury, infection, and duration of hospital stay. Neonatal outcomes included infant birth trauma, Apgar score at 5 min or Apgar<7 at 5 min, umbilical cord pH, neonatal intensive care unit (NICU) admission, and neonatal death.

#### 2.1.5 Study design

All randomized controlled trials (RCTs) or non‐randomized comparative prospective or retrospective cohort studies with *n* ≥ 30 per treatment arm.

#### 2.1.6 Setting

Any maternity unit or delivery suite setting worldwide.

### 2.2 Information sources and search strategy

The search strategy, developed with experienced information specialists, included MeSH headings, text words and synonyms for IFH and cesarean birth (Appendix S2). We searched the following databases to 1 January 2023: Cochrane Central Register of Controlled Trials (CENTRAL), Cochrane Database of Systematic Reviews, MEDLINE, Emcare, EMBASE and DARE. We also searched reference lists of other systematic reviews, websites of professional and maternity care organizations, and gray literature sources (Appendix S2).

### 2.3 Study selection and data extraction

One reviewer conducted title/abstract screening. A second reviewer independently screened 10% of records. Full‐text screening was conducted by one reviewer in consultation with a second reviewer. Disagreements were resolved by discussion or with a third reviewer (eg senior clinical expert). One reviewer extracted data into a standardized form. A second reviewer checked all extractions.

### 2.4 Risk of bias of individual studies

Risk of bias of RCTs was assessed using version 2.0 of Cochrane's Risk of Bias tool (RoB2).^30^ For non‐randomized studies, we used Risk Of Bias In Non‐Randomized Studies—of Interventions (ROBINS‐I).^31^ One reviewer performed the assessments. A second reviewer checked all ratings, with disagreements resolved by discussion or by a third reviewer.

### 2.5 Certainty in the overall evidence for each outcome (GRADE)

We used Grading of Recommendations Assessment, Development and Evaluation (GRADE) to assess the certainty of findings for the overall body of evidence for each outcome (http://www.gradeworkinggroup.org/). GRADE categorizes the overall evidence for an outcome as providing high, moderate, low or very low certainty.^26^ The approach initially classifies certainty of evidence from RCTs as high and from non‐randomized studies as low, and downgrades certainty if there is serious risk of bias, imprecision, inconsistency, indirectness or publication bias.^26^

### 2.6 Data synthesis

We extracted means and standard deviations for continuously reported outcomes to calculate mean differences, and event rates for dichotomously reported outcomes to calculate risk ratios. In case of zero events in at least one of the intervention groups, we calculated Peto odds ratios or risk differences. We used the inverse variance method for mean differences, Mantel‐Henszel method for risk ratios and risk differences, and the Peto method for Peto odds ratios.^27^

We pooled data for meta‐analysis (using Cochrane RevMan 5∙4) where multiple studies reported on the same outcome for the same comparison. We checked the full study descriptions to ascertain suitability for pooling, with heterogeneity additionally assessed using the *I* ^2^ statistic.^34^, ^35^ For meta‐analyses, we used random effects when *I* ^2^ was ≤80% (when *I* ^2^ was zero, fixed effects were also checked), but did not pool the data when *I* ^2^ was >80%.^27^

### 2.7 Publication bias and sensitivity analyses

To address potential publication bias, we conducted sensitivity analyses excluding data from studies where the journal or publishers were not listed on Medline, Embase, PubMed, or the Directory of Open Access Journals, and were listed on Beall's List of Potential Predatory Journals and Publishers.^36^, ^37^ We also conducted sensitivity analyses excluding other studies with the same first or last author of retracted trial/s (eg other studies with the first or last author of a retracted RCT^25^ on Fetal Pillow®). The number and sample sizes of the studies did not allow meaningful exploration of subgroup analyses.

### 2.8 Protocol and registration

The protocol was registered prospectively on PROSPERO (CRD420212750016). After registration, ‘inverted T or J incision’ was added as an additional secondary outcome. We also included a combined outcome for meta‐analysis of various types of uterine incision extensions given the variable descriptions and definitions used across identified studies.

## 3 RESULTS

**FIGURE 1:**
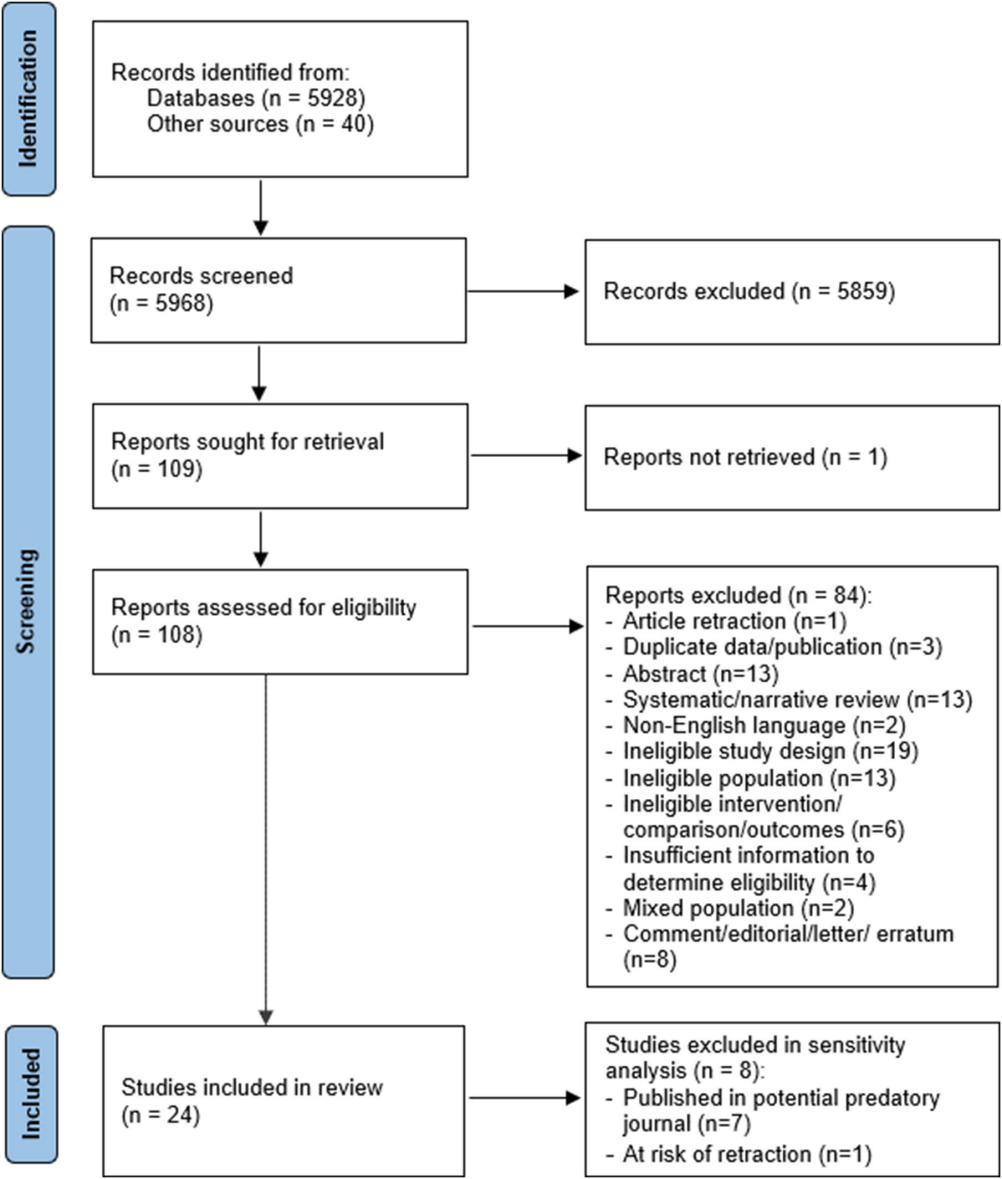
PRISMA flow diagram.

We included 24 eligible studies,^19^, ^38^, ^39^, ^40^, ^41^, ^42^, ^43^, ^44^, ^45^, ^46^, ^47^, ^48^, ^49^, ^50^, ^51^, ^52^, ^53^, ^54^, ^55^, ^56^, ^57^, ^58^, ^59^, ^60^ after screening of 5928 records and full‐text review of 108 studies (Figure 1). Table S1 lists the 84 studies excluded after full‐text review.

### 3.1 Description of included studies

The 24 included studies are summarized in Table 2, with full extraction details provided in Table S2. The studies comprised 11 RCTs and 13 non‐randomized retrospective cohort studies, most frequently conducted in low or lower‐middle income countries (18 studies), and including 3558 women overall. The studies reported on seven different comparisons involving prevention or management techniques for IFH (Table 2). None reported on a comparison that included tocolysis.

**TABLE 2:** Summary of characteristics of included studies (see Tables S3 and S4 for full details).^19^, ^35^, ^36^, ^37^, ^38^, ^39^, ^40^, ^41^, ^42^, ^43^, ^44^, ^45^, ^46^, ^47^, ^48^, ^49^, ^50^, ^51^, ^52^, ^53^, ^54^, ^55^, ^56^, ^57^

First author & year	Country	Study design^a^	Cervical dilatation	Intervention (*n*) vs. Comparator (*n*)
Vaginal disimpaction vs. reverse breech extraction				
Bastani 2012	Iran	RCT	10 cm	*n* = 30 vs. *n* = 29
Fasubaa 2002	Nigeria	RCT	Unclear	*n* = 54 vs. *n* = 54
Frass 2011	Yemen	RCT	Unclear	*n* = 59 vs. *n* = 59
Javed 2022	Pakistan	RCT	10 cm	*n* = 43 vs. *n* = 43
Nooh 2017	Egypt	RCT	10 cm	*n* = 96 vs. *n* = 96
Saleh 2014	Egypt	RCT	Unclear	*n* = 40 vs. *n* = 40
Tahir 2020	Pakistan	RCT	Unclear	*n* = 55 vs. *n* = 55
Veisi 2012	Iran	RCT	10 cm	*n* = 37 vs. *n* = 35
Vaginal disimpaction vs. Patwardhan				
Beeresh 2016	India	Non‐randomized	10 cm	*n* = 52 vs. *n* = 46
Bhattycharya 2020	India	Non‐randomized	10 cm	*n* = 50 vs. *n* = 50
Keepanasseril 2019	India	Non‐randomized	10 cm	*n* = 221 vs. *n* = 77
Lal 2018	India	Non‐randomized	10 cm	*n* = 64 vs. *n* = 56
Lenz 2019	Switzerland	Non‐randomized	10 cm	*n* = 58 vs. *n* = 45
			<10 cm	*n* = 24 vs. *n* = 10
Rakholia 2019	India	Non‐randomized	10 cm	*n* = 54 vs. *n* = 62
Vaginal disimpaction or reverse breech extraction vs. Patwardhan				
Bansiwal 2017	India	Non‐randomized	10 cm	*n* = 71 vs. *n* = 64
Bhoi 2019	India	RCT	Unclear	*n* = 291 vs. *n* = 129
Saha 2014	India	Non‐randomized	10 cm	*n* = 44 vs. *n* = 35
Fetal Pillow® vs. no pillow				
Chooi 2022	Australia	Non‐randomized	≥7 cm	*n* = 53 vs. *n* = 48
Hanley 2020	Australia	Non‐randomized	10 cm	*n* = 114 vs. *n* = 60
Sacre 2021	UK	Non‐randomized	10 cm	*n* = 170 vs. *n* = 221
Seal 2014	India	Non‐randomized	10 cm	*n* = 50 vs. *n* = 124
Inflated Fetal Pillow® vs. non‐inflated pillow				
Lassey 2020	US	RCT	10 cm	*n* = 30 vs. *n* = 30
Fetal Pillow® vs. vaginal disimpaction				
Safa 2016	Australia	Non‐randomized	10 cm	*n* = 91 vs. *n* = 69
Fetal Pillow® vs. Patwardhan				
Dutta 2019	India	RCT	10 cm	*n* = 25 vs. *n* = 25

Abbreviation: RCT, randomized controlled trial.

^a^ All non‐randomized designs were retrospective cohort study designs.

Training or competence status of the clinicians performing the techniques was not reported in any of the studies. Only two studies included women with cesarean birth prior to full cervical dilatation (Table 2).^43^, ^52^ The other 22 studies either did not report cervical dilatation or included only those undergoing cesarean birth at full cervical dilatation. In most of these studies, cesarean birth at full cervical dilatation was used as an entry criterion, with IFH assumed if the cesarean was undertaken in the second stage of labor with the fetal head at or below the ischial spines.

### 3.2 Risk of bias of individual studies

Tables S2 and S3 provide details on risk of bias assessed using RoB 2.0 or ROBINS‐I for each study. Of the 11 RCTs, one was at low risk of bias,^51^ seven at some risk of bias,^19^, ^39^, ^45^, ^46^, ^48^, ^59^, ^60^ and three at high risk of bias.^42^, ^44^, ^57^ All 13 non‐randomized studies were at serious or critical risk of bias.^38^, ^40^, ^41^, ^43^, ^47^, ^49^, ^50^, ^52^, ^53^, ^54^, ^55^, ^56^, ^58^

### 3.3 Certainty in the overall evidence for each outcome (GRADE)

Certainty of evidence was low or very low for all 96 outcomes across the seven reported comparisons (Table S4).

### 3.4 Data synthesis

**FIGURE 2:**
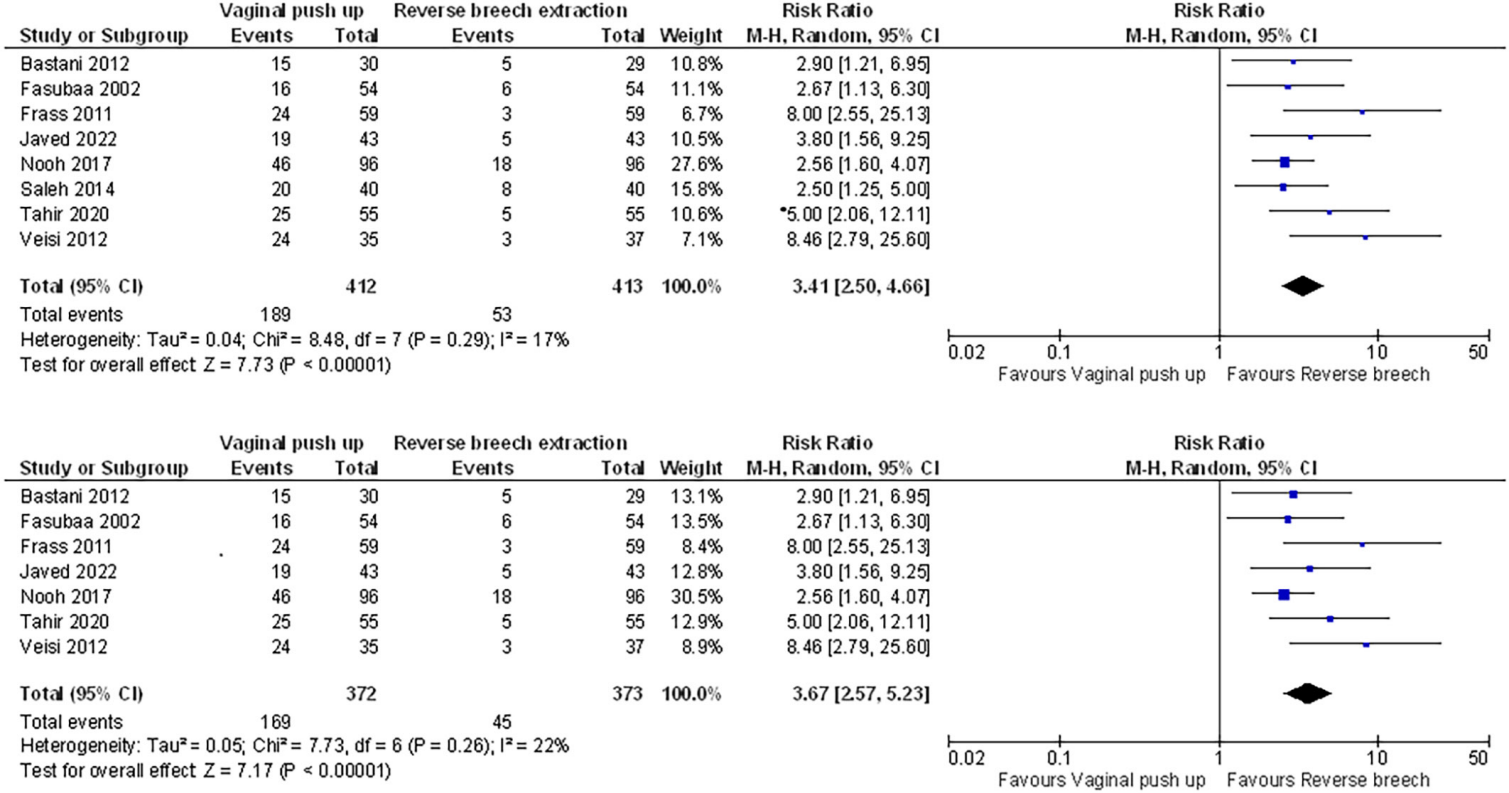
Uterine incision extension in vaginal disimpaction vs. reverse breech extraction, including (top) and excluding (bottom) a study published in a potential predatory journal. All studies reported a randomized controlled design. GRADE assessment showed very low certainty in the evidence for the outcome of this comparison (with and without the study published in a potential predatory journal). See Figure S2 and Table S4 for all outcomes and comparisons.

**FIGURE 3:**
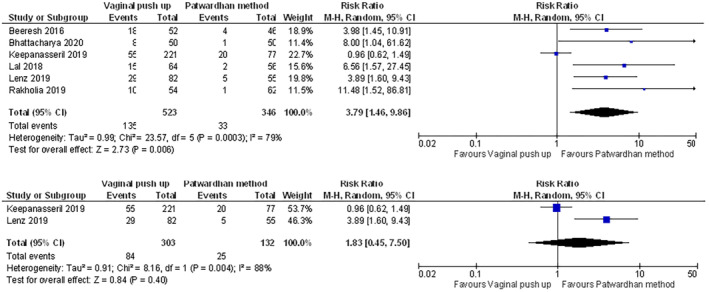
Uterine incision extension in vaginal disimpaction vs. Patwardhan method, including (top) and excluding (bottom) four studies published in potential predatory journals. All studies reported a non‐randomized design. The pooled estimate is shown, but its reliability severely impacted by heterogeneity (*I* ^2^ = 79% and 88%). GRADE assessment showed very low certainty in the evidence for the outcome of this comparison (with and without the studies published in potential predatory journals). See Figure S2 and Table S4 for all outcomes and comparisons.

**FIGURE 4:**
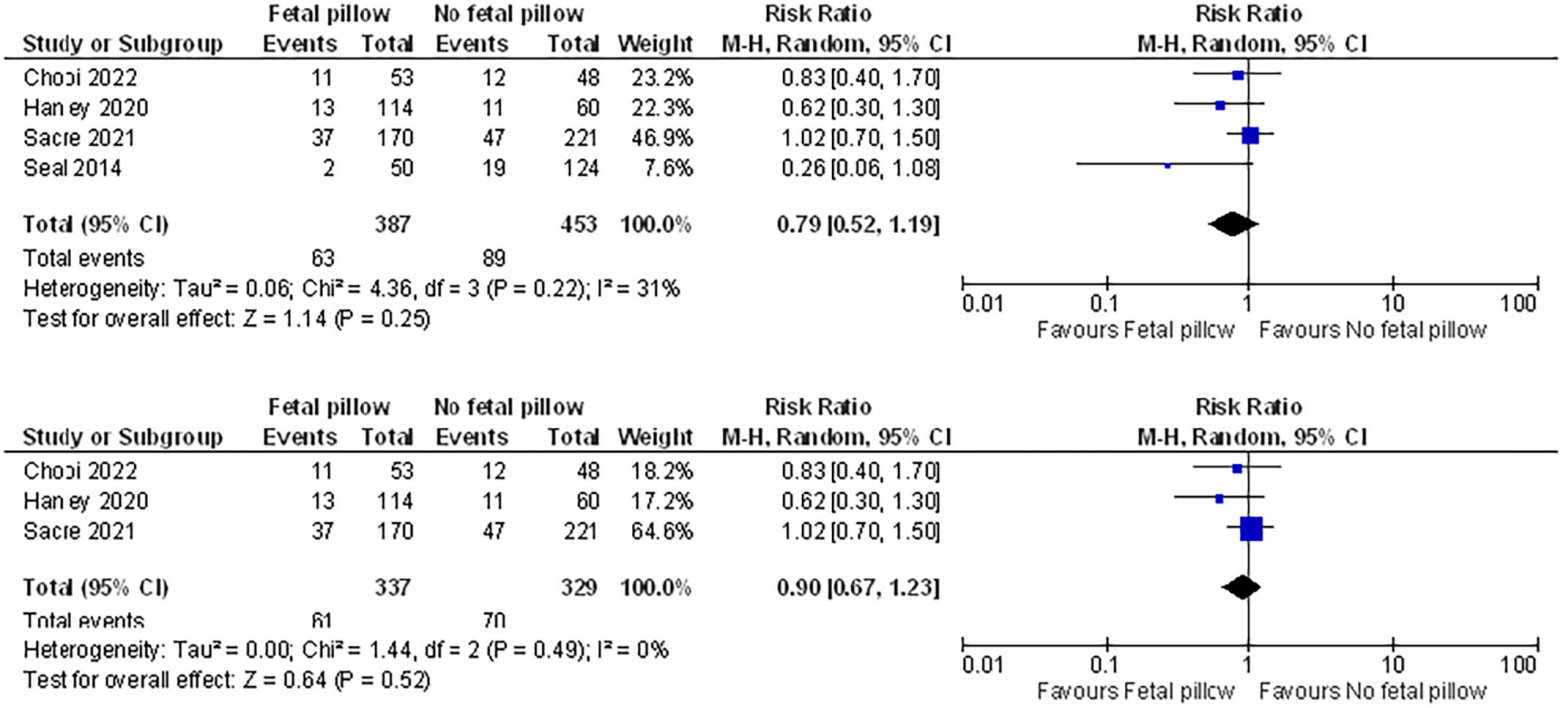
Uterine incision extension in Fetal Pillow® vs. no pillow, including (top) and excluding (bottom) a study at risk of retraction. All studies reported a non‐randomized design. GRADE assessment showed very low certainty in the evidence for the outcome of this comparison (with and without the study at risk of retraction). See Figure S2 and Table S4 for all outcomes and comparisons.

**FIGURE 5:**
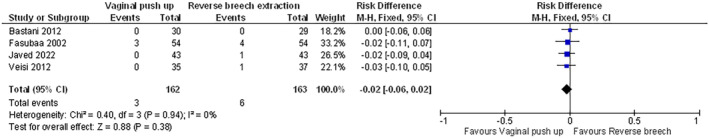
Infant birth trauma in vaginal disimpaction vs. reverse breech extraction. All studies reported a randomized controlled design, using a variety of trauma outcomes (eg “fetal injury” and “femoral fracture”). GRADE assessment showed very low certainty in the evidence for the outcome of this comparison. See Figure S2 and Table S4 for all outcomes and comparisons.

**FIGURE 6:**
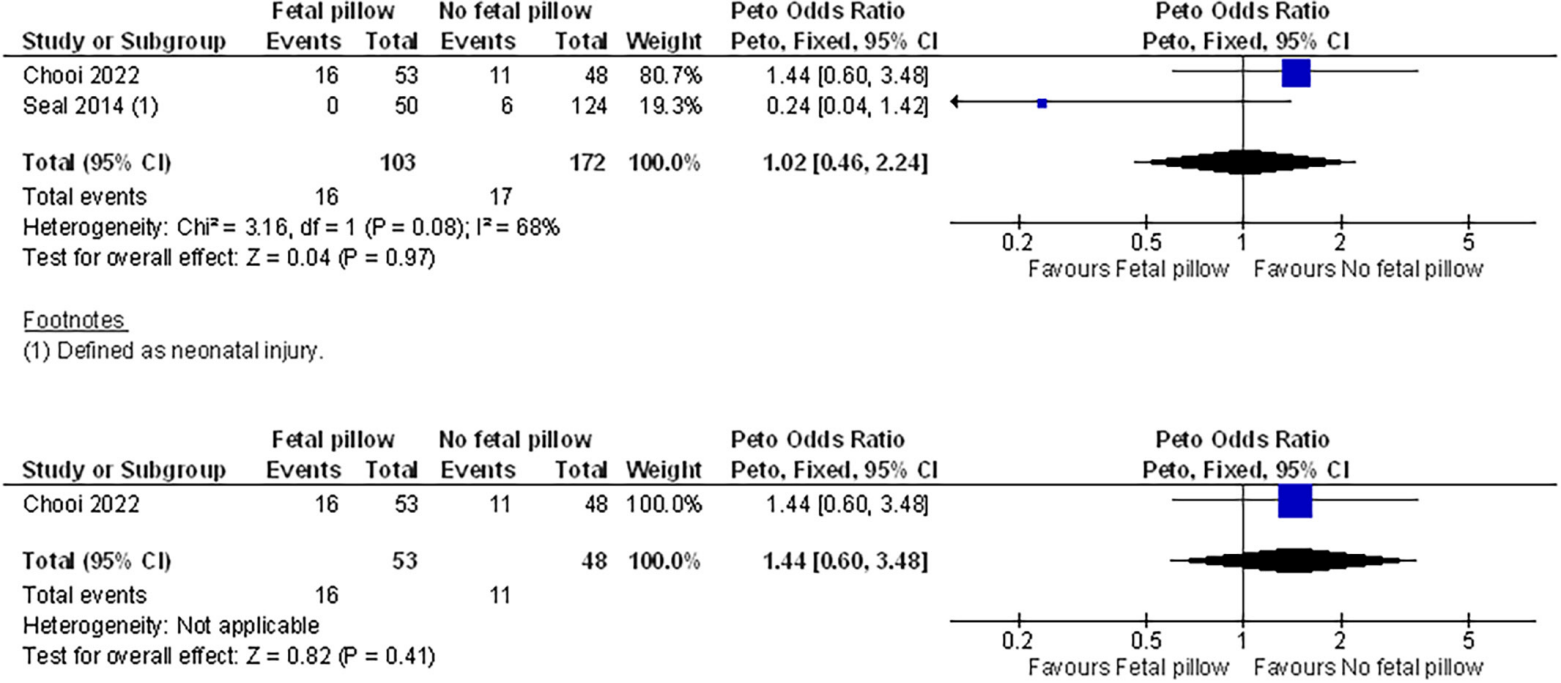
Infant birth trauma in Fetal Pillow® vs. no pillow, including (top) and excluding (bottom) a study at risk of retraction. Both studies reported a non‐randomized design. As there were zero events in one of the comparison groups for Seal 2014, precluding the calculation of a risk ratio (RR), we calculated Peto odds ratios (POR). GRADE assessment showed very low certainty in the evidence for the outcome of this comparison (with and without the study at risk of retraction). See Figure S2 and Table S4 for all outcomes and comparisons.

Table S4 provides an overview of meta‐analysis for each outcome across the seven comparisons. Figure S1 presents all pooled analyses and sensitivity analyses. Figures 2, 3, 4, 5, 6 present a selection of forest plots with pooled analysis and sensitivity analyses for studies reporting on the same outcome for the same comparison.

### 3.5 Publication bias and sensitivity analyses

We identified seven studies published in journals or with publishers listed on Beall's List of Predatory Journals and Publishers (Table S5).^38^, ^40^, ^41^, ^44^, ^50^, ^53^, ^57^ They were all identified from the reference lists of previous reviews^11^, ^12^—none of them were indexed in established databases used in our search. We also identified that several of these studies, most notably those on the Patwardhan technique, reported very similar data (eg patient characteristics), but could not verify whether these were based on the same or different datasets.^40^, ^41^, ^50^, ^53^ We conducted sensitivity analyses excluding these studies (Figure S1). We mostly found no substantial differences in pooled analyses as compared to the original analysis (see Figure 2 for an example), or found that initial larger differences in risk ratio between techniques became smaller and more equivocal (see Figure 3 for an example). After the sensitivity analyses, GRADE certainty in the evidence for all associated outcomes remained very low.

We also conducted sensitivity analyses that excluded a non‐randomized study on Fetal Pillow® (Figure S1),^58^ given that its authors, data and methods were very similar to those used in a recently retracted RCT.^25^ After exclusion of this study, we found a similar absence of risk differences when comparing Fetal Pillow® with no pillow (see Figure 4 for an example). GRADE certainty in the evidence for outcomes of this comparison also remained very low.

### 3.6 Narrative summary of data synthesis and sensitivity analyses: maternal outcomes

Efforts to compare IFH techniques and measures for effects on maternal outcomes were hampered by very serious risk of bias and/or serious imprecision (Table S4). One example is the pooled analysis of eight RCTs, suggesting a higher risk of uterine incision extensions for vaginal disimpaction vs. reverse breech extraction (risk ratio [95% CI]: 3.41 [2.50–4.66]; see Figure 2).^19^, ^39^, ^45^, ^46^, ^48^, ^57^, ^59^, ^60^ A similar risk remained in sensitivity analysis without one study in a potentially predatory journal^57^ (risk ratio [95% CI]: 3.67 [2.57–5.23]; see Figure 2). However, the GRADE assessment demonstrated we can have very little confidence in these estimates, given serious risk of bias and serious imprecision (Table S4).

Another example is the pooled analysis of six non‐randomized studies examining vaginal disimpaction vs. the Patwardhan method, which needs to be interpreted with extreme caution. Initial analysis suggested a higher risk of uterine incision extension (risk ratio [95% CI]: 3.79 [1.46–9.86]; see Figure 3),^40^, ^41^, ^49^, ^50^, ^52^, ^53^ but interpretation of the pooled estimate was hampered by high heterogeneity (*I* ^2^ of 79%). Furthermore, four of the six studies were published in potentially predatory journals.^40^, ^41^, ^50^, ^53^ Following exclusion of these in sensitivity analyses, a smaller and more equivocal difference in risk was found (risk ratio [95% CI]: 1.83 [0.45–7.50]; see Figure 3). Other data for studies comparing vaginal disimpaction with the Patwardhan method also need to be interpreted cautiously due to very serious risk of bias as well as inconsistency and/or imprecision (Table S4). For example, initial differences in risks of postpartum hemorrhage (risk ratio [95% CI]: 3.35 [1.29–8.66]) and blood transfusion (risk ratio [95% CI]: 1.85 [0.99–3.47]) largely disappeared in sensitivity analyses where the four studies published in potential predatory journals^40^, ^41^, ^50^, ^53^ were excluded (respective risk ratios [95% CI] of 1.13 [0.54–2.39] and 0.65 [0.27–1.56]; see Figure S1).

Pooled analysis that included Fetal Pillow®—either compared to no pillow, non‐inflated Fetal Pillow®, or vaginal disimpaction—demonstrated no or equivocal differences in maternal outcomes based on very low or low certainty evidence. For example, pooled analysis of four non‐randomized studies comparing Fetal Pillow® vs. no pillow identified an equivocal difference in risk of uterine incision extension (risk ratio [95% CI]: 0.79 [0.52–1.19]; see Figure 4) and postpartum hemorrhage (risk ratio [95% CI]:1.10 [0.81–1.49]; see Figure S1).^43^, ^47^, ^54^, ^58^ These differences were similar after excluding a study at risk of retraction^58^ in sensitivity analyses (respective risk ratios [95% CI] of 0.90 [0.67–1.23] and 1.13 [0.83–1.54]; see Figure 4; Figure S1). Although one non‐randomized study found a significantly shorter operative time when using Fetal Pillow® compared with no pillow (mean difference [95% CI]: 20.81 [22.31–19.31] minutes),^58^ it was at serious risk of bias with risk of retraction and very low certainty in the effect estimates.

### 3.7 Narrative summary of data synthesis and sensitivity analyses: neonatal outcomes

Efforts to compare IFH techniques and measures for effects on neonatal outcomes were similarly hampered by very serious risk of bias and/or very serious imprecision (Table S4). For example, pooled analysis of four non‐randomized studies comparing vaginal disimpaction and reverse breech extraction suggested that the risk of infant birth trauma is largely similar (risk ratio [95% CI]: −0.02 [−0.05 to 0.02]; see Figure 5),^39^, ^45^, ^48^, ^60^ though this result was prone to serious risk of bias and very serious imprecision (Table S4). Evidence for comparisons among vaginal disimpaction, reverse breech extraction and/or the Patwardhan method were equivocal for Apgar scores, infant birth trauma, umbilical artery pH, or NICU admission (Table S4 and Figure S1). This evidence remained equivocal after excluding studies published in potentially predatory journals (Figure S1).

Comparisons that included Fetal Pillow® also demonstrated equivocal findings for differences in risk of infant birth trauma, Apgar scores, umbilical artery pH, or NICU admission (Figure 6; Figure S1 and Table S4). For example, difference in risk of infant birth trauma was equivocal for Fetal Pillow® vs. no pillow (risk ratio [95% CI]: 1.02 [0.46–2.24]; see Figure 6), even more so after excluding a study at risk of retraction (Figure 6).^58^ Another example is the risk of Apgar <7 at 5 min, which was very similar in pooled analysis of two non‐randomized studies comparing Fetal Pillow® vs. no pillow (risk ratio [95% CI]: 1.01 [0.84–1.21], see Figure S1).^47^, ^54^

## 4 DISCUSSION

This review has identified, assessed, and synthesized studies comparing techniques and adjunctive measures to prevent and manage IFH at emergency cesarean birth. The current evidence for the safety and effectiveness of techniques demonstrates multiple weaknesses. We can have little or very little confidence in the majority of the effect estimates of currently available studies, given imprecision (eg wide confidence intervals) and inconsistency (eg high heterogeneity) in some cases, as well as serious study limitations in most cases. Moreover, the review highlights and addresses wider issues regarding publication bias in this area. Given the increasing scale of the problem of IFH at cesarean birth and its consequences for families,^1^, ^2^, ^3^, ^4^, ^5^, ^6^, ^7^, ^8^, ^9^, ^10^ these deficiencies in the evidence base are very concerning.

This review was conducted consistent with the latest guidance for systematic review and meta‐analysis.^27^, ^30^, ^31^ Compared with previous reviews,^2^, ^3^, ^11^, ^12^, ^24^ this methodology provides improved rigor in identifying risk of bias when synthesizing studies. For example, we assessed certainty of evidence across outcomes using GRADE,^26^, ^27^ avoided pooling data for meta‐analysis in cases of high heterogeneity,^27^ and excluded an RCT on Fetal Pillow® that was retracted owing to concerns about scientific integrity.^25^ We used a systematic and rigorous approach to identify studies published in potential predatory journals or at risk of retraction,^36^, ^37^ and performed sensitivity analyses to determine the effects of their exclusion on comparisons. One limitation is that the review only contains data from published studies that are in English. Additionally, we did not contact individual authors for under‐reported data such as dilatation status, fetal position, or level of competence or training of clinicians performing the IFH techniques.

The current evidence base for comparisons of techniques and measures to prevent or manage IFH demonstrates substantial and serious weaknesses in study design, execution and reporting. Caution is required in the interpretation of any reported differences in outcomes, since the identified weaknesses mean that the true effect may be substantially different from the estimates. Further, it remains unclear whether the findings from the low or lower middle income settings, where most of the studies were conducted, are universally generalizable because settings differ strongly in patient demographics, characteristics and risk factors, as well as service organization and maternity practices.^61^

The available evidence also suffers from lack of attention to important areas of clinical uncertainty, with many clinical IFH scenarios left unaddressed. For example, a considerable proportion of IFH cases are encountered prior to full cervical dilatation,^1^ yet most studies either focused on people undergoing cesarean birth at full cervical dilatation or did not report dilatation status at all. Future research should investigate the clinical factors that increase the risk of IFH to ensure that an adequately trained team are present for the cesarean and identify preventative measures.^62^ Similarly, the effect of tocolysis or other commonly used adjunctive measures^13^, ^20^ remain poorly understood, since they were not reported in any of the studies.

Studies included in our review did not report on training status or competence of the clinicians performing the techniques, even though deficiencies in training and competence may contribute to poor outcomes.^4^, ^5^, ^6^, ^7^ This frustrates evaluation of the techniques, as it remains unclear whether they were performed appropriately. Future research should assess and report on training, competence, and learning curve of clinicians performing techniques to prevent or manage IFH.^63^

As in other reviews,^2^, ^3^, ^11^, ^12^, ^24^ our review identified a lack of standardization in the measurement and reporting of outcomes relating to cesarean birth, and there were very few data on specific neonatal outcomes. This complicated combining of results for meta‐analysis and contributed to inconclusive findings. Future research should focus on developing a core outcome set to standardize the reporting of outcomes, both for purposes of research and for routine data collection in clinical practice.^1^, ^20^

This review also highlighted the need for a consensus‐based, universally accepted definition of IFH to streamline future research and training.^22^, ^28^ For example, in most studies IFH was assumed if the cesarean was undertaken in the second stage of labor with the fetal head at or below the ischial spines,^64^ despite recent reports suggesting that IFH may only complicate 16%–32% of second stage cesareans.^1^, ^13^ A recent consultation with over 200 obstetricians determined a consensus around a clinical definition of IFH at cesarean birth: *“a cesarean birth where the obstetrician is unable to deliver the fetal head with their usual delivering hand, and additional manoeuvres and/or tocolysis are required to disimpact and deliver the fetal head*.”^22^ This standardized definition should be used in future IFH research.

Evaluations of prevention strategies such as the Fetal Pillow® are especially urgently required, given increased use^13^, ^20^ with limited evidence of benefit. NICE guidance from December 2022 concluded that Fetal Pillow® is safe for use.^14^ However, retraction of a key RCT on Fetal Pillow® in June 2023 has led to NICE withdrawing this guidance until further review,^25^ indicating that widespread use cannot be recommended at present.

The current evidence is currently of insufficient quality to recommend universal adoption or rejection of any of the available disimpaction techniques (see Table 1) for cases of IFH. Well‐designed, adequately powered trials, based on an agreed core outcome set and with clinicians trained in applied techniques, are urgently required to further investigate management of IFH at cesarean birth. Some births also require the use of several techniques employed sequentially.^1^ Future trials should, therefore, also address the need to report whether, and in what order, consecutive IFH techniques or measures are employed.

Given the current state of the evidence of non‐superiority of a single technique for management of IFH, it is not possible to recommend or prioritize the use of one particular technique. A reasonable conclusion is that maternity professionals should be trained in the range of available techniques.^23^, ^65^ Training should include general guidance on sequential use of techniques from less to more complex techniques. It should also enable professionals to develop competence in selection and performance of the techniques most appropriate for a particular clinical situation.^23^, ^65^ This requires implementation of a standardized, high quality training program for maternity professionals based on best available evidence, including anticipation, preparation, identification, and management of IFH.^65^ Such training should also address issues concerning communication and shared decision‐making with those in labor and their birth partners.^28^, ^65^

## 5 CONCLUSION

This review provides the first evidence review and meta‐analysis of prevention and management techniques for IFH conducted in accordance with international reporting standards. It also excluded a recently retracted RCT,^25^ and used sensitivity analyses to address the very high risk of publication bias of studies^38^, ^40^, ^41^, ^44^, ^50^, ^53^, ^57^, ^58^ included in previous reviews.^11^, ^12^ The current weaknesses in the evidence base mean that no firm recommendations can be made about the superiority of any one IFH technique over another, indicating that high quality training is needed across the range of techniques. This will also be helpful where the use of multiple techniques is required. Future studies to improve the evidence base for both the prevention and management of IFH are urgently required, using a standard definition of IFH, agreed maternal and neonatal outcome sets for IFH, and internationally recommended reporting standards.

## AUTHOR CONTRIBUTION

Katie Cornthwaite and Rachna Bahl contributed to conceptualization, formal analysis, investigation, methodology, project administration, writing – original draft preparation, and writing – review and editing. Jan W. van der Scheer contributed to formal analysis, investigation, methodology, project administration, writing – original draft preparation, and writing – review and editing. Sarah Kelly contributed to formal analysis, investigation, methodology, writing – original draft preparation, and writing – review and editing. Mia Schmidt‐Hansen contributed to formal analysis, investigation, methodology, study design, and writing – review and editing. Jenni Burt and Mary Dixon‐Woods contributed to methodology, and writing – review and editing. Tim Draycott contributed to conceptualization, study design, and writing – review and editing. All authors read and approved the final manuscript.

## FUNDING INFORMATION

The Avoiding Brain Injury in Childbirth programme (Department of Health and Social Care, UK) supported this work. THIS Institute is funded by the Health Foundation, Grant/Award Number: RHZF/001 – RG88620. The Health Foundation is an independent charity committed to bringing about better health and health care for people in the UK. Mary Dixon‐Woods was an NIHR Senior Investigator (NF‐SI‐0617‐10026) during the conduct of this study.

## CONFLICT OF INTEREST STATEMENT

All authors declare they have no competing interests.

## Supporting information


Appendix S1.

Appendix S2.

Figure S1.

Table S1.

Table S2.

Table S3.

Table S4.

Table S5.

## References

[1] Cornthwaite K , Draycott T , Bahl R , Hotton E , Winter C , Lenguerrand E . Impacted fetal head: a retrospective cohort study of emergency cesarean section. Eur J Obstet Gynecol Reprod Biol. 2021;261:85‐91. 33901776 10.1016/j.ejogrb.2021.04.021

[2] Jeve YB , Navti OB , Konje JC . Comparison of techniques used to deliver a deeply impacted fetal head at full dilation: a systematic review and meta‐analysis. BJOG. 2016;123:337‐345. 26301522 10.1111/1471-0528.13593

[3] Waterfall H , Grivell RM , Dodd JM . Techniques for assisting difficult delivery at cesarean section. Cochrane Database Syst Rev. 2016;1‐30. 10.1002/14651858.CD004944.pub3 PMC8676064 26827159

[4] NHS Resolution . The Second Report: the Evolution of the Early Notification Scheme. NHS Resolution; 2022.

[5] Steer PJ . Is a fractured skull discovered in the neonate after cesarean section delivery always evidence of negligence? BJOG. 2016;123:336. 26810673 10.1111/1471-0528.13613

[6] Walker K , Thornton J . 'Negligent' technique for dis‐impacting the fetal head at cesarean section: a scientific opinion paper. BJOG. 2013;120:459.

[7] Lock J . Inquest into the Death of Nixon Martin Tonkin. Coroners Court of Queensland; 2017.

[8] NHS Resolution . The Early Notification Scheme Progress Report: Collaboration and Improved Experience for Families. NHS Resolution; 2019.

[9] Bloch C , Dore S , Hobson S . Committee opinion no. 415: impacted fetal head, second‐stage cesarean delivery. J Obstet Gynaecol Can. 2021;43:406‐413. 33640101 10.1016/j.jogc.2021.01.005

[10] Rice A , Tydeman G , Briley A , Seed PT . The impacted foetal head at cesarean section: incidence and techniques used in a single UK institution. J Obstet Gynecol. 2019;39:948‐951. 31215269 10.1080/01443615.2019.1593333

[11] Rada MP , Ciortea R , Măluțan AM , et al. Maternal and neonatal outcomes associated with delivery techniques for impacted fetal head at cesarean section: a systematic review and meta‐analysis. J Perinat Med. 2022;50:446‐456. 35119802 10.1515/jpm-2021-0572

[12] Peak AG , Barwise E , Walker KF . Techniques for managing an impacted fetal head at cesarean section: a systematic review. Eur J Obstet Gynecol Reprod Biol. 2023;281:12‐22. 36525940 10.1016/j.ejogrb.2022.12.017

[13] Wyn Jones N , Mitchell EJ , Wakefield N , et al. Impacted fetal head during second stage cesarean birth: a prospective observational study. Eur J Obstet Gynecol Reprod Biol. 2022;272:77‐81. 35290876 10.1016/j.ejogrb.2022.03.004

[14] National Institute for Health and Care Excellence (NICE) . Balloon disimpaction of the baby's head at emergency cesarean during the second stage of labour. Interventional procedures guidance [IPG744]. Published: 16 November 2022. 2022.

[15] Landesman R , Graber EA . Abdominovaginal delivery: modification of the cesarean section operation to facilitate delivery of the impacted head. Am J Obstet Gynecol. 1984;148:707‐710. 6702937 10.1016/0002-9378(84)90551-9

[16] Tan EK . Difficult cesarean delivery of an impacted head and neonatal skull fracture: can the morbidity be avoided? J Obstet Gynecol. 2007;27:427‐428. 17654204 10.1080/01443610701325861

[17] David M , Halle H , Lichtenegger W , Sinha P , Zimmermann T . Nitroglycerin to facilitate fetal extraction during cesarean delivery. Obstet Gynecol. 1998;91:119‐124. 9464734 10.1016/s0029-7844(97)00594-2

[18] Berhan Y , Berhan A . A meta‐analysis of reverse breech extraction to deliver a deeply impacted head during cesarean delivery. Int J Gynecol Obstet. 2014;124:99‐105. 10.1016/j.ijgo.2013.08.014 24290068

[19] Nooh AM , Abdeldayem HM , Ben‐Affan O . Reverse breech extraction versus the standard approach of pushing the impacted fetal head up through the vagina in cesarean section for obstructed labour: a randomised controlled trial. J Obstet Gynecol. 2017;37:459‐463. 28141942 10.1080/01443615.2016.1256958

[20] Cornthwaite K , Bahl R , Lenguerrand E , Winter C , Kingdom J , Draycott T . Impacted foetal head at cesarean section: a national survey of practice and training. J Obstet Gynecol. 2021;41:360‐366. 32723197 10.1080/01443615.2020.1780422

[21] Hanley SJ , Walker KF , Wakefield N , et al. Managing an impacted fetal head at cesarean section: a UK survey of healthcare professionals and parents. Eur J Obstet Gynecol Reprod Biol. 2022;271:88‐92. 35168126 10.1016/j.ejogrb.2022.01.033

[22] Cornthwaite K , Hewitt P , van der Scheer JW , et al. Definition, management, and training in impacted fetal head at cesarean birth: a national survey of maternity professionals. Acta Obstet Gynecol Scand. 2023;102:1219‐1226. 37430482 10.1111/aogs.14600 PMC10407013

[23] Cornthwaite K , Bahl R , Winter C , et al. Management of impacted fetal head at cesarean birth. Scientific Impact Paper No. 73. 2023. doi:10.1111/1471-0528.17534 37303275

[24] Di Girolamo R , Galliani C , Buca D , Liberati M , D'Antonio F . Outcomes of second stage cesarean section following the use of a fetal head elevation device: a systematic review and meta‐analysis. Eur J Obstet Gynecol Reprod Biol. 2021;262:1‐6. 33984724 10.1016/j.ejogrb.2021.04.043

[25] International Journal of Gynecology & Obstetrics. Retraction. Retraction , Seal SL , Dey A , et al. Randomized controlled trial of elevation of the fetal head with a fetal pillow during cesarean delivery at full cervical dilatation. First published: 15 June 2023. Int J Gynecol Obstet. 2016;2023:1129. doi:10.1002/ijgo.14924 26868074

[26] Balshem H , Helfand M , Schünemann HJ , et al. GRADE guidelines: 3. Rating the quality of evidence. J Clin Epidemiol. 2011;64:401‐406. 21208779 10.1016/j.jclinepi.2010.07.015

[27] Higgins J , Thomas J , Chandler J , et al. Cochrane Handbook for Systematic Reviews of Interventions Version 6.3 (Updated February 2022). Cochrane. 2022 www.training.cochrane.org/handbook

[28] Briley AL , Silverio SA , Shennan AH , Tydeman G . Experiences of impacted Foetal head: findings from a pragmatic focus group study of mothers and midwives. Int J Environ Res Public Health. 2023;20:7009. 37947566 10.3390/ijerph20217009 PMC10647298

[29] Cornthwaite K , Draycott T , Winter C , Lenguerrand E , Hewitt P , Bahl R . Validation of a novel birth simulator for impacted fetal head at cesarean section: an observational simulation study. Acta Obstet Gynecol Scand. 2023;102:43‐50. 36349412 10.1111/aogs.14432 PMC9780722

[30] Sterne JAC , Savović J , Page MJ , et al. RoB 2: a revised tool for assessing risk of bias in randomised trials. BMJ. 2019;l4898. 31462531 10.1136/bmj.l4898

[31] Sterne JA , Hernán MA , Reeves BC , et al. ROBINS‐I: a tool for assessing risk of bias in non‐randomised studies of interventions. BMJ. 2016;355:i4919. 27733354 10.1136/bmj.i4919 PMC5062054

[32] Page MJ , Moher D , Bossuyt PM , et al. PRISMA 2020 explanation and elaboration: updated guidance and exemplars for reporting systematic reviews. BMJ. 2021;372:n160. 33781993 10.1136/bmj.n160 PMC8005925

[33] Brooke BS , Schwartz TA , Pawlik TM . MOOSE reporting guidelines for meta‐analyses of observational studies. JAMA Surg. 2021;156:787. 33825847 10.1001/jamasurg.2021.0522

[34] Higgins JP , Thompson SG . Quantifying heterogeneity in a meta‐analysis. Stat Med. 2002;21:1539‐1558. 12111919 10.1002/sim.1186

[35] Higgins JPT , Thompson SG , Deeks JJ , Altman DG . Measuring inconsistency in meta‐analyses. BMJ. 2003;327:557‐560. 12958120 10.1136/bmj.327.7414.557 PMC192859

[36] Rice DB , Skidmore B , Cobey KD . Dealing with predatory journal articles captured in systematic reviews. Syst Rev. 2021;10:175. 34116713 10.1186/s13643-021-01733-2 PMC8194037

[37] Munn Z , Barker T , Stern C , et al. Should I include studies from “predatory” journals in a systematic review? Interim guidance for systematic reviewers. JBI Evid Synth. 2021;19:1915‐1923. 34171895 10.11124/JBIES-21-00138

[38] Bansiwal R , Anand H , Jindal M . Safety of patwardhan technique in deeply engaged head. Int J Reprod Contracept Obstet Gynecol. 2016;1562‐1565.

[39] Bastani P , Pourabolghasem S , Abbasalizadeh F , Motvalli L . Comparison of neonatal and maternal outcomes associated with head‐pushing and head‐pulling methods for impacted fetal head extraction during cesarean delivery. Int J Gynecol Obstet. 2012;118:1‐3. 10.1016/j.ijgo.2012.03.005 22521199

[40] Beeresh CS , Divyasree D , Pradeep S , Krishna L . Disengagement of the deeply engaged fetal head during cesarean section in advanced labor: Patwardhan versus push extraction. Int J Reprod Contracept Obstet Gynecol. 2016;5:68‐73.

[41] Bhattacharya R , Ramesh AC . Cesarean section of an impacted fetal head at full cervical dilatation – evaluation of Patwardhan technique. Crit Care Obst Gyne. 2020;6:4‐9.

[42] Bhoi NR , Nayak L , Sethy M , et al. Lower segment cesarean section in second stage of labor: comparison of Patwardhan method with conventional pushing method (a 3‐year study). J South Asian Feder Obst Gynae. 2018;11:263‐265.

[43] Chooi KYL , Deussen AR , Louise J , Cash S , Dodd JM . Maternal and neonatal outcomes following the introduction of the fetal pillow at a tertiary maternity hospital: a retrospective cohort study. Aust N Z J Obstet Gynecol. 2023;63:360‐364. 10.1111/ajo.13635 36480348

[44] Dutta S , Bhattacharyya S , Adhikary S , Seal S . A comparative study between modified Patwardhan technique and Foetal pillow during cesarean section in full dilatation in cases of deeply engaged foetal head. IOSR J Dental Med Sci. 2019;18:1‐7.

[45] Fasubaa O , Ezechi O , Orji E , et al. Delivery of the impacted head of the fetus at cesarean section after prolonged obstructed labour: a randomised comparative study of two methods. J Obstet Gynecol. 2002;22:375‐378. 12521457 10.1080/01443610220141290

[46] Frass KA , Al Eryani A , Al‐Harazi AH . Reverse breech extraction versus head pushing in cesarean section for obstructed labor. A comparative study in Yemen. Saudi Med J. 2011;32:1261‐1266. 22159381

[47] Hanley I , Sivanesan K , Veerasingham M , Vasudevan J . Comparison of outcomes at full‐dilation cesarean section with and without the use of a fetal pillow device. Int J Gynecol Obstet. 2020;150:228‐233. 10.1002/ijgo.13177 32320471

[48] Javed A , Noreen H , Batool I , Arshad N . Comparison of push and pull methods of delivery for deeply engaged fetal head during cesarean section for prolong second stage of labor in preventing extension of uterine incision. Rawal Med J. 2022;47.

[49] Keepanasseril A , Shaik N , Kubera N , Adhisivam B , Maurya DK . Comparison of ‘push method’ with ‘Patwardhan's method’ on maternal and perinatal outcomes in women undergoing cesarean section in second stage. J Obstet Gynaecol. 2019;39:606‐611. 30917720 10.1080/01443615.2018.1537259

[50] Lal M , Goyal P , Shamim S . Evaluation of Patwardhan technique in second stage cesarean section. Int Arch BioMed Clin Res. 2018;4:47‐49.

[51] Lassey SC , Little SE , Saadeh M , et al. Cephalic elevation device for second‐stage cesarean delivery. Obstet Gynecol. 2020;135:879‐884. 32168216 10.1097/AOG.0000000000003746 PMC7098440

[52] Lenz F , Kimmich N , Zimmermann R , Kreft M . Maternal and neonatal outcome of reverse breech extraction of an impacted fetal head during cesarean section in advanced stage of labour: a retrospective cohort study. BMC Pregnancy Childbirth. 2019;19:98. 30917799 10.1186/s12884-019-2253-3 PMC6437943

[53] Rakholia R , Jain G . Comparison of Patwardhan and push method for impacted fetal head extraction during cesarean section. Int J Adv Res. 2019;7:1203‐1206.

[54] Sacre H , Bird A , Clement‐Jones M , Sharp A . Effectiveness of the fetal pillow to prevent adverse maternal and fetal outcomes at full dilatation cesarean section in routine practice. Acta Obstet Gynecol Scand. 2021;100:949‐954. 33141937 10.1111/aogs.14038

[55] Safa H , Beckmann M . Comparison of maternal and neonatal outcomes from full‐dilatation cesarean deliveries using the fetal pillow or hand‐push method. Int J Gynecol Obstet. 2016;135:281‐284. 10.1016/j.ijgo.2016.06.013 27599604

[56] Saha PK , Gulati R , Goel P , Tandon R , Huria A . Second stage cesarean section: evaluation of patwardhan technique. J Clin Diagn Res. 2014;8:93. 24596734 10.7860/JCDR/2014/6709.3782 PMC3939599

[57] Saleh HS , Kassem GA , Mohamed MES , Ibrahiem MA , El Behery MM . Pull breech out versus push impacted head up in emergency cesarean section: a comparative study. Open J Obstet Gynecol. 2014;04:260‐265.

[58] Seal SL , Dey A , Barman SC , Kamilya G , Mukherji J . Does elevating the fetal head prior to delivery using a fetal pillow reduce maternal and fetal complications in a full dilatation cesarean section? A prospective study with historical controls. J Obstet Gynecol. 2014;34:241‐244. 24483234 10.3109/01443615.2013.844108

[59] Tahir N , Shahid G , Adil M , Fatima S . Reverse breech extraction vs head pushing for delivery of deeply impacted fetal head in emergency cesarean section. J Ayub Med Coll Abbottabad. 2020;32:497‐501. 33225651

[60] Veisi F , Zangeneh M , Malekkhosravi S , Rezavand N . Comparison of “push” and “pull” methods for impacted fetal head extraction during cesarean delivery. Int J Gynecol Obstet. 2012;118:4‐6. 10.1016/j.ijgo.2011.12.027 22541809

[61] Brown KK , Boateng GO , Ossom‐Williamson P , Haygood L . Defining, conceptualizing, and measuring perceived maternal care quality in low‐ to high‐income countries: a scoping review protocol. Syst Rev. 2021;10:61. 33627182 10.1186/s13643-021-01608-6 PMC7903867

[62] Cavoretto PI , Candiani M , Farina A . Cesarean delivery uptake trends associated with patient features and threshold for labor anomalies. JAMA Netw Open. 2023;6:e235436. 36988960 10.1001/jamanetworkopen.2023.5436

[63] Alsagheir A , Koziarz A , Belley‐Côté EP , Whitlock RP . Expertise‐based design in surgical trials: a narrative review. Can J Surg. 2021;64:E594‐E602. 34759044 10.1503/cjs.008520 PMC8592777

[64] Krispin E , Fischer O , Kneller M , et al. Fetal extraction maneuvers during cesarean delivery in the second stage of labor. J Matern Fetal Neonatal Med. 2022;35:2070‐2076. 32546078 10.1080/14767058.2020.1777273

[65] van der Scheer JW , Cornthwaite K , Hewitt P , et al. Training for managing impacted fetal head at cesarean birth: multi‐method evaluation of a pilot. BMJ Open Qual. 2023;12:e002340. 10.1136/bmjoq-2023-002340 PMC10391817 37524515

