# Supplementary material for: Management of impacted fetal head at cesarean birth: A systematic review and meta‐analysis
Source: Acta Obstet Gynecol Scand. 2024 May 24;103(9):1702–13. doi: 10.1111/aogs.14873 (PMC11324922; doi:10.1111/aogs.14873)
Supplement: Supplementary file 1 — Appendix S1. [file AOGS-103-1702-s006.pdf]

Appendix S1: Inclusion criteria

Criteria for considering studies for this review, including types of studies, participants, interventions and comparators and primary and secondary outcomes

We included studies in any maternity unit or delivery suite setting world-wide in women undergoing emergency caesarean section, either prior to (first stage) or at full cervical dilatation (second stage), who were at risk of impacted fetal head (risk factors include full cervical dilatation, failed assisted vaginal birth, prolonged labour, low fetal station or features of obstructed labour such as caput or moulding) (prevention) or who had an impacted fetal head (management). We excluded studies in women who were having an elective caesarean section or with non-cephalic presentation (i.e., breech, transverse presentation or unstable lie).

The studies should include one of the following comparisons: (1) fetal pillow versus no fetal pillow (including inserted but not inflated fetal pillow) (prevention); (2) vaginal push up versus reverse breech extraction or Patwardhan method (management); (3) reverse breech extraction versus the Patwardhan method (management); or (4) tocolysis (e.g., GTN (glyceryl trinitrate / nitroglycerine), terbutaline, salbutamol) versus other tocolysis, no tocolysis or placebo (management). Studies that compared interventions across prevention and management were also included, e.g., fetal pillow versus vaginal push up.

The studies should be randomised controlled trials of any size or non-randomised comparative prospective or retrospective cohort studies with $N \geq 30$ per treatment arm that were published in full-text articles in English. The studies should be published from 1980 and non-randomised studies should adjust for the following covariates in their analysis when there were differences between groups at baseline: maternal age, maternal BMI, smoking, parity, diabetes, gestational age +/- full cervical dilatation. If they did not adequately adjust for important covariates, they were still included, but they were downgraded for risk of bias. Studies published in languages other than English or before 1980 were not included due to time and resource constraints with translation and change in clinical practice since 1980, respectively.

The primary outcomes were uterine incision extension (incision extension on lower segment (at angles or towards cervix), angle extensions into broad ligaments); operative blood loss or post-partum haemorrhage (operative blood loss > 500ml); operative time (duration of surgery) (maternal); infant birth trauma (skull fracture / intracranial haemorrhage / other bony fracture / nerve injury); and Apgar score (at five minutes or Apgar score < 7 at five minutes) (perinatal).

The secondary outcomes were blood transfusion; visceral injury (uterine incision extension into cervix / vagina, injury to urinary tract (including ureteric injury and bladder injury), hysterectomy); infection (wound infection, endometritis, urinary tract infection, post-partum pyrexia / maternal sepsis); duration of hospital stay; decision-to-delivery interval; incision-to-delivery interval (maternal); neonatal intensive care unit (NICU) admission; umbilical artery pH / cord pH < 7.10; neonatal death (defined as death within the first 28 days of life) (perinatal); and cost.

After registration of the protocol during data extraction, ‘inverted T or J incision’ was added as an additional secondary maternal outcome. We also included a combined outcome for meta-analysis of various types of uterine incision extensions given the variable descriptions and definitions used across identified studies.
