# Supplementary material for: Management of impacted fetal head at cesarean birth: A systematic review and meta‐analysis
Source: Acta Obstet Gynecol Scand. 2024 May 24;103(9):1702–13. doi: 10.1111/aogs.14873 (PMC11324922; doi:10.1111/aogs.14873)
Supplement: Supplementary file 2 — Appendix S2. [file AOGS-103-1702-s004.pdf]

Appendix S2: Search strategies

Database(s): Medline, Emcare and Embase (Multifile) – OVID interface 1
Database(s): Cochrane Library – Wiley interface 3
Database(s): CINAHL – Proquest interface 4
Database(s): Database of Abstracts of Reviews of Effects (DARE – CRD interface https://www.crd.york.ac.uk/CRDWeb/) 11
INAHTA search strategy 11
Grey literature sources 11

Database(s): Medline, Emcare and Embase (Multifile) – OVID interface

Embase Classic+Embase 1947 to December 31 2022, **EMCARE** 1995 to December 31 2022**; Ovid MEDLINE(R) and Epub Ahead of Print, In-Process & Other Non-Indexed Citations and Daily** 1946 to December 31 2022
Date of last search: Last search conducted 12 July 2023, using date limiters to December 31 2022.
Multifile database codes: emczd = Embase Classic+Embase; emcr = Emcare; medall = MEDLINE(R) and Epub Ahead of Print, In-Process & Other Non-Indexed Citations and Daily

1	Cesarean Section/ use emczd,medall,emcr or (caesarean or cesarean or caesarian or cesarian).tw.
2	((disimpact* or impact*) adj3 (fetal or fetus or foetal) adj3 head).tw.
3	or/1-2
4	breech extraction/ use emczd,emcr or breech presentation/ use medall or ((revers* adj2 breech*) or feet first or (pull* adj3 (deliver* or extract* or method or technique*)) or pull* group*).ti,ab.
5	Patwardhan*.tw.
6	Tocolysis/ or glyceryl trinitrate/ or exp terbutaline/ or salbutamol/
7	6 use emczd,emcr
8	Tocolysis/ or Nitroglycerin/ or Terbutaline/ or exp Albuterol/
9	8 use medall
10	(tocolyses or tocolysis or tocolytic).tw.
11	(adesitrin or anginine or angiolingual or angised or angitrine or anglix or anogesic or antipressan or cardinit or cellegesic or corangil or corditrine or coro nitro or deponit or diafusor or dinamite or dynamite or epinitril or gilustenon or glonoine or gly trate or glycerol trinitrate or glyceryltrinitrate or glytrin spray or gonitro or klavikordal or lenitral or longacting trinitrine or lycinate or mil?isrol or minitran or myonit or myovin or natirose or natispray or niong or nirmin or nit ret or nitracut or nitradisc or nitrangin or nitrek or nitriderm or nitrilex or nitro or nitro bid or nitro dur or nitro?or or nitrobaat or nitrobid or nitrobid or nitro-bid or nitrocard or nitrocene or nitrocerin or nitrocin* or nitrocontin or nitroderm or nitrodisc or nitro?dur or nitrodyl or nitrofortin or nitrogard or

	nitrogesic or nitroglin or nitroglycerin* or nitroglycerol or nitroglyn or nitrol or nitrolan or nitrolande or nitrolate or nitrolingual or nitromac retard or nitromack or nitromaz or nitromed or nitromex or nitromint or nitromist or nitronal or nitronet or nitrong or nitrong or nitropaste or nitropen or nitroplast or nitroprol or nitropront or nitroprontan or nitroquick or nitroran or nitrorand or nitrorectal or nitrospan or nitrostat or nitrozell retard or nysconitrine or pancoran or percutol or perglottal or perlinganit or perlinganit or ratiopharm or rectiv or rectogesic or reminitrol or solinitrina or susadrin or susadrin or suscard or sustac or sustak or sustax or sustonit or transderm nitro or transdermnitro or tridil or trinipatch or trinitr?n or trinitrate or trinitrin* or trinitroglycerin or trinitroglycerol or trinitrol or trinitrolong or trinitrosan or trinter or trocaps or vasolator or venitrin or vernies).ti,ab.
12	(aerodur or arubendol or asmabet or asthmasian or asthmoprotect or ataline or blucodil or brasmatic or brethair or brethaire or brethine or bricanyl or bricasma or bronchodam or bronco asmo or bucanil or bucaril or butaliret or butalitab or butylin or contimit or draconyl or lanterbine or monovent or nairet or spiranyl durules or taziken or tedipulmo or terasma or terbasmin or terbron or terbul or terbulin or terburop or terbutalin* or terbuturmant or tismalin or tolbin or vacanyl).ti,ab.
13	(aero clenil or aeroclenil or aerolin or airhexal or airomir or airsalb or albuterol or almotex or asmacaire or asma?il or asmasal or asmatol or asmaven or asmavent or asmidon or asmol or asmovent or assal or asthalin or azmasol or broncho spray or broncovaleas or bronter or brytolin or butahale or buto-asma or butomix or butotal or butovent or buventol or cibutamol or cletal or cobutolin or cybutol or dilatamol or ecosal or ecovent or emplusal or epaq or exafil or farcolin or frespire or glisend or grafalin or hivent or krosalburol or libretin or loftan or medolin or mozal or novosalmol or parasma or proair or proventil or provexel or prox-s or pulmol s or repetabs or respax or respolin or respreve or salamol or salbetol or salbron or salbu easyhaler or salbuair or salbulair or salbulin or salbumol or salbupart or salbutalan or salbutamol or salbutan or salbutax or salbutin or salbutol or salbutron or salbuven or salbuvent or salda or salden or salgem or salmaplon or salmol or salmundin or salomol or saltos or salbutamol or spacehaler or sultanol or sultanol or suprasma or teoden or tobybron or venalax or vencronyl or venetlin or ventamol or ventilan or ventilastin or ventimax or ventmax or ventoaldo or ventodisk* or ventol or ventolin* or volmac or volmax or vospire or zenmolin or zibil).ti,ab.
14	or/7,9-13
15	exp fetal extraction device/ use emczd,emcr
16	(Cesarean Section/is or (Cesarean Section and protective devices).sh.) use medall
17	((elevat* adj5 (device* or method* or technique* or head*)) or pillow*).tw.
18	or/15-17
19	((push* adj3 (deliver* or extract* or head* or method or technique*)) or (push* adj (group* or push* up*1))).ti,ab.
20	or/4-5,14,18-19
21	3 and 20
22	(animal/ not human/) or nonhuman/ or exp animal experiment/ or exp experimental animal/ or animal model/ or exp rodent/
23	22 use emczd,emcr
24	(animals not humans).sh. or exp animals, laboratory/ or exp animal experimentation/ or exp models, animal/ or exp rodentia/
25	24 use medall
26	(rat or rats or mouse or mice).ti.
27	or/23,25-26
28	21 not 27

29	limit 28 to (conference abstract or conference paper or conference review or conference proceeding) [Limit not valid in Ovid MEDLINE(R),Ovid MEDLINE(R) Daily Update,Ovid MEDLINE(R) PubMed not MEDLINE,Ovid MEDLINE(R) In-Process,Ovid MEDLINE(R) Publisher; records were retained]
30	29 use emczd,emcr
31	28 not 30

Database(s): Cochrane Library – Wiley interface

Cochrane Database of Systematic Reviews, **Cochrane Central Register of Controlled Trials**, Date of last search: Dec 31 2022.

#1	MeSH descriptor: [Cesarean Section] explode all trees
#2	((caesarean or cesarean or caesarian or cesarian)):ti,ab,kw
#3	((impacted near/3 (fetal or fetus or foetal) near/3 head)):ti,ab,kw
#4	#1 or #2 or #3
#5	MeSH descriptor: [Breech Presentation] this term only
#6	(((revers* near/2 breech*) or “feet first” or (pull* near/3 (deliver* or extract* or method or technique*)) or "pull* group*")):ti,ab,kw
#7	(Patwardhan*):ti,ab,kw
#8	MeSH descriptor: [Tocolysis] this term only
#9	MeSH descriptor: [Nitroglycerin] this term only
#10	MeSH descriptor: [Terbutaline] this term only
#11	MeSH descriptor: [Albuterol] explode all trees
#12	((tocolyses or tocolysis or tocolytic)) :ti,ab,kw
#13	((adesitrin or anginine or angiolingual or angised or angitrine or anglix or anogesic or antipressan or cardinit or cellegesic or corangil or corditrine or coro nitro or deponit or diafusor or dinamite or dynamite or epinitril or gilustenon or glonoine or “gly trate” or “glycerol trinitrate” or glyceryltrinitrate or “glytrin spray” or gonitro or klavikordal or lenitral or “longacting trinitrin”e or lycinate or mil?isrol or minitran or myonit or myovin or natirose or natispray or niong or nirmin or nit ret or nitracut or nitradisc or nitrangin or nitrek or nitriderm or nitrilex or nitro or “nitro bid” or “nitro dur” or “nitro?or” or nitrobaat or nitrobid or nitrobid or “nitro-bid” or nitrocard or nitrocene or nitrocerin or nitrocin* or nitrocontin or nitroderm or nitrodisc or “nitro?dur” or nitrodyl or nitrofortin or nitrogard or nitrogesic or nitroglin or nitroglycerin* or nitroglycerol or nitroglyn or nitrol or nitrolan or nitrolande or nitrolate or nitrolingual or “nitromac retard” or nitromack or nitromaz or nitromed or nitromex or nitromint or nitromist or nitronal or nitronet or nitrong or nitrong or nitropaste or nitropen or nitroplast or nitroprol or nitropront or nitroprontan or nitroquick or nitroran or nitrorand or nitrorectal or nitrospan or nitrostat or “nitrozell retard” or nysconitrine or pancoran or percutol or perglottal or perlinganit or perlinganit or ratiopharm or rectiv or rectogesic or reminitrol or solinitrina or susadrin or susadrin or suscard or sustac or sustak or sustax or sustonit or “transderm nitro” or transdermnitro or tridil or trinipatch or trinitr?n or trinitrate or trinitrin* or trinitroglycerin or trinitroglycerol or trinitrol or trinitrolong or trinitrosan or trinter or trocaps or vasolator or venitrin or vernies)) :ti,ab,kw
#14	((aerodur or arubendol or asmabet or asthmasian or asthmoprotect or ataline or blucodil or brasmatic or brethair or brethaire or brethine or bricanyl or bricasma or bronchodam or “bronco asmo” or bucanil or bucaril or butaliret or butalitab or butylin or contimit or draconyl or lanterbine or monovent or nairet or spiranyl durules or taziken

	or tedipulmo or terasma or terbasmin or terbron or terbul or terbulin or terburop or terbutalin* or terbuturmant or tismalin or tolbin or vacanyl)):ti,ab,kw
#15	((aero clenil or aeroclenil or aerolin or airhexal or airomir or airsalb or albuterol or almotex or asmacaire or asma?il or asmasal or asmatol or asmaven or asmavent or asmidon or asmol or asmovent or assal or asthalin or azmasol or “broncho spray” or broncovaleas or bronter or brytolin or butahale or “buto-asma” or butomix or butotal or butovent or buventol or cibutamol or cletal or cobutolin or cybutol or dilatamol or ecosal or ecovent or emplusal or epaq or exafil or farcolin or frespire or glisend or grafalin or hivent or krosalburol or libretin or loftan or medolin or mozal or novosalmol or parasma or proair or proventil or provexel or “prox-s” or pulmol s or repetabs or respax or respolin or respreve or salamol or salbetol or salbron or “salbu easyhaler” or salbuair or salbulair or salbulin or salbumol or salbupart or salbutalan or salbutamol or salbutan or salbutax or salbutin or salbutol or salbutron or salbuven or salbuvent or salda or salden or salgem or salmaplon or salmol or salmundin or salomol or saltos or salbutamol or spacehaler or sultanol or sultanol or suprasma or teoden or tobybron or venalax or vencronyl or venetlin or ventamol or ventilan or ventilastin or ventimax or ventmax or ventoaldo or ventodisk* or ventol or ventolin* or volmac or volmax or vospire or zenmolin or zibil)):ti,ab,kw
#16	MeSH descriptor: [Cesarean Section] explode all trees and with qualifier(s): [instrumentation - IS]
#17	((“Cesarean Section” and ” protective devices”)):kw
#18	(((elevat* near/5 (device* or method* or technique* or head*)) or pillow*)):ti,ab,kw
#19	(((push* near/3 (deliver* or extract* or head* or method or technique*)) or "push group" or “push* up*”)):ti,ab,kw
#20	#5 or #6 or #7 or #8 or #9 or #10 or #11 or #12 or #13 or #14 or #15 or #16 or #17 or #18 or #19
#21	#4 and #20 with Cochrane Library publication date Between Jan 1980 and Sep 2021
#22	"conference":pt or (clinicaltrials or trialsearch):so
#23	#21 not #22

Database(s): CINAHL – Proquest interface

Searched up to September 2021 (then updated using EBSCO Host interface from 1/9/2021 to 31/12/2022).
Date of last search: 31/12/2022

CINAHL - Proquest interface search strategy (search to September 2021)

S16	S3 and S15
S15	S4 OR S5 OR S6 OR S7 OR S8 OR S9 OR S10 OR S11 OR S12 OR S13 OR S14
S14	TX ((((elevat* n5 (device* or method* or technique* or head*)) or pillow*))) OR TX ((((push* n3 (deliver* or extract* or head* or method or technique*)) or "push group" or “push* up*”)))
S13	MW ((“Cesarean Section” and ” protective devices”))
S12	TX ((aero clenil or aeroclenil or aerolin or airhexal or airomir or airsalb or albuterol or almotex or asmacaire or asma?il or asmasal or asmatol or asmaven or asmavent or asmidon or asmol or asmovent or assal or asthalin or azmasol or “broncho spray” or broncovaleas or bronter or brytolin or butahale or “buto-asma” or butomix or butotal or butovent or buventol or cibutamol or cletal or cobutolin or cybutol or dilatamol or ecosal or ecovent or emplusal or epaq or exafil or farcolin or frespire or glisend or

	grafalin or hivent or krosalburol or libretin or loftan or medolin or mozal or novosalmol or parasma or proair or proventil or provexel or “prox-s” or pulmol s or repetabs or respax or respolin or respreve or salamol or salbetol or salbron or “salbu easyhaler” or salbuair or salbulair or salbulin or salbumol or salbupart or salbutalan or salbutamol or salbutan or salbutax or salbutin or salbutol or salbutron or salbuven or salbuvent or salda or salden or salgem or salmaplon or salmol or salmundin or salomol or saltos or salbutamol or spacehaler or sultanol or sultanol or suprasma or teoden or tobybron or venalax or vencronyl or venetlin or ventamol or ventilan or ventilastin or ventimax or ventmax or ventoaldo or ventodisk* or ventol or ventolin* or volmac or volmax or vospire or zenmolin or zibil))
S11	TX (((tocolyses or tocolysis or tocolytic))) OR TX (((adesitrin or anginine or angiolingual or angised or angitrine or anglix or anogesic or antipressan or cardinit or cellegesic or corangil or corditrine or coro nitro or deponit or diafusor or dinamite or dynamite or epinitril or gilustenon or glonoine or “gly trate” or “glycerol trinitrate” or glyceryltrinitrate or “glytrin spray” or gonitro or klavikordal or lenitral or “longacting trinitrin”e or lycinate or mil?isrol or minitran or myonit or myovin or natirose or natispray or niong or nirmin or nit ret or nitracut or nitradisc or nitrangin or nitrek or nitriderm or nitrilex or nitro or “nitro bid” or “nitro dur” or “nitro?or” or nitrobaat or nitrobid or nitrobid or “nitro-bid” or nitrocard or nitrocene or nitrocerin or nitrocin* or nitrocontin or nitroderm or nitrodisc or “nitro?dur” or nitrodyl or nitrofortin or nitrogard or nitrogesic or nitroglin or nitroglycerin* or nitroglycerol or nitroglyn or nitrol or nitrolan or nitrolande or nitrolate or nitrolingual or “nitromac retard” or nitromack or nitromaz or nitromed or nitromex or nitromint or nitromist or nitronal or nitronet or nitrong or nitrong or nitropaste or nitropen or nitroplast or nitroprol or nitropront or nitroprontan or nitroquick or nitroran or nitrorand or nitrorectal or nitrospan or nitrostat or “nitrozell retard” or nysconitrine or pancoran or percutol or perglottal or perlinganit or perlinganit or ratiopharm or rectiv or rectogesic or reminitrol or solinitrina or susadrin or susadrin or suscard or sustac or sustak or sustax or sustonit or “transderm nitro” or transdermnitro or tridil or trinipatch or trinitr?n or trinitrate or trinitrin* or trinitroglycerin or trinitroglycerol or trinitrol or trinitrolong or trinitrosan or trinter or trocaps or vasolator or venitrin or vernies))) OR TX (((aerodur or arubendol or asmabet or asthmasian or asthmoprotect or ataline or blucodil or brasmatic or brethair or brethaire or brethine or bricanyl or bricasma or bronchodam or “bronco asmo” or bucanil or bucaril or butaliret or butalitab or butylin or contimit or draconyl or lanterbine or monovent or nairet or spiranyl durules or taziken or tedipulmo or terasma or terbasmin or terbron or terbul or terbulin or terburop or terbutalin* or terbuturmant or tismalin or tolbin or vacanyl)))
S10	(MH "Albuterol")
S9	(MH "Terbutaline")
S8	(MH "Nitroglycerin")
S7	(MH "Tocolytic Agents+")
S6	TX (Patwardhan*)
S5	TX (((revers* n2 breech*) or “feet first” or (pull* n3 (deliver* or extract* or method or technique*)) or "pull* group*"))
S4	(MH "Breech Presentation")
S3	S1 OR S2
S2	TX (((caesarean or cesarean or caesarian or cesarian))) OR TX (((impacted n3 (fetal or fetus or foetal) n3 head)))
S1	(MH "Cesarean Section+")

CINAHL – EBSCO host interface search strategy (update search from 1/9/2021 to 31/12/2022)

#	Query	Limiters/Expanders	Last Run Via	Results
S17	S3 and S15	Limiters - Published Date: 20210901-20221231<br>Expanders - Apply equivalent subjects<br>Search modes - Boolean/Phrase	Interface - EBSCOhost Research Databases<br>Search Screen - Advanced Search<br>Database - CINAHL	53
S16	S3 and S15	Expanders - Apply equivalent subjects<br>Search modes - Boolean/Phrase	Interface - EBSCOhost Research Databases<br>Search Screen - Advanced Search<br>Database - CINAHL	994
S15	S4 OR S5 OR S6 OR S7 OR S8 OR S9 OR S10 OR S11 OR S12 OR S13 OR S14	Expanders - Apply equivalent subjects<br>Search modes - Boolean/Phrase	Interface - EBSCOhost Research Databases<br>Search Screen - Advanced Search<br>Database - CINAHL	18,605
S14	TX ((((elevat* n5 (device* or method* or technique* or head*)) or pillow*))) OR TX ((((push* n3 (deliver* or extract* or head* or method or technique*)) or "push group" or “push* up*”)))	Expanders - Apply equivalent subjects<br>Search modes - Boolean/Phrase	Interface - EBSCOhost Research Databases<br>Search Screen - Advanced Search<br>Database - CINAHL	5,591
S13	MW ((“Cesarean Section” and ” protective devices”))	Expanders - Apply equivalent subjects<br>Search modes - Boolean/Phrase	Interface - EBSCOhost Research Databases<br>Search Screen - Advanced Search<br>Database - CINAHL	5

S12	TX ((aero clenil or aeroclenil or aerolin or airhexal or airomir or airsalb or albuterol or almotex or asmacaire or asma?il or asmasal or asmatol or asmaven or asmavent or asmidon or asmol or asmovent or assal or asthalin or azmasol or “broncho spray” or broncovaleas or bronter or brytolin or butahale or “buto-asma” or butomix or butotal or butovent or buventol or cibutamol or cletal or cobutolin or cybutol or dilatamol or ecosal or ecovent or emplusal or epaq or exafil or farcolin or frespire or glisend or grafalin or hivent or krosalburol or libretin or loftan or medolin or mozal or novosalmol or parasma or proair or proventil or provexel or “prox-s” or pulmol s or repetabs or respax or respolin or respreve or salamol or salbetol or salbron or “salbu easyhaler” or salbuair or salbulair or salbulin or salbumol or salbupart or salbutalan or salbutamol or salbutan or salbutax or salbutin or salbutol or salbutron or salbuven or salbuvent or salda or salden or salgem or salmaplon or salmol or salmundin or salomol or saltos or salbutamol or spacehaler or sultanol or sultanol or suprasma or teoden or tobybron or venalax or vencronyl or venetlin or ventamol or ventilan or ventilastin or ventimax or ventmax or ventoaldo or ventodisk* or ventol or ventolin* or volmac or volmax or vospire or zenmolin or zibil))	Expanders - Apply equivalent subjects Search modes - Boolean/Phrase	Interface - EBSCOhost Research Databases Search Screen - Advanced Search Database - CINAHL	3,614
S11	TX (((tocolyses or tocolysis or tocolytic))) OR TX (((adesitrin or anginine or angiolingual or angised or angitrine or anglix or anogesic or antipressan or cardinit or cellegesic or corangil or corditrine or coro nitro or deponit or diafusor or dinamite or dynamite or epinitril or gilustenon or glonoine or “gly trate” or “glycerol trinitrate” or glyceryltrinitrate or “glytrin spray” or gonitro or klavikordal or lenitral or “longacting trinitrin”e or lycinate or mil?isrol or minitran or myonit or myovin or natirose or natispray or niong or nirmin or nit ret or nitracut or	Expanders - Apply equivalent subjects Search modes - Boolean/Phrase	Interface - EBSCOhost Research Databases Search Screen - Advanced Search Database - CINAHL	5,815

	nitradisc or nitrangin or nitrek or nitriderm or nitrilex or nitro or “nitro bid” or “nitro dur” or “nitro?or” or nitrobaat or nitrobid or nitrobid or “nitro-bid” or nitrocard or nitrocene or nitrocerin or nitrocin* or nitrocontin or nitroderm or nitrodisc or “nitro?dur” or nitrodyl or nitrofortin or nitrogard or nitrogesic or nitroglin or nitroglycerin* or nitroglycerol or nitroglyn or nitrol or nitrolan or nitrolande or nitrolate or nitrolingual or “nitromac retard” or nitromack or nitromaz or nitromed or nitromex or nitromint or nitromist or nitronal or nitronet or nitrong or nitrong or nitropaste or nitropen or nitroplast or nitroprol or nitropront or nitroprontan or nitroquick or nitroran or nitrorand or nitrorectal or nitrospan or nitrostat or “nitrozell retard” or nysconitrine or pancoran or percutol or perglottal or perlinganit or perlinganit or ratiopharm or rectiv or rectogesic or reminitrol or solinitrina or susadrin or susadrin or suscard or sustac or sustak or sustax or sustonit or “transderm nitro” or transdermnitro or tridil or trinipatch or trinitr?n or trinitrate or trinitrin* or trinitroglycerin or trinitroglycerol or trinitrol or trinitrolong or trinitrosan or trinter or trocaps or vasolator or venitrin or vernies))) OR TX (((aerodur or arubendol or asmabet or asthmasian or asthmoprotect or ataline or blucodil or brasmatic or brethair or brethaire or brethine or bricanyl or bricasma or bronchodam or “bronco asmo” or bucanil or bucaril or butaliret or butalitab or butylin or contimit or draconyl or lanterbine or monovent or nairet or spiranyl durules or taziken or tedipulmo or terasma or terbasmin or terbron or terbul or terbulin or terburop or terbutalin* or terbuturmant or tismalin or tolbin or vacanyl)))			
S10	(MH "Albuterol")	Expanders - Apply equivalent subjects Search modes - Boolean/Phrase	Interface - EBSCOhost Research Databases Search Screen -	2,119

			Advanced Search Database - CINAHL	
S9	(MH "Terbutaline")	Expanders - Apply equivalent subjects Search modes - Boolean/Phrase	Interface - EBSCOhost Research Databases Search Screen - Advanced Search Database - CINAHL	344
S8	(MH "Nitroglycerin")	Expanders - Apply equivalent subjects Search modes - Boolean/Phrase	Interface - EBSCOhost Research Databases Search Screen - Advanced Search Database - CINAHL	1,700
S7	(MH "Tocolytic Agents+")	Expanders - Apply equivalent subjects Search modes - Boolean/Phrase	Interface - EBSCOhost Research Databases Search Screen - Advanced Search Database - CINAHL	2,588
S6	TX (Patwardhan*)	Expanders - Apply equivalent subjects Search modes - Boolean/Phrase	Interface - EBSCOhost Research Databases Search Screen - Advanced Search Database - CINAHL	403
S5	TX (((revers* n2 breech*) or “feet first” or (pull* n3 (deliver* or extract* or method or technique*)) or "pull* group*"))	Expanders - Apply equivalent subjects Search modes - Boolean/Phrase	Interface - EBSCOhost Research Databases Search Screen - Advanced Search	684

			Database - CINAHL	
S4	(MH "Breech Presentation")	Expanders - Apply equivalent subjects Search modes - Boolean/Phrase	Interface - EBSCOhost Research Databases Search Screen - Advanced Search Database - CINAHL	1,328
S3	S1 OR S2	Expanders - Apply equivalent subjects Search modes - Boolean/Phrase	Interface - EBSCOhost Research Databases Search Screen - Advanced Search Database - CINAHL	33,195
S2	TX (((caesarean or cesarean or caesarian or cesarian))) OR TX (((impacted n3 (fetal or fetus or foetal) n3 head)))	Expanders - Apply equivalent subjects Search modes - Boolean/Phrase	Interface - EBSCOhost Research Databases Search Screen - Advanced Search Database - CINAHL	33,195
S1	(MH "Cesarean Section+")	Expanders - Apply equivalent subjects Search modes - Boolean/Phrase	Interface - EBSCOhost Research Databases Search Screen - Advanced Search Database - CINAHL	21,632

Database(s): Database of Abstracts of Reviews of Effects (DARE – CRD interface https://www.crd.york.ac.uk/CRDWeb/)

Date of last search: September 2021

#1	MeSH DESCRIPTOR Cesarean Section IN DARE
#2	(caesarean or cesarean or caesarian or cesarian) IN DARE
#3	(impacted near3 (fetal or fetus or foetal) near3 head) IN DARE
#4	#1 OR #2 OR #3

Since 31 March 2018, the version of the HTA database on the CRD page remains available but is no longer receiving new records as submissions have transitioned to the new platform offered by INAHTA.

Update searches (from 2021 to 2023) were conducted on INAHTA (https://database.inahta.org/).

INAHTA search strategy

(("Cesarean Section"[mhe]) OR (caesarean or cesarean or caesarian or cesarian) OR ((impacted near3 (fetal or fetus or foetal) near3 head))) OR ((impacted near3 (fetal or fetus or foetal) near3 head)) OR ((caesarean or cesarean or caesarian or cesarian)) FROM 2021 TO 2023

Grey literature sources

**Website/literature source**
NHS Resolution (previously NHS Litigation Authority)<br>https://resolution.nhs.uk/ (last accessed 19/7/2023)<br>NHS Resolution Early notification report, 2019. The Early Notification scheme progress report: collaboration and improved experience for families - NHS Resolution (last accessed 19/7/2023)<br>Ten Years of Maternity Claims. An Analysis of NHS Litigation Authority Data, 2012. Ten Years of Maternity Claims: An analysis of NHS Litigation Authority data - NHS Resolution (last accessed 19/7/2023)
MBRRACE (UK)<br>https://www.npeu.ox.ac.uk/mbrrace-uk<br><br>Saving Lives, Improving Mothers' Care. Lessons learned to inform maternity care from the UK and Ireland Confidential Enquiries into Maternal Deaths and Morbidity 2018-20 (published Oct 2022). https://www.npeu.ox.ac.uk/assets/downloads/mbrrace-uk/reports/maternal-report-2022/MBRRACE-UK_Maternal_MAIN_Report_2022_UPDATE.pdf (last accessed 19/07/2023).<br><br>MBRRACE-UK Perinatal Mortality Surveillance Report UK Perinatal Deaths for Births from January to December 2020. MBRRACE-UK_Perinatal_Surveillance_Report_2020.pdf (ox.ac.uk) (last accessed 19/7/2023)<br>Previous versions of these reports also searched (https://www.npeu.ox.ac.uk/mbrrace-uk/reports/perinatal-mortality-surveillance (last accessed 19/7/2023)
NICE (UK)<br>https://www.nice.org.uk/

NICE guidance (IPG744). Balloon disimpaction of the baby's head at emergency caesarean during the second stage of labour. Published: 16 November 2022. https://www.nice.org.uk/guidance/ipg744 (last accessed December 2022; statement on website June 2023: 'As the evidence base has now changed, it will be returning to committee for re-discussion'.
Royal College of Obstetricians and Gynaecologists (RCOG)<br>https://www.rcog.org.uk/ (last accessed 19/7/2023)
Royal College of Midwives (RCM)<br>https://www.rcm.org.uk/ (last accessed 19/7/2023)
Royal College of Nursing<br>https://www.rcn.org.uk/ (last accessed 19/7/2023)
HSIB (Healthcare Safety Investigation Branch) https://www.hsib.org.uk/ (last accessed 19/7/2023)
NHS maternity review national-maternity-review-report.pdf (england.nhs.uk) (last accessed 19/7/2023)
NHS England https://www.england.nhs.uk/ (last accessed 19/7/2023)<br><br>NHS England: Saving Babies Lives Care Bundle v1 https://www.england.nhs.uk/mat-transformation/saving-babies/ (last accessed 19/7/2023)
King's Fund https://www.kingsfund.org.uk/ (last accessed 19/7/2023)
NHS Scotland/gov.Scotland/Healthcare Improvement Scotland www.healthscotland.scot/ (last accessed December 2022)<br><br>Public Health Scotland https://www.publichealthscotland.scot/ (last accessed 19/7/2023)<br><br>www.healthcareimprovementscotland.org (last accessed 19/7/2023)<br><br>https://www.perinatalnetwork.scot/ (last accessed 19/7/2023)
NHS Wales/ gov.wales<br><br>https://www.wales.nhs.uk/ (last accessed 19/7/2023)
Northern Ireland Dept. of Health<br>https://www.health-ni.gov.uk/ (last accessed 19/7/2023)
Royal College Of Physicians Of Ireland<br>https://www.rcpi.ie/Faculties-Institutes/Institute-of-Obstetricians-and-Gynaecologists/National-Clinical-Guidelines-in-Obstetrics-and-Gynaecology (last accessed 19/7/2023)<br><br>HSE Ireland<br>https://www.hse.ie/ (last accessed 19/7/2023)<br><br>https://www.hse.ie/eng/about/who/acute-hospitals-division/woman-infants/national-reports-on-womens-health/irish-maternity-indicator-system-national-report-2020.pdf (last accessed 19/7/2023)

RANZCOG (Royal Australian and New Zealand College of Obstetricians and Gynaecologists) https://ranzcog.edu.au/ (last accessed 19/7/2023)
ACOG (American College of Obstetricians and Gynecologists) https://www.acog.org/ (last accessed 19/7/2023)
FIGO (International Federation of Gynecology and Obstetrics) https://www.figo.org (last accessed 19/7/2023)
MIDAS study of Impacted Fetal Head at Caesarean Section https://www.npeu.ox.ac.uk/ukoss/completed-surveillance/ifh (last accessed 19/7/2023)
Healthcare Safety Investigation Branch UK (HSIB) https://www.hsib.org.uk/ (last accessed 19/7/2023)
Society of Obstetricians and Gynaecologists of Canada https://www.sogc.org/ (last accessed 19/7/2023)
PROMPT maternity foundation (UK) https://www.promptmaternity.org/ (last accessed 19/7/2023)
