# Supplementary material for: Management of impacted fetal head at cesarean birth: A systematic review and meta‐analysis
Source: Acta Obstet Gynecol Scand. 2024 May 24;103(9):1702–13. doi: 10.1111/aogs.14873 (PMC11324922; doi:10.1111/aogs.14873)
Supplement: Supplementary file 3 — Figure S1. [file AOGS-103-1702-s003.pdf]

Figure S1: Pooled analyses and forest plots

Contents

Vaginal disimpaction versus reverse breech extraction 3

Uterine incision extension combined for the comparison of vaginal disimpaction versus reverse breech extraction. 3

Uterine incision extension on the lower segment for the comparison of vaginal disimpaction versus reverse breech extraction. 4

Uterine incision angle extensions into broad ligaments for the comparison of vaginal disimpaction versus reverse breech extraction. 5

Maternal operative blood loss (ml) for the comparison of vaginal disimpaction versus reverse breech extraction. 6

Maternal operative blood loss >1000 ml (postpartum haemorrhage) for the comparison of vaginal disimpaction versus reverse breech extraction. 7

Operative time (minutes) for the comparison of vaginal disimpaction versus reverse breech extraction. 8

Infant birth trauma for the comparison of vaginal disimpaction versus reverse breech extraction. 9

Apgar score at five minutes for the comparison of vaginal disimpaction versus reverse breech extraction. 10

Apgar score <7 at five minutes for the comparison of vaginal disimpaction versus reverse breech extraction. 10

Maternal blood transfusion for the comparison of vaginal disimpaction versus reverse breech extraction. 11

Uterine incision extension into cervix or vagina for the comparison of vaginal disimpaction versus reverse breech extraction. 12

Injury to the urinary tract for the comparison of vaginal disimpaction versus reverse breech extraction. 13

Wound infection for the comparison of vaginal disimpaction versus reverse breech extraction. .. 14

Endometritis for the comparison of vaginal disimpaction versus reverse breech extraction. 15

Post-partum pyrexia/maternal sepsis for the comparison of vaginal disimpaction versus reverse breech extraction. 16

Maternal duration of hospital stay (days) for the comparison of vaginal disimpaction versus reverse breech extraction. 17

NICU admission for the comparison of vaginal disimpaction versus reverse breech extraction. 18

Neonatal death for the comparison of vaginal disimpaction versus reverse breech extraction. 19

Vaginal disimpaction versus Patwardhan method 20

Uterine incision extensions (combined) for the comparison of vaginal disimpaction versus Patwardhan method. 20

Uterine incision extension on lower segment for the comparison of vaginal disimpaction versus Patwardhan method. 21

Post-partum haemorrhage (operative blood loss >1000 ml) for the comparison of vaginal disimpaction versus Patwardhan method 22
Maternal operative blood loss (ml) for the comparison of vaginal disimpaction versus Patwardhan method. 23
Operative time (minutes) for the comparison of vaginal disimpaction versus Patwardhan method. 24
Infant birth trauma for the comparison of vaginal disimpaction versus Patwardhan method. 25
Apgar score <7 at 5 minutes for the comparison of vaginal disimpaction versus Patwardhan method. 26
Blood transfusion for the comparison of vaginal disimpaction versus Patwardhan method. 27
Visceral injury or hysterectomy (injury to urinary tract) for the comparison of vaginal disimpaction versus Patwardhan method 28
NICU admission for the comparison of vaginal disimpaction versus Patwardhan method. 29
Neonatal death for the comparison of vaginal disimpaction versus Patwardhan method. 30
Visceral injury or hysterectomy (hysterectomy) for the comparison of vaginal disimpaction versus Patwardhan method. 31
Vaginal disimpaction or reverse breech extraction versus Patwardhan method 32
Uterine incision extension: Incision extension on lower segment for the comparison of vaginal push or pull versus Patwardhan method. 32
Maternal blood transfusion for the comparison of vaginal push or pull versus Patwardhan method 33
NICU admission for the comparison of vaginal push or pull versus Patwardhan method 34
Fetal pillow® versus no pillow 35
Uterine incision extensions combined for the comparison of Fetal pillow® versus no pillow. 35
Uterine incision extension on lower segment for the comparison of Fetal pillow® versus no pillow. 36
Maternal operative blood loss (ml) for the comparison of Fetal pillow® versus no pillow. 37
Maternal operative blood loss >1000 ml (postpartum haemorrhage) for the comparison of Fetal pillow® versus no pillow. 38
Infant birth trauma for the comparison of Fetal pillow® versus no pillow. 39
Apgar score <7 at five minutes for the comparison of Fetal pillow® versus no pillow 40
Maternal blood transfusion for the comparison of Fetal pillow® versus no pillow. 41
Uterine incision extension into cervix or vagina for the comparison of Fetal pillow® versus no pillow. 42
Maternal duration of hospital stay (days) for the comparison of Fetal pillow® versus no pillow. 43
NICU admission for the comparison of Fetal pillow® versus no pillow 44
Umbilical artery pH for the comparison of Fetal pillow® versus no pillow 45

Vaginal disimpaction versus reverse breech extraction

Uterine incision extension combined for the comparison of vaginal disimpaction versus reverse breech extraction.

Please note all the studies are randomised controlled trials. Both Frass 2011 and Saleh 2014 defined this outcome as inadvertent extension of uterine incision beyond normal limits. In Nooh 2017 the outcome was defined as “inadvertent increase in the length of incision beyond normal limits: any extra length beyond the initial incision made by the scalpel, with or without the use of scissors and/or digital widening, which can go laterally further than the anterior surface of the uterus or downwards, that required anything more than the standard uterine closure” (p. 460). Javed 2022 defined this outcome as ‘Extension of uterine incision was assessed subjectively by surgeon as presence or absence of inadvertent extension of uterine incision beyond 1-2cm from normal lower segment uterine incision or tear/laceration more than 2cm in lower uterine segment.’

	Vaginal push up		Reverse breech extraction			Risk Ratio
**Study or Subgroup**	**Events**	**Total**	**Events**	**Total**	**Weight**	**M-H, Random, 95% CI**
Bastani 2012	15	30	5	29	10.8%	2.90 [1.21, 6.95]
Fasubaa 2002	16	54	6	54	11.1%	2.67 [1.13, 6.30]
Frass 2011	24	59	3	59	6.7%	8.00 [2.55, 25.13]
Javed 2022	19	43	5	43	10.5%	3.80 [1.56, 9.25]
Nooh 2017	46	96	18	96	27.6%	2.56 [1.60, 4.07]
Saleh 2014	20	40	8	40	15.8%	2.50 [1.25, 5.00]
Tahir 2020	25	55	5	55	10.6%	5.00 [2.06, 12.11]
Veisi 2012	24	35	3	37	7.1%	8.46 [2.79, 25.60]
**Total (95% CI)**		**412**		**413**	**100.0%**	**3.41 [2.50, 4.66]**
Total events	189		53			

Heterogeneity: Tau² = 0.04; Chi² = 8.48, df = 7 (P = 0.29); I² = 17%
Test for overall effect: Z = 7.73 (P < 0.00001)

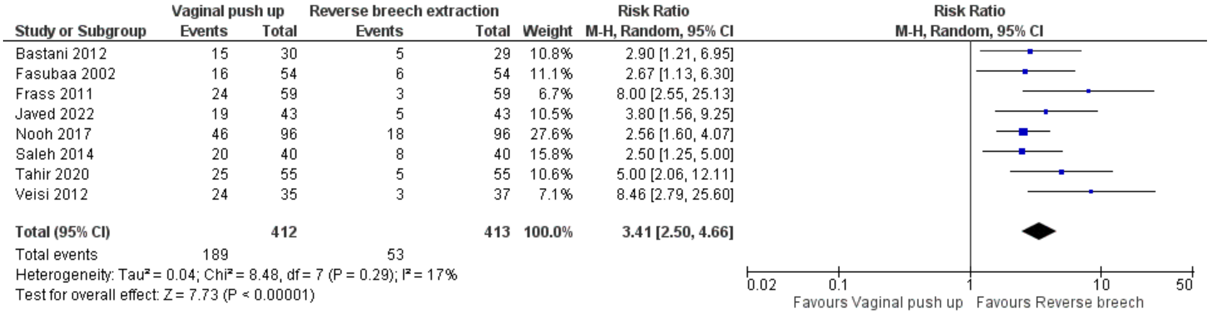

Sensitivity analysis without Saleh 2014 study data

	Vaginal push up		Reverse breech extraction			Risk Ratio
**Study or Subgroup**	**Events**	**Total**	**Events**	**Total**	**Weight**	**M-H, Random, 95% CI**
Bastani 2012	15	30	5	29	13.1%	2.90 [1.21, 6.95]
Fasubaa 2002	16	54	6	54	13.5%	2.67 [1.13, 6.30]
Frass 2011	24	59	3	59	8.4%	8.00 [2.55, 25.13]
Javed 2022	19	43	5	43	12.8%	3.80 [1.56, 9.25]
Nooh 2017	46	96	18	96	30.5%	2.56 [1.60, 4.07]
Tahir 2020	25	55	5	55	12.9%	5.00 [2.06, 12.11]
Veisi 2012	24	35	3	37	8.9%	8.46 [2.79, 25.60]
**Total (95% CI)**		**372**		**373**	**100.0%**	**3.67 [2.57, 5.23]**
Total events	169		45			

Heterogeneity: Tau² = 0.05; Chi² = 7.73, df = 6 (P = 0.26); I² = 22%
Test for overall effect: Z = 7.17 (P < 0.00001)

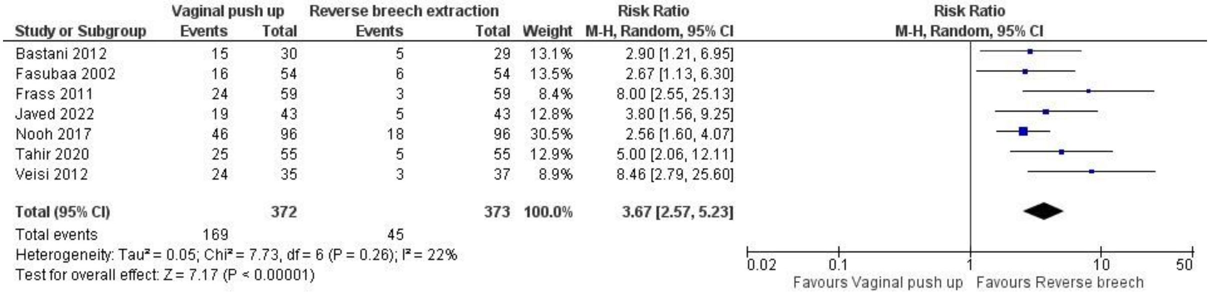

Uterine incision extension on the lower segment for the comparison of vaginal disimpaction versus reverse breech extraction.

Please note all the studies are randomised controlled trials. Both Frass 2011 and Salah 2014 defined this outcome as inadvertent extension of uterine incision beyond normal limits. In Nooh 2017 the outcome was defined as “inadvertent increase in the length of incision beyond normal limits: any extra length beyond the initial incision made by the scalpel, with or without the use of scissors and/or digital widening, which can go laterally further than the anterior surface of the uterus or downwards, that required anything more than the standard uterine closure” (p. 460). Javed 2022 defined this outcome as ‘Extension of uterine incision was assessed subjectively by surgeon as presence or absence of inadvertent extension of uterine incision beyond 1-2cm from normal lower segment uterine incision or tear/laceration more than 2cm in lower uterine segment.’

	Vaginal push up		Reverse breech extraction			Risk Ratio
**Study or Subgroup**	**Events**	**Total**	**Events**	**Total**	**Weight**	**M-H, Random, 95% CI**
Frass 2011	24	59	3	59	10.3%	8.00 [2.55, 25.13]
Javed 2022	19	43	5	43	14.8%	3.80 [1.56, 9.25]
Nooh 2017	46	96	18	96	28.8%	2.56 [1.60, 4.07]
Saleh 2014	20	40	8	40	20.1%	2.50 [1.25, 5.00]
Tahir 2020	25	55	5	55	15.0%	5.00 [2.06, 12.11]
Veisi 2012	24	35	3	37	10.9%	8.46 [2.79, 25.60]
**Total (95% CI)**		**328**		**330**	**100.0%**	**3.82 [2.51, 5.82]**
Total events	158		42			

Heterogeneity: Tau² = 0.10; Chi² = 8.16, df = 5 (P = 0.15); I² = 39%
Test for overall effect: Z = 6.27 (P < 0.00001)

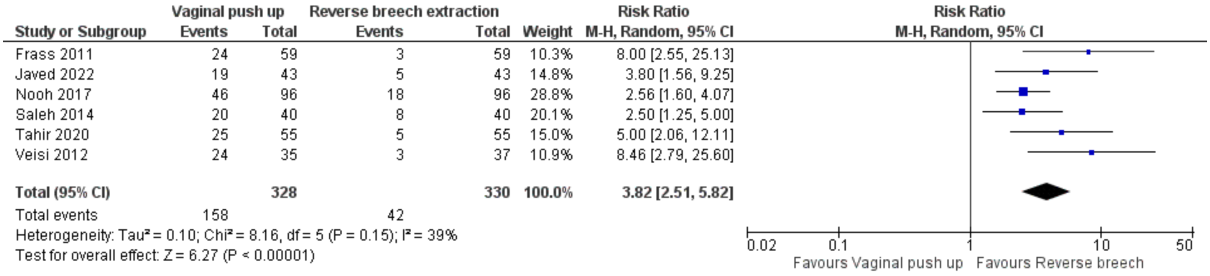

Sensitivity analysis without Saleh 2014 study data

	Vaginal push up		Reverse breech extraction			Risk Ratio
**Study or Subgroup**	**Events**	**Total**	**Events**	**Total**	**Weight**	**M-H, Random, 95% CI**
Frass 2011	24	59	3	59	13.7%	8.00 [2.55, 25.13]
Javed 2022	19	43	5	43	19.1%	3.80 [1.56, 9.25]
Nooh 2017	46	96	18	96	33.6%	2.56 [1.60, 4.07]
Tahir 2020	25	55	5	55	19.2%	5.00 [2.06, 12.11]
Veisi 2012	24	35	3	37	14.4%	8.46 [2.79, 25.60]
**Total (95% CI)**		**288**		**290**	**100.0%**	**4.36 [2.63, 7.21]**
Total events	138		34			

Heterogeneity: Tau² = 0.14; Chi² = 7.11, df = 4 (P = 0.13); I² = 44%
Test for overall effect: Z = 5.73 (P < 0.00001)

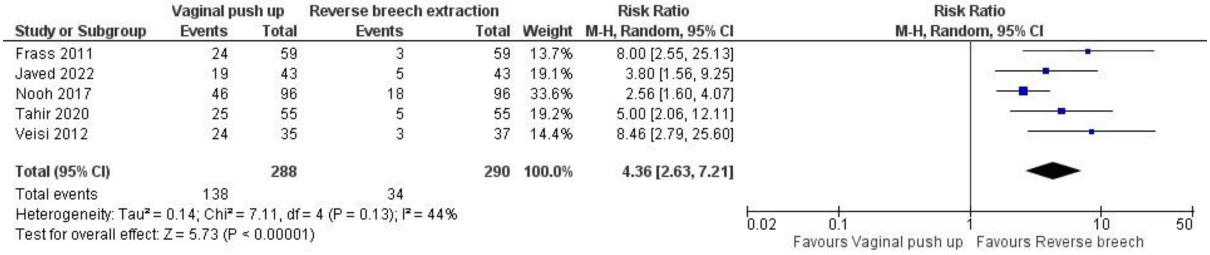

Uterine incision angle extensions into broad ligaments for the comparison of vaginal disimpaction versus reverse breech extraction.

Please note all the studies are randomised controlled trials.

	Vaginal push up		Reverse breech extraction			Risk Ratio
Study or Subgroup	Events	Total	Events	Total	Weight	M-H, Random, 95% CI
Bastani 2012	15	30	5	29	49.1%	2.90 [1.21, 6.95]
Fasubaa 2002	16	54	6	54	50.9%	2.67 [1.13, 6.30]
**Total (95% CI)**		**84**		**83**	**100.0%**	**2.78 [1.51, 5.13]**
Total events	31		11			

Heterogeneity: Tau² = 0.00; Chi² = 0.02, df = 1 (P = 0.89); I² = 0%
Test for overall effect: Z = 3.27 (P = 0.001)

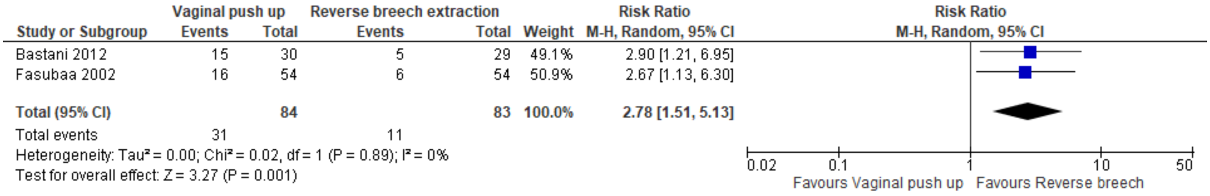

As I^2=0%, fixed effects analysis also shown below.

	Vaginal push up		Reverse breech extraction			Risk Ratio
Study or Subgroup	Events	Total	Events	Total	Weight	M-H, Fixed, 95% CI
Bastani 2012	15	30	5	29	45.9%	2.90 [1.21, 6.95]
Fasubaa 2002	16	54	6	54	54.1%	2.67 [1.13, 6.30]
**Total (95% CI)**		**84**		**83**	**100.0%**	**2.77 [1.50, 5.12]**
Total events	31		11			

Heterogeneity: Chi² = 0.02, df = 1 (P = 0.89); I² = 0%
Test for overall effect: Z = 3.26 (P = 0.001)

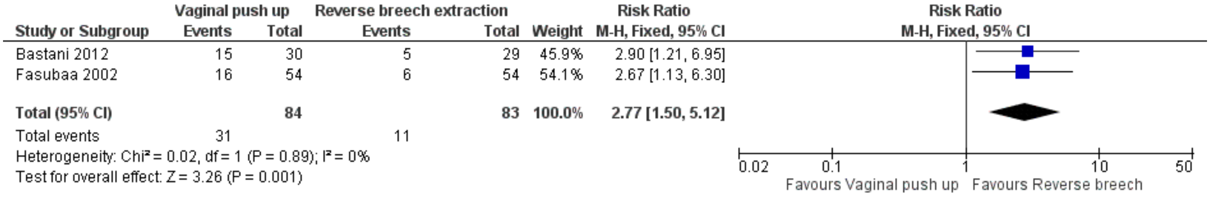

Maternal operative blood loss (ml) for the comparison of vaginal disimpaction versus reverse breech extraction.

Please note the overall pooled estimate has not been included due to very high levels of heterogeneity (I^2=98%). All the studies are randomised controlled trials.

	Favours Vaginal push up			Reverse breech extraction			Mean Difference
Study or Subgroup	Mean	SD	Total	Mean	SD	Total	IV, Random, 95% CI
Fasubaa 2002	1,256.5	54.3	54	898.4	35.2	54	358.10 [340.84, 375.36]
Frass 2011	1,231	471	59	787	519	59	444.00 [265.17, 622.83]
Javed 2022	515	97.2	43	438	72.4	43	77.00 [40.77, 113.23]
Nooh 2017	1,367	322	96	832	221	96	535.00 [456.88, 613.12]
Saleh 2014	1,321	572	40	878	674	40	443.00 [169.05, 716.95]
Tahir 2020	1,542.36	188.27	55	1,090	130.08	55	452.36 [391.88, 512.84]
Veisi 2012	571	106	35	457	68	37	114.00 [72.61, 155.39]

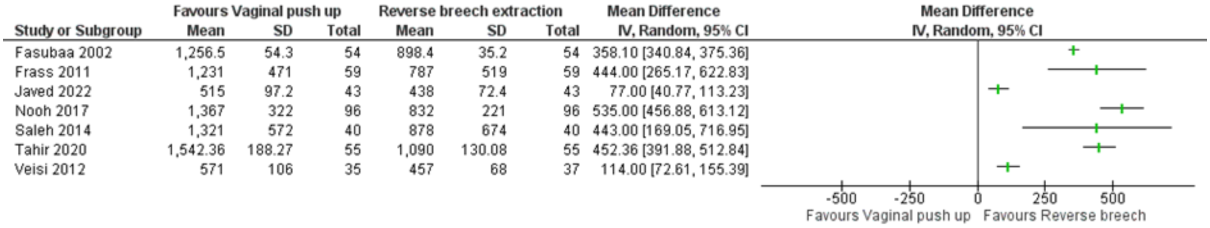

Sensitivity analysis without Saleh 2014 study data

	Favours Vaginal push up			Reverse breech extraction			Mean Difference
Study or Subgroup	Mean	SD	Total	Mean	SD	Total	IV, Random, 95% CI
Fasubaa 2002	1,256.5	54.3	54	898.4	35.2	54	358.10 [340.84, 375.36]
Frass 2011	1,231	471	59	787	519	59	444.00 [265.17, 622.83]
Javed 2022	515	97.2	43	438	72.4	43	77.00 [40.77, 113.23]
Nooh 2017	1,367	322	96	832	221	96	535.00 [456.88, 613.12]
Tahir 2020	1,542.36	188.27	55	1,090	130.08	55	452.36 [391.88, 512.84]
Veisi 2012	571	106	35	457	68	37	114.00 [72.61, 155.39]

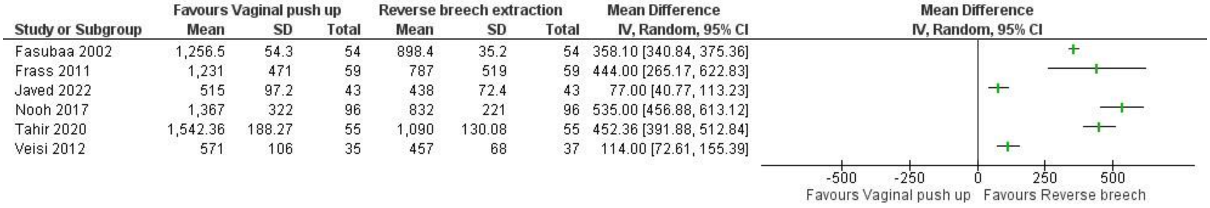

Maternal operative blood loss >1000 ml (postpartum haemorrhage) for the comparison of vaginal disimpaction versus reverse breech extraction.

All the studies are randomised controlled trials.

	Vaginal push up		Reverse breech extraction			Risk Ratio
Study or Subgroup	Events	Total	Events	Total	Weight	M-H, Random, 95% CI
Frass 2011	10	59	5	59	55.5%	2.00 [0.73, 5.50]
Nooh 2017	5	96	2	96	21.8%	2.50 [0.50, 12.57]
Saleh 2014	5	40	2	40	22.7%	2.50 [0.51, 12.14]
**Total (95% CI)**		**195**		**195**	**100.0%**	**2.21 [1.04, 4.69]**
Total events	20		9			
Heterogeneity: Tau² = 0.00; Chi² = 0.08, df = 2 (P = 0.96); I² = 0%						
Test for overall effect: Z = 2.06 (P = 0.04)						

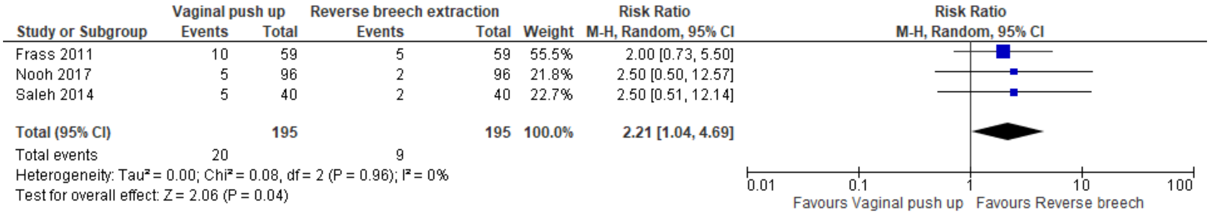

As I^2=0% fixed effects analysis also shown below.

	Vaginal push up		Reverse breech extraction			Risk Ratio
Study or Subgroup	Events	Total	Events	Total	Weight	M-H, Fixed, 95% CI
Frass 2011	10	59	5	59	55.6%	2.00 [0.73, 5.50]
Nooh 2017	5	96	2	96	22.2%	2.50 [0.50, 12.57]
Saleh 2014	5	40	2	40	22.2%	2.50 [0.51, 12.14]
**Total (95% CI)**		**195**		**195**	**100.0%**	**2.22 [1.05, 4.72]**
Total events	20		9			
Heterogeneity: Chi² = 0.08, df = 2 (P = 0.96); I² = 0%						
Test for overall effect: Z = 2.08 (P = 0.04)						

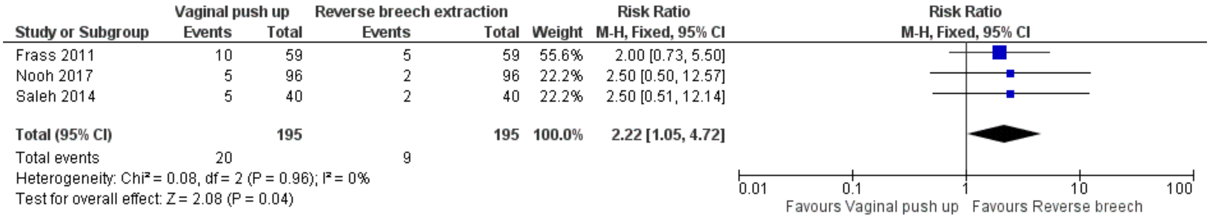

Sensitivity analysis without Saleh 2014 study data

	Vaginal push up		Reverse breech extraction			Risk Ratio
Study or Subgroup	Events	Total	Events	Total	Weight	M-H, Random, 95% CI
Frass 2011	10	59	5	59	71.8%	2.00 [0.73, 5.50]
Nooh 2017	5	96	2	96	28.2%	2.50 [0.50, 12.57]
**Total (95% CI)**		**155**		**155**	**100.0%**	**2.13 [0.90, 5.02]**
Total events	15		7			
Heterogeneity: Tau² = 0.00; Chi² = 0.05, df = 1 (P = 0.82); I² = 0%						
Test for overall effect: Z = 1.73 (P = 0.08)						

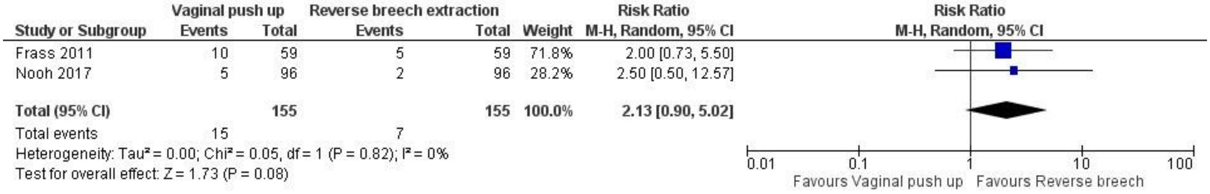

Operative time (minutes) for the comparison of vaginal disimpaction versus reverse breech extraction.

Please note the overall pooled estimate has not been included due to very high levels of heterogeneity (I^2=99%). All the studies are randomised controlled trials.

	Vaginal push up			Reverse breech extraction			Mean Difference
Study or Subgroup	Mean	SD	Total	Mean	SD	Total	IV, Random, 95% CI
Bastani 2012	75.6	12.2	30	75.5	10.8	29	0.10 [-5.77, 5.97]
Fasubaa 2002	88.5	4.5	54	55.6	5.5	54	32.90 [31.00, 34.80]
Frass 2011	67.2	4.7	59	52.9	5.1	59	14.30 [12.53, 16.07]
Javed 2022	46.37	7.44	43	36.54	8.56	43	9.83 [6.44, 13.22]
Nooh 2017	71.2	5.4	96	56.3	4.7	96	14.90 [13.47, 16.33]
Saleh 2014	75.2	6.1	40	59.7	4.2	40	15.50 [13.20, 17.80]
Tahir 2020	51.73	2.14	55	42.47	3	55	9.26 [8.29, 10.23]
Veisi 2012	45.29	8.74	35	33.38	6.77	37	11.91 [8.28, 15.54]

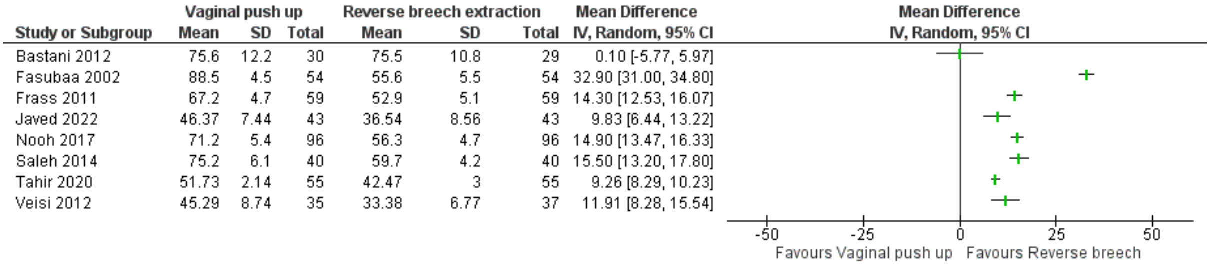

Sensitivity analysis without Saleh 2014 study data

	Vaginal push up			Reverse breech extraction			Mean Difference
Study or Subgroup	Mean	SD	Total	Mean	SD	Total	IV, Random, 95% CI
Bastani 2012	75.6	12.2	30	75.5	10.8	29	0.10 [-5.77, 5.97]
Fasubaa 2002	88.5	4.5	54	55.6	5.5	54	32.90 [31.00, 34.80]
Frass 2011	67.2	4.7	59	52.9	5.1	59	14.30 [12.53, 16.07]
Javed 2022	46.37	7.44	43	36.54	8.56	43	9.83 [6.44, 13.22]
Nooh 2017	71.2	5.4	96	56.3	4.7	96	14.90 [13.47, 16.33]
Tahir 2020	51.73	2.14	55	42.47	3	55	9.26 [8.29, 10.23]
Veisi 2012	45.29	8.74	35	33.38	6.77	37	11.91 [8.28, 15.54]

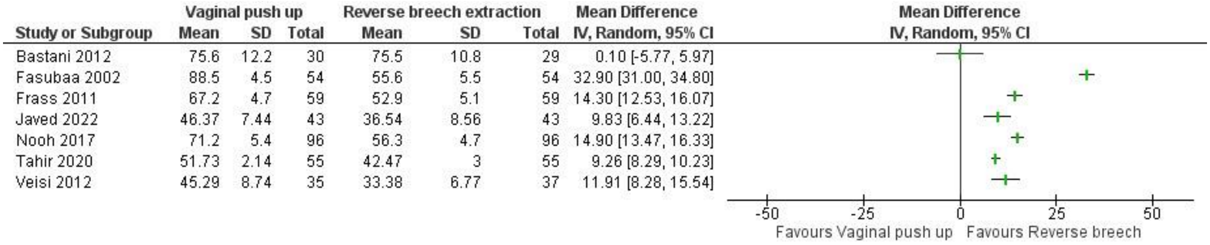

Infant birth trauma for the comparison of vaginal disimpaction versus reverse breech extraction.

Please note all the studies are randomised controlled trials. Bastani 2012 reported bony fracture and other types of fetal injury, Fasubaa 2002 and Javed 2022 defined the outcome as fetal injury, and Veisi 2012 reported femoral fracture.

	Vaginal push up		Reverse breech extraction			Risk Difference
Study or Subgroup	Events	Total	Events	Total	Weight	M-H, Random, 95% CI
Bastani 2012	0	30	0	29	30.4%	0.00 [-0.06, 0.06]
Fasubaa 2002	3	54	4	54	14.3%	-0.02 [-0.11, 0.07]
Javed 2022	0	43	1	43	32.0%	-0.02 [-0.09, 0.04]
Veisi 2012	0	35	1	37	23.3%	-0.03 [-0.10, 0.05]
**Total (95% CI)**		**162**		**163**	**100.0%**	**-0.02 [-0.05, 0.02]**
Total events	3		6			

Heterogeneity: Tau² = 0.00; Chi² = 0.40, df = 3 (P = 0.94); I² = 0%
Test for overall effect: Z = 0.91 (P = 0.36)

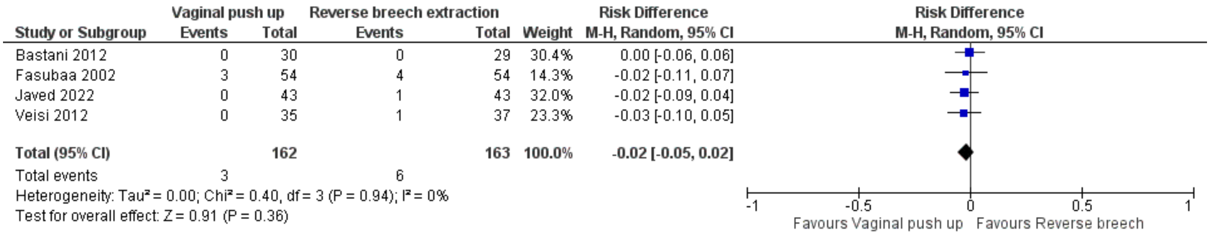

As I^2=0%, fixed effects analysis also shown below.

	Vaginal push up		Reverse breech extraction			Risk Difference
Study or Subgroup	Events	Total	Events	Total	Weight	M-H, Fixed, 95% CI
Bastani 2012	0	30	0	29	18.2%	0.00 [-0.06, 0.06]
Fasubaa 2002	3	54	4	54	33.2%	-0.02 [-0.11, 0.07]
Javed 2022	0	43	1	43	26.5%	-0.02 [-0.09, 0.04]
Veisi 2012	0	35	1	37	22.1%	-0.03 [-0.10, 0.05]
**Total (95% CI)**		**162**		**163**	**100.0%**	**-0.02 [-0.06, 0.02]**
Total events	3		6			

Heterogeneity: Chi² = 0.40, df = 3 (P = 0.94); I² = 0%
Test for overall effect: Z = 0.88 (P = 0.38)

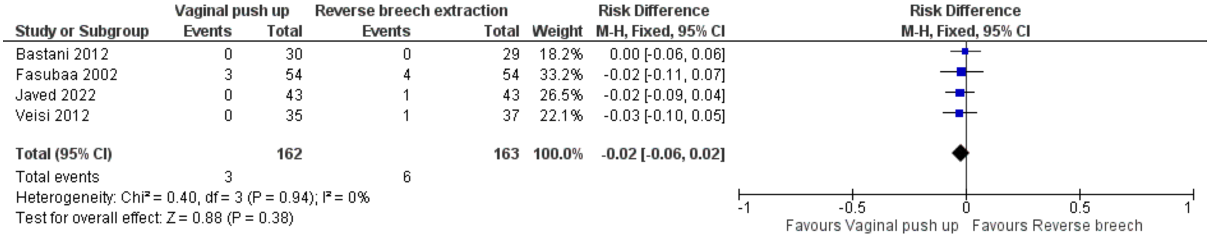

Apgar score at five minutes for the comparison of vaginal disimpaction versus reverse breech extraction.

Please note the overall pooled estimate has not been included due to very high levels of heterogeneity (I^2=98%). All the studies are randomised controlled trials.

	Vaginal push up			Reverse breech extraction				Mean Difference
**Study or Subgroup**	**Mean**	**SD**	**Total**	**Mean**	**SD**	**Total**	**Weight**	**IV, Random, 95% CI**
Bastani 2012	7.7	0.5	30	7.6	0.6	29		0.10 [-0.18, 0.38]
Fasubaa 2002	7.8	0.2	54	9	0.2	54		-1.20 [-1.28, -1.12]
Veisi 2012	9.54	0.63	35	9.51	0.69	37		0.03 [-0.27, 0.33]

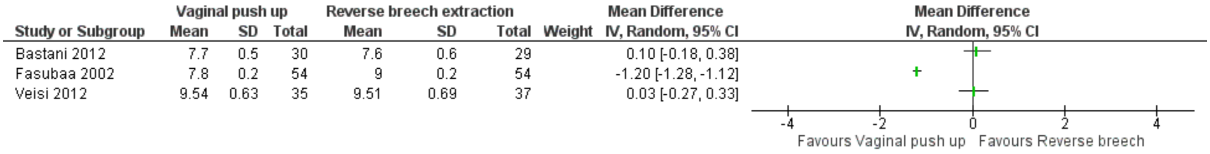

Apgar score <7 at five minutes for the comparison of vaginal disimpaction versus reverse breech extraction.

Please note all the studies are randomised controlled trials.

	Vaginal push up		Reverse breech extraction			Risk Ratio
**Study or Subgroup**	**Events**	**Total**	**Events**	**Total**	**Weight**	**M-H, Random, 95% CI**
Frass 2011	14	59	12	59	52.3%	1.17 [0.59, 2.31]
Nooh 2017	21	96	8	96	47.7%	2.63 [1.22, 5.63]
**Total (95% CI)**		**155**		**155**	**100.0%**	**1.72 [0.77, 3.82]**
Total events	35		20			
Heterogeneity: Tau² = 0.20; Chi² = 2.44, df = 1 (P = 0.12); I² = 59%						
Test for overall effect: Z = 1.33 (P = 0.18)						

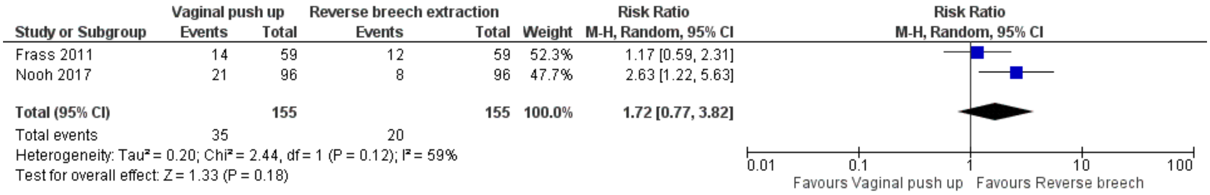

Maternal blood transfusion for the comparison of vaginal disimpaction versus reverse breech extraction.

Please note all the studies are randomised controlled trials. In Nooh 2017 in the Reverse breech extraction group n = 2 were intra-operative blood transfusions and n = 1 was post-operative, whereas in the Vaginal push group n = 11 were intra-operative blood transfusions and n = 3 were post-operative. In Saleh 2014 in the Vaginal disimpaction group, n = 10 blood transfusions were intraoperative and n = 7 were postoperative, whilst in the Reverse breech extraction group, n = 2 were intraoperative blood transfusions and n = 4 were postoperative blood transfusions.

	Vaginal push up		Reverse breech extraction			Risk Ratio
Study or Subgroup	Events	Total	Events	Total	Weight	M-H, Random, 95% CI
Bastani 2012	3	30	1	29	6.8%	2.90 [0.32, 26.30]
Frass 2011	6	59	4	59	22.3%	1.50 [0.45, 5.04]
Nooh 2017	14	96	3	96	22.3%	4.67 [1.39, 15.72]
Saleh 2014	17	40	6	40	48.7%	2.83 [1.25, 6.44]
**Total (95% CI)**		**225**		**224**	**100.0%**	**2.75 [1.55, 4.88]**
Total events	40		14			

Heterogeneity: Tau² = 0.00; Chi² = 1.71, df = 3 (P = 0.63); I² = 0%
Test for overall effect: Z = 3.46 (P = 0.0005)

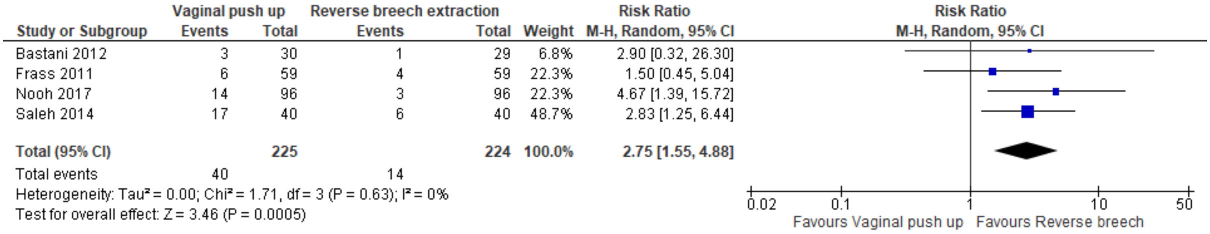

As I^2=0%, fixed effects analysis also shown below.

	Vaginal push up		Reverse breech extraction			Risk Ratio
Study or Subgroup	Events	Total	Events	Total	Weight	M-H, Fixed, 95% CI
Bastani 2012	3	30	1	29	7.3%	2.90 [0.32, 26.30]
Frass 2011	6	59	4	59	28.5%	1.50 [0.45, 5.04]
Nooh 2017	14	96	3	96	21.4%	4.67 [1.39, 15.72]
Saleh 2014	17	40	6	40	42.8%	2.83 [1.25, 6.44]
**Total (95% CI)**		**225**		**224**	**100.0%**	**2.85 [1.62, 5.03]**
Total events	40		14			

Heterogeneity: Chi² = 1.71, df = 3 (P = 0.63); I² = 0%
Test for overall effect: Z = 3.61 (P = 0.0003)

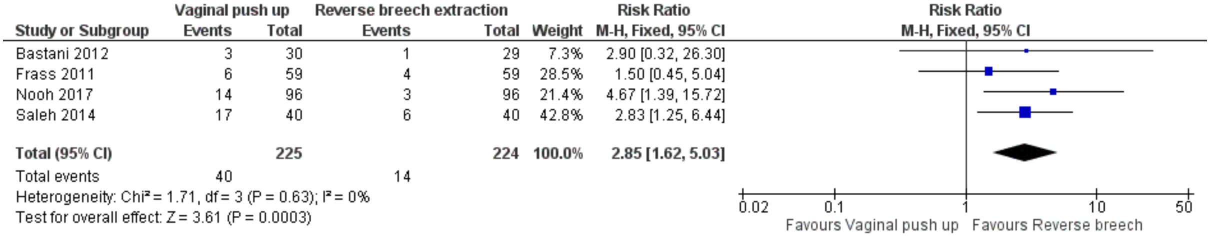

Sensitivity analysis without Saleh 2014 study data

	Vaginal push up		Reverse breech extraction			Risk Ratio
Study or Subgroup	Events	Total	Events	Total	Weight	M-H, Random, 95% CI
Bastani 2012	3	30	1	29	13.2%	2.90 [0.32, 26.30]
Frass 2011	6	59	4	59	43.5%	1.50 [0.45, 5.04]
Nooh 2017	14	96	3	96	43.4%	4.67 [1.39, 15.72]
**Total (95% CI)**		**185**		**184**	**100.0%**	**2.68 [1.20, 5.95]**
Total events	23		8			

Heterogeneity: Tau² = 0.00; Chi² = 1.71, df = 2 (P = 0.42); I² = 0%
Test for overall effect: Z = 2.41 (P = 0.02)

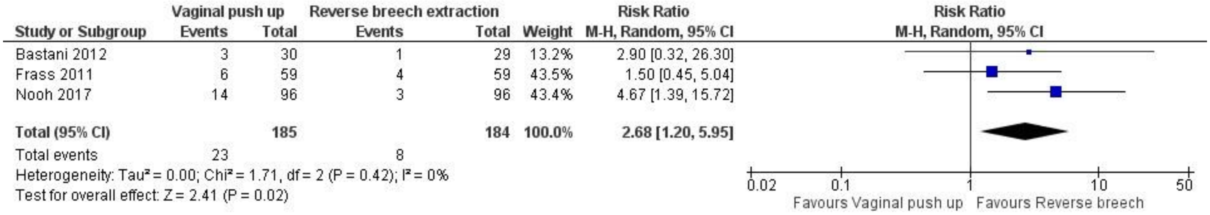

Uterine incision extension into cervix or vagina for the comparison of vaginal disimpaction versus reverse breech extraction.

Please note all the studies are randomised controlled trials. The data from Bastani 2012 include both uterine incision extension to vagina or need for J incision as these were not reported separately. Fasubaa 2002 reported uterine incision extension to vagina.

	Vaginal push up		Reverse breech extraction			Risk Ratio
Study or Subgroup	Events	Total	Events	Total	Weight	M-H, Random, 95% CI
Bastani 2012	3	30	4	29	50.9%	0.72 [0.18, 2.96]
Fasubaa 2002	9	54	2	54	49.1%	4.50 [1.02, 19.87]
**Total (95% CI)**		**84**		**83**	**100.0%**	**1.78 [0.29, 10.85]**
Total events	12		6			
Heterogeneity: Tau² = 1.16; Chi² = 3.13, df = 1 (P = 0.08); I² = 68%						
Test for overall effect: Z = 0.62 (P = 0.53)						

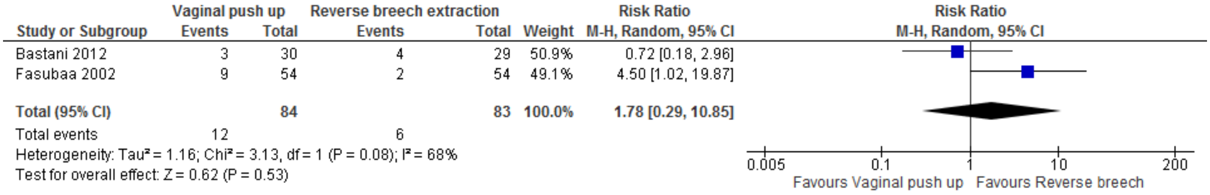

Injury to the urinary tract for the comparison of vaginal disimpaction versus reverse breech extraction.

Please note all the studies are randomised controlled trials. Bastani 2012 reported ureteral / bladder injury, hypogastric vessel ligation or hysterectomy. Frass 2011 did not fully report this outcome, but the authors stated that "no ureteral or bladder involvement was observed" (p. 1263). All the events in Nooh 2017, Saleh 2014, Veisi 2012 and Javed 2022 were bladder injuries.

	Vaginal push up		Reverse breech extraction			Risk Difference
Study or Subgroup	Events	Total	Events	Total	Weight	M-H, Random, 95% CI
Bastani 2012	0	30	0	29	9.6%	0.00 [-0.06, 0.06]
Frass 2011	0	59	0	59	36.7%	0.00 [-0.03, 0.03]
Javed 2022	0	43	0	43	19.8%	0.00 [-0.04, 0.04]
Nooh 2017	5	96	2	96	13.9%	0.03 [-0.02, 0.08]
Saleh 2014	2	40	0	40	6.0%	0.05 [-0.03, 0.13]
Veisi 2012	0	35	0	37	14.0%	0.00 [-0.05, 0.05]
**Total (95% CI)**		**303**		**304**	**100.0%**	**0.01 [-0.01, 0.03]**
Total events	7		2			

Heterogeneity: Tau² = 0.00; Chi² = 3.11, df = 5 (P = 0.68); I² = 0%
Test for overall effect: Z = 0.73 (P = 0.47)

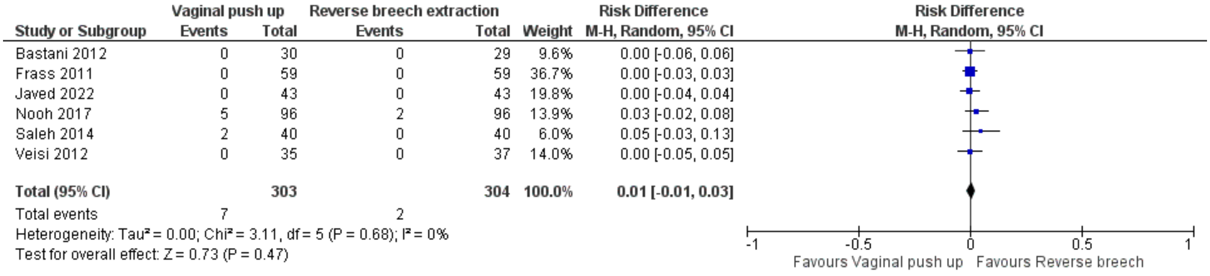

As I^2=0%, fixed effects analysis also shown below.

	Vaginal push up		Reverse breech extraction			Risk Difference
Study or Subgroup	Events	Total	Events	Total	Weight	M-H, Fixed, 95% CI
Bastani 2012	0	30	0	29	9.7%	0.00 [-0.06, 0.06]
Frass 2011	0	59	0	59	19.4%	0.00 [-0.03, 0.03]
Javed 2022	0	43	0	43	14.2%	0.00 [-0.04, 0.04]
Nooh 2017	5	96	2	96	31.6%	0.03 [-0.02, 0.08]
Saleh 2014	2	40	0	40	13.2%	0.05 [-0.03, 0.13]
Veisi 2012	0	35	0	37	11.9%	0.00 [-0.05, 0.05]
**Total (95% CI)**		**303**		**304**	**100.0%**	**0.02 [-0.01, 0.04]**
Total events	7		2			

Heterogeneity: Chi² = 3.11, df = 5 (P = 0.68); I² = 0%
Test for overall effect: Z = 1.36 (P = 0.17)

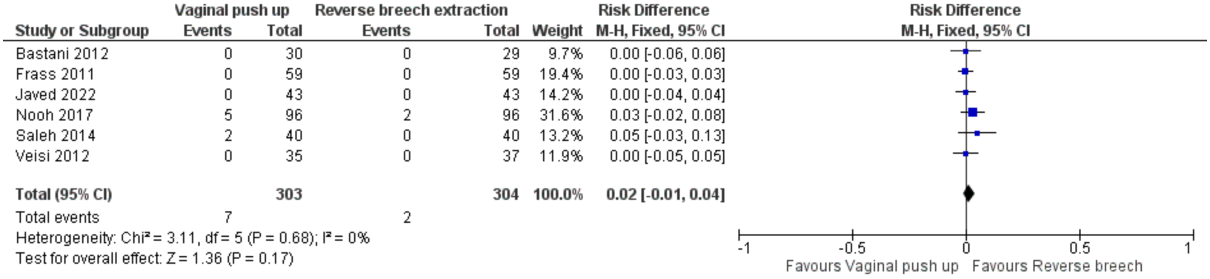

Sensitivity analysis without Saleh 2014 study data

	Vaginal push up		Reverse breech extraction			Risk Difference
Study or Subgroup	Events	Total	Events	Total	Weight	M-H, Fixed, 95% CI
Bastani 2012	0	30	0	29	11.2%	0.00 [-0.06, 0.06]
Frass 2011	0	59	0	59	22.4%	0.00 [-0.03, 0.03]
Javed 2022	0	43	0	43	16.3%	0.00 [-0.04, 0.04]
Nooh 2017	5	96	2	96	36.4%	0.03 [-0.02, 0.08]
Veisi 2012	0	35	0	37	13.7%	0.00 [-0.05, 0.05]
**Total (95% CI)**		**263**		**264**	**100.0%**	**0.01 [-0.01, 0.04]**
Total events	5		2			

Heterogeneity: Chi² = 1.57, df = 4 (P = 0.81); I² = 0%
Test for overall effect: Z = 0.92 (P = 0.36)

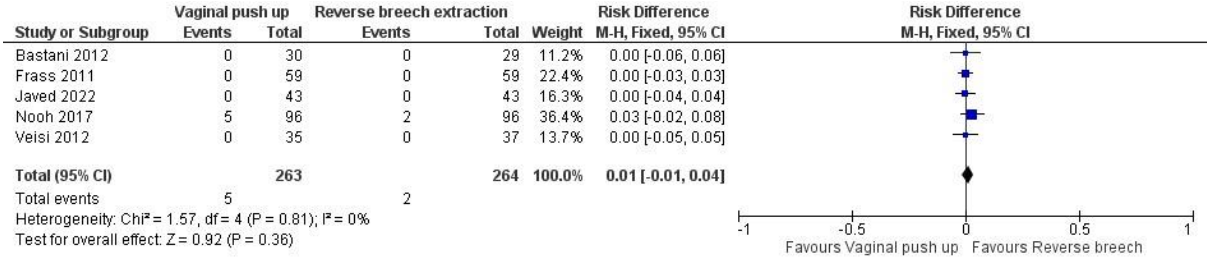

Wound infection for the comparison of vaginal disimpaction versus reverse breech extraction.

Please note all the studies are randomised controlled trials. Veisi 2012 and Javed 2022 reported wound complication.

	Vaginal push up		Reverse breech extraction			Risk Ratio
**Study or Subgroup**	**Events**	**Total**	**Events**	**Total**	**Weight**	**M-H, Random, 95% CI**
Bastani 2012	4	30	1	29	8.8%	3.87 [0.46, 32.57]
Fasubaa 2002	15	54	18	54	39.1%	0.83 [0.47, 1.48]
Frass 2011	4	59	3	59	15.9%	1.33 [0.31, 5.70]
Javed 2022	2	43	1	43	7.4%	2.00 [0.19, 21.24]
Nooh 2017	12	96	2	96	15.7%	6.00 [1.38, 26.09]
Saleh 2014	2	40	1	40	7.4%	2.00 [0.19, 21.18]
Veisi 2012	1	35	1	37	5.7%	1.06 [0.07, 16.26]
**Total (95% CI)**		**357**		**358**	**100.0%**	**1.62 [0.81, 3.23]**
Total events	40		27			

Heterogeneity: $Tau^2 = 0.23$; $Chi^2 = 8.34$, $df = 6$ ($P = 0.21$); $I^2 = 28\%$
Test for overall effect: $Z = 1.36$ ($P = 0.17$)

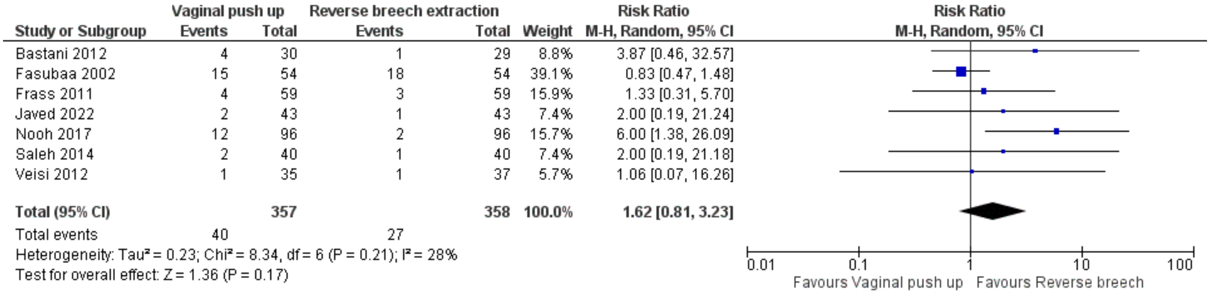

Sensitivity analysis without Saleh 2014 study data

	Vaginal push up		Reverse breech extraction			Risk Ratio
**Study or Subgroup**	**Events**	**Total**	**Events**	**Total**	**Weight**	**M-H, Random, 95% CI**
Bastani 2012	4	30	1	29	10.6%	3.87 [0.46, 32.57]
Fasubaa 2002	15	54	18	54	37.2%	0.83 [0.47, 1.48]
Frass 2011	4	59	3	59	18.1%	1.33 [0.31, 5.70]
Javed 2022	2	43	1	43	9.0%	2.00 [0.19, 21.24]
Nooh 2017	12	96	2	96	17.8%	6.00 [1.38, 26.09]
Veisi 2012	1	35	1	37	7.1%	1.06 [0.07, 16.26]
**Total (95% CI)**		**317**		**318**	**100.0%**	**1.67 [0.76, 3.70]**
Total events	38		26			

Heterogeneity: $Tau^2 = 0.35$; $Chi^2 = 8.18$, $df = 5$ ($P = 0.15$); $I^2 = 39\%$
Test for overall effect: $Z = 1.27$ ($P = 0.20$)

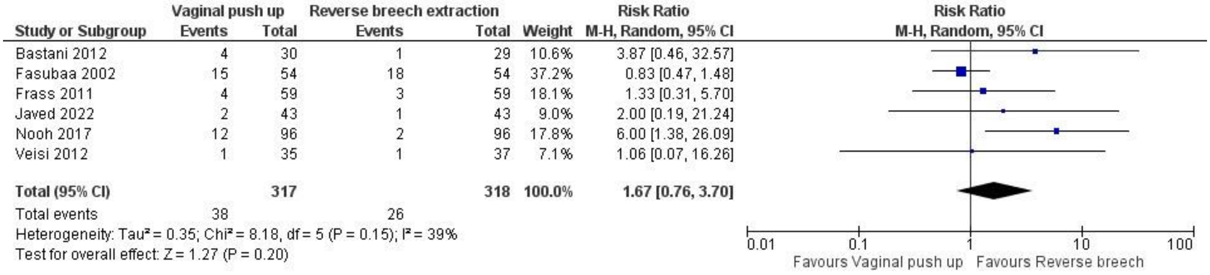

Endometritis for the comparison of vaginal disimpaction versus reverse breech extraction.

Please note all the studies are randomised controlled trials.

Study or Subgroup	Vaginal push up Events	Vaginal push up Total	Reverse breech extraction Events	Reverse breech extraction Total	Weight	Risk Ratio M-H, Random, 95% CI
Fasubaa 2002	31	54	19	54	83.0%	1.63 [1.06, 2.51]
Frass 2011	8	59	7	59	17.0%	1.14 [0.44, 2.95]
**Total (95% CI)**		**113**		**113**	**100.0%**	**1.54 [1.04, 2.27]**
Total events	39		26			

Heterogeneity: Tau² = 0.00; Chi² = 0.46, df = 1 (P = 0.50); I² = 0%
Test for overall effect: Z = 2.15 (P = 0.03)

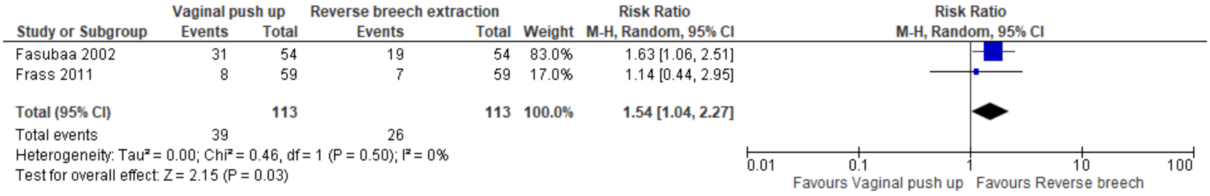

As I^2=0%, fixed effects analysis also shown below.

Study or Subgroup	Vaginal push up Events	Vaginal push up Total	Reverse breech extraction Events	Reverse breech extraction Total	Weight	Risk Ratio M-H, Fixed, 95% CI
Fasubaa 2002	31	54	19	54	73.1%	1.63 [1.06, 2.51]
Frass 2011	8	59	7	59	26.9%	1.14 [0.44, 2.95]
**Total (95% CI)**		**113**		**113**	**100.0%**	**1.50 [1.01, 2.23]**
Total events	39		26			

Heterogeneity: Chi² = 0.46, df = 1 (P = 0.50); I² = 0%
Test for overall effect: Z = 2.01 (P = 0.04)

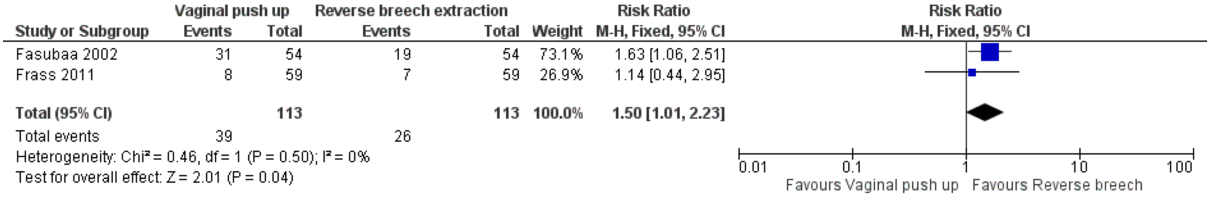

Post-partum pyrexia/maternal sepsis for the comparison of vaginal disimpaction versus reverse breech extraction.

Please note all the studies are randomised controlled trials. Bastani 2012, Veisi 2012 and Javed 2022 reported postpartum fever.

	Vaginal push up		Reverse breech extraction			Risk Ratio
**Study or Subgroup**	**Events**	**Total**	**Events**	**Total**	**Weight**	**M-H, Random, 95% CI**
Bastani 2012	16	30	3	29	29.3%	5.16 [1.68, 15.84]
Javed 2022	5	43	3	43	23.2%	1.67 [0.42, 6.54]
Nooh 2017	19	96	3	96	27.6%	6.33 [1.94, 20.70]
Veisi 2012	3	35	3	37	19.9%	1.06 [0.23, 4.89]
**Total (95% CI)**		**204**		**205**	**100.0%**	**3.06 [1.34, 6.98]**
Total events	43		12			

Heterogeneity: Tau² = 0.28; Chi² = 4.93, df = 3 (P = 0.18); I² = 39%
Test for overall effect: Z = 2.66 (P = 0.008)

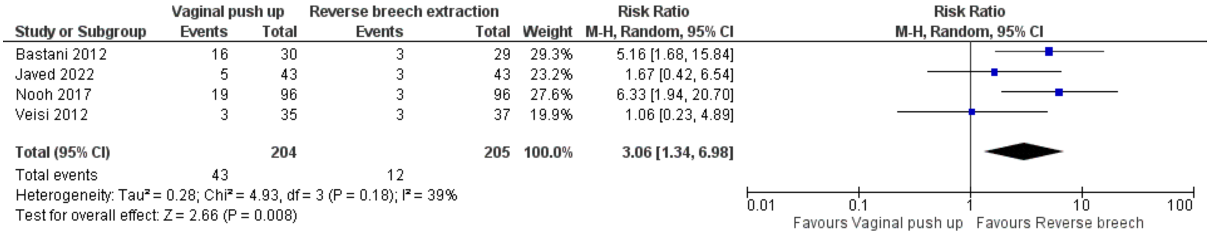

Maternal duration of hospital stay (days) for the comparison of vaginal disimpaction versus reverse breech extraction.

Please note the overall pooled estimate has not been included due to very high levels of heterogeneity (I^2=94%). All studies are randomised controlled trials. Saleh 2014 did not explicitly state the unit of measure for this outcome but we have assumed that the outcome is measured in days.

	Vaginal push up			Reverse breech extraction			Mean Difference	Mean Difference
Study or Subgroup	Mean	SD	Total	Mean	SD	Total	IV, Random, 95% CI	IV, Random, 95% CI
Bastani 2012	2.8	0.6	30	2.9	0.6	29	-0.10 [-0.41, 0.21]	
Fasubaa 2002	14.5	2.5	54	11.4	1.2	54	3.10 [2.36, 3.84]	
Frass 2011	6.6	1.2	59	6.1	1.7	59	0.50 [-0.03, 1.03]	
Nooh 2017	3.9	1.2	96	3.1	1.5	96	0.80 [0.42, 1.18]	
Saleh 2014	3.8	1.3	40	3.2	1.4	40	0.60 [0.01, 1.19]	

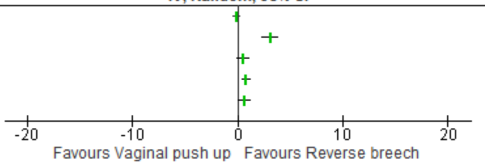

Sensitivity analysis without Saleh 2014 study data

	Vaginal push up			Reverse breech extraction			Mean Difference	Mean Difference
Study or Subgroup	Mean	SD	Total	Mean	SD	Total	IV, Random, 95% CI	IV, Random, 95% CI
Bastani 2012	2.8	0.6	30	2.9	0.6	29	-0.10 [-0.41, 0.21]	
Fasubaa 2002	14.5	2.5	54	11.4	1.2	54	3.10 [2.36, 3.84]	
Frass 2011	6.6	1.2	59	6.1	1.7	59	0.50 [-0.03, 1.03]	
Nooh 2017	3.9	1.2	96	3.1	1.5	96	0.80 [0.42, 1.18]	

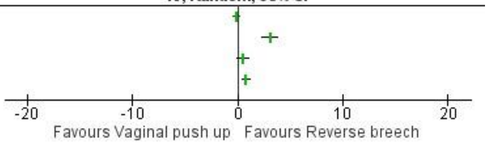

NICU admission for the comparison of vaginal disimpaction versus reverse breech extraction.

Please note the overall pooled estimate has not been included due to very high levels of heterogeneity (I^2=90%). All studies are randomised controlled trials. Frass 2011 reported admission to nursery.

	Vaginal push up		Reverse breech extraction		Risk Difference
Study or Subgroup	Events	Total	Events	Total	M-H, Random, 95% CI
Bastani 2012	0	30	0	29	0.00 [-0.06, 0.06]
Fasubaa 2002	39	54	14	54	0.46 [0.30, 0.63]
Frass 2011	13	59	11	59	0.03 [-0.11, 0.18]
Javed 2022	0	43	1	43	-0.02 [-0.09, 0.04]
Nooh 2017	15	96	7	96	0.08 [-0.01, 0.17]
Saleh 2014	7	40	4	40	0.07 [-0.08, 0.23]
Veisi 2012	0	35	0	37	0.00 [-0.05, 0.05]

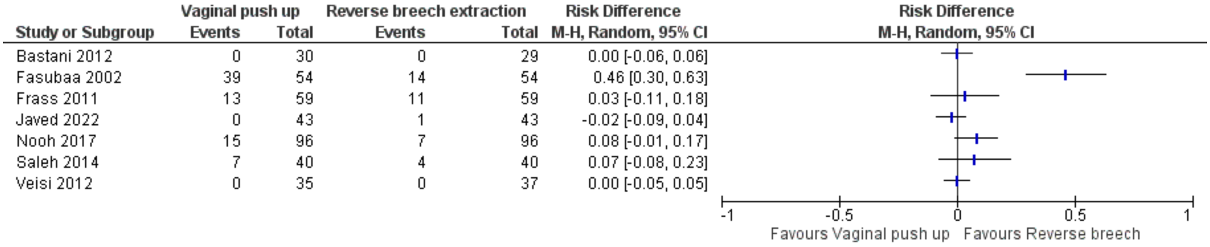

Sensitivity analysis without Saleh 2014 study data

	Vaginal push up		Reverse breech extraction		Risk Difference
Study or Subgroup	Events	Total	Events	Total	M-H, Random, 95% CI
Bastani 2012	0	30	0	29	0.00 [-0.06, 0.06]
Fasubaa 2002	39	54	14	54	0.46 [0.30, 0.63]
Frass 2011	13	59	11	59	0.03 [-0.11, 0.18]
Javed 2022	0	43	1	43	-0.02 [-0.09, 0.04]
Nooh 2017	15	96	7	96	0.08 [-0.01, 0.17]
Veisi 2012	0	35	0	37	0.00 [-0.05, 0.05]

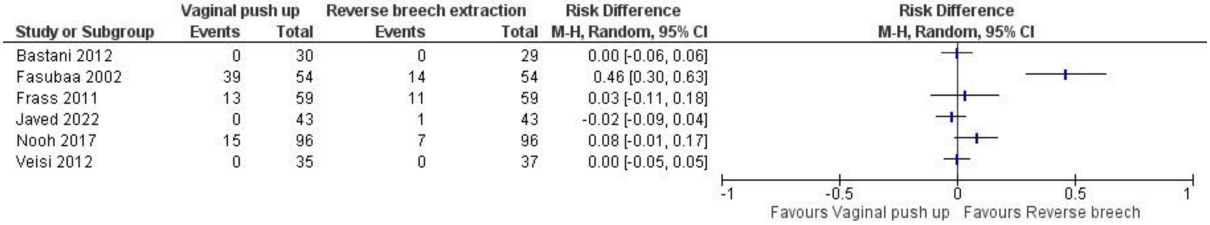

Neonatal death for the comparison of vaginal disimpaction versus reverse breech extraction.

Please note all the studies are randomised controlled trials. In Bastani 2012 this outcome was defined as fetal death, in Fasubaa 2002 this outcome was defined as early neonatal death, and in Nooh 2017 this outcome was defined as neonatal death. None of the studies clearly reported the time frame for this outcome.

	Vaginal push up		Reverse breech extraction			Risk Difference
Study or Subgroup	Events	Total	Events	Total	Weight	M-H, Random, 95% CI
Bastani 2012	0	30	0	29	36.4%	0.00 [-0.06, 0.06]
Fasubaa 2002	13	54	7	54	12.2%	0.11 [-0.03, 0.26]
Nooh 2017	3	96	0	96	51.3%	0.03 [-0.01, 0.07]
**Total (95% CI)**		**180**		**179**	**100.0%**	**0.03 [-0.03, 0.09]**
Total events	16		7			

Heterogeneity: Tau² = 0.00; Chi² = 3.92, df = 2 (P = 0.14); I² = 49%

Test for overall effect: Z = 1.04 (P = 0.30)

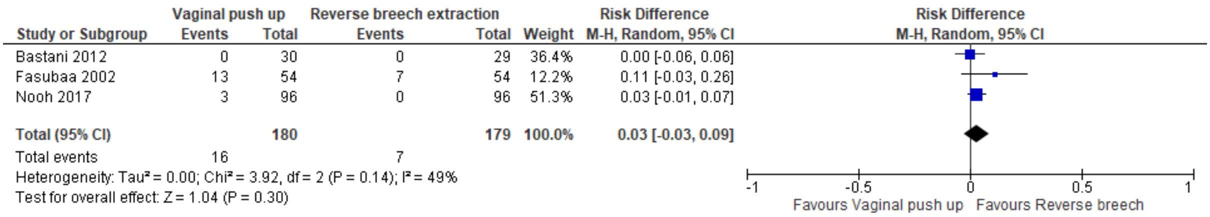

Vaginal disimpaction versus Patwardhan method

Uterine incision extensions (combined) for the comparison of vaginal disimpaction versus Patwardhan method.

All studies are non-randomised. The pooled estimate is shown but please note the high level of heterogeneity (I^2=79%).

	Vaginal push up		Patwardhan method			Risk Ratio
**Study or Subgroup**	**Events**	**Total**	**Events**	**Total**	**Weight**	**M-H, Random, 95% CI**
Beeresh 2016	18	52	4	46	18.9%	3.98 [1.45, 10.91]
Bhattacharya 2020	8	50	1	50	11.4%	8.00 [1.04, 61.62]
Keepanasseril 2019	55	221	20	77	22.7%	0.96 [0.62, 1.49]
Lal 2018	15	64	2	56	15.6%	6.56 [1.57, 27.45]
Lenz 2019	29	82	5	55	19.8%	3.89 [1.60, 9.43]
Rakholia 2019	10	54	1	62	11.5%	11.48 [1.52, 86.81]
**Total (95% CI)**		**523**		**346**	**100.0%**	**3.79 [1.46, 9.86]**
Total events	135		33			

Heterogeneity: Tau² = 0.99; Chi² = 23.57, df = 5 (P = 0.0003); I² = 79%
Test for overall effect: Z = 2.73 (P = 0.006)

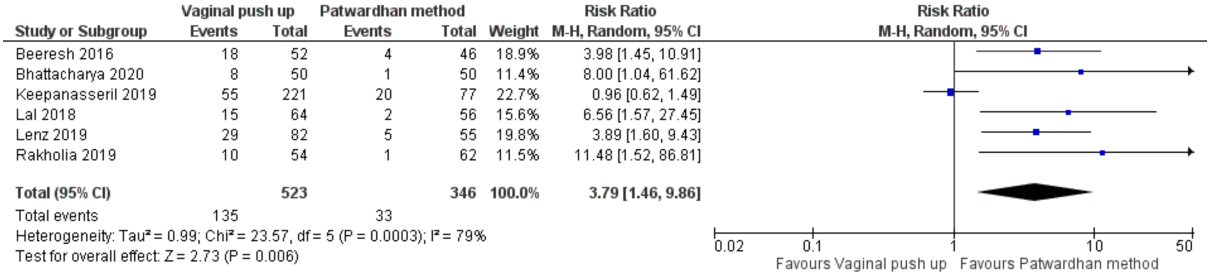

Sensitivity analysis without Beeresh 2016, Bhattacharya 2020, Lal 2018, Rakholia 2019 study data

	Vaginal push up		Patwardhan method			Risk Ratio
**Study or Subgroup**	**Events**	**Total**	**Events**	**Total**	**Weight**	**M-H, Random, 95% CI**
Keepanasseril 2019	55	221	20	77	53.7%	0.96 [0.62, 1.49]
Lenz 2019	29	82	5	55	46.3%	3.89 [1.60, 9.43]
**Total (95% CI)**		**303**		**132**	**100.0%**	**1.83 [0.45, 7.50]**
Total events	84		25			

Heterogeneity: Tau² = 0.91; Chi² = 8.16, df = 1 (P = 0.004); I² = 88%
Test for overall effect: Z = 0.84 (P = 0.40)

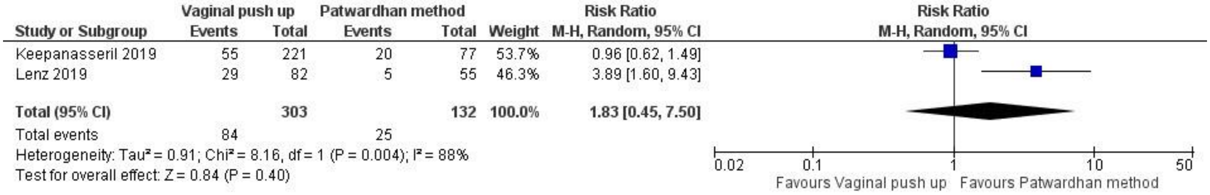

Uterine incision extension on lower segment for the comparison of vaginal disimpaction versus Patwardhan method.

Please note all studies are non-randomised. In Keepanasserill 2019 in the Vaginal disimpaction group n = 42 and 10, were extensions to the lower uterine flap and the uterine artery, respectively, whereas in the Patwardhan method group n = 9 and 5 were extensions to the lower uterine flap and the uterine artery, respectively. Bhattacharya 2020, Beeresh 2016 and Rakholia 2019 reported 'uterine incision extension'. Lal 2018 reported data for 'uterine incision extension' and 'uterine artery injury' combined.

	Vaginal push up		Patwardhan method			Risk Ratio
Study or Subgroup	Events	Total	Events	Total	Weight	M-H, Random, 95% CI
Battycharya 2020	8	50	1	50	9.4%	8.00 [1.04, 61.62]
Beeresh 2016	18	52	4	46	19.5%	3.98 [1.45, 10.91]
Keepanasseril 2019	52	221	14	77	26.1%	1.29 [0.76, 2.20]
Lal 2018	15	64	2	56	14.4%	6.56 [1.57, 27.45]
Lenz 2019	29	82	5	55	21.2%	3.89 [1.60, 9.43]
Rakholia 2019	10	54	1	62	9.5%	11.48 [1.52, 86.81]
**Total (95% CI)**		**523**		**346**	**100.0%**	**3.75 [1.76, 7.96]**
Total events	132		27			

Heterogeneity: Tau² = 0.49; Chi² = 13.51, df = 5 (P = 0.02); I² = 63%
Test for overall effect: Z = 3.44 (P = 0.0006)

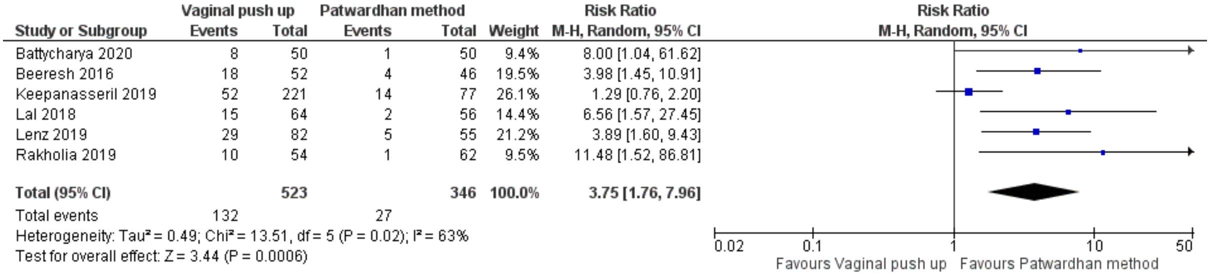

Sensitivity analysis without Beeresh 2016, Bhattacharya 2020, Lal 2018, Rakholia 2019 study data

	Vaginal push up		Patwardhan method			Risk Ratio
Study or Subgroup	Events	Total	Events	Total	Weight	M-H, Random, 95% CI
Keepanasseril 2019	52	221	14	77	55.3%	1.29 [0.76, 2.20]
Lenz 2019	29	82	5	55	44.7%	3.89 [1.60, 9.43]
**Total (95% CI)**		**303**		**132**	**100.0%**	**2.12 [0.71, 6.28]**
Total events	81		19			

Heterogeneity: Tau² = 0.48; Chi² = 4.49, df = 1 (P = 0.03); I² = 78%
Test for overall effect: Z = 1.35 (P = 0.18)

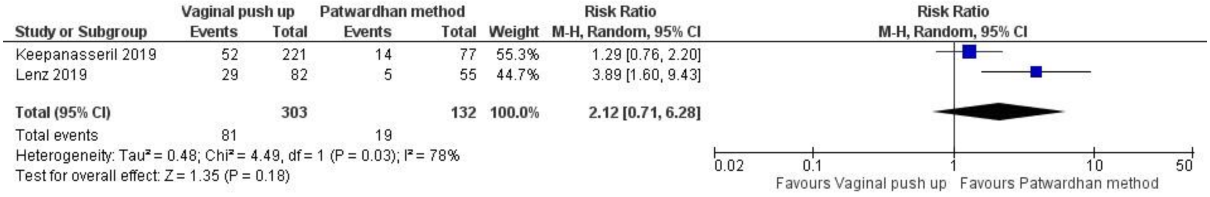

Post-partum haemorrhage (operative blood loss >1000 ml) for the comparison of vaginal disimpaction versus Patwardhan method

All studies are non-randomised. Keepanasseril reported 'post-partum haemorrhage'. Beeresh 2016 and Lal 2018 reported 'traumatic' and 'atonic' PPH separately. These data have been combined to give an overall number of events for PPH. Rakholia 2019 only reported 'traumatic PPH'.

	Vaginal push up		Patwardhan method			Risk Ratio
**Study or Subgroup**	**Events**	**Total**	**Events**	**Total**	**Weight**	**M-H, Random, 95% CI**
Beeresh 2016	18	52	4	46	26.6%	3.98 [1.45, 10.91]
Keepanasseril 2019	26	221	8	77	30.8%	1.13 [0.54, 2.39]
Lal 2018	32	64	5	56	28.8%	5.60 [2.34, 13.39]
Rakholia 2019	8	54	1	62	13.8%	9.19 [1.19, 71.11]
**Total (95% CI)**		**391**		**241**	**100.0%**	**3.35 [1.29, 8.66]**
Total events	84		18			

Heterogeneity: Tau² = 0.62; Chi² = 10.00, df = 3 (P = 0.02); I² = 70%
Test for overall effect: Z = 2.49 (P = 0.01)

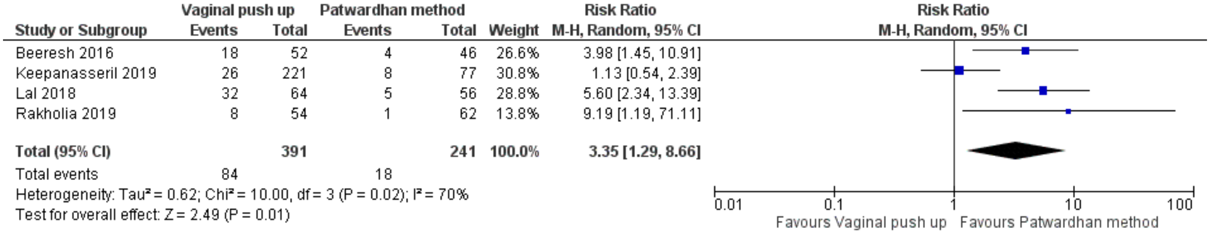

Sensitivity analysis without Beeresh 2016, Bhattacharya 2020, Lal 2018, Rakholia 2019 study data

	Vaginal push up		Patwardhan method			Risk Ratio
**Study or Subgroup**	**Events**	**Total**	**Events**	**Total**	**Weight**	**M-H, Random, 95% CI**
Keepanasseril 2019	26	221	8	77	100.0%	1.13 [0.54, 2.39]
**Total (95% CI)**		**221**		**77**	**100.0%**	**1.13 [0.54, 2.39]**
Total events	26		8			

Heterogeneity: Not applicable
Test for overall effect: Z = 0.33 (P = 0.74)

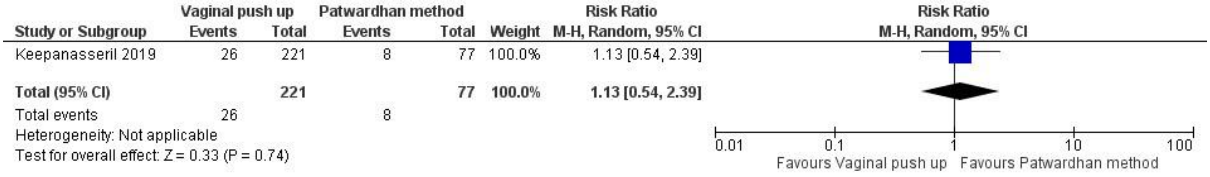

Maternal operative blood loss (ml) for the comparison of vaginal disimpaction versus Patwardhan method.

Please note the overall pooled estimate has not been included due to very high levels of heterogeneity (I^2=85%). Both studies are non-randomised.

	Vaginal push up			Patwardhan method			Mean Difference
Study or Subgroup	Mean	SD	Total	Mean	SD	Total	IV, Random, 95% CI
Keepanasseril 2019	647.1	250.3	221	653.2	285.08	77	-6.10 [-77.82, 65.62]
Lenz 2019	712.2	375	82	562.7	195.1	55	149.50 [53.34, 245.66]

Mean Difference
IV, Random, 95% CI

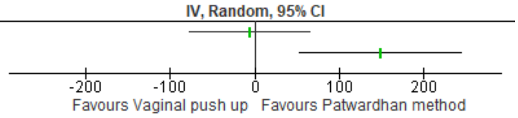

Operative time (minutes) for the comparison of vaginal disimpaction versus Patwardhan method.

Please note both studies are non-randomised.

	Vaginal push up			Patwardhan method				Mean Difference
**Study or Subgroup**	**Mean**	**SD**	**Total**	**Mean**	**SD**	**Total**	**Weight**	**IV, Random, 95% CI**
Keepanasseril 2019	69	22.3	221	67.3	23.7	77	50.0%	1.70 [-4.36, 7.76]
Lenz 2019	44.8	16.7	82	38.3	18.4	55	50.0%	6.50 [0.44, 12.56]
**Total (95% CI)**			**303**			**132**	**100.0%**	**4.10 [-0.61, 8.80]**

Heterogeneity: Tau² = 1.97; Chi² = 1.21, df = 1 (P = 0.27); I² = 17%
Test for overall effect: Z = 1.71 (P = 0.09)

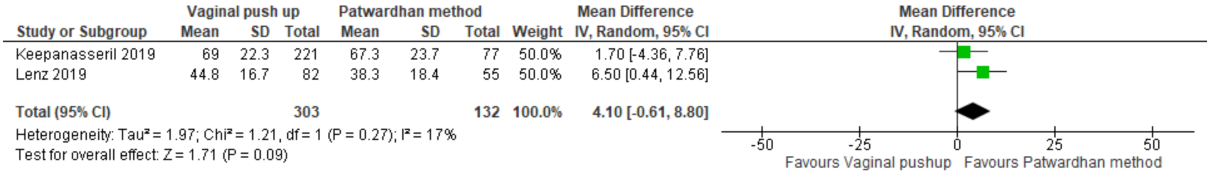

Infant birth trauma for the comparison of vaginal disimpaction versus Patwardhan method.

Please note all studies are non-randomised. Keepanasseril 2019 reported "injuries to the visceral organ injury or the fracture of long bones or the skull during the attempt to deliver the baby" (p. 608). Birth injuries included: subgaleal haemorrhage ('Vaginal disimpaction': n = 2; 'Patwardhan method': n = 1); laceration over the forehead/scalp ('Vaginal disimpaction': n = 6; 'Patwardhan method': n = 1); fracture of a rib and humerus ('Vaginal disimpaction': n=0; 'Patwardhan method': n = 2). In Lenz 2019 in the push group both events were due to perinatal skull fractures that resulted in neonatal admissions; the single event in the Patwardhan's group was a fetal humours fracture. Beeresh 2016, Bhattacharya 2020 and Lal 2018 reported 'fetal injury' without any further details. As there were zero events in one of the comparison groups for Beeresh 2016 and Lal 2018, precluding the calculation of RR, we calculated Peto odds ratios (POR).

	Vaginal push up		Patwardhan method			Peto Odds Ratio
Study or Subgroup	Events	Total	Events	Total	Weight	Peto, Fixed, 95% CI
Beeresh 2016	0	52	2	46	9.6%	0.12 [0.01, 1.89]
Bhattacharya 2020	2	50	3	50	23.5%	0.66 [0.11, 3.95]
Keepanasseril 2019	8	221	4	77	43.3%	0.67 [0.18, 2.49]
Lal 2018	0	64	2	56	9.7%	0.12 [0.01, 1.88]
Lenz 2019	2	82	1	55	13.9%	1.33 [0.13, 13.64]
**Total (95% CI)**		**469**		**284**	**100.0%**	**0.52 [0.22, 1.24]**
Total events	12		12			

Heterogeneity: Chi² = 3.06, df = 4 (P = 0.55); I² = 0%
Test for overall effect: Z = 1.47 (P = 0.14)

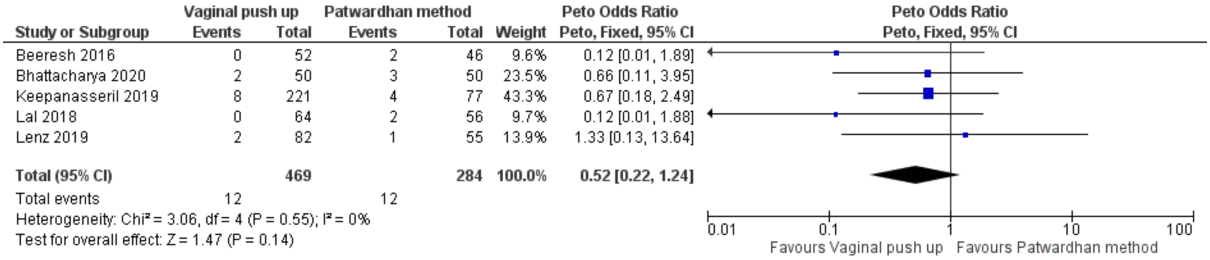

Sensitivity analysis without Beeresh 2016, Bhattacharya 2020, Lal 2018, Rakholia 2019 study data

	Vaginal push up		Patwardhan method			Peto Odds Ratio
Study or Subgroup	Events	Total	Events	Total	Weight	Peto, Fixed, 95% CI
Keepanasseril 2019	8	221	4	77	75.7%	0.67 [0.18, 2.49]
Lenz 2019	2	82	1	55	24.3%	1.33 [0.13, 13.64]
**Total (95% CI)**		**303**		**132**	**100.0%**	**0.79 [0.25, 2.48]**
Total events	10		5			

Heterogeneity: Chi² = 0.26, df = 1 (P = 0.61); I² = 0%
Test for overall effect: Z = 0.41 (P = 0.68)

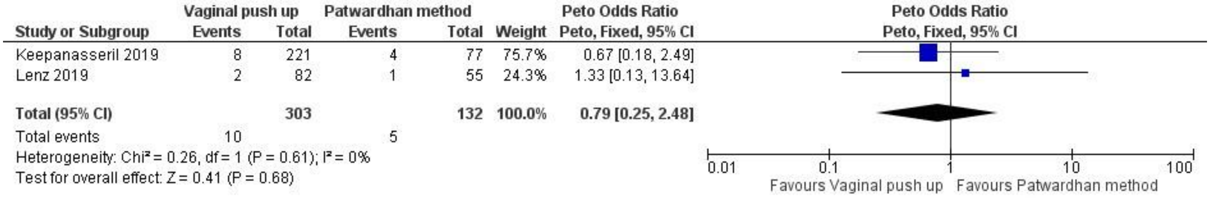

Apgar score <7 at 5 minutes for the comparison of vaginal disimpaction versus Patwardhan method.

All studies are non-randomised. Lal 2018 reported 'Apgar score' only without reporting the time at which the measurement was taken so has not been included in the analysis. As there were zero events in one of the comparison groups for Lenz 2019 precluding the calculation of RR, we calculated Peto odds ratios (POR).

	Vaginal push up		Patwardhan method			Peto Odds Ratio
Study or Subgroup	Events	Total	Events	Total	Weight	Peto, Fixed, 95% CI
Beeresh 2016	7	52	5	46	78.9%	1.27 [0.38, 4.23]
Lenz 2019	3	82	0	55	21.1%	5.45 [0.53, 55.77]
**Total (95% CI)**		**134**		**101**	**100.0%**	**1.73 [0.59, 5.03]**
Total events	10		5			

Heterogeneity: Chi² = 1.19, df = 1 (P = 0.28); I² = 16%
Test for overall effect: Z = 1.00 (P = 0.32)

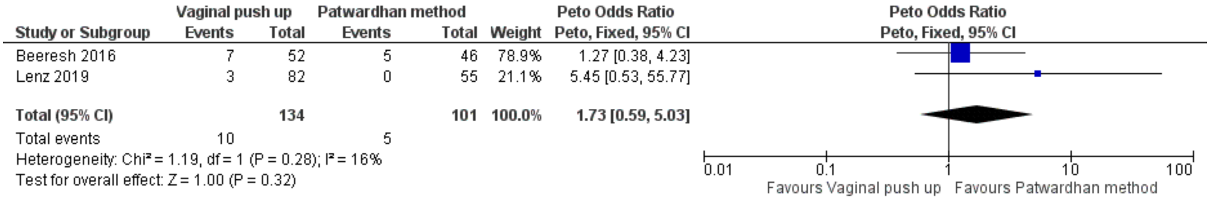

Blood transfusion for the comparison of vaginal disimpaction versus Patwardhan method.

All studies are non-randomised.

	Vaginal push up		Patwardhan method			Risk Ratio
Study or Subgroup	Events	Total	Events	Total	Weight	M-H, Random, 95% CI
Beeresh 2016	20	52	8	46	23.4%	2.21 [1.08, 4.53]
Bhattacharya 2020	19	50	12	50	25.6%	1.58 [0.86, 2.91]
Keepanasseril 2019	13	221	7	77	20.2%	0.65 [0.27, 1.56]
Lal 2018	21	64	8	56	23.1%	2.30 [1.11, 4.77]
Rakholia 2019	16	54	1	52	7.7%	15.41 [2.12, 112.05]
**Total (95% CI)**		**441**		**281**	**100.0%**	**1.85 [0.99, 3.47]**
Total events	89		36			

Heterogeneity: $Tau^2 = 0.30$; $Chi^2 = 11.04$, df = 4 (P = 0.03); $I^2 = 64\%$
Test for overall effect: Z = 1.93 (P = 0.05)

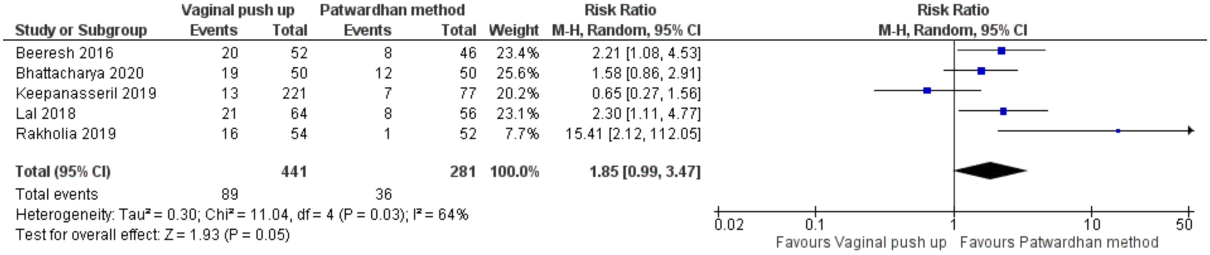

Sensitivity analysis without Beeresh 2016, Bhattacharya 2020, Lal 2018, Rakholia 2019 study data

In sensitivity analysis without studies at high risk of publication bias (Beeresh 2016, Lal 2018, Rakholia 2019, Bhattacharya 2020), only one study (Keepanaseril 2019) remains so it is not possible to present a pooled result.

	Vaginal push up		Patwardhan method			Risk Ratio
Study or Subgroup	Events	Total	Events	Total	Weight	M-H, Random, 95% CI
Keepanasseril 2019	13	221	7	77	100.0%	0.65 [0.27, 1.56]
**Total (95% CI)**		**221**		**77**	**100.0%**	**0.65 [0.27, 1.56]**
Total events	13		7			

Heterogeneity: Not applicable
Test for overall effect: Z = 0.97 (P = 0.33)

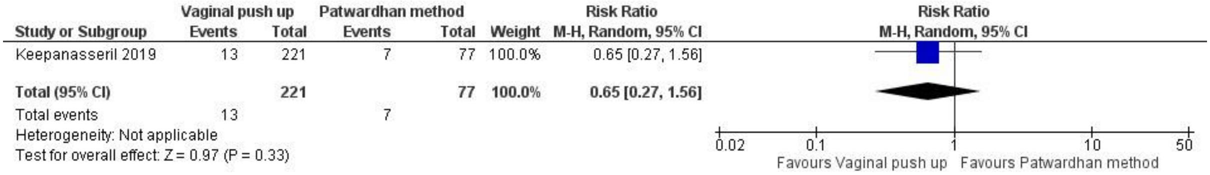

Visceral injury or hysterectomy (injury to urinary tract) for the comparison of vaginal disimpaction versus Patwardhan method

All studies are non-randomised. Keepanaseril 2019 reported 'intraoperative bowel or bladder injury'. Beeresh 2016, Lal 2018 and Bhattacharya 2020 reported 'bladder injury' without any further details. As there were zero events in one of the comparison groups for Beeresh 2016, Keepanaseril 2019 and Lal 2018, precluding the calculation of RR, we calculated Peto odds ratios (POR).

	Vaginal push up		Patwardhan method			Peto Odds Ratio
**Study or Subgroup**	**Events**	**Total**	**Events**	**Total**	**Weight**	**Peto, Fixed, 95% CI**
Beeresh 2016	2	52	0	46	16.7%	6.71 [0.41, 109.47]
Bhattacharya 2020	3	50	1	50	32.8%	2.80 [0.38, 20.52]
Keepanasseril 2019 (1)	4	221	0	77	25.7%	3.90 [0.41, 37.05]
Lal 2018	3	64	0	56	24.8%	6.73 [0.68, 66.33]
**Total (95% CI)**		**387**		**229**	**100.0%**	**4.39 [1.40, 13.73]**
Total events	12		1			

Heterogeneity: Chi² = 0.43, df = 3 (P = 0.93); I² = 0%
Test for overall effect: Z = 2.54 (P = 0.01)

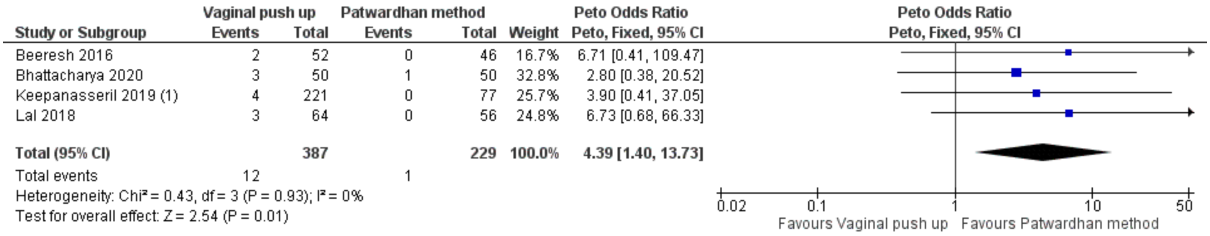

Footnotes
(1) Intraoperative bowel or bladder injury.

Sensitivity analysis without Beeresh 2016, Bhattacharya 2020, Lal 2018, Rakholia 2019 study data

In sensitivity analysis without studies at high risk of publication bias (Beeresh 2016, Lal 2018, Bhattacharya 2020), only one study (Keepanaseril 2019) remains so it is not possible to present a pooled result.

	Vaginal push up		Patwardhan method			Peto Odds Ratio
**Study or Subgroup**	**Events**	**Total**	**Events**	**Total**	**Weight**	**Peto, Fixed, 95% CI**
Keepanasseril 2019 (1)	4	221	0	77	100.0%	3.90 [0.41, 37.05]
**Total (95% CI)**		**221**		**77**	**100.0%**	**3.90 [0.41, 37.05]**
Total events	4		0			

Heterogeneity: Not applicable
Test for overall effect: Z = 1.19 (P = 0.24)

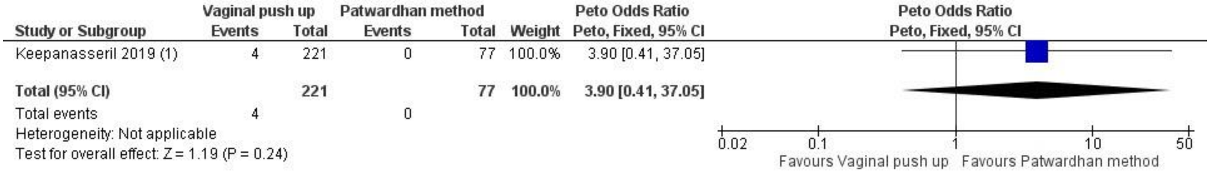

Footnotes
(1) Intraoperative bowel or bladder injury.

NICU admission for the comparison of vaginal disimpaction versus Patwardhan method.

Please note all studies are non-randomised.

	Vaginal push up		Patwardhan method			Risk Ratio
Study or Subgroup	Events	Total	Events	Total	Weight	M-H, Random, 95% CI
Beeresh 2016	11	52	9	46	15.2%	1.08 [0.49, 2.37]
Bhattacharya 2020	17	50	17	50	22.2%	1.00 [0.58, 1.73]
Keepanasseril 2019	80	221	45	77	33.3%	0.62 [0.48, 0.80]
Lal 2018	13	64	10	56	16.2%	1.14 [0.54, 2.39]
Lenz 2019	2	82	1	55	2.5%	1.34 [0.12, 14.44]
Rakholia 2019	9	54	5	62	10.5%	2.07 [0.74, 5.79]
**Total (95% CI)**		**523**		**346**	**100.0%**	**0.96 [0.65, 1.42]**
Total events	132		87			

Heterogeneity: $Tau^2 = 0.10$; $Chi^2 = 9.86$, $df = 5$ ($P = 0.08$); $I^2 = 49\%$
Test for overall effect: $Z = 0.21$ ($P = 0.83$)

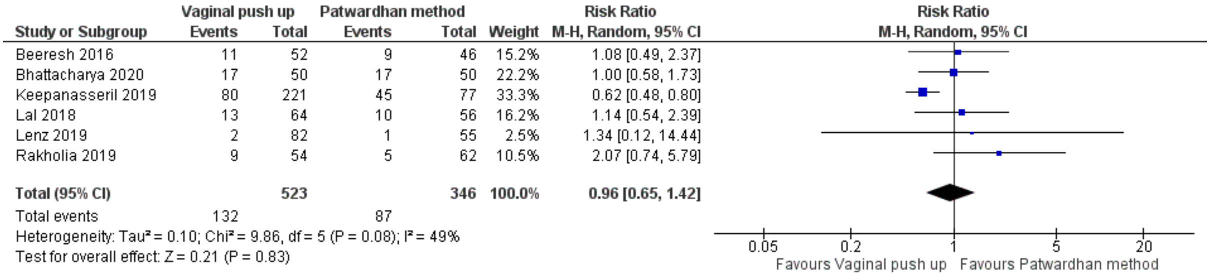

Sensitivity analysis without Beeresh 2016, Bhattacharya 2020, Lal 2018, Rakholia 2019 study data

	Vaginal push up		Patwardhan method			Risk Ratio
Study or Subgroup	Events	Total	Events	Total	Weight	M-H, Random, 95% CI
Keepanasseril 2019	80	221	45	77	98.8%	0.62 [0.48, 0.80]
Lenz 2019	2	82	1	55	1.2%	1.34 [0.12, 14.44]
**Total (95% CI)**		**303**		**132**	**100.0%**	**0.62 [0.48, 0.81]**
Total events	82		46			

Heterogeneity: $Tau^2 = 0.00$; $Chi^2 = 0.41$, $df = 1$ ($P = 0.52$); $I^2 = 0\%$
Test for overall effect: $Z = 3.60$ ($P = 0.0003$)

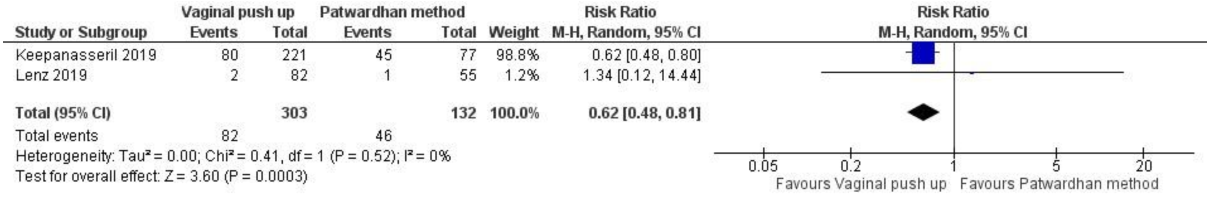

Neonatal death for the comparison of vaginal disimpaction versus Patwardhan method.

Please note all studies are non-randomised. Keepanasseril 2019 reported early neonatal death within 7 days of birth. Lenz 2019 reported death within the first 28 days of life. Bhattacharya 2020 and Rakholia 2019 gave no definition of neonatal death. Lal 2018 and Beeesh 2016 reported stillbirth only so that data has not been included in this analysis. As there were zero events in one of the comparison groups for Lenz 2019, precluding the calculation of RR, we calculated Peto odds ratios (POR).

Study or Subgroup	Vaginal push up Events	Vaginal push up Total	Patwardhan method Events	Patwardhan method Total	Weight	Peto Odds Ratio Peto, Fixed, 95% CI
Bhattacharya 2020	7	50	6	50	55.0%	1.19 [0.37, 3.80]
Keepanasseril 2019	3	221	3	77	21.8%	0.28 [0.04, 1.75]
Lenz 2019	1	82	0	55	4.6%	5.32 [0.10, 289.77]
Rakholia 2019	3	54	1	62	18.7%	3.23 [0.44, 23.68]
**Total (95% CI)**		**407**		**244**	**100.0%**	**1.12 [0.47, 2.65]**
Total events	14		10			

Heterogeneity: Chi² = 3.89, df = 3 (P = 0.27); I² = 23%
Test for overall effect: Z = 0.26 (P = 0.80)

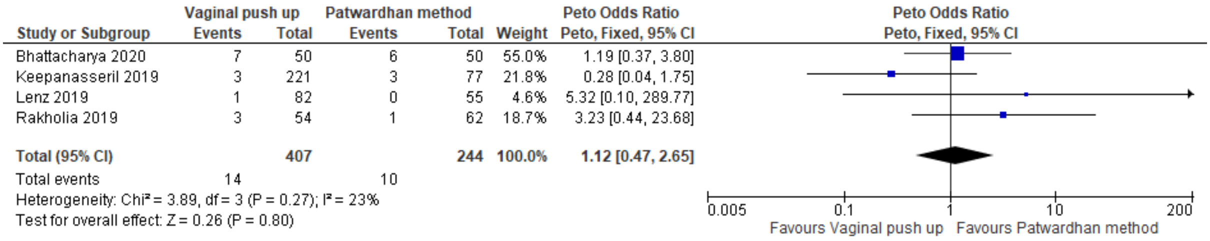

Sensitivity analysis without Beeresh 2016, Bhattacharya 2020, Lal 2018, Rakholia 2019 study data

Study or Subgroup	Vaginal push up Events	Vaginal push up Total	Patwardhan method Events	Patwardhan method Total	Weight	Peto Odds Ratio Peto, Fixed, 95% CI
Keepanasseril 2019	3	221	3	77	82.5%	0.28 [0.04, 1.75]
Lenz 2019	1	82	0	55	17.5%	5.32 [0.10, 289.77]
**Total (95% CI)**		**303**		**132**	**100.0%**	**0.47 [0.09, 2.48]**
Total events	4		3			

Heterogeneity: Chi² = 1.73, df = 1 (P = 0.19); I² = 42%
Test for overall effect: Z = 0.90 (P = 0.37)

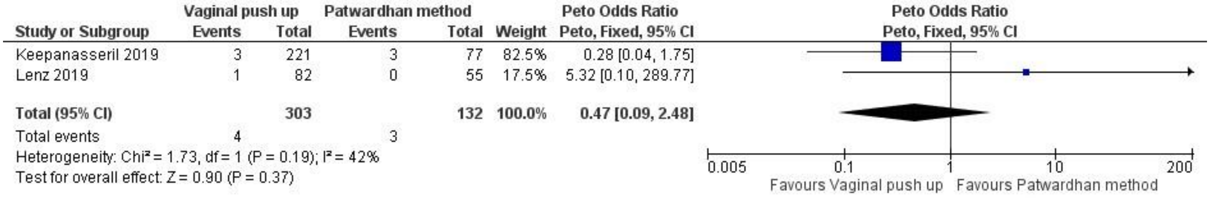

Visceral injury or hysterectomy (hysterectomy) for the comparison of vaginal disimpaction versus Patwardhan method.

All studies are non-randomised. As there were zero events in one of the comparison groups for both studies, precluding the calculation of RR, we calculated Peto odds ratios (POR).

	Vaginal push up		Patwardhan method			Peto Odds Ratio
Study or Subgroup	Events	Total	Events	Total	Weight	Peto, Fixed, 95% CI
Bhattacharya 2020	1	50	0	50	33.6%	7.39 [0.15, 372.38]
Lal 2018	2	64	0	56	66.4%	6.63 [0.41, 107.84]
**Total (95% CI)**		**114**		**106**	**100.0%**	**6.87 [0.71, 66.72]**
Total events	3		0			
Heterogeneity: Chi² = 0.00, df = 1 (P = 0.96); I² = 0%						
Test for overall effect: Z = 1.66 (P = 0.10)						

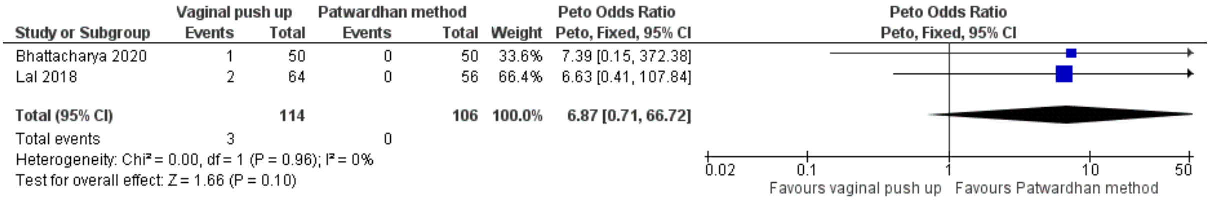

Sensitivity analysis without Beeresh 2016, Bhattacharya 2020, Lal 2018, Rakholia 2019 study data

No studies remain after removal of data for Bhattacharya and Lal

Vaginal disimpaction or reverse breech extraction versus Patwardhan method

Uterine incision extension: Incision extension on lower segment for the comparison of vaginal push or pull versus Patwardhan method.

Bhoi 2019 is an RCT. Bansiwal 2017 and Saha 2014 are non-randomised studies so data in the graph is only shown for each sub-group. Data from the different study designs has not been pooled. Please note all three studies defined this outcome as extension of uterine incision, and provided no further details.

	Vaginal push or pull		Patwardhan method			Peto Odds Ratio
**Study or Subgroup**	**Events**	**Total**	**Events**	**Total**	**Weight**	**Peto, Fixed, 95% CI**
**8.1.1 RCTs**						
Bhoi 2019	34	291	0	129	100.0%	4.79 [2.24, 10.24]
**Subtotal (95% CI)**		**291**		**129**	**100.0%**	**4.79 [2.24, 10.24]**
Total events	34		0			
Heterogeneity: Not applicable						
Test for overall effect: Z = 4.04 (P < 0.0001)						
**8.1.2 NRS**						
Bansiwal 2017	17	71	2	64	65.3%	5.52 [2.10, 14.54]
Saha 2014	10	44	0	35	34.7%	7.61 [2.02, 28.68]
**Subtotal (95% CI)**		**115**		**99**	**100.0%**	**6.17 [2.82, 13.49]**
Total events	27		2			
Heterogeneity: $Chi^2 = 0.15$, df = 1 (P = 0.70); $I^2 = 0\%$						
Test for overall effect: Z = 4.56 (P < 0.00001)						

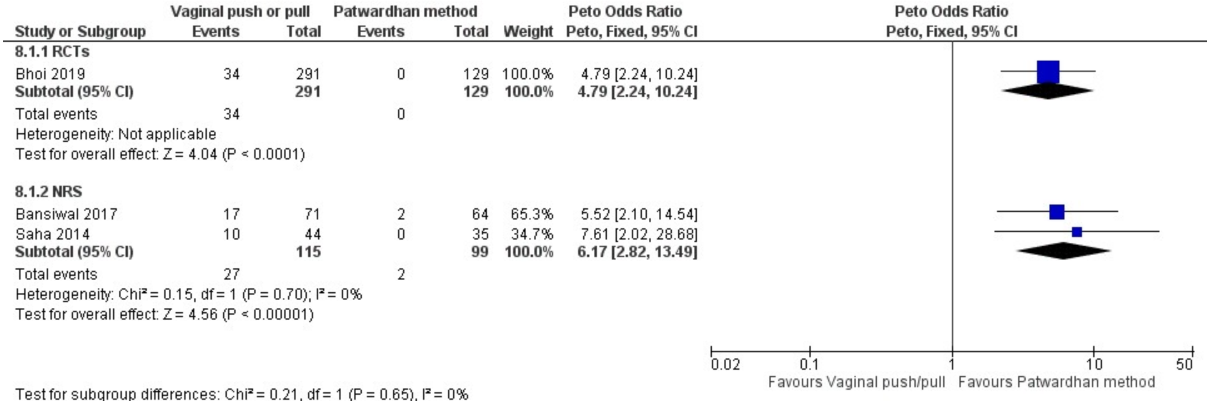

Test for subgroup differences: $Chi^2 = 0.21$, df = 1 (P = 0.65), $I^2 = 0\%$

Sensitivity analysis without Bansiwal 2017 study data

	Vaginal push or pull		Patwardhan method			Peto Odds Ratio
**Study or Subgroup**	**Events**	**Total**	**Events**	**Total**	**Weight**	**Peto, Fixed, 95% CI**
**8.1.1 RCTs**						
Bhoi 2019	34	291	0	129	100.0%	4.79 [2.24, 10.24]
**Subtotal (95% CI)**		**291**		**129**	**100.0%**	**4.79 [2.24, 10.24]**
Total events	34		0			
Heterogeneity: Not applicable						
Test for overall effect: Z = 4.04 (P < 0.0001)						
**8.1.2 NRS**						
Saha 2014	10	44	0	35	100.0%	7.61 [2.02, 28.68]
**Subtotal (95% CI)**		**44**		**35**	**100.0%**	**7.61 [2.02, 28.68]**
Total events	10		0			
Heterogeneity: Not applicable						
Test for overall effect: Z = 3.00 (P = 0.003)						

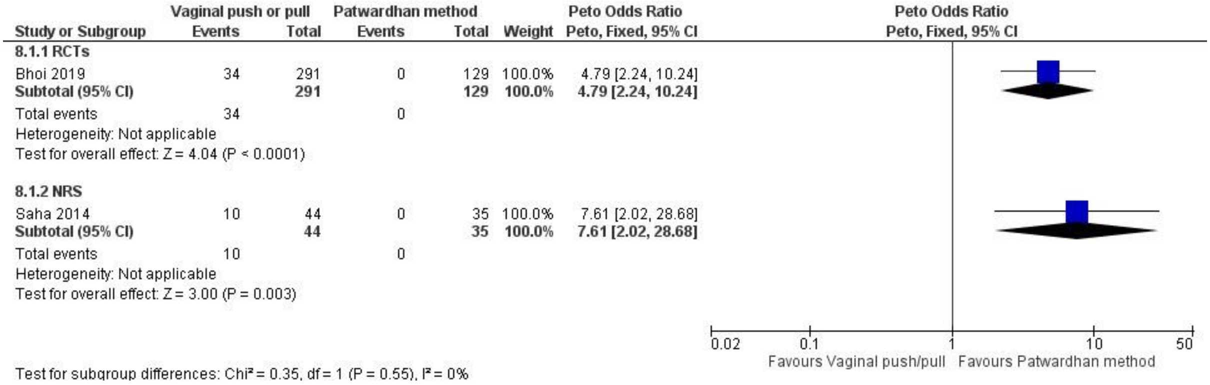

Test for subgroup differences: $Chi^2 = 0.35$, df = 1 (P = 0.55), $I^2 = 0\%$

Maternal blood transfusion for the comparison of vaginal push or pull versus Patwardhan method

Bhoi 2019 is an RCT. Bansiwal 2017 and Saha 2014 are non-randomised studies, so data in the graph is only shown for each sub-group. Data from the different study designs has not been pooled.

	Vaginal push or pull		Patwardhan method			Risk Ratio
Study or Subgroup	Events	Total	Events	Total	Weight	M-H, Random, 95% CI
**8.6.1 RCTs**						
Bhoi 2019	39	291	6	129	100.0%	2.88 [1.25, 6.64]
**Subtotal (95% CI)**		**291**		**129**	**100.0%**	**2.88 [1.25, 6.64]**
Total events	39		6			
Heterogeneity: Not applicable						
Test for overall effect: Z = 2.49 (P = 0.01)						
**8.6.2 NRS**						
Bansiwal 2017	16	71	1	64	36.8%	14.42 [1.97, 105.70]
Saha 2014	12	44	3	35	63.2%	3.18 [0.97, 10.40]
**Subtotal (95% CI)**		**115**		**99**	**100.0%**	**5.55 [1.23, 24.96]**
Total events	28		4			
Heterogeneity: Tau² = 0.57; Chi² = 1.81, df = 1 (P = 0.18); I² = 45%						
Test for overall effect: Z = 2.23 (P = 0.03)						

Test for subgroup differences: Chi² = 0.56, df = 1 (P = 0.45), I² = 0%

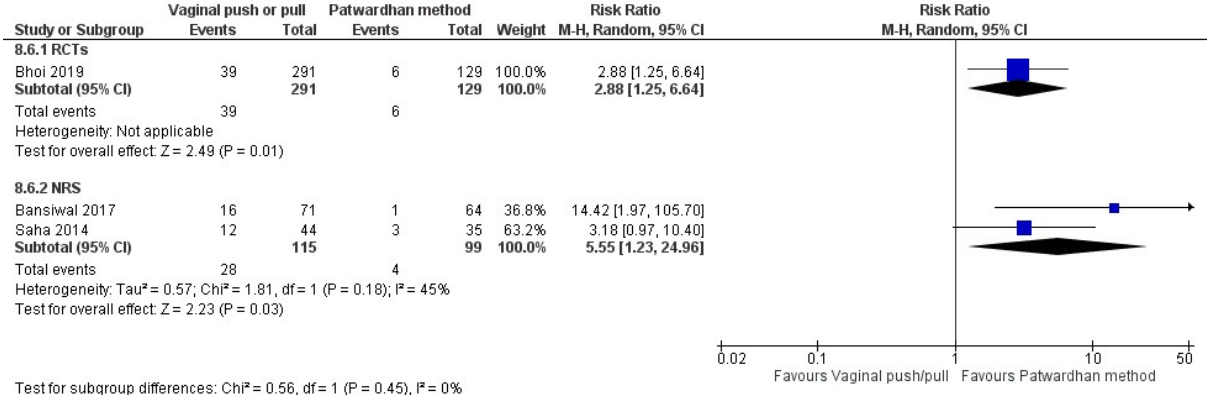

Sensitivity analysis without Bansiwal 2017 study data

	Vaginal push or pull		Patwardhan method			Risk Ratio
Study or Subgroup	Events	Total	Events	Total	Weight	M-H, Random, 95% CI
**8.6.1 RCTs**						
Bhoi 2019	39	291	6	129	100.0%	2.88 [1.25, 6.64]
**Subtotal (95% CI)**		**291**		**129**	**100.0%**	**2.88 [1.25, 6.64]**
Total events	39		6			
Heterogeneity: Not applicable						
Test for overall effect: Z = 2.49 (P = 0.01)						
**8.6.2 NRS**						
Saha 2014	12	44	3	35	100.0%	3.18 [0.97, 10.40]
**Subtotal (95% CI)**		**44**		**35**	**100.0%**	**3.18 [0.97, 10.40]**
Total events	12		3			
Heterogeneity: Not applicable						
Test for overall effect: Z = 1.91 (P = 0.06)						

Test for subgroup differences: Chi² = 0.02, df = 1 (P = 0.89), I² = 0%

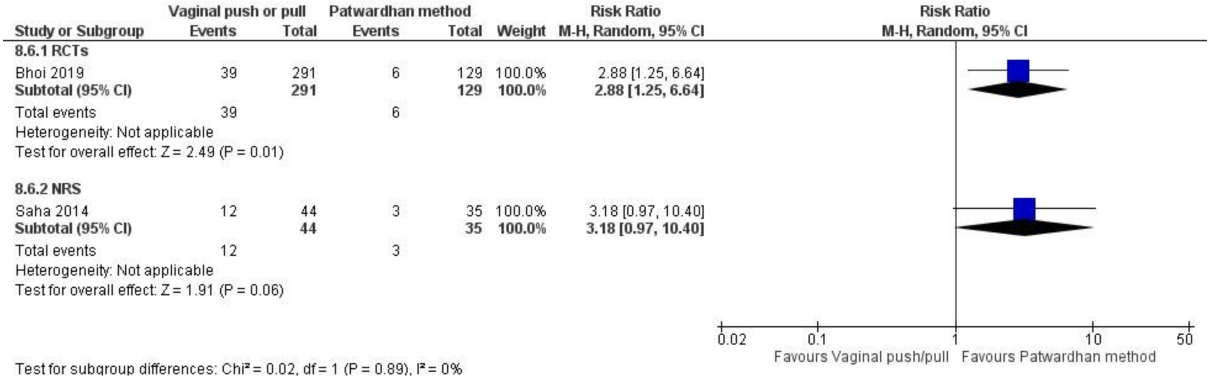

NICU admission for the comparison of vaginal push or pull versus Patwardhan method

Bhoi 2019 is an RCT. Bansiwal 2017 and Saha 2014 are non-randomised studies, so data in the graph is only shown for each sub-group. Data from the different study designs has not been pooled.

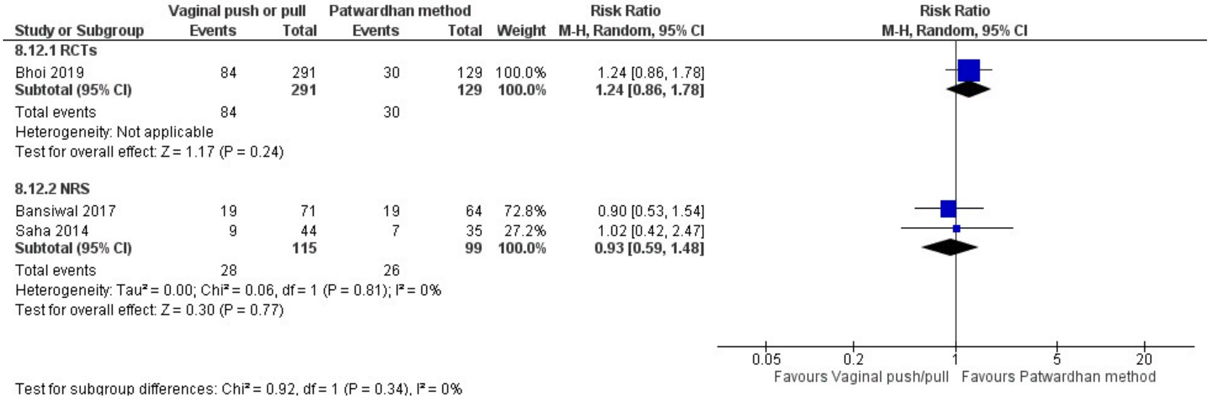

Study or Subgroup	Vaginal push or pull Events	Vaginal push or pull Total	Patwardhan method Events	Patwardhan method Total	Weight	Risk Ratio M-H, Random, 95% CI
**8.12.1 RCTs**						
Bhoi 2019	84	291	30	129	100.0%	1.24 [0.86, 1.78]
**Subtotal (95% CI)**		**291**		**129**	**100.0%**	**1.24 [0.86, 1.78]**
Total events	84		30			
Heterogeneity: Not applicable						
Test for overall effect: Z = 1.17 (P = 0.24)						
**8.12.2 NRS**						
Bansiwal 2017	19	71	19	64	72.8%	0.90 [0.53, 1.54]
Saha 2014	9	44	7	35	27.2%	1.02 [0.42, 2.47]
**Subtotal (95% CI)**		**115**		**99**	**100.0%**	**0.93 [0.59, 1.48]**
Total events	28		26			
Heterogeneity: Tau² = 0.00; Chi² = 0.06, df = 1 (P = 0.81); I² = 0%						
Test for overall effect: Z = 0.30 (P = 0.77)						
Test for subgroup differences: Chi² = 0.92, df = 1 (P = 0.34), I² = 0%						

Sensitivity analysis without Bansiwal 2017 study data

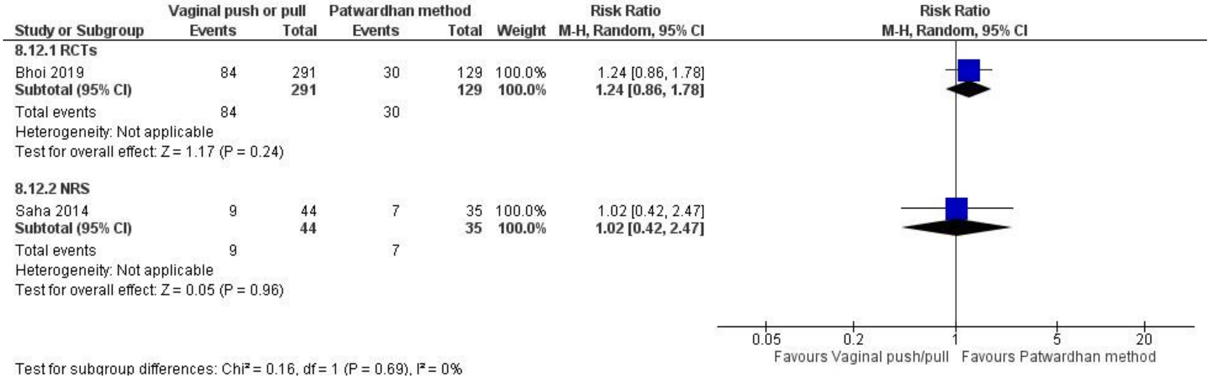

Study or Subgroup	Vaginal push or pull Events	Vaginal push or pull Total	Patwardhan method Events	Patwardhan method Total	Weight	Risk Ratio M-H, Random, 95% CI
**8.12.1 RCTs**						
Bhoi 2019	84	291	30	129	100.0%	1.24 [0.86, 1.78]
**Subtotal (95% CI)**		**291**		**129**	**100.0%**	**1.24 [0.86, 1.78]**
Total events	84		30			
Heterogeneity: Not applicable						
Test for overall effect: Z = 1.17 (P = 0.24)						
**8.12.2 NRS**						
Saha 2014	9	44	7	35	100.0%	1.02 [0.42, 2.47]
**Subtotal (95% CI)**		**44**		**35**	**100.0%**	**1.02 [0.42, 2.47]**
Total events	9		7			
Heterogeneity: Not applicable						
Test for overall effect: Z = 0.05 (P = 0.96)						
Test for subgroup differences: Chi² = 0.16, df = 1 (P = 0.69), I² = 0%						

Fetal pillow® versus no pillow

Uterine incision extensions combined for the comparison of Fetal pillow® versus no pillow.

All studies are non-randomised.

Study or Subgroup	Fetal pillow Events	Fetal pillow Total	No fetal pillow Events	No fetal pillow Total	Weight	Risk Ratio M-H, Random, 95% CI
Chooi 2022	11	53	12	48	23.2%	0.83 [0.40, 1.70]
Hanley 2020	13	114	11	60	22.3%	0.62 [0.30, 1.30]
Sacre 2021	37	170	47	221	46.9%	1.02 [0.70, 1.50]
Seal 2014	2	50	19	124	7.6%	0.26 [0.06, 1.08]
**Total (95% CI)**		**387**		**453**	**100.0%**	**0.79 [0.52, 1.19]**
Total events	63		89			

Heterogeneity: Tau² = 0.06; Chi² = 4.36, df = 3 (P = 0.22); I² = 31%
Test for overall effect: Z = 1.14 (P = 0.25)

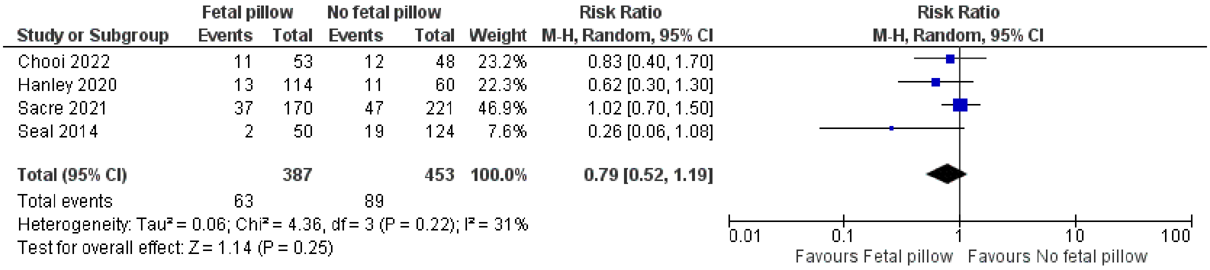

Sensitivity analysis without Seal 2014 data

Study or Subgroup	Fetal pillow Events	Fetal pillow Total	No fetal pillow Events	No fetal pillow Total	Weight	Risk Ratio M-H, Random, 95% CI
Chooi 2022	11	53	12	48	18.2%	0.83 [0.40, 1.70]
Hanley 2020	13	114	11	60	17.2%	0.62 [0.30, 1.30]
Sacre 2021	37	170	47	221	64.6%	1.02 [0.70, 1.50]
**Total (95% CI)**		**337**		**329**	**100.0%**	**0.90 [0.67, 1.23]**
Total events	61		70			

Heterogeneity: Tau² = 0.00; Chi² = 1.44, df = 2 (P = 0.49); I² = 0%
Test for overall effect: Z = 0.64 (P = 0.52)

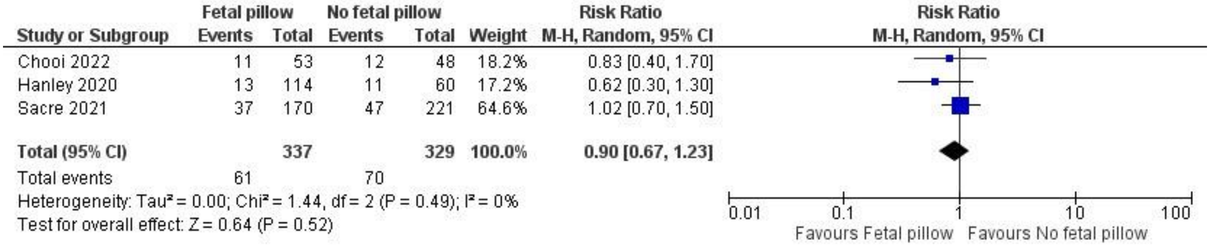

Uterine incision extension on lower segment for the comparison of Fetal pillow® versus no pillow.

All studies are non-randomised. Please note in Sacre 2021 uterine incision extension was defined by the clinician (no other details provided).

	Fetal pillow		No fetal pillow			Risk Ratio
**Study or Subgroup**	**Events**	**Total**	**Events**	**Total**	**Weight**	**M-H, Random, 95% CI**
Hanley 2020	13	114	11	60	28.9%	0.62 [0.30, 1.30]
Sacre 2021	37	170	47	221	71.1%	1.02 [0.70, 1.50]
**Total (95% CI)**		**284**		**281**	**100.0%**	**0.89 [0.57, 1.38]**
Total events	50		58			

Heterogeneity: Tau² = 0.03; Chi² = 1.38, df = 1 (P = 0.24); I² = 27%
Test for overall effect: Z = 0.54 (P = 0.59)

Risk Ratio
M-H, Random, 95% CI

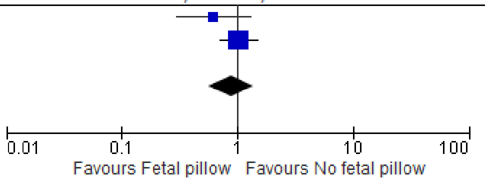

Maternal operative blood loss (ml) for the comparison of Fetal pillow® versus no pillow.

All studies are non-randomised.

	Fetal pillow			No fetal pillow				Mean Difference
**Study or Subgroup**	**Mean**	**SD**	**Total**	**Mean**	**SD**	**Total**	**Weight**	**IV, Random, 95% CI**
Chooi 2022	699.06	393.88	53	797.92	488.26	48	32.4%	-98.86 [-273.00, 75.28]
Hanley 2020	730	400	113	725	374	59	67.6%	5.00 [-115.61, 125.61]
**Total (95% CI)**			**166**			**107**	**100.0%**	**-28.67 [-127.82, 70.48]**

Heterogeneity: Tau² = 0.00; Chi² = 0.92, df = 1 (P = 0.34); I² = 0%

Test for overall effect: Z = 0.57 (P = 0.57)

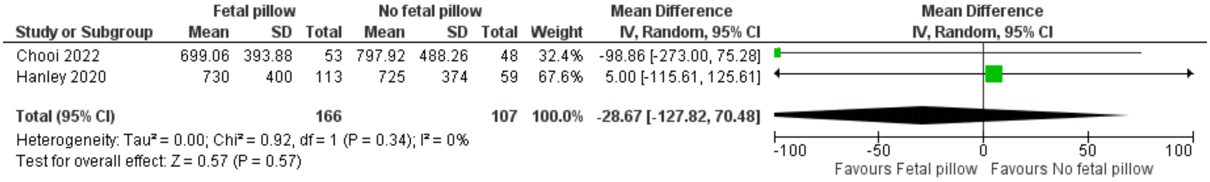

Maternal operative blood loss >1000 ml (postpartum haemorrhage) for the comparison of Fetal pillow® versus no pillow.

All studies are non-randomised. In Sacre 2021 in the Fetal pillow group 15/170 had operative blood loss >1500 ml, and in the no-pillow group 14/221 had operative blood loss >1500 ml.

	Fetal pillow		No fetal pillow			Risk Ratio
Study or Subgroup	Events	Total	Events	Total	Weight	M-H, Random, 95% CI
**1.3.2 NRS**						
Chooi 2022	14	53	14	48	23.5%	0.91 [0.48, 1.70]
Hanley 2020	15	114	7	60	13.2%	1.13 [0.49, 2.61]
Sacre 2021	39	170	41	221	61.1%	1.24 [0.84, 1.83]
Seal 2014	1	50	10	124	2.3%	0.25 [0.03, 1.89]
**Subtotal (95% CI)**		**387**		**453**	**100.0%**	**1.10 [0.81, 1.49]**
Total events	69		72			

Heterogeneity: Tau² = 0.00; Chi² = 2.83, df = 3 (P = 0.42); I² = 0%
Test for overall effect: Z = 0.58 (P = 0.56)

Test for subgroup differences: Not applicable

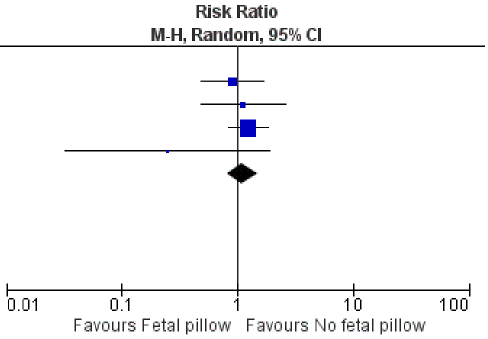

Sensitivity analysis without Seal 2014 data

	Fetal pillow		No fetal pillow			Risk Ratio
Study or Subgroup	Events	Total	Events	Total	Weight	M-H, Random, 95% CI
**1.3.2 NRS**						
Chooi 2022	14	53	14	48	24.0%	0.91 [0.48, 1.70]
Hanley 2020	15	114	7	60	13.5%	1.13 [0.49, 2.61]
Sacre 2021	39	170	41	221	62.5%	1.24 [0.84, 1.83]
**Subtotal (95% CI)**		**337**		**329**	**100.0%**	**1.13 [0.83, 1.54]**
Total events	68		62			

Heterogeneity: Tau² = 0.00; Chi² = 0.68, df = 2 (P = 0.71); I² = 0%
Test for overall effect: Z = 0.80 (P = 0.43)

Test for subgroup differences: Not applicable

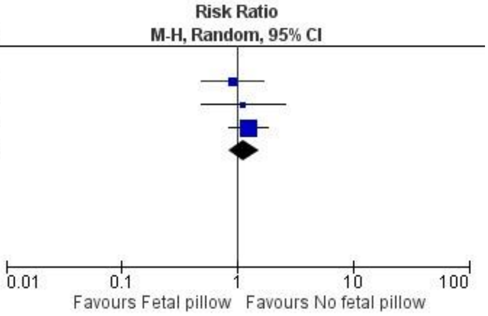

Infant birth trauma for the comparison of Fetal pillow® versus no pillow.

All studies are non-randomised. As there were zero events in one of the comparison groups for Seal 2014, precluding the calculation of RR, we calculated Peto odds ratios (POR).

	Fetal pillow		No fetal pillow			Peto Odds Ratio
**Study or Subgroup**	**Events**	**Total**	**Events**	**Total**	**Weight**	**Peto, Fixed, 95% CI**
Chooi 2022	16	53	11	48	80.7%	1.44 [0.60, 3.48]
Seal 2014 (1)	0	50	6	124	19.3%	0.24 [0.04, 1.42]
**Total (95% CI)**		**103**		**172**	**100.0%**	**1.02 [0.46, 2.24]**
Total events	16		17			
Heterogeneity: Chi² = 3.16, df = 1 (P = 0.08); I² = 68%						
Test for overall effect: Z = 0.04 (P = 0.97)						

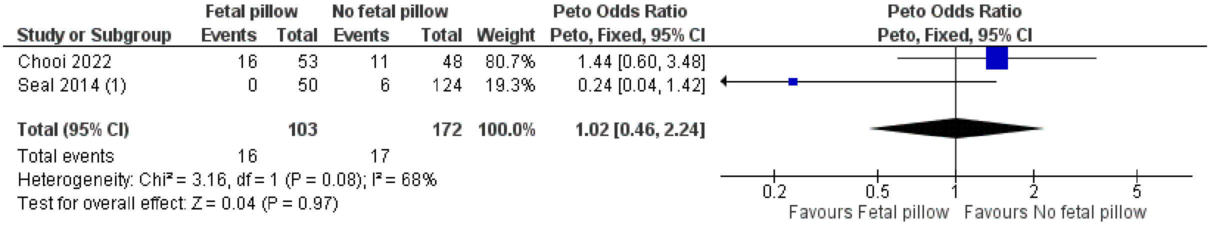

Footnotes
(1) Defined as neonatal injury.

Apgar score <7 at five minutes for the comparison of Fetal pillow® versus no pillow

All studies are non-randomised.

	Fetal pillow		No fetal pillow			Risk Ratio
Study or Subgroup	Events	Total	Events	Total	Weight	M-H, Random, 95% CI
Chooi 2022	2	53	3	48	0.2%	0.60 [0.11, 3.46]
Hanley 2020	105	113	56	60	98.6%	1.00 [0.91, 1.08]
Sacre 2021	12	170	12	221	1.2%	1.30 [0.60, 2.82]
**Total (95% CI)**		**336**		**329**	**100.0%**	**1.00 [0.92, 1.09]**
Total events	119		71			

Heterogeneity: Tau² = 0.00; Chi² = 0.99, df = 2 (P = 0.61); I² = 0%

Test for overall effect: Z = 0.06 (P = 0.95)

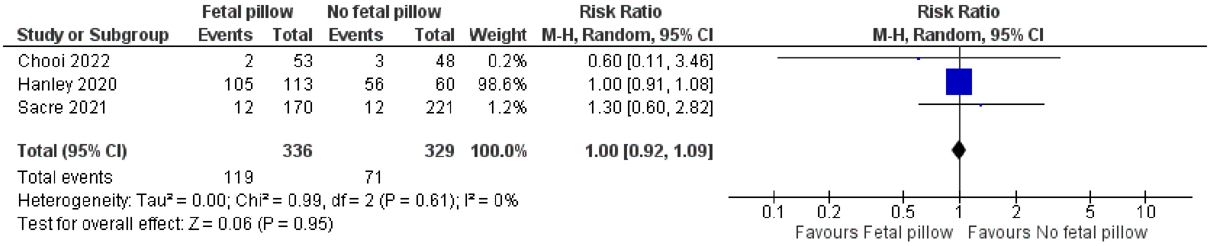

Maternal blood transfusion for the comparison of Fetal pillow® versus no pillow.

All studies are non-randomised.

Study or Subgroup	Fetal pillow Events	Fetal pillow Total	No fetal pillow Events	No fetal pillow Total	Weight	Risk Ratio M-H, Random, 95% CI
**1.9.2 NRS**						
Chooi 2022	2	53	1	48	11.8%	1.81 [0.17, 19.35]
Hanley 2020	1	114	3	58	13.2%	0.17 [0.02, 1.59]
Sacre 2021	8	170	9	221	60.0%	1.16 [0.46, 2.93]
Seal 2014	1	50	6	124	15.0%	0.41 [0.05, 3.35]
**Subtotal (95% CI)**		**387**		**451**	**100.0%**	**0.81 [0.35, 1.87]**
Total events	12		19			
Heterogeneity: Tau² = 0.08; Chi² = 3.28, df = 3 (P = 0.35); I² = 9%						
Test for overall effect: Z = 0.49 (P = 0.62)						
**Total (95% CI)**		**387**		**451**	**100.0%**	**0.81 [0.35, 1.87]**
Total events	12		19			
Heterogeneity: Tau² = 0.08; Chi² = 3.28, df = 3 (P = 0.35); I² = 9%						
Test for overall effect: Z = 0.49 (P = 0.62)						
Test for subgroup differences: Not applicable						

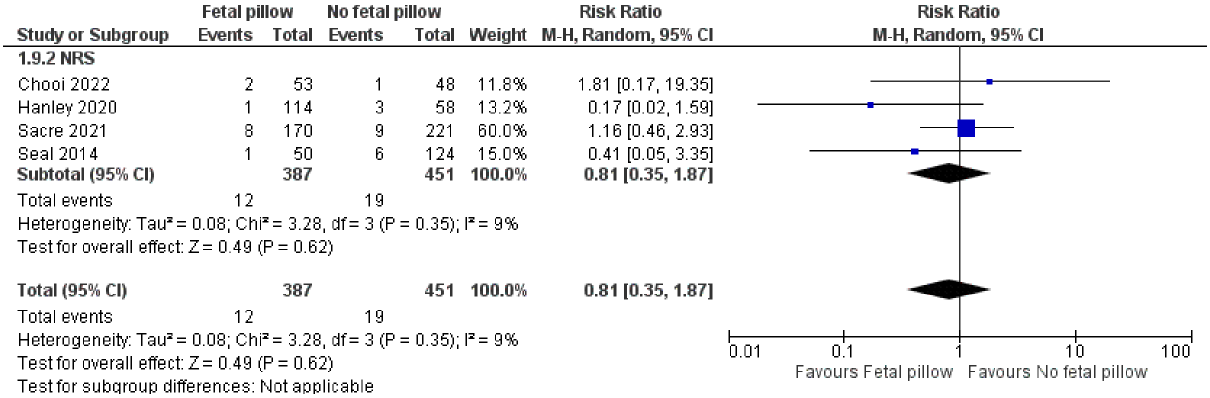

Sensitivity analysis without Seal 2014 data

Study or Subgroup	Fetal pillow Events	Fetal pillow Total	No fetal pillow Events	No fetal pillow Total	Weight	Risk Ratio M-H, Random, 95% CI
**1.9.2 NRS**						
Chooi 2022	2	53	1	48	18.6%	1.81 [0.17, 19.35]
Hanley 2020	1	114	3	58	20.3%	0.17 [0.02, 1.59]
Sacre 2021	8	170	9	221	61.1%	1.16 [0.46, 2.93]
**Subtotal (95% CI)**		**337**		**327**	**100.0%**	**0.85 [0.28, 2.62]**
Total events	11		13			
Heterogeneity: Tau² = 0.31; Chi² = 2.74, df = 2 (P = 0.25); I² = 27%						
Test for overall effect: Z = 0.28 (P = 0.78)						
**Total (95% CI)**		**337**		**327**	**100.0%**	**0.85 [0.28, 2.62]**
Total events	11		13			
Heterogeneity: Tau² = 0.31; Chi² = 2.74, df = 2 (P = 0.25); I² = 27%						
Test for overall effect: Z = 0.28 (P = 0.78)						
Test for subgroup differences: Not applicable						

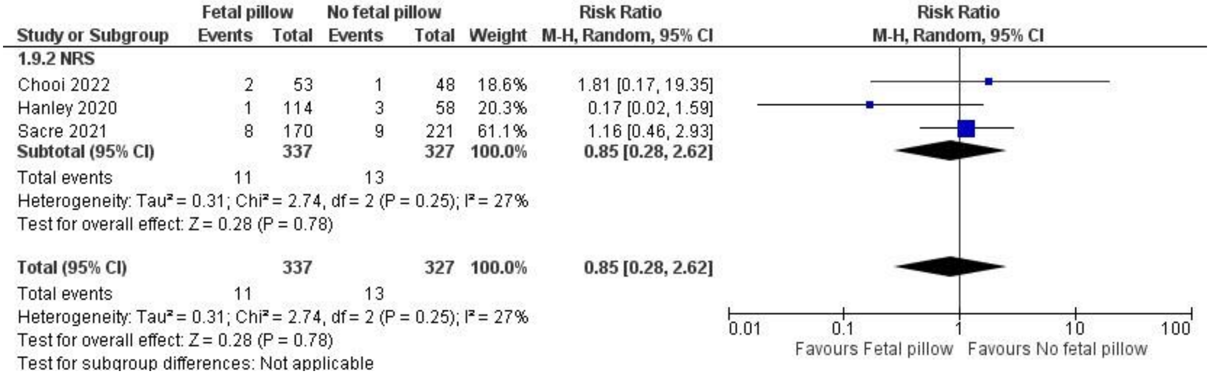

Uterine incision extension into cervix or vagina for the comparison of Fetal pillow® versus no pillow.

All studies are non-randomised. The overall pooled estimates have not been included due to very high levels of heterogeneity (I^2= 95%). Hanley 2020 reported this outcome as vaginal trauma. In Seal 2014 this outcome was defined as uterine extension Grades II (extension that increases the operating time and blood loss) and III (extension that involves one or both uterine arteries, cervix, vagina or other organs).

	Fetal pillow		No fetal pillow		Risk Difference
Study or Subgroup	Events	Total	Events	Total	M-H, Random, 95% CI
**1.11.2 NRS**					
Hanley 2020	0	114	0	60	0.00 [-0.03, 0.03]
Seal 2014	2	50	19	124	-0.11 [-0.20, -0.03]

Risk Difference
M-H, Random, 95% CI

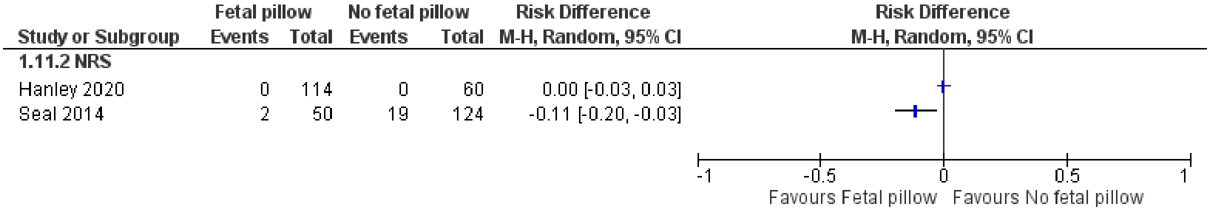

Sensitivity analysis without Seal 2014 data

Only one study (Hanley 2020) remaining so pooling not possible. Plot shown for illustrative purposes only.

	Fetal pillow		No fetal pillow		Risk Difference
Study or Subgroup	Events	Total	Events	Total	M-H, Random, 95% CI
**1.11.2 NRS**					
Hanley 2020	0	114	0	60	0.00 [-0.03, 0.03]

Risk Difference
M-H, Random, 95% CI

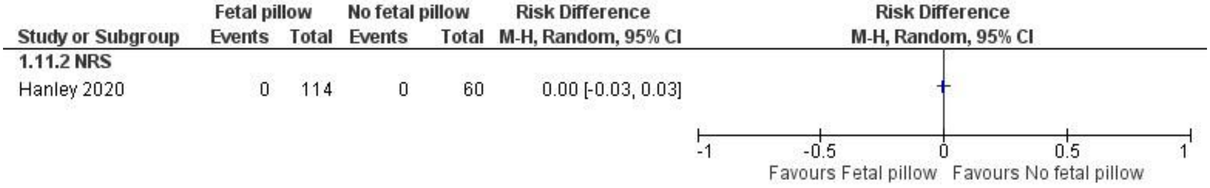

Maternal duration of hospital stay (days) for the comparison of Fetal pillow® versus no pillow.

All studies are non-randomised. Please note in Seal 2014 standard deviations were not reported.

Study or Subgroup	Fetal pillow Mean	Fetal pillow SD	Fetal pillow Total	No fetal pillow Mean	No fetal pillow SD	No fetal pillow Total	Weight	Mean Difference IV, Random, 95% CI
**1.14.2 NRS**								
Chooi 2022	3.22	1.41	53	3.45	1.23	48	32.0%	-0.23 [-0.74, 0.28]
Hanley 2020	2.74	1.01	114	3.02	1.18	59	68.0%	-0.28 [-0.63, 0.07]
Seal 2014	4.1	0	50	6.4	0	124		Not estimable
**Subtotal (95% CI)**			**217**			**231**	**100.0%**	**-0.26 [-0.56, 0.03]**

Heterogeneity: Tau² = 0.00; Chi² = 0.02, df = 1 (P = 0.88); I² = 0%

Test for overall effect: Z = 1.77 (P = 0.08)

Test for subgroup differences: Not applicable

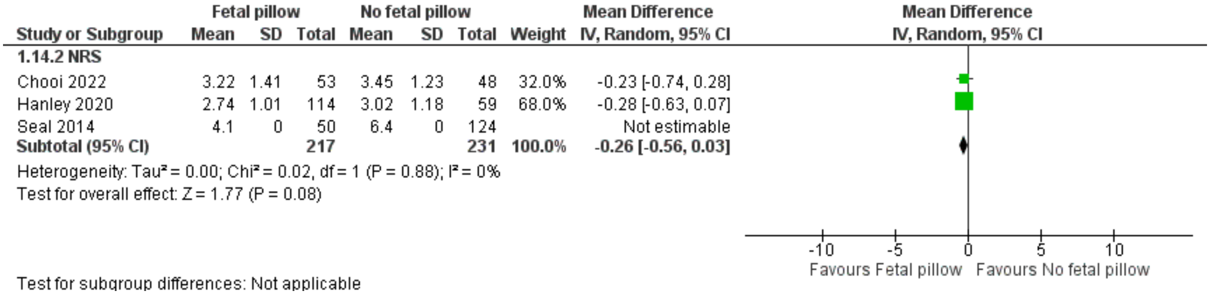

No sensitivity analysis conducted without Seal 2014 as while this study reports mean data, no SD was reported, so Seal 2014 data is not included in the above pooled analysis.

NICU admission for the comparison of Fetal pillow® versus no pillow

All studies are non-randomised. Please note: Chooi 2022 has not been included in the analysis – only a combined ‘admission to nursery’ outcome was reported which included both the special baby care unit and the neonatal intensive care unit and a separate ‘admission to high dependency unit’ outcome, so the NICU unit admission data is not separate from the other outcomes.

	Fetal pillow		No fetal pillow			Risk Ratio
**Study or Subgroup**	**Events**	**Total**	**Events**	**Total**	**Weight**	**M-H, Random, 95% CI**
**1.17.2 NRS**						
Hanley 2020	37	112	24	60	63.6%	0.83 [0.55, 1.24]
Sacre 2021	15	170	27	221	29.4%	0.72 [0.40, 1.31]
Seal 2014	3	50	12	124	7.1%	0.62 [0.18, 2.10]
**Subtotal (95% CI)**		**332**		**405**	**100.0%**	**0.78 [0.56, 1.08]**
Total events	55		63			
Heterogeneity: Tau² = 0.00; Chi² = 0.29, df = 2 (P = 0.87); I² = 0%						
Test for overall effect: Z = 1.52 (P = 0.13)						
**Total (95% CI)**		**332**		**405**	**100.0%**	**0.78 [0.56, 1.08]**
Total events	55		63			
Heterogeneity: Tau² = 0.00; Chi² = 0.29, df = 2 (P = 0.87); I² = 0%						
Test for overall effect: Z = 1.52 (P = 0.13)						
Test for subgroup differences: Not applicable						

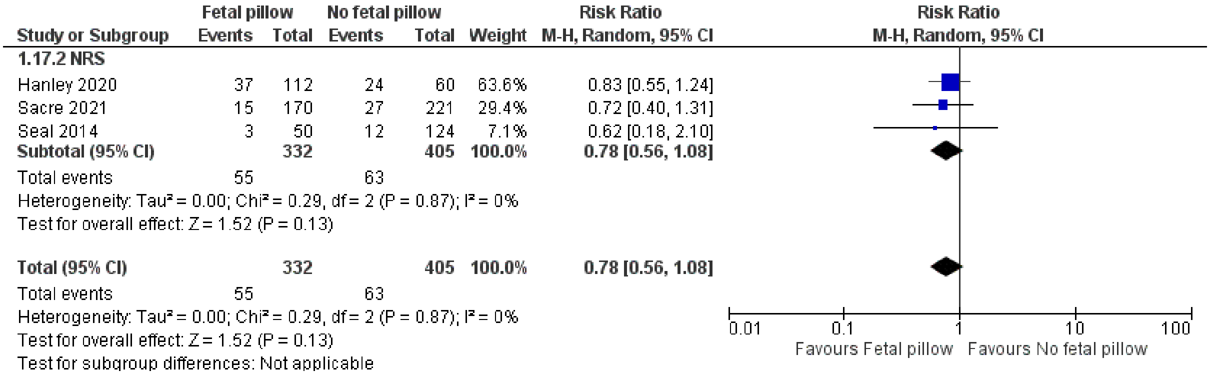

Sensitivity analysis without Seal 2014 data

	Fetal pillow		No fetal pillow			Risk Ratio
**Study or Subgroup**	**Events**	**Total**	**Events**	**Total**	**Weight**	**M-H, Random, 95% CI**
**1.17.2 NRS**						
Hanley 2020	37	112	24	60	68.4%	0.83 [0.55, 1.24]
Sacre 2021	15	170	27	221	31.6%	0.72 [0.40, 1.31]
**Subtotal (95% CI)**		**282**		**281**	**100.0%**	**0.79 [0.57, 1.11]**
Total events	52		51			
Heterogeneity: Tau² = 0.00; Chi² = 0.14, df = 1 (P = 0.71); I² = 0%						
Test for overall effect: Z = 1.36 (P = 0.17)						
**Total (95% CI)**		**282**		**281**	**100.0%**	**0.79 [0.57, 1.11]**
Total events	52		51			
Heterogeneity: Tau² = 0.00; Chi² = 0.14, df = 1 (P = 0.71); I² = 0%						
Test for overall effect: Z = 1.36 (P = 0.17)						
Test for subgroup differences: Not applicable						

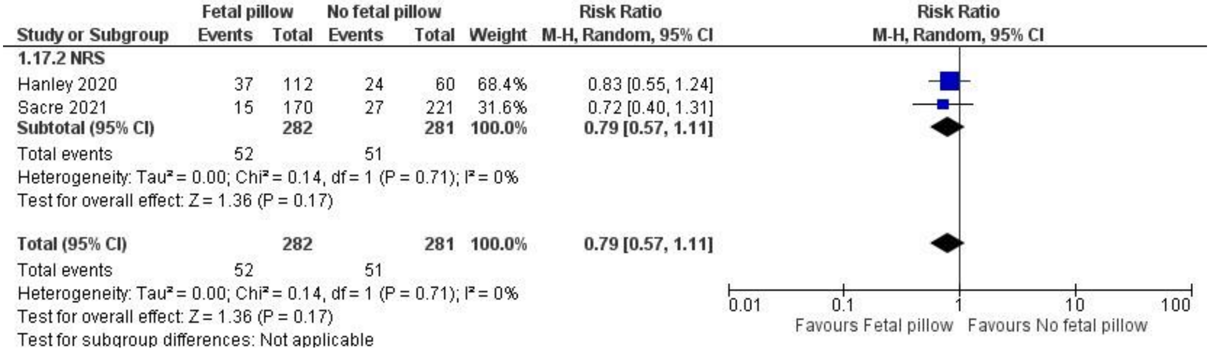

Umbilical artery pH for the comparison of Fetal pillow® versus no pillow

All studies are non-randomised. Please note due to high heterogeneity (I^2=81%) pooled totals are not shown.

	Fetal pillow			No fetal pillow				Mean Difference
Study or Subgroup	Mean	SD	Total	Mean	SD	Total	Weight	IV, Random, 95% CI
Chooi 2022	7.25	0.06	53	7.24	0.08	48		0.01 [-0.02, 0.04]
Hanley 2020 (1)	7.25	0.08	98	7.19	0.1	49		0.06 [0.03, 0.09]

Mean Difference
IV, Random, 95% CI

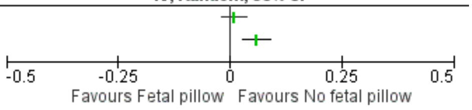

Footnotes
(1) Normal pH: 7.18 to 7.38.
