# Supplementary material for: Management of impacted fetal head at cesarean birth: A systematic review and meta‐analysis
Source: Acta Obstet Gynecol Scand. 2024 May 24;103(9):1702–13. doi: 10.1111/aogs.14873 (PMC11324922; doi:10.1111/aogs.14873)
Supplement: Supplementary file 4 — Table S1. [file AOGS-103-1702-s007.pdf]

# Table S1: Excluded studies and reasons for exclusion

| Study | Reason for exclusion |
|---|---|
| Ashraf M S I. Reverse Breech Extraction Versus Dis-Impaction of the Head During Cesarean Section for Obstructed Labor. J Gynecol Women's Health. 2020: 19(2): 556006.a | The study states it is an RCT using a 1:1 randomisation, but describes that "the decision for the performance of the reverse breech extraction or to go on with push approach was taken in the operating theatre by the operating surgeon". This suggests that the comparison of interest - the push approach versus reverse breech extraction - was not randomised. Hence the study is assumed to be a non-randomised study with N=20 in both arms, which would not meet the minimum N≥30 per treatment arm criterion |
| Bastani P., Pourabolghase S., Abbasalizadeh, F., Motvalli, L. Erratum: Comparison of neonatal and maternal outcomes associated with head-pushing and head-pulling methods for impacted fetal head extraction during cesarean delivery (International Journal of Gynecology and Obstetrics (2012) 118 (1-3)). | Erratum referring to a correction in the author names |
| Bellad M.B. Safety and effectiveness of ventouse extraction versus manual extraction of fetal head at cesarean section - a randomized controlled trial. Clinical Trials Registry - India | Ineligible intervention: Ventouse extraction |
| Berhan Y., Berhan A. A meta-analysis of reverse breech extraction to deliver a deeply impacted head during cesarean delivery. International Journal of Gynecology & Obstetrics 2014;124:99–105 | Systematic review, included studies checked for relevance |
| Bloch C., Dore S., Hobson S. Committee Opinion No. 415: Impacted Fetal Head, Second-Stage Cesarean Delivery. J Obstetrics Gynaecol Can 2021;43:406–13 | Canadian guideline, included studies checked for relevance |
| Bofill J.A., Lencki S.G., Barhan S., Ezenagu L.C. Instrumental delivery of the fetal head at the time of elective repeat cesarean: a randomized pilot study. American Journal of Perinatology 2000;17(5):265-9 | Ineligible population: Women who are having an elective repeat caesarean section |
| Buckley V.A., Wu J., De Vries B. Outcomes following acute tocolysis prior to emergency caesarean section. Australian and New Zealand Journal of Obstetrics and Gynaecology. 2020;60(6):884-9 | Ineligible population: More than half of the women experienced fetal compromise; none of the women were reported to have experienced impacted fetal head |
| Buhimischi C.S., Buhimischi I.A., Malinow A.M., Weiner C.P. Effects of sublingual nitroglycerin on human uterine contractility during the active phase of labor (abstract). American Journal of Obstetrics and Gynecology 2001;185:S209 | Abstract |

| Study | Reason for exclusion |
|---|---|
| Buhimschi C.S., Buhimschi I.A., Malinow A.M., Wiener C.P. Effects of sublingual nitroglycerin on human uterine contractility during the active phase of labor. American Journal of Obstetrics and Gynecology 2002;187:235-8 | Ineligible population: Women undergoing vaginal delivery, not emergency caesarean section |
| Chopra S., Bagga R., Keepanasseril A., Jain V., Kalra J., Suri V. Disengagement of the deeply engaged fetal head during cesarean section in advanced labor: conventional method versus reverse breech extraction. Acta Obstetricia et Gynecologica Scandinavica 2009;88:1163–6 | Insufficient details about methods employed in cephalic extraction group (i.e., unclear what proportion of women had a vaginal push up method) |
| Colletta A., Lassey S., Pomerleau M., Robinson J.N. Double-Blind Randomized Controlled Trial of a Cephalic Elevation Device for Second-Stage Cesarean Birth...Association of Women's Health, Obstetric and Neonatal Nurses, Virtual Convention, November 1-4, 2020. JOGNN: journal of obstetric, gynecologic & neonatal nursing. 2020;49(6):S22 | Conference abstract of included study (Lassey 2020) |
| Cook Jr V.D., Spinnato Ii J.A. Terbutaline tocolysis prior to cesarean section for fetal distress. Journal of Maternal-Fetal Medicine. 1994;3(5):219-26 | Ineligible population: Women at risk of tachycardia; none of the women were reported to have experienced impacted fetal head |
| de Heus, R., et al. A prospective randomized trial of acute tocolysis in term labour with atosiban or ritodrine. European journal of obstetrics, gynecology, and reproductive biology. 2008;139(2): 139-145 | Mixed population: Outcome data not reported separately for women undergoing emergency caesarean section, waiting for caesarean section or continuing with labour (N=44% emergency caesarean section); unclear whether women experienced impacted fetal head |
| Dewick L., Raouf S., Uzoma A. Morbidity from caesarean section at full dilatation. BJOG: An International Journal of Obstetrics and Gynaecology. 2018;125(Supplement 2):31 | Conference abstract |
| Di Girolamo, R., et al. "Outcomes of second stage cesarean section following the use of a fetal head elevation device: A systematic review and meta-analysis." European Journal of Obstetrics and Gynecology and Reproductive Biology. 2021;262: 1-6 | Systematic review, included studies checked for relevance |
| Dodd J.M., Reid K. Tocolysis for assisting delivery at caesarean section. Cochrane Database of Systematic Reviews. 2006(4):CD004944 | Systematic review, included studies checked for relevance |
| Domini, E., et al. "Delivery of an impacted head during caesarean section. An easy and reliable manoeuvre: To assess adequate and safe hysterotomy and to control bleeding." Giornale Italiano di Ostetricia e Ginecologia. 2006;28(5): 207-208 | Descriptive narrative of author’s experience of ‘push method’ delivery (non-comparative study) |
| Draycott, T., et al. "Impacted fetal head: A retrospective cohort study of emergency caesarean section." European Journal of Obstetrics and Gynecology and Reproductive Biology. 2021;261: 85-91 | Ineligible study intervention and outcomes: Study compares risk factors associated with the presence or absence of impacted fetal head |

| Study | Reason for exclusion |
|---|---|
| Ekechi, C. I. "Rotational forceps. Is it safe? A re-evaluation of the role of rotational forceps: Retrospective comparison of maternal and perinatal outcomes following different methods of birth for malposition in the second stage of labour." BJOG: An International Journal of Obstetrics and Gynaecology. 2014;121(5): 644 | Comment and author's reply |
| Ezra, O., et al. "Cephalic extraction versus breech extraction in second-stage caesarean section: a retrospective study." BJOG: An International Journal of Obstetrics and Gynaecology. 2020;127(12): 1568-1574 | Insufficient details about methods employed in cephalic extraction group, unclear how many women had impacted fetal head |
| Fong, Y. F. and S. Arulkumaran. "Breech extraction - An alternative method of delivering a deeply engaged head at cesarean section." International Journal of Gynecology and Obstetrics. 1997;56(2): 183-184 | Brief communication relating to case studies (N=3) |
| Fothergill R.J., Robertson A., Bond R.A. Neonatal acidaemia related to procrastination at caesarean section. Journal of Obstetrics and Gynaecology of the British Commonwealth 1978;78:1010-23 | Ineligible population: Not women undergoing emergency caesarean section for impacted fetal head; assessment of anaesthetic duration |
| Gil M., et al. Preferred way of delivery of the impacted fetal head in cesarean sections during second stage of labor. Journal of Obstetrics and Gynaecology Research. 2019;45(12): 2386-2393 | Mixed population: "In 55 (27.5%) cases within the 'head first' group, there was need for pushing of the fetal head vaginally by the surgeon's assistant compared to 8 (6.6%) cases in the 'legs first' group (P < 0.001)." (p.2390). Results not reported separately for the 55 women who received vaginal push-up |
| Gopal, G., et al. "Fetal pillow in impacted fetal head: Number needed to show reduction in morbidity." European Journal of Obstetrics Gynecology and Reproductive Biology. 2019;234: e4-e5 | Conference abstract; 2-arm, non-randomised comparative cohort study (prospective intervention arm versus historical controls) including N<30 in one treatment arm |
| Gopal, G., et al. "Does fetal pillow reduce maternal and neonatal morbidity in full dilatation caesarean sections in East of Scotland women: A comparative case control study." BJOG: An International Journal of Obstetrics and Gynaecology. 2018;125(Supplement 2): 45 | Conference abstract; 2-arm, non-randomised comparative cohort study (prospective intervention arm versus historical controls) including N<30 in one treatment arm |
| Hare, A. "A new indication for breech extraction." The Australian & New Zealand journal of obstetrics & gynaecology. 2000;40(4): 477-478 | Letter to the Editor describing case study (N=1) |
| Hassanien, T. "A new indication for breech extraction." Australian and New Zealand Journal of Obstetrics and Gynaecology. 1999;39(4): 509-510 | Editorial comment |
| Hong Y.J., Lin C.F., Chen J.C., Pan P., Wong K.L., Wei T.T. Nifedipine in preeclampsia for cesarean section. Acta Anesthesiologica Sinica 1993;31(1):43-8 | Ineligible population: Women with preeclampsia, not impacted fetal head |
| Iffy, L. and J. J. Apuzzio. "Reverse breech extraction for cesarean section." European | Letter to the Editor; no original data |

| Study | Reason for exclusion |
|---|---|
| Journal of Obstetrics and Gynecology and Reproductive Biology.2006;126(1): 126 | |
| Iffy, L., et al. "Abdominal rescue after entrapment of the aftercoming head." American Journal of Obstetrics and Gynecology. 1986;154(3): 623-624 | Case report (N=1 woman) involving twin vaginal and caesarean delivery; comment |
| Jeve, Y. B., et al. "Authors' reply re: Comparison of techniques used to deliver a deeply impacted fetal head at full dilation: a systematic review and meta-analysis." BJOG : an international journal of obstetrics and gynaecology.2016;123(13): 2226-2227 | Comment and author’s reply |
| Jeve Y.B., Navti O.B., Konje J.C. Comparison of techniques used to deliver a deeply impacted fetal head at full dilation: a systematic review and meta-analysis. BJOG: An International Journal of Obstetrics & Gynaecology 2015;123:337–45 | Systematic review, included studies checked for relevance |
| Jordan A, Herbert N, Rundle-Thiele D, Holland S & Wong A (2022) Foetal Pillow associated uterine and bladder rupture, Journal of Obstetrics and Gynaecology, 42:5, 1551-1552. | Case study report of one woman only |
| Kadhum T. Head pushing versus reverse breech extraction for delivery of impacted head during cesarean section. Kufa Medical Journal 2009;12(1):200-5 | 2-arm, non-randomised study with N<30 per treatment arm |
| Kalburgi, S., et al. "Impact of the fetal pillow device on maternal morbidity." BJOG: An International Journal of Obstetrics and Gynaecology. 2018;125(Supplement 3): 61 | Conference abstract; 2-arm, non-randomised comparative cohort study including N<30 in one treatment arm |
| Karasahin K.E., Ercan M., Alanbay I., Baser I. Comment on 'Disengagement of the deeply engaged fetal head during cesarean section in advanced labor: Conventional method versus reverse breech extraction'. Acta Obstetricia et Gynecologica Scandinavica. 2010;89(6):849-50 | Letter to the Editor; no original data |
| Kaukinen S., Kaukinen L. The harmful effects of beta-2 sympathomimetic drugs as uterine relaxants on caesarean section. Anaesthetist 1978;27(8):388-91 | Article in German |
| Khosla A.H., Dahiya K., Sangwan K. Cesarean section in a wedged head. Indian journal of medical sciences. 2003;57(5):187-91 | Mixed population: Unclear how many women in the “Non-Patwardhan method (delivered as head)” group received vaginal push-up |
| Krispin E., Fischer O., Kneller M., Arbib N., Salman L., Wiznitzer A., et al. Fetal extraction maneuvers during cesarean delivery in the second stage of labor. Journal of Maternal-Fetal and Neonatal Medicine. 2020 (and print version 2022) | 3-arm, non-randomised comparative study with N<30 in one of the two target intervention groups 3 arms (Standard vertex extraction (N=206; not in PICO) versus vaginal push up (N=116) versus reverse breech (N=28) |
| Kujabi, M. L., et al. Impacted foetal head at emergency caesarean sections. Ugeskrift for laeger. 2021;183(32) | Narrative review, references checked for relevance |
| Kulier R., Gulmezoglu A.M., Hofmeyr G.J., Van Gelderen C.J. Betamimetics in fetal distress: | Ineligible population: Women who had developed fetal heart rate abnormalities consistent with fetal distress; none of the |

| Study | Reason for exclusion |
|---|---|
| randomised controlled trial. Journal of Perinatal Medicine 1997;25(1):97-100 | women were reported to have experienced impacted fetal head |
| Levy R., Chernomoretz T., Appelman Z., Levin D., Or Y., Hagay Z.J. Head pushing versus reverse breech extraction in cases of impacted fetal head during Cesarean section. European Journal of Obstetrics & Gynecology and Reproductive Biology. 2005 Jul;121(1):24–6 | Non-randomised comparative retrospective cohort study with N<30 per treatment arm (28 cases delivered using ‘Push method’ and 20 cases using ‘Pull method’) |
| Magann E.F., Cleveland R.S., Dockery J.R., Chauhan S.P., Martin J.N., Morrison J.C. Acute tocolysis for fetal distress: terbutaline versus magnesium sulphate. Australian and New Zealand Journal of Obstetrics and Gynaecology 1993;33(4):362-4 | Ineligible population: Caesarean section for fetal distress; none of the women were reported to have experienced impacted fetal head |
| Malik N., Gupta A., Dahiya D., Nanda S., Singhal S.R., Perumal V. Caesarean Delivery in the Second Stage: Incidence, Effect, and How to Address Rising Rates. Journal of Gynecologic Surgery. 2021;37(1):10-5 | Non-randomised comparative study comparing 4 different delivery techniques with only one arm including N>30 women |
| Manning J.B., Tolcher M.C., Chandraharan E., Rose C.H. Delivery of an Impacted Fetal Head During Cesarean: A Literature Review and Proposed Management Algorithm. Obstetrical & Gynecological Survey 2015;70:719–24 | Literature review, included studies checked for relevance |
| Markin L.B., Rachkevych O.S., Zhemela O.M. Reverse breech extraction at cesarean section in second stage of labor. Wiadomosci lekarskie (Warsaw, Poland : 1960). 2020;73(5):1028-31 | Literature review, included studies checked for relevance |
| Matsubara S., Takahashi H., Usui R. Tydeman tube for impacted foetal head at caesarean section: Some concerns and suggestions. Journal of obstetrics and gynaecology : the journal of the Institute of Obstetrics and Gynaecology. 2018;38(1):146-7 | Letter to the Editor; no original data |
| Menticoglou S. Delivering the Impacted Head at Caesarean Section. Journal of Obstetrics and Gynaecology Canada. 2016;38(3):234 | Letter to the Editor; no original data |
| Mukhopadhyay P, Naskar T, Dalui R, Hazra S, Bhattacharya D. Evaluation of Patwardhan's technic - a four year study in a rural teaching hospital. Journal of Obstetrics and Gynaecology of India 2005;55(3):244-6 | Insufficient details about methods employed in group undergoing conventional method of delivery during lower segment caesarean section |
| Naghibi K. Randomized comparison of glyceryl trinitrate and volatile anesthetics to facilitate fetal extraction in caesarean section. Regional Anesthesia and Pain Medicine 2008;33(5 Suppl 1):137 | Conference abstract |
| Owens M., Bhullar A., Carlan S.J., O'Brien W.F., Hirano K. Effect of fundal pressure on maternal to fetal microtransfusion at the time of cesarean delivery. Journal of Obstetrics and Gynaecology Research 2003;29(3):152-6 | Ineligible population: Women undergoing elective (scheduled caesareans without labour) or emergency caesarean section (n=31 and n=53, respectively; results not reported separately) for the following reasons: arrest disorder, fetal distress in labour, repeat caesarean, malpresentation, prolapsed cord, macrosomia, previous shoulder dystocia, herpes simplex virus, leiomyoma, intrauterine |

| Study | Reason for exclusion |
|---|---|
| | growth retardation, postdates, maternal indications, and antepartum non-reassuring fetal surveillance |
| Papanikolaou N., Tillisi A., Louay L., Singh M., Ikomi A., Varma R. Reducing complications related to caesarean section in second stage: UK experience in the use of fetal disimpacting system (FDS). International Journal of Gynecology and Obstetrics. 2009:S304 | Abstract |
| Pergialiotis V., Vlachos D.G., Rodolakis A., Haidopoulos D., Thomakos N., Vlachos G.D. First versus second stage C/S maternal and neonatal morbidity: A systematic review and meta-analysis. European Journal of Obstetrics and Gynecology and Reproductive Biology. 2014;175(1):15-24 | Ineligible population: Systematic review comparing outcomes in women with first versus second stage of labour |
| Philpott R.H. Obstructed labour. Clinics in obstetrics and gynaecology | Narrative review, references pre-date 1980 |
| Radha P., Tagore S., Rahman M.F.A, Tee J. Maternal and perinatal morbidity after caesarean delivery at full cervical dilatation. Singapore Medical Journal. 2012;53(10):655-8 | Ineligible population: Analysis in women receiving versus not receiving glyceryl trinitrate; none of the women were reported to have experienced impacted fetal head |
| Raz N., Lurie S., Sadan O., Golan A., Boaz M. Comparison of maternal outcomes from primary cesarean section during the second compared with first stage of labor by indication for the operation. European Journal of Obstetrics and Gynecology and Reproductive Biology. 2014;182:43-7 | Ineligible comparison: Compares outcomes in women with first versus second stage of labour |
| Rice A., Tydeman G., Briley A., Seed P.T. The impacted foetal head at caesarean section: incidence and techniques used in a single UK institution. Journal of obstetrics and gynaecology : the journal of the Institute of Obstetrics and Gynaecology. 2019;39(7):948-51 | Non-comparative study (audit); incidence of using additional techniques to facilitate delivery of fetus |
| Safa H., Beckmann M. Comparison of maternal and neonatal outcomes from full-dilatation cesarean deliveries using the Fetal Pillow or hand-push method. MIDIRS Midwifery Digest. 2017;27(2):203 | Conference abstract of included study (Safa 2017) |
| Safa H., Wight K., Beckmann M. The use of the fetal pillow to deliver the fetal head at caesarean section at full dilatation. BJOG: An International Journal of Obstetrics and Gynaecology. 2015;122(SUPPL. 2):211 | Conference abstract of included study (Safa 2017) |
| Sarkar P., Das S. Prospective study evaluating the role of fetal pillow in caesareans section at full dilatation in reducing maternal and fetal morbidity. The American Journal of Obstetrics & Gynecology 2018;218:S242 | Abstract (poster); non-comparative study including 39 cases |
| Schwake D., Petchenkin L., Younis J.S. Reverse breech extraction in cases of second stage caesarean section. Journal of Obstetrics & Gynaecology 2012;32:548–51 | Non-randomised comparative retrospective cohort study with N<30 per treatment arm (29 cases were delivered by the reverse breech extraction manoeuvre and 21 women were delivered by the conventional approach) |

| Study | Reason for exclusion |
|---|---|
| Seal S., Mukherji J. A novel technique to reduce the complications of 2nd stage caesarean delivery using fetal pillow: A randomised controlled trial. BJOG: An International Journal of Obstetrics and Gynaecology. 2015;122(SUPPL. 2):375 | Conference abstract (possibly Seal 2016) |
| Seal S., Tibriwal R., De A., Kanrar P., Mukherjii J., Barman S.C. Reducing complications in a caesarean section at full dilation using fetal pillow: A prospective randomised trial. BJOG: An International Journal of Obstetrics and Gynaecology. 2013;120(SUPPL. 1):184 | Conference abstract (Possibly Seal 2016) |
| Seal S., Tibriwal R., Kanrar P., De A., Mukherjii J., Barman S.C. Elevating fetal head prior to performing a caesarean section at full dilation using fetal pillow: A prospective randomised trial. BJOG: An International Journal of Obstetrics and Gynaecology. 2015;122(SUPPL. 2):215 | Conference abstract of included study (Seal 2016) |
| Seal S.L., Dey A., Mukherji J., Barman S.C., Kamilya G., Mahsud-Dornan S. Fetal pillow: A novel device to reduce morbidity in a second stage caesarean section. A case controlled study. BJOG: An International Journal of Obstetrics and Gynaecology. 2012;119(SUPPL. 1):7-8 | Conference abstract of included study (Seal 2014) |
| Seal S.L, Dey A., Barman S.C., Kamilya ., Mukherji J. Does elevating the fetal head prior to delivery using a fetal pillow reduce maternal and fetal complications in a full dilatation caesarean section? A prospective study with historical controls. Journal of Obstetrics and Gynaecology. 2014;34(3):241-4 | Duplicate |
| Seal SL, Dey A, Barman SC, Kamilya G, Mukherji J, Onwude JL. Randomized controlled trial of elevation of the fetal head with a fetal pillow during cesarean delivery at full cervical dilatation. Int J Gynecol Obstet. 2016;133(2):178-82. | Study was retracted by the journal in June 2023 (Retraction. Int J Gynecol Obstet. https://doi.org/10.1002/ijgo.14924) due to discrepancies between the retrospective trial registration and the published article and considerable number of inconsistencies in the results presented. There was no patient data available to explain or clarify these inconsistencies. |
| Sengupta M, Dutta S. A comparative study of maternal and foetal outcome between reversed breech extraction technique and foetal pillow, during caesarean section in full dilatation (CSFD), in second stage of labour. J Evol Med Dent Sci 2019;8:1463–8 | Publication of the exact same data and study as Dutta 2019 (which is included) although the articles differ in what they call the intervention that is being compared to fetal pillow, with Dutta 2019 labelling it “modified Patwardhan technique” and Sengupta 2019 labelling it “reverse breech extraction”, but providing no definition for “reverse breech extraction” |
| Singh M., Varma R. Reducing complications associated with a deeply engaged head at caesarean section: a simple instrument. The Obstetrician & Gynaecologist 2008;10:38–41. Available from: https://obgyn.onlinelibrary. | Narrative review, included studies checked for relevance |

| Study | Reason for exclusion |
|---|---|
| wiley.com/doi/epdf/10.1576/toag.10.1.017.27372 | |
| Sritippayawan S., Chantrapitak W. Assisted delivery of high floating fetal head: A comparison of vacuum-assisted delivery with manual extraction. Asian Biomedicine. 2011;5(5):699-703 | Ineligible population and intervention: women undergoing elective caesarean using vacuum-assisted delivery versus manual extraction |
| Steer P.J. Is a fractured skull discovered in the neonate after caesarean section delivery always evidence of negligence? BJOG : an international journal of obstetrics and gynaecology. 2016;123(3):336 | Narrative commentary, references checked for relevance |
| Stolwijk N.N., de Jong P.R. An audit of the caesaid vacuum-assisted delivery cup compared with forceps delivery of the fetal head in caesarean section. South African Journal of Obstetrics and Gynaecology. 2019;25(1):20-4 | Mixed population and ineligible intervention/ comparison: Outcome data not reported separately for women undergoing elective or emergency caesarean section; vacuum cup (N=24.6% emergency caesarean section) versus forceps delivery (N=37.3% emergency caesarean section) |
| Sung J.F., Daniels K.I., Brodzinsky L., El-Sayed Y.Y., Caughey A.B., Lyell D.J. Cesarean delivery outcomes after a prolonged second stage of labor. American journal of obstetrics and gynecology. 2007;197(3):306.e1-5 | Ineligible intervention/comparison: Compares second stage of labour lasting 1 to 3 hours versus >4 hours, does not compare different delivery techniques |
| Tabiri-Essuman J. National Institute for Health and Care Excellence 2014:1–15 | NICE guideline, included studies checked for relevance |
| Terzic M., Kontic-Vucinic O. Fetal head impaction as a cause of iliofemoral phlebothrombosis: Contribution to the pathogenesis of deep vein thrombosis during pregnancy [1]. Thrombosis and Haemostasis. 2007;97(2):322 | Letter to the Editor describing additional case study (N=1) |
| Thornton J.G., Walker K.F. 'Negligent' technique for dis-impacting the fetal head at caesarean section: A scientific opinion paper. BJOG: An International Journal of Obstetrics and Gynaecology. 2013;120(SUPPL. 1):459 | Abstract; description of case study (N=1) |
| Verkayl D.A.A. The use of a balloon catheter for breech extraction. Tropical Doctor. 2002;32(4):244-5 | Case studies (N=2) |
| Vousden N., Briley A., Seed P.T., Shennan A.H., Tydeman G. Assessment of a vaginal device for delivery of the impacted foetal head at caesarean section. Journal of Obstetrics and Gynaecology. 2017;37(2):157-61 | Ineligible intervention; assesses Tydeman Tube in 10 women with no comparator group |
| Waterfall H., Grivell R.M., Dodd J.M. Techniques for assisting difficult delivery at caesarean section. The Cochrane Database of Systematic Reviews 2016:CD004944 | Systematic review, included studies checked for relevance |
| Wright M. Silcup extraction of the fetal head at elective caesarean section. 27th British Congress of Obstetrics and Gynaecology; 1995 July 4-7; Dublin, Ireland. 1995:Abstract no: 510 | Abstract; ineligible population: Women undergoing elective caesarean section |

| Study | Reason for exclusion |
|---|---|
| Wyn Jones N, Mitchell EJ, Wakefield N, Knight M, Dorling J, Thornton JG, et al. European Journal of Obstetrics & Gynecology & Reproductive Biology 2022, Vol. 272, Pages 77-81. | Observational report of a prospective cohort of which techniques were used for IFH in practice. Outcomes not reported separately for each method of delivery. |
| Zill-E-Huma R., Haran S., Mantovani E., Colley C., Subair S. Maternal and perinatal morbidity following caesarean delivery at full dilatation of cervix. BJOG: An International Journal of Obstetrics and Gynaecology. 2017;124(Supplement 2):50 | Conference abstract; insufficient information on study methods and techniques, and outcome data |
| Zimmermann, R. Difficult delivery of babies during cesarean section. Gynakologe. 2015;48(8): 599-610. | Article in German |
| Ziyauddin F., Hakim S., Khan T. Delivery of the deeply engaged fetal head during cesarean section in advanced labour: A comparative study of head pushing versus reverse breech extraction. Current Pediatric Research. 2013;17(1):41-3 | Article unavailable |
