# Supplementary material for: Management of impacted fetal head at cesarean birth: A systematic review and meta‐analysis
Source: Acta Obstet Gynecol Scand. 2024 May 24;103(9):1702–13. doi: 10.1111/aogs.14873 (PMC11324922; doi:10.1111/aogs.14873)
Supplement: Supplementary file 5 — Table S2. [file AOGS-103-1702-s005.pdf]

Supplementary Table S2: Full characteristics of included studies

Contents

1. Bansiwal 2017 2
2. Bastani 2012 5
3. Beeresh 2016 8
4. Bhattacharya 2020 12
5. Bhoi 2019 15
6. Chooi 2022 18
7. Dutta 2019 21
8. Fasubaa 2002 24
9. Frass 2011 27
10. Hanley 2020 29
11. Javed 2022 34
12. Keepanasseril 2019 37
13. Lal 2018 40
14. Lassey 2020 43
15. Lenz 2019 46
16. Nooh 2017 49
17. Rakholia 2019 52
18. Sacre 2021 55
19. Safa 2016 58
20. Saha 2014 61
21. Saleh 2014 64
22. Seal 2014 66
23. Tahir 2020 70
24. Veisi 2012 73

1. Bansiwal 2017

**Study details**	**Full citation:** Bansiwal R., Anand H., Jindal M. Safety of Patwardhan technique in deeply engaged head. Int J Reproduction Contracept Obstetrics Gynecol 2017;5:1562–5<br>**Study type:** Non-randomised comparative retrospective cohort study<br>**Country of study:** India<br>**Study dates:** 2011 to 2013
**Participants**	**Inclusion criteria:** Women undergoing caesarean section for obstruction in second stage of labour<br>**Exclusion criteria:** Not reported<br>**Participant characteristics:**<br>- Vaginal push up or Reverse breech extraction group:<br>o N women: 71<br>o Mean (SD) maternal age: Only presented in graph format. Mean or median age appears to be 23-24 years No further details can be reliably extracted.<br>o Maternal BMI: Not reported<br>o Smoking status: Not reported<br>o Parity: Range 0 to 3 (only presented in graph format. No further details can be reliably extracted.)<br>o Diabetes: Not reported<br>o Gestational age: Not reported<br>o Numbers with caesarean section at full (10cm) cervical dilatation: Not explicitly stated, but presumably 71, all women in second stage of labour<br>o Numbers with caesarean prior to (<10cm) full cervical dilatation: 0<br>- Patwardhan method group:<br>o N women: 64<br>o Mean (SD) maternal age: Only presented in graph format. Mean or median age appears to be 22-23 years. No further details can be reliably extracted.<br>o Maternal BMI: Not reported<br>o Smoking status: Not reported<br>o Parity: Range 0 to 4 (only presented in graph format. No further details can be reliably extracted.)<br>o Diabetes: Not reported<br>o Gestational age: Not reported<br>o Numbers with caesarean section at full (10cm) cervical dilatation: Not explicitly stated, but presumably 64, all women in second stage of labour<br>o Numbers with caesarean section prior to (<10cm) full cervical dilatation: 0
**Intervention & comparator**	**Intervention:** ‘ Vaginal push up or Reverse breech extraction’: “by pushing the deeply engaged head through vagina (push method), as cephalic by putting the hand in lower segment or by pulling the legs of the baby and delivering as breech (pull method)” (p. 1562).

	**Comparator:** ‘Patwardhan method’: In the "‘Patwardhan’s technique’, in case of occipito-anterior and transverse positions with the head deeply impacted in the pelvis, incision is made in the lower uterine segment, shoulders are present usually at incision level in deeply engaged head, the anterior shoulder is delivered out by hooking the arm first by hooking the arm. With gentle traction on this shoulder, the posterior shoulder is also delivered out. Next, the surgeon holds the trunk of baby gently with both thumbs parallel to spine and with fundal pressure given by assistant the buttocks are delivered followed by legs. Now the baby’s head which is the only part of the fetus which is still inside the uterus, is gently lifted out of the pelvis by making an arc" (p. 1563).<br>"‘Modified Patwardhan’s technique’, in case of occipito-posterior position with the head deeply impacted in the pelvis, incision is made in the lower uterine segment, shoulders are present usually at incision level in deeply engaged head, the anterior shoulder is delivered out by hooking the arm first by hooking the arm followed by delivering the same side leg. The other side leg is then delivered gently followed by same side arm. Buttocks and the trunk of baby and are delivered by gently pulling baby legs. Lastly the baby head is delivered" (p.1563).
**Primary outcomes and results**	**Maternal:**<br>• Uterine incision extension (angle extensions on lower segments and / or into broad ligaments):<br>○ Incision extension on lower segment (at angles or towards cervix): Vaginal push up or Reverse breech extraction’: 17/71; ‘Patwardhan method’: 2/64 (p=0.01)<br>○ Angle extensions into broad ligaments: Not reported<br>• Operative blood loss / postpartum haemorrhage (operative blood loss > 500ml): Only post-partum haemorrhage reported: Study reported traumatic post-partum haemorrhage (amount of blood loss not defined). ‘Vaginal push up or Reverse breech extraction’: 16/71; ‘Patwardhan method’: 1/64 (p=0.01)<br>• Operative time (duration of surgery): Not reported<br>**Perinatal:**<br>• Infant birth trauma (skull fracture / intracranial haemorrhage / other bony fracture / nerve injury): Not reported<br>• Apgar score at five minutes: Although the authors do report some data on this outcome, it is unclear what exactly these data reflect (e.g., means/medians/count?)
**Secondary outcomes and results**	**Maternal:**<br>• Blood transfusion: ‘Vaginal push up or Reverse breech extraction’: 16/71; ‘Patwardhan method’: 1/64 (p=0.01)<br>• Inverted T or J incision: Not reported<br>• Visceral injury (ureteral / bladder / cervical) or hysterectomy:<br>○ Uterine incision extension into cervix / vagina: Not reported<br>○ Injury to urinary tract (including ureteric injury and bladder injury): Not reported<br>○ Hysterectomy: Not reported<br>• Infection (wound infection / endometritis / maternal sepsis):<br>○ Wound infection: Not reported<br>○ Endometritis: Not reported

	○ Urinary tract infection: Not reported<br>○ Post-partum pyrexia / maternal sepsis: Not reported<br>• Duration of hospital stay: Not reported<br>• Decision-to-delivery interval: Not reported<br>• Incision-to-delivery interval: Not reported<br>**Perinatal:**<br>• NICU (Neonatal Intensive Care Unit) admission: 'Vaginal push up or Reverse breech extraction': 19/71; 'Patwardhan method': 19/64 (p=0.34)<br>• Umbilical artery pH / cord pH < 7.10: Not reported<br>• Neonatal death (defined as death within the first 28 days of life): Not reported separately for intervention groups (early neonatal death: n=3; severe birth asphyxia due to prolonged second stage arrest)<br>Cost: Not reported
**Risk of bias**	**Assessed by ROBINS-I:**<br>• Bias due to confounding: Serious risk of bias - the authors acknowledged that the caesarean sections were performed by different senior residents, and therefore the skill of individual surgeons differed, which, the authors reported, explains fewer uterine incision extensions in the 'Patwardhan method' group; the authors did not adjust for confounding in any of the analyses.<br>• Bias in selection of participants into study: Low risk of bias - selection of participants does not appear to be related to both intervention and outcomes; initial follow-up time appears to be similar for all women.<br>• Bias in classification of interventions: Moderate risk of bias – clear definitions provided for 'Patwardhan method' but limited details provided on 'Vaginal push up or Reverse breech extraction'.<br>• Bias due to deviations from intended intervention: Low risk of bias – no deviations from the intended intervention reported.<br>• Bias due to missing data: Low risk of bias – no reported missing outcome data.<br>• Bias on measurement of outcomes: Unclear risk of bias – lack of information regarding how some outcomes were defined or measured<br>• Bias in selection of the reported result: Critical risk of bias - no mention of pre-registered protocol or statistical analysis plan, and published in a potential predatory journal according to Beall's List of Potential Predatory Journals and Publishers.<br>Overall risk of bias: Critical risk of bias.
**Source of funding**	None

2. Bastani 2012

**Study details**	**Full citation:** Bastani P., Pourabolghase S., Abbasalizadeh F., Motvalli L. Comparison of neonatal and maternal outcomes associated with head-pushing and head-pulling methods for impacted fetal head extraction during cesarean delivery. International Journal of Gynecology & Obstetrics. 2012 Jul;118(1):1–3<br>**Study type:** Randomised controlled trial<br>**Country of study:** Iran<br>**Study dates:** June 2008 to January 2010
**Participants**	**Inclusion criteria:** Women with in the second stage of delivery with very low station of fetal head; dystocia; and vertex presentation<br>**Exclusion criteria:** Women with multiple pregnancy; fetal anomalies; previous caesarean section; and premature delivery<br>**Participant characteristics:** All women had a fully dilated cervix, arrest of descent for more than 1 hour and obstructed labour at station +2<br>- Vaginal push up group:<br>o N women: 30<br>o Mean (SD) maternal age: 27.2 (4.3) years<br>o Mean (SD) maternal weight: 63 (9.7) kg<br>o Smoking status: Not reported<br>o Parity: Not reported<br>o Diabetes: Not reported<br>o Mean (SD) gestational age: 39.6 (1.3) weeks<br>o Numbers with caesarean section at full (10cm) cervical dilatation: 30, all women had a fully dilated cervix<br>o Numbers with caesarean prior to (<10cm) full cervical dilatation: 0<br>- Reverse breech extraction group:<br>o N women: 29<br>o Mean (SD) maternal age: 28.7 (4.6) years<br>o Mean (SD) maternal weight: 66 (9.3) kg<br>o Smoking status: Not reported<br>o Parity: Not reported<br>o Diabetes: Not reported<br>o Mean (SD) gestational age: 39.6 (1.4) weeks<br>o Numbers with caesarean section at full (10cm) cervical dilatation: 29, all women had a fully dilated cervix<br>o Numbers with caesarean section prior to (<10cm) full cervical dilatation: 0
**Intervention & comparator**	**Intervention:** 'Vaginal push up': "In the push group, an assistant helped to dislodge the head vaginally, and the surgeon delivered the dislodged head through the uterine incision; the rest of the fetus was delivered via routine caesarean techniques" (p. 1-2).

	**Comparator:** 'Reverse breech extraction': "In the pull (reverse breech) group, the surgeon inserted their hand toward the upper segment of the uterus and searched for the fetal leg; gentle traction was then applied until the second leg appeared. While holding both legs, the surgeon gently pulled the fetus up and out of the uterus through the uterine incision—similar to an assisted vaginal breech delivery" (p. 2).<br><br>Women without complications were discharged on the third post-operative day.
**Primary outcomes and results**	**Maternal:**<br>• Uterine incision extension (angle extensions on lower segments and / or into broad ligaments):<br>○ Incision extension on lower segment (at angles or towards cervix): Not reported<br>○ Angle extensions into broad ligaments: Study reported uterine incision extension to ligaments: 'Vaginal push up': 15/30; 'Reverse breech extraction': 5/29 (p=0.008);<br>• Operative blood loss / postpartum haemorrhage (operative blood loss > 500ml): Not reported<br>• Operative time (duration of surgery): 'Vaginal push up': Mean (SD) = 75.6 (12.2) minutes; 'Reverse breech extraction': Mean (SD) = 75.5 (10.8) minutes (p=0.987)<br>**Perinatal:**<br>• Infant birth trauma (skull fracture / intracranial haemorrhage / other bony fracture / nerve injury): Study reported this outcome defined as bony fracture / other types of fetal injury: 'Vaginal push up': 0/30; 'Reverse breech extraction': 0/29<br>• Apgar score at five minutes: 'Vaginal push up': Mean (SD) = 7.7 (0.5); 'Reverse breech extraction': Mean (SD) = 7.6 (0.6) (p=0.62)
**Secondary outcomes and results**	**Maternal:**<br>• Blood transfusion: Vaginal push up': 3/30; 'Reverse breech extraction': 1/29(p=0.61)<br>• Inverted T or J incision: See uterine incision extension into cervix / vagina<br>• Visceral injury (ureteral / bladder / cervical) or hysterectomy:<br>○ Uterine incision extension into cervix / vagina: The authors reported this outcome as uterine incision extension to vagina/need for J incision (which involves extension of Kerr incision toward the upper segment of the uterus in order to enlarge the incision): 'Vaginal push up': 3/30; 'Reverse breech extraction': 4/29 (p=0.7)<br>○ Injury to urinary tract (including ureteric injury and bladder injury): Study reported this outcome defined as ureteral / bladder injury, hypogastric vessel ligation or hysterectomy: 'Vaginal push up': 0/30; 'Reverse breech extraction': 0/29<br>○ Hysterectomy: See injury to urinary tract<br>• Infection (wound infection / endometritis / maternal sepsis):<br>○ Wound infection: 'Vaginal push up': 4/30 (p=0.35); 'Reverse breech extraction': 1/29<br>○ Endometritis: Not reported<br>○ Urinary tract infection: 'Vaginal push up': 10/30; 'Reverse breech extraction': 0/29 (p<0.001)

	○ Post-partum pyrexia / maternal sepsis: The study reported this outcome as fever after delivery: 'Vaginal push up': 16/30; 'Reverse breech extraction': 3/29 (p<0.001)<br>• Duration of hospital stay: 'Vaginal push up': Mean (SD) = 2.8 (0.6) days; 'Reverse breech extraction': Mean (SD) = 2.9 (0.6) days (p=0.69)<br>• Decision-to-delivery interval: Not reported<br>• Incision-to-delivery interval: Not reported<br>**Perinatal:**<br>• NICU (Neonatal Intensive Care Unit) admission: 'Vaginal push up': 0/30; 'Reverse breech extraction': 0/29<br>• Umbilical artery pH: 'Vaginal push up': Mean (SD) = 7.21 (0.08); 'Reverse breech extraction': Mean (SD) = 7.22 (0.07) (p=0.83)<br>• Neonatal death (defined as death within the first 28 days of life): 'Vaginal push up': 0/30; 'Reverse breech extraction': 0/29<br>Cost: Not reported
**Risk of bias**	**Assessed by RoB2:**<br>• Risk of bias arising from the randomisation process: Some concerns – the authors stated that the women were randomly assigned to the study groups, but no other details provided.<br>• Risk of bias due to deviations from the intended interventions (effect of assignment to intervention): Low risk of bias – operative blood loss estimated by the anaesthetist, who was blinded to the study; no deviations from intended intervention.<br>• Risk of bias due to missing outcome data: Low risk of bias – no reported missing outcome data.<br>• Risk of bias in measurement of the outcome: Low risk of bias – outcomes measured using objective measurement tools; comparable outcome detection methods and thresholds used, and same definitions and measurements.<br>• Risk of bias in selection of the reported result: Some concerns - no details provided.<br>Overall risk of bias: Some concerns.
**Source of funding**	Not reported

3.Beeresh 2016

**Study details**	**Full citation:** Beeresh C. S., Divyasree D., Pradeep S., Krishna L. Disengagement of the deeply engaged fetal head during caesarean section in advanced labor: Patwardhan versus push extraction. Int J Reprod Contracept Obstet Gynecol. 2016 Jan;5(1):68-73.<br><br>**Study type:** Non-randomised comparative retrospective cohort study<br><br>**Country of study:** India<br><br>**Study dates**: May 2012 to April 2015
**Participants**	**Inclusion criteria:** Women with single fetus at term in anterior vertex position, with the head deeply impacted in pelvis and needing cesarean delivery.<br><br>**Exclusion criteria:** intrauterine fetal death, congenital fetal anomaly, multiple pregnancy, preterm caesarean, previous caesarean section.<br><br>**Participant characteristics:**<br><br>- Vaginal push up group (Group B):<br>o N women: 52<br>o Mean (SD) maternal age: Not reported<br>o Mean (SD) maternal BMI: Not reported<br>o Smoking status: Not reported<br>o Parity: Not reported<br>o Diabetes: Not reported<br>o Mean (SD) gestational age: 39.14 (1.0) weeks<br>o Numbers with caesarean section at full (10cm) cervical dilatation: 52 (100%). Study reports 5.41% of all deliveries were second stage caesarean and also that 5.41% were at full dilatation.<br>o Numbers with caesarean section prior to (<10cm) full cervical dilatation: 0<br>- Patwardhan method group (Group A):<br>o N women: 46<br>o Mean (SD) maternal age: Not reported<br>o Mean (SD) maternal BMI: Not reported<br>o Smoking status: Not reported<br>o Parity: Not reported<br>o Diabetes: Not reported<br>o Mean (SD) gestational age: 38.85 (0.85) weeks

	○ Numbers with caesarean section at full (10cm) cervical dilatation: 46 (100%). Study reports 5.41% of all deliveries were second stage caesarean and also that 5.41% were at full dilatation.<br>○ Numbers with caesarean prior to (<10cm) full cervical dilatation: 0
**Intervention & comparator**	**Intervention:** “Patwardhan Technique”: “In case of occipito-transverse or occipito-anterior positions with the head deeply impacted in the pelvis, incision is made in the lower uterine segment, at the level of the anterior shoulder, which is delivered out. With gentle traction on this shoulder, the posterior shoulder is also delivered out. Next, the surgeon hooks the fingers through both the axillae and with gentle traction, aided by fundal pressure applied by assistant, the body of the foetus is brought out of the uterus. Now the baby’s head which is the only part of the foetus which is still inside the uterus is gently lifted out of the pelvis.”<br><br>**Comparator:** ‘Push method’: After opening the uterus, the patient is positioned in the supine position with the knees flexed and the lower legs abducted by two assistants. One of the assistants under sterile condition introduces his gloved finger into the vagina and then pushes the head up disimpacting it. The surgeon then introduces his hand into the uterus between the fetal head and the uterine wall, manoeuvring his hand downwards to get beneath the fetal head as the assistant disimpacts the fetal head from below. The patient’s legs are then returned to normal position. The Surgeon then delivers the fetal head and the rest of the fetus as it is performed in routine caesarean section.
**Primary outcomes and results**	**Maternal:**<br><br>• Uterine incision extension (angle extensions on lower segments and / or into broad ligaments): Study appears to report extension of uterine incision involving the lower segment, as description of Patwardhan method states:- “incision is made in the lower uterine segment”, however this is not explicitly stated and unclear for vaginal push-up group”:<br>○ Incision extension on lower segment (at angles or towards cervix): ‘Vaginal push up’: 18/52; ‘Patwardhan method’: 4/46 (p=0.0031)<br>• Angle extensions into broad ligaments: Not reported<br>• Operative blood loss / post-partum haemorrhage (operative blood loss >1000 ml): Study reports ‘traumatic PPH’ and ‘atonic’ PPH but does not give definitions for these. ‘Traumatic PPH’: ‘Vaginal push up’: 13/52; ‘Patwardhan method’: 2/46 (p=0.0049); ‘atonic PPH: ‘Vaginal push up’: 5/52; ‘Patwardhan method’ 2/46.<br>• Operative time (duration of surgery): Not reported<br><br>• **Perinatal:**<br>• Infant birth trauma (skull fracture / intracranial haemorrhage / other bony fracture / nerve injury): The study reported ‘fetal injury’ without specifying the type of injury. ‘Vaginal push up’: 0/52; ‘Patwardhan method’ : 2/46 (p= 0.4967)<br>• Apgar score at five minutes / Apgar score < 7 at five minutes: Study reports Apgar score </=7 at 5 minutes: ‘Vaginal push up’: 7/52; ‘Patwardhan method’: 5/46 (p=0.7652)

**Secondary outcomes and results**	**Maternal:**<br>• Blood transfusion: 'Vaginal push up': 20/52; 'Patwardhan method': 8/46 (p=0.0259)<br>• Inverted T or J incision: Not reported<br>• Visceral injury (ureteral / bladder / cervical) or hysterectomy:<br>○ Uterine incision extension into cervix / vagina: Not reported<br>○ Injury to urinary tract (including ureteric injury and bladder injury): The study reported 'bladder injury': 'Vaginal push up': 2/52; 'Patwardhan method': 0/46 (p=0.4967).<br>○ Hysterectomy: 'Vaginal push up': 2/52; 'Patwardhan method': 0/46 (p=0.4967).<br>• Infection (wound infection / endometritis / maternal sepsis):<br>○ Wound infection: Not reported<br>○ Endometritis: Not reported<br>○ Urinary tract infection: Not reported<br>○ Post-partum pyrexia / maternal sepsis: Not reported<br>• Duration of hospital stay: Not reported<br>• Decision-to-delivery interval: Not reported<br>• Incision-to-delivery interval: Not reported<br>**Perinatal:**<br>• NICU (Neonatal Intensive Care Unit) admission: 'Vaginal push up': 11/52; 'Patwardhan method': 9/46 (p=1.0000)<br>• Umbilical artery pH / cord pH < 7.10: Not reported<br>• Neonatal death (defined as death within the first 28 days of life): Study reported 'stillbirth': 'Vaginal push up': 2/52; 'Patwardhan method': 1/46 (p=1.0000)<br>Cost: Not reported
**Risk of bias**	**Assessed by ROBINS-I:**<br>• Bias due to confounding: Serious risk of bias – only limited baseline parameters reported so limited comparisons between baseline groups possible; the authors did not adjust for confounding in any of the analyses.<br>• Bias in selection of participants into study: Serious risk of bias - the decision for the performance of the Patwardhan extraction or to go on with push approach was taken in the operating theatre by the operating surgeon. Criteria for decision unclear.<br>• Bias in classification of interventions: Low risk of bias – clear definitions provided for 'Vaginal push up' and 'Patwardhan method'.<br>• Bias due to deviations from intended intervention: Unclear risk of bias – not reported.<br>• Bias due to missing data: Unclear risk of bias – outcomes reported for all women, but limited outcomes reported.

	• Bias on measurement of outcomes: Moderate risk of bias - unclear definition of some outcomes e.g. position of uterine incision extension not reported, criteria not reported.<br>• Bias in selection of the reported result: Critical risk of bias - No mention of pre-registered protocol or statistical analysis plan, and published in a potential predatory journal according to Beall's List of Potential Predatory Journals and Publishers.<br>• Overall risk of bias: Critical risk of bias.
**Source of funding**	Not reported

4. Bhattacharya 2020

**Study details**	**Full citation:** Bhattacharya R., Ramesh A.C. Cesarean Section of an Impacted Fetal Head at Full Cervical Dilatation – Evaluation of Patwardhan Technique. Crit Care Obst Gyne. 2020, vol 6 (4:9).<br>**Study type:** Non-randomised comparative retrospective cohort study<br>**Country of study:** India<br>**Study dates**: October 2018 to September 2019
**Participants**	**Inclusion criteria:** Women with single fetus at term in anterior vertex position with full cervical dilatation with head deeply impacted in the maternal pelvis that underwent second stage caesarean section with fetal head at or below the level of ishchial spine.<br>**Exclusion criteria:** Intrauterine fetal death, congenital fetal anomaly, multiple pregnancy, ruptured uterus, previous caesarean section, antepartum hemorrhage, pregnancy less than 37 weeks.<br><br>**Participant characteristics:**<br>- Vaginal push up group ('Push' group, Group 2):<br>o N women: 50<br>o Mean (SD) maternal age: 23.78 (4.13) years<br>o Mean (SD) maternal BMI: Not reported<br>o Smoking status: Not reported<br>o Parity: Mean (SD) 1.98 (0.67)<br>o Diabetes: Not reported<br>o Mean (SD) gestational age: 38.42 (2.05) weeks<br>o Numbers with caesarean section at full (10cm) cervical dilatation: 50 (100%) as per the inclusion criteria<br>o Numbers with caesarean section prior to (<10cm) full cervical dilatation: 0 as per the inclusion criteria<br>- Patwardhan method group (Group 1):<br>o N women: 50<br>o Mean (SD) maternal age: 23.16 (4.21) years<br>o Mean (SD) maternal BMI: Not reported<br>o Smoking status: Not reported<br>o Parity: Mean (SD) 1.89 (0.54)<br>o Diabetes: Not reported<br>o Mean (SD) gestational age: 38.14 (2.04) weeks<br>o Numbers with caesarean section at full (10cm) cervical dilatation: 50 (100%) as per the inclusion criteria<br>o Numbers with caesarean prior to (<10cm) full cervical dilatation: 0 as per the inclusion criteria
**Intervention & comparator**	**Intervention:** Patwardhan method: In cases with occipito-anterior position with head deeply impacted in the pelvis, an incision is made in the lower uterine segment at the level of the anterior shoulder which is delivered out. With gentle traction on this shoulder, the posterior shoulder is also delivered out. Next, the surgeon hooks the fingers through the

	axillae and with gentle traction, aided by fundal pressure applied by assistant, the body of the fetus is brought out of the uterus.<br><br>**Comparator:** Vaginal push-up (‘Push method’): In the Push Method after opening the uterus, fetal head is pushed up by an assistant′s hand introduced through the vagina. The surgeon then introduces his hand into the uterus, between the baby′s head & uterine wall, to get beneath the fetal head as the assistant pushes the fetal head up from down below. The surgeon then delivers the fetal head and rest of the body as in routine caesarean sections.
**Primary outcomes and results**	**Maternal:**<br>○ Uterine incision extension (angle extensions on lower segments and / or into broad ligaments): Study reported ‘uterine incision extension’ without any further details. However, in the discussion it states ‘impaction of fetal head which gives rise to a thin, easily lacerated lower uterine segment and cervix, which is predisposed to more extensions while delivering fetal head’ which may imply that the ‘uterine incision extension’ reported relates to the lower segment.<br>○ Incision extension on lower segment (at angles or towards cervix): ‘Vaginal push up’: 8/50 (16%); Patwardhan method’: 1/50 (2%) (p=0.009).<br>• Angle extensions into broad ligaments: Not reported<br>• Operative blood loss / post-partum haemorrhage (operative blood loss >1000 ml): Not reported<br>• Operative time (duration of surgery): Not reported<br>**Perinatal:**<br>• Infant birth trauma (skull fracture / intracranial haemorrhage / other bony fracture / nerve injury): The study reported ‘fetal injury’ without specifying the type of injury. ‘Vaginal push up’ (push): 2/50 (4%); Patwardhan method: 3/50 (6%).<br>• Apgar score at five minutes / Apgar score < 7 at five minutes: Study reports mean Apgar score at 5 mins only; Mean (SD): ‘Vaginal push up’ (push): 7.18 (0.74); Patwardhan method: 6.68 (0.78) (p=0.01).
**Secondary outcomes and results**	**Maternal:**<br>• Blood transfusion: ‘Vaginal push up’: 19/50 (38%); ‘Patwardhan method’: 12/50 (24%) (p=0.172)<br>• Inverted T or J incision: Not reported<br>• Visceral injury (ureteral / bladder / cervical) or hysterectomy:<br>○ Uterine incision extension into cervix / vagina: Not reported<br>○ Injury to urinary tract (including ureteric injury and bladder injury): The study reported ‘bladder injury’ only: ‘Vaginal push up’ (push): 3/50 (6%); Patwardhan method: 1/50 (2%) (p=0.617)<br><br>• Hysterectomy: ‘Vaginal push up’ (push): 1/50 (2%); Patwardhan method: 0/50 (0%) (p=1.000)<br>• Infection (wound infection / endometritis / maternal sepsis):<br>○ Wound infection: Not reported

	○ Endometritis: Not reported<br>○ Urinary tract infection: Not reported<br>○ Post-partum pyrexia / maternal sepsis: Not reported<br>• Duration of hospital stay: Not reported<br>• Decision-to-delivery interval: Not reported<br>• Incision-to-delivery interval: Not reported<br>**Perinatal:**<br>• NICU (Neonatal Intensive Care Unit) admission: ‘Vaginal push up’: (0-2 days): 17/50, (5-10 days) 16/50, >10 days 16/50; ‘Patwardhan method’: (0-2 days): 17/50, (5-10 days) 16/50, >10 days 16/50.<br>• Umbilical artery pH / cord pH < 7.10: Not reported<br>• Neonatal death (no definition given): ‘Vaginal push up’: 7/50 (14%); ‘Patwardhan method’: 6/50 (12%) (p=0.766)<br>Cost: Not reported
**Risk of bias**	**Assessed by ROBINS-I:**<br>• Bias due to confounding: Serious risk of bias - only limited baseline parameters reported so limited comparisons between baseline groups possible; The authors did not adjust for confounding in any of the analyses.<br>• Bias in selection of participants into study: Serious risk of bias – criteria for selection for each technique unclear.<br>• Bias in classification of interventions: Low risk of bias – clear definitions provided for ‘Vaginal push up’ and ‘Patwardhan method’.<br>• Bias due to deviations from intended intervention: Unclear risk of bias – not reported.<br>• Bias due to missing data: Unclear risk of bias – outcomes reported for all women, but limited outcomes reported.<br>• Bias on measurement of outcomes: Moderate risk of bias - unclear definition of some outcomes e.g. position of uterine incision extension not reported, criteria not reported.<br>• Bias in selection of the reported result: Critical risk of bias - no mention of pre-registered protocol or statistical analysis plan, and published in a potential predatory journal according to Beall’s List of Potential Predatory Journals and Publishers.<br>• Overall risk of bias: Critical risk of bias.
**Source of funding**	Not reported

5. Bhoi 2019

**Study details**	**Full citation:** Bhoi N.R., Nayak L., Sethy M., Pradhan K., Mishra P., Mahapatra T., Bera P. Lower segment cesarean section in second stage of labor: comparison of patwardhan method with conventional pushing method (a 3-year study). Journal of SAFOG. 2019;11(4):263-5<br>**Study type:** Randomised controlled study<br>**Country of study:** India<br>**Study dates**: April 2012 to March 2015
**Participants**	**Inclusion criteria:** Women in advanced labour with deeply impacted fetal head<br>**Exclusion criteria:** Women with non-vertex presentation with labour, existing maternal disease, pregnancy complications such as gestational diabetes or gestational hypertension, major fetal anomaly<br>**Participant characteristics:**<br>- ‘Vaginal push up or Reverse breech extraction’ group:<br>o N women: 291<br>o Maternal age: <25 years: 86 (30%); 25 to 30 years: 122 (42%); >30 years: 82 (28%)<br>o Maternal weight: Not reported<br>o Smoking status: Not reported<br>o Parity: Not reported<br>o Diabetes: Not reported<br>o Gestational age: <37 weeks: 26 (9%); 37 to 40 weeks: 218 (75%); >40 weeks: 47 (16%)<br>o Numbers with caesarean section at full (10cm) cervical dilatation: Not reported<br>o Numbers with caesarean prior to (<10cm) full cervical dilatation: Not reported<br>- ‘Patwardhan method’ group:<br>o N women: 129<br>o Maternal age: <25 years: 34 (26%); 25 to 30 years: 62 (48%); >30 years: 34 (26%)<br>o Maternal weight: Not reported<br>o Smoking status: Not reported<br>o Parity: Not reported<br>o Diabetes: Not reported<br>o Gestational age: <37 weeks: 9 (7%); 37 to 40 weeks: 104 (81%); >40 weeks: 16 (12%)<br>o Numbers with caesarean section at full (10cm) cervical dilatation: Not reported<br>o Numbers with caesarean section prior to (<10cm) full cervical dilatation: Not reported
**Intervention & comparator**	**Intervention:** ‘Vaginal push up or Reverse breech extraction’: No details provided.<br><br>**Comparator:** ’Patwardhan method’: “In case of occipit transverse or occipit anterior positions with the head deeply impacted in the pelvis, incision was made in the lower uterine segment, at the level of the anterior shoulder, which was delivered out. With gentle traction on this shoulder, the posterior shoulder was also delivered out. Next, the surgeon

	hooks the fingers through both the axillae and with gentle traction, aided by fundal pressure applied by assistant, the body of the fetus was brought out of the uterus. Now the baby's head which was the only part of the fetus which was still inside the uterus, was gently lifted out of the pelvis" (p. 264).
**Primary outcomes and results**	**Maternal:**<br>• Uterine incision extension (angle extensions on lower segments and / or into broad ligaments):<br>○ Incision extension on lower segment (at angles or towards cervix): 'Vaginal push up or Reverse breech extraction': 34/291; 'Patwardhan method': 0/129 (p<0.0001)<br>○ Angle extensions into broad ligaments: Not reported<br>• Operative blood loss / post-partum haemorrhage (operative blood loss > 500ml): Not reported<br>• Operative time (duration of surgery): 'Vaginal push up or Reverse breech extraction': Mean (SD) = 59.14 (21.92) minutes; 'Patwardhan method': Mean (SD) = 52.56 (12.44) minutes (p<0.0001)<br>**Perinatal:**<br>• Infant birth trauma (skull fracture / intracranial haemorrhage / other bony fracture / nerve injury): This outcome was not fully reported, but the authors stated that there was no increased risk of neonatal injuries or asphyxia with the 'Patwardhan method', as compared to 'Push and Pull' method (i.e. 'Vaginal push up or Reverse breech extraction')<br>• Apgar score at five minutes / Apgar score < 7 at five minutes: Study reported Apgar score <3 at 5 minutes: 'Vaginal push up or Reverse breech extraction': 38/291; 'Patwardhan method': 10/129 (p<0.0001)
**Secondary outcomes and results**	**Maternal:**<br>• Blood transfusion: 'Vaginal push up or Reverse breech extraction': 39/291; 'Patwardhan method': 6/129 (p<0.0001)<br>• Inverted T or J incision: Not reported<br>• Visceral injury (ureteral / bladder / cervical) or hysterectomy:<br>○ Uterine incision extension into cervix / vagina: Not reported<br>○ Injury to urinary tract (including ureteric injury and bladder injury): Not reported<br>○ Hysterectomy: Not reported<br>• Infection (wound infection / endometritis / maternal sepsis):<br>○ Wound infection: Not reported<br>○ Endometritis: Not reported<br>○ Urinary tract infection: Not reported<br>○ Post-partum pyrexia / maternal sepsis: Not reported<br>• Duration of hospital stay: 'Vaginal push up or Reverse breech extraction': Mean (SD) = 7.73 (5.44) days; 'Patwardhan method': Mean (SD) = 7.25 (4.44) days (p=0.06)<br>• Decision-to-delivery interval: Not reported<br>• Incision-to-delivery interval: Not reported<br>**Perinatal:**

	• NICU (Neonatal Intensive Care Unit) admission: 'Vaginal push up or Reverse breech extraction': 84/291; 'Patwardhan method': 30/129 ($p<0.0001$)<br>• Umbilical artery pH / cord pH < 7.10: Not reported<br>• Neonatal death (defined as death within the first 28 days of life): Not reported<br>Cost: Not reported
**Risk of bias**	**Assessed by RoB2:**<br>• Risk of bias arising from the randomisation process: Some concerns – the authors reported that women were randomly divided into two groups, but no details of the randomisation process or allocation concealment reported. Imbalance in sample size between groups not explained.<br>• Risk of bias due to deviations from the intended interventions (effect of assignment to intervention): Low risk of bias – no information provided relating to blinding to treatment assignment; no deviations from intended intervention.<br>• Risk of bias due to missing outcome data: Low risk of bias – no reported missing outcome data.<br>• Risk of bias in measurement of the outcome: Low risk of bias – outcomes measured using objective measurement tools; comparable outcome detection methods and thresholds used, and same definitions and measurements.<br>• Risk of bias in selection of the reported result: Some concerns - no details provided.<br>Overall risk of bias: High risk of bias
**Source of funding**	Not reported

6. Chooi 2022

**Study details**	**Full citation:** Chooi KYL, Deussen AR, Louise J, Cash S, Dodd JM. Maternal and neonatal outcomes following the introduction of the Fetal Pillow at a tertiary maternity hospital: A retrospective cohort study. Aust N Z J Obstet Gynaecol. Epub 2022 Dec 8. 2023 Jun;63(3):360-364.<br>**Study type:** Non-randomised comparative retrospective cohort study<br>**Country of study:** Australia<br>**Study dates**: October 2018 to December 2019
**Participants**	**Inclusion criteria:** Women undergoing singleton pregnancy at term gestation in the second stage of labour requiring caesarean section at full dilation. Women who were 7 cm dilated and above, but not fully dilated, were also included.<br>**Exclusion criteria:** Women <7cm dilated.<br>**Participant characteristics:**<br>- Fetal pillow group:<br>o N women: 53<br>o Mean (SD) maternal age: 31.00 (5.14) years<br>o Mean (SD) maternal BMI: (N=53) 25.92 (5.34) kg/m^2<br>o Smoking status: Not reported<br>o Mean (SD) parity: mean not reported; parity 0: n=43 (81.13%); parity 1+: n=10 (18.87%)<br>o Diabetes: Not reported<br>o Gestational age: mean (SD): 39.58 (1.14) weeks<br>o Numbers with caesarean section at full (10cm) cervical dilatation: Not stated,<br>o Numbers with caesarean section prior to (<10cm) full cervical dilatation: Not stated, some women were included who were 7cm and above dilated but numbers not reported.<br>- No fetal pillow group:<br>o N women: 48<br>o Mean (SD) maternal age: 29.87 (5.38) years<br>o Mean (SD) maternal BMI: 25.95 (4.45) kg/m^2<br>o Smoking status: Not reported<br>o Mean (SD) parity: mean not reported; parity 0: n=34 (70.83%); parity 1+: n=14 (29.17%)<br>o Diabetes: Not reported<br>o Gestational age: mean: 39.33 (1.33) weeks<br>o Numbers with caesarean section at full (10cm) cervical dilatation: Not stated<br>o Numbers with caesarean prior to (<10cm) full cervical dilatation: Not stated, some women were included who were 7cm and above dilated but numbers not reported.
**Intervention & comparator**	**Intervention:** Fetal pillow: “The fetal pillow is a silicone balloon device designed to gently elevate a

	deeply engaged fetal head out of the pelvis to minimise trauma. It is inserted into the vagina with sterile lubrication. The silicone balloon is positioned posterior to the fetal head with the base plate against the pelvic floor. It is inflated with 180 mL of normal saline, and after the birth is achieved, the balloon is deflated and removed."<br>**Comparator:** No fetal pillow
**Primary outcomes and results**	**Maternal:**<br>• Uterine incision extension (angle extensions on lower segments and / or into broad ligaments):<br>○ Incision extension on lower segment (at angles or towards cervix): Not reported. Study reported 'uterine incision extension' without any further details. Uterine incision extension: Fetal pillow: 11/53 (20.75%); No fetal pillow 12/48 (25.00%); estimated relative risk (95% CI): 0.83 (0.40, 1.72), p=0.612.<br>○ Angle extensions into broad ligaments: Not reported<br>• Operative blood loss / post-partum haemorrhage (operative blood loss >500ml): Study reported 'blood loss' (ml): Fetal pillow (N=53): Mean (SD) = 699.06 (393.88) ml; No-fetal pillow (N=48): Mean (SD) = 797.92 (488.26) ml; estimated relative risk (95% CI): −98.86 (−273.30, 75.58) p=0.264;<br>• Estimated blood loss >1000 ml: Fetal pillow: 14/53 (26.42%) ; No-fetal pillow: 14/48 (29.17%); estimated RR (95% CI): 0.91 (0.48, 1.72) p=0.758<br>• Operative time (duration of surgery): Not reported<br>**Perinatal:**<br>• Infant birth trauma (skull fracture / intracranial haemorrhage / other bony fracture / nerve injury): Study reported 'neonatal trauma' - defined as any soft tissue injury or bony trauma as well as intracranial bleed as diagnosed by paediatric examination. Fetal pillow: 16/53 (30.19%); No fetal pillow: 11/48 (23.40%); estimated relative risk: 1.29 (0.67–2.59), p=0.449.<br>• Apgar score at five minutes / Apgar score < 7 at five minutes:<br>• Apgar score at 5 minutes: Not reported.<br>• Apgar score <7 at 5 minutes: Fetal pillow : 2/53 (3.77%); No-fetal pillow: 3/48 (6.25%); estimated relative risk (95% CI): 0.60 (0.08–3.50) p=0.571
**Secondary outcomes and results**	**Maternal:**<br>• Blood transfusion: Fetal pillow: 2/53 (3.77%); No-fetal pillow: 1/48 (2.08%); estimated relative risk (95% CI): 0.83 (0.40, 1.72), p= 0.612.<br>• Inverted T or J incision: Not reported<br>• Visceral injury (ureteral / bladder / cervical) or hysterectomy:<br>○ Uterine incision extension into cervix / vagina: Not reported<br>○ Injury to urinary tract (including ureteric injury and bladder injury): Not reported<br>○ Hysterectomy: Not reported<br>• Infection (wound infection / endometritis / maternal sepsis):<br>○ Wound infection: Not reported

	○ Endometritis: Not reported<br>○ Urinary tract infection: Not reported<br>○ Post-partum pyrexia / maternal sepsis: Not reported<br>• Duration of hospital stay: Reports 'length of post-operative stay': Fetal pillow (N=53): Mean (SD) = 3.22 (1.41) days ; No-fetal pillow (N=48): Mean (SD) = 3.45 (1.23) days; estimated relative risk (95% CI): −0.23 (−0.75, 0.29), p= 0.386<br>• Decision-to-delivery interval: Not reported<br>• Incision-to-delivery interval: Not reported<br>**Perinatal:**<br>• NICU (Neonatal Intensive Care Unit) admission: Reports 'admission to nursery' which includes special care baby unit and neonatal intensive care unit: Fetal pillow: 16/53 (30.19%); No-fetal pillow: 22/48 (46.81%); estimated relative risk (95% CI): 0.64 (0.38–1.07), p=0.092.<br>• Umbilical artery pH: Reports 'cord arterial pH'; Fetal pillow (N=53): Mean (SD) = 7.25 (0.06); No-fetal pillow (N=48): Mean (SD) = 7.24 (0.08): estimated relative risk (95% CI): 0.00 (−0.03–0.03), p=0.918.<br>• Neonatal death (defined as death within the first 28 days of life): Not reported<br>Cost: Not reported
**Risk of bias**	**Assessed by ROBINS-I:**<br>• Bias due to confounding: Serious risk of bias – no adjustment for confounding. The authors report: 'Due to the small sample size, particularly in relation to binary outcomes, adjustment for potential confounders was not possible'.<br>• Bias in selection of participants into study: Moderate risk of bias – obstetrician preference or situation-specific factors (such as urgency of delivery) may affect whether fetal pillow is used. Initial follow-up time appears to be similar for all women.<br>• Bias in classification of interventions: Moderate risk of bias – clear definition provided for fetal pillow group but not for the no-fetal pillow group.<br>• Bias due to deviations from intended intervention: Low risk of bias – no deviations from the intended intervention reported.<br>• Bias due to missing data: Low risk of bias – outcomes reported for all women.<br>• Bias on measurement of outcomes: Moderate risk of bias - unclear definition of some outcomes e.g. position of uterine incision extension not reported, criteria not reported.<br>• Bias in selection of the reported result: Moderate risk of bias - no mention of pre-registered protocol or statistical analysis plan.<br>Overall risk of bias: Serious risk of bias.
**Source of funding**	Not reported

7. Dutta 2019

**Study details**	**Full citation:** Dutta S., Bhattacharyya S.K., Adhikary S., Seal S.L. A comparative study between modified Patwardhan technique and Foetal Pillow during caesarean section in full dilatation in cases of deeply engaged foetal head, IOSR Journal of Dental and Medical Sciences (IOSR-JDMS), vol. 18, no. 9, 2019, pp 01-07<br>**Study type:** Randomised controlled trial<br>**Country of study:** Bengal<br>**Study dates**: May 2016 to April 2017
**Participants**	**Inclusion criteria:** Women with single pregnancy, a gestational age of ≥36 weeks and deeply engaged fetal head<br>**Exclusion criteria:** Women with active genital infections, fetal malpresentation or non-cephalic presentation<br>**Participant characteristics:**<br>- ‘Fetal pillow’ group:<br>o N women: 25<br>o Mean (SD) maternal age: 24.92 (3.74) years<br>o Mean (SD) maternal weight: 55.64 (7.04) kg<br>o Smoking status: Not reported<br>o Parity: 0: 16 (32%); 1: 6 (12%); 2: 3 (6%); 3: 0 (0%)<br>o Diabetes: Not reported<br>o Mean (SD) gestational age: 38.04 (0.93) weeks<br>o Numbers with caesarean section at full (10cm) cervical dilatation: Not explicitly stated, but presumably 25 as all women at full dilatation<br>o Numbers with caesarean section prior to (<10cm) full cervical dilatation: 0<br>- ‘Modified Patwardhan method’ group:<br>o N women: 25<br>o Mean (SD) maternal age: 25.04 (4.75) years<br>o Mean (SD) maternal weight: 56 (9.05) kg<br>o Smoking status: Not reported<br>o Parity: 0: 14 (28%); 1: 6 (12%); 2: 3 (6%); 3: 2 (4%)<br>o Diabetes: Not reported<br>o Mean (SD) gestational age: 38.96 (1.33) weeks<br>o Numbers with caesarean section at full (10cm) cervical dilatation: Not explicitly stated, but presumably 25 as all women at full dilatation<br>o Numbers with caesarean prior to (<10cm) full cervical dilatation: 0

**Intervention & comparator**	**Intervention:**'Fetal pillow': "The device is inserted per vaginally during the second stage of labour before Caesarean Section at Full Dilatation with balloon in contact with foetal head. To inflate the balloon, 180 cc of normal saline is pushed via the distal 2– way tap (with the help of 60 ml syringe); thereby disimpacting the foetal head from the maternal pelvis up to the level of incision. Once the baby is delivered, the balloon is deflated and the device is taken out Per Vaginally" (p. 2).<br>**Comparator:** 'Modified Patwardhan method': "In cases of occipito–transverse or occipito– anterior positions with the head deeply engaged in the pelvis, incision is made in the lower uterine segment, at the level of anterior shoulder, which is delivered out. This is followed by delivery of the posterior shoulder, axillae (by hooking method) and the trunk (by application of fundal pressure by the assistant). Finally, the foetal head, which is deeply impacted in pelvis and is still inside the uterus, is gently lifted out of the pelvis" (p. 2).
**Primary outcomes and results**	**Maternal:**<br>• Uterine incision extension (angle extensions on lower segments and / or into broad ligaments):<br>○ Incision extension on lower segment (at angles or towards cervix): Study reported as extension of incision (no further details): 'Fetal pillow': 2/25; 'Modified Patwardhan method': 6/25 (p=0.001)<br>○ Angle extensions into broad ligaments: Not reported<br>• Operative blood loss / post-partum haemorrhage (operative blood loss > 500ml): Not reported<br>• Operative time (duration of surgery): 30 to 40 minutes: 'Fetal pillow': 20/25; 'Modified Patwardhan method': 16/25; 40 to 50 minutes: 'Fetal pillow': 3/25; 'Modified Patwardhan method': 7/25; 50 to 60 minutes: 'Fetal pillow': 2/25; 'Modified Patwardhan method': 1/25; >1 hour: 'Fetal pillow': 0/25; 'Modified Patwardhan method': 1/25 (p=0.02)<br>**Perinatal:**<br>• Infant birth trauma (skull fracture / intracranial haemorrhage / other bony fracture / nerve injury): Not reported<br>• Apgar score at five minutes / Apgar score < 7 at five minutes: Not reported
**Secondary outcomes and results**	**Maternal:**<br>• Blood transfusion: 'Fetal pillow': 0/25; 'Modified Patwardhan method': 4/25 (p=0.002)<br>• Inverted T or J incision: Not reported<br>• Visceral injury (ureteral / bladder / cervical) or hysterectomy:<br>○ Uterine incision extension into cervix / vagina: Not reported<br>○ Injury to urinary tract (including ureteric injury and bladder injury): Not reported<br>○ Hysterectomy: Not reported<br>• Infection (wound infection / endometritis / maternal sepsis):<br>○ Wound infection: Not reported<br>○ Endometritis: Not reported<br>○ Urinary tract infection: Not reported<br>○ Post-partum pyrexia / maternal sepsis: Not reported<br>• Duration of hospital stay: Not reported<br>• Decision-to-delivery interval: Not reported

	• Incision-to-delivery interval: 0 to 2 minutes: 'Fetal pillow': 13/25; 'Modified Patwardhan method': 2/25; 2 to 4 minutes: 'Fetal pillow': 12/25; 'Modified Patwardhan method': 11/25; 4 to 6 minutes: 'Fetal pillow': 0/25; 'Modified Patwardhan method': 12/25 (p=0.04)<br>**Perinatal:**<br>• NICU (Neonatal Intensive Care Unit) admission: 'Fetal pillow': 3/25; 'Modified Patwardhan method': 15/25 (p=0.04)<br>• Umbilical artery pH / cord pH < 7.10: Not reported<br>• Neonatal death (defined as death within the first 28 days of life): This outcome was not fully reported, but the authors stated that one baby in the 'Modified Patwardhan method' expired on day 11 (early neonatal death).<br>Cost: Not reported
**Risk of bias**	**Assessed by RoB2:**<br>• Risk of bias arising from the randomisation process: Low risk of bias – women were randomly assigned to the study groups using computer-generated randomisation; treatment allocation was concealed in identical opaque, sealed, sequentially numbered envelopes in the operating room.<br>• Risk of bias due to deviations from the intended interventions (effect of assignment to intervention): Low risk of bias – no information provided relating to blinding to treatment assignment; no deviations from intended intervention.<br>• Risk of bias due to missing outcome data: Low risk of bias – no reported missing outcome data..<br>• Risk of bias in measurement of the outcome: Low risk of bias – outcomes measured using objective measurement tools; comparable outcome detection methods and thresholds used, and same definitions and measurements.<br>• Risk of bias in selection of the reported result: High risk of bias - the authors stated that data were collected in a pre-designed and pre-tested schedule, but no further details were provided and there is no protocol. Additionally, there is very little detail regarding the outcomes reported in the methods section. Importantly, the study was published in a potential predatory journal according to Beall's List of Potential Predatory Journals and Publishers.<br>Overall risk of bias: High risk of bias.
**Source of funding**	The authors reported that the cephalic elevation devices used in this study were donated by Safe Obstetrics Systems, a medical device company acquired in 2021 by CooperCompanies, a global medical device company.

8. Fasubaa 2002

**Study details**	**Full citation:** Fasubaa O.B., Ezechi O.C., Orji E.O., Ogunniyi S.O., Akindele S.T., Loto O.M., et al. Delivery of the impacted head of the fetus at caesarean section after prolonged obstructed labour: a randomised comparative study of two methods. Journal of Obstetrics & Gynaecology. 2002 Sep 10<br>**Study type:** Randomised controlled trial<br>**Country of study:** Nigeria<br>**Study dates:** June 1998 to May 2000
**Participants**	**Inclusion criteria:** Women with prolonged obstructed labour with a live fetus<br>**Exclusion criteria:** Women with multiple pregnancy; intrauterine fetal death; congenital fetal anomaly; ruptured uterus; previous caesarean section; fetal head more than 2 finger-breadths palpable per abdomen<br>**Participant characteristics:**<br>- Vaginal push up group:<br>o N women: 54<br>o Mean (SD; unclear if value is SD/SE or something else) maternal age: 23.3 (3.1) years<br>o Mean (SD) maternal weight: Not reported<br>o Smoking status: Not reported<br>o Mean (SD; unclear if value is SD/SE or something else) parity: 3.1 (0.4)<br>o Diabetes: Not reported<br>o Mean (SD; unclear if value is SD/SE or something else) gestational age: 38 (0.5) weeks<br>o Numbers with caesarean section at full (10cm) cervical dilatation: Not reported<br>o Numbers with caesarean prior to (<10cm) full cervical dilatation: Not reported<br>- Reverse breech extraction group:<br>o N women: 54<br>o Mean (SD; unclear if value is SD/SE or something else) maternal age: 22.4 (2.5) years<br>o Mean (SD) maternal weight: Not reported<br>o Smoking status: Not reported<br>o Mean (SD; unclear if value is SD/SE or something else) parity: 3.1 (0.3)<br>o Diabetes: Not reported<br>o Mean (SD; unclear if value is SD/SE or something else) gestational age: 38.1 (0.1) weeks<br>o Numbers with caesarean section at full (10cm) cervical dilatation: Not reported<br>o Numbers with caesarean section prior to (<10cm) full cervical dilatation: Not reported
**Intervention & comparator**	**Intervention:** 'Vaginal push up': "After opening into the uterus the patient is positioned in the supine position with the knees flexed and the lower legs abducted by two assistants. One of the assistants under sterile condition introduces his finger into the vagina and then pushes the head up to disimpact it. The surgeon then introduces his hand into the uterus between the fetal head and the uterine wall, moving his hand downwards to get beneath the fetal head as the

<table>
<tr><td></td><td>assistant disimpacts the fetal head from below. The patient's legs are then returned to normal position. The Surgeon then delivers the fetal head and the rest of the fetus as it is performed in routine caesarean section" (p. 376).

Comparator: 'Reverse breech extraction': "After opening into the uterus the surgeon introduces his hand towards the upper segment of the uterus and searches for a fetal leg. He then applies gentle traction until the second leg appears. With the two legs held together, he gently pulls the fetus up and out of the uterus through the uterine incision until delivered completely, as is performed for assisted vaginal breech delivery. Assistance is given to the delivery of the fetus at the shoulder and at the delivery of the following head" (p. 376).

"After delivery of the fetus, the same procedure was adopted in both methods to complete the operation. The uterine muscle was closed in two layers with number 2 chromic catgut and peritoneum with 2/0 chromic catgut. The anterior abdominal wall was closed in layers with non-absorbable number 1 nylon sutures to the rectus sheet and 3/0 nylon to the skin. All skin incisions were midline infra-umbilical incisions" (p. 376).

All women received rehydration, antibiotics, correction of electrolyte imbalance and anaemia if present, and urethral catheterisation for 10 days as prophylaxis against genital fistula, as per departmental protocol. Women without complications were discharged on the 11th post-operative day after removal of the urethral catheter on day 10.</td></tr>
<tr><td>**Primary outcomes and results**</td><td>**Maternal:**
• Uterine incision extension (angle extensions on lower segments and / or into broad ligaments):
  ○ Incision extension on lower segment (at angles or towards cervix): Not reported
  ○ Angle extensions into broad ligaments: Study reported uterine incision extension to ligaments: 'Vaginal push up': 16/54; 'Reverse breech extraction': 6/54;
• Operative blood loss / postpartum haemorrhage (operative blood loss > 500ml): Study reported operative blood loss (ml): 'Vaginal push up': Mean (SD) = 1256.5 (54.3) ml; 'Reverse breech extraction': Mean (SD) = 898.4 (35.2) ml (p<0.001)
• Operative time (duration of surgery): 'Vaginal push up': Mean (SD) = 88.5 (4.5) minutes; 'Reverse breech extraction': Mean (SD) = 55.6 (5.5) minutes (p<0.001)
Perinatal:
• Infant birth trauma (skull fracture / intracranial haemorrhage / other bony fracture / nerve injury): 'Vaginal push up': 3/54; 'Reverse breech extraction': 4/54
• Apgar score at five minutes: 'Vaginal push up': Mean (SD) = 7.8 (0.2); 'Reverse breech extraction': Mean (SD) = 9 (0.2) (p<0.001)</td></tr>
<tr><td>**Secondary outcomes and results**</td><td>**Maternal:**
• Blood transfusion: Not reported
• Inverted T or J incision: Not reported
• Visceral injury (ureteral / bladder / cervical) or hysterectomy:</td></tr>
</table>

	○ Uterine incision extension into cervix / vagina: Study reported uterine incision extension to vagina: ‘Vaginal push up’: 9/54; ‘Reverse breech extraction’: 2/54<br>○ Injury to urinary tract (including ureteric injury and bladder injury): Not reported<br>○ Hysterectomy: Not reported<br>• Infection (wound infection / endometritis / maternal sepsis):<br>○ Wound infection: ‘Vaginal push up’: 15/54; ‘Reverse breech extraction’: 18/54<br>○ Endometritis: ‘Vaginal push up’: 31/54; ‘Reverse breech extraction’: 19/54<br>○ Urinary tract infection: Not reported<br>○ Post-partum pyrexia / maternal sepsis: Not reported<br>• Duration of hospital stay: ‘Vaginal push up’: Mean (SD) = 14.5 (2.5) days; ‘Reverse breech extraction’: Mean (SD) = 11.4 (1.2) days (p<0.001)<br>• Decision-to-delivery interval: Not reported<br>• Incision-to-delivery interval: Not reported<br>**Perinatal:**<br>• NICU (Neonatal Intensive Care Unit) admission: Study reported neonatal admission rate: ‘Vaginal push up’: 39/54; ‘Reverse breech extraction’: 14/54<br>• Umbilical artery pH / cord pH < 7.10: Not reported<br>• Neonatal death (defined as death within the first 28 days of life): Study reported early neonatal death (not otherwise defined): ‘Vaginal push up’: 13/54; ‘Reverse breech extraction’: 7/54<br>Cost: Not reported
**Risk of bias**	**Assessed by RoB2:**<br>• Risk of bias arising from the randomisation process: Low risk of bias – women were randomly assigned to the study groups using computer-generated randomisation.<br>• Risk of bias due to deviations from the intended interventions (effect of assignment to intervention): Low risk of bias - operative blood loss estimated by the anaesthetist, who was blinded; no deviations from intended intervention.<br>• Risk of bias due to missing outcome data: Low risk of bias – no reported missing outcome data.<br>• Risk of bias in measurement of the outcome: Low risk of bias – outcomes measured using objective measurement tools; comparable outcome detection methods and thresholds used, and same definitions and measurements.<br>• Risk of bias in selection of the reported result: Some concerns - no details provided.<br>Overall risk of bias: Some concerns.
**Source of funding**	Not reported

9. Frass 2011

**Study details**	**Full citation:** Frass A.K., Eryani A.A, Al-Hardzi A.H. Reverse breech extraction versus head pushing in caesarean section for obstructed labor: A comparative study in Yemen<br>**Study type:** Randomised controlled trial<br>**Country of study:** Yemen<br>**Study dates**: January to December 2010
**Participants**	**Inclusion criteria:** Women with single, term pregnancy (dependent on last menstrual period or early first trimester ultrasonography), cephalic presentation, obstructed labour and requiring abdominal delivery<br>**Exclusion criteria:** Women with multiple pregnancy, non-cephalic presentation, previous scar, or pre-term labour<br>**Participant characteristics:**<br>- ‘Vaginal push up’ group:<br>o N women: 59<br>o Mean (SD) maternal age: 26.1 (5.9) years<br>o Mean (SD) maternal BMI: Not reported<br>o Smoking status: Not reported<br>o Mean (SD) parity: 1.89 (1.1)<br>o Diabetes: Not reported<br>o Mean (SD) gestational age: 38.61 (1.1)<br>o Numbers with caesarean section at full (10cm) cervical dilatation: Not reported<br>o Numbers with caesarean section prior to (<10cm) full cervical dilatation: Not reported<br>- ‘Reverse breech extraction’ group:<br>o N women: 59<br>o Mean (SD) maternal age: 25.6 (5.7) years<br>o Mean (SD) maternal BMI: Not reported<br>o Smoking status: Not reported<br>o Mean (SD) parity: 1.93 (1.2)<br>o Diabetes: Not reported<br>o Mean (SD) gestational age: 38.49 (1)<br>o Numbers with caesarean section at full (10cm) cervical dilatation: Not reported<br>o Numbers with caesarean prior to (<10cm) full cervical dilatation: Not reported
**Intervention & comparator**	**Intervention:** ‘Vaginal push up’: “The surgeon inserted the right hand into the uterus down to dislodge the fetal head from pelvis and when difficulty was encountered, the assistant inserted the right hand through the vagina applying pressure to the fetal head upwards until it can be easily grasped by the surgeon’s “push” method, the surgeon then delivers the fetus manually without using instruments” (p. 1262).

	**Comparator:** 'Reverse breech extraction': "The surgeon's right hand was inserted upwards into the upper segment to find and grasp a foot and deliver it along with leg through the incision. Traction on that foot brought the contra lateral lower limb into the operative field. The surgeon then grasped both feet and proceed in a manner similar to that practiced in breech extraction" (p. 1262).<br><br>"Preoperative preparations and anesthetic technique administered were similar to all patients. The standard cesarean section techniques were followed in both groups. The uterus was opened with a transverse incision in the lower segment…. The standard postoperative care protocol is mostly similar for each woman including antibiotics for the first 3 days, prophylactic anti-coagulant, early ambulation, and analgesics, which are given depending on the "on need principle". Liquid are allowed 24 hours later and gradual solid diet is allowed 2-3 days postoperation. The indwelling Foley's catheter is kept in place for 7-10 days in some selected cases who were complicated by prolonged obstructed labor, or significant extension of the uterine incision" (p. 1262).
**Primary outcomes and results**	**Maternal:**<br>• Uterine incision extension (angle extensions on lower segments and / or into broad ligaments):<br>○ Incision extension on lower segment (at angles or towards cervix): Study reported inadvertent extension of uterine incision beyond normal limits: 'Vaginal push up': 24/59; 'Reverse breech extraction': 3/59; (95% CI: 0.02 to 0.28; p=0.0001)<br>○ Angle extensions into broad ligaments: Not reported<br>• Operative blood loss / post-partum haemorrhage (operative blood loss > 500ml): Study reported operative blood loss: 'Vaginal push up': Mean (SD) = 1231 (471) ml; 'Reverse breech extraction': Mean (SD) = 787 (519) ml; (95% CI: 263.28 to 624.72; p<0.0001); Post-partum haemorrhage: 'Vaginal push up': 10/59; 'Reverse breech extraction': 5/59; (p=NS)<br>• Operative time (duration of surgery): 'Vaginal push up': Mean (SD) = 67.2 (4.7) minutes; 'Reverse breech extraction': Mean (SD) = 52.9 (5.1) minutes; (95% CI: 12.51 to 16.09; p<0.0001)<br>**Perinatal:**<br>• Infant birth trauma (skull fracture / intracranial haemorrhage / other bony fracture / nerve injury): Not reported<br>• Apgar score at five minutes / Apgar score < 7 at five minutes: Study reported Apgar score <7 at 5 minutes: 'Vaginal push up': 14/59; 'Reverse breech extraction': 12/59; (p=NS)
**Secondary outcomes and results**	**Maternal:**<br>• Blood transfusion: 'Vaginal push up': 6/59; 'Reverse breech extraction': 4/59; (p=NS)<br>• Inverted T or J incision: This outcome was not fully reported, but the authors also stated that "there was difficult extraction of the fetal legs due to severe firmly contracted uterine muscles over the fetus in 4 women and the inverted T-incision was needed" (p. 1263): 'Vaginal push up': 0/59; 'Reverse breech extraction': 4/59<br>• Visceral injury (ureteral / bladder / cervical) or hysterectomy:<br>○ Uterine incision extension into cervix / vagina: Not reported

	○ Injury to urinary tract (including ureteric injury and bladder injury): This outcome was not fully reported, but the authors stated that “no ureteral or bladder involvement was observed” (p. 1263): ‘Vaginal push up’: 0/59; ‘Reverse breech extraction’: 0/59<br>○ Hysterectomy: Not reported<br>• Infection (wound infection / endometritis / maternal sepsis):<br>○ Wound infection: ‘Vaginal push up’: 4/59; ‘Reverse breech extraction’: 3/59; (p=NS)<br>○ Endometritis: ‘Vaginal push up’: 8/59; ‘Reverse breech extraction’: 7/59; (p=NS)<br>○ Urinary tract infection: Not reported<br>○ Post-partum pyrexia / maternal sepsis: Not reported<br>• Duration of hospital stay: ‘Vaginal push up’: Mean (SD) = 6.6 (1.2) days; ‘Reverse breech extraction’: Mean (SD) = 6.1 (1.7) days; (95% CI -0.04 to 1.04; p=0.07)<br>• Decision-to-delivery interval: Not reported<br>• Incision-to-delivery interval: Not reported<br>**Perinatal:**<br>• NICU (Neonatal Intensive Care Unit) admission: Study reported admission to nursery (special care nursery): ‘Vaginal push up’: 13/59; (95% CI: 12.51 to 16.09; ‘Reverse breech extraction’: 11/59; (p=NS)<br>• Umbilical artery pH / cord pH < 7.10: Not reported<br>• Neonatal death (defined as death within the first 28 days of life): Not reported<br>Cost: Not reported
**Risk of bias**	**Assessed by RoB2:**<br>• Risk of bias arising from the randomisation process: Some concerns – 'Distribution of women to either group was made randomly based on 1:1 ratio', no other details were provided.<br>• Risk of bias due to deviations from the intended interventions (effect of assignment to intervention): Low risk of bias – no information provided relating to blinding to treatment assignment; no deviations from intended intervention.<br>• Risk of bias due to missing outcome data: Low risk of bias – no reported missing outcome data..<br>• Risk of bias in measurement of the outcome: Low risk of bias – outcomes measured using objective measurement tools; comparable outcome detection methods and thresholds used, and same definitions and measurements.<br>• Risk of bias in selection of the reported result: Some concerns - no details provided.<br>Overall risk of bias: Some concerns.
**Source of funding**	None

10. Hanley 2020

**Study details**	**Full citation:** Hanley I., Sivanesan K., Veerasingham M., Vasudevan J. Comparison of outcomes at full-dilation cesarean section with and without the use of a fetal pillow device. Int J Gynecol Obstet 2020;150:228–33

	**Study type:** Non-randomised comparative retrospective cohort study<br>**Country of study:** Australia<br>**Study dates**: January 2014 to October or December 2018 (main text states the former whereas the abstract states the latter)
**Participants**	**Inclusion criteria:** Women undergoing caesarean section at full dilation or had use of a fetal pillow<br>**Exclusion criteria:** Women at full dilation with breech presentation or women at less than full dilation<br>**Participant characteristics:**<br>- Fetal pillow group:<br>o N women: 114<br>o Mean (SD) maternal age: 28.04 (5.33) years<br>o Mean (SD) maternal BMI: (N=113) 28.91 (7.76) kg/m^2<br>o Smoking status: 14/114<br>o Mean (SD) parity: 0.41 (1.12)<br>o Diabetes: 15/114<br>o Gestational age: Not reported<br>o Numbers with caesarean section at full (10cm) cervical dilatation: Not explicitly stated, but presumably 114 as per the inclusion criteria<br>o Numbers with caesarean section prior to (<10cm) full cervical dilatation: Not explicitly state, but presumably 0 as per the inclusion criteria<br>- No fetal pillow group:<br>o N women: 60<br>o Mean (SD) maternal age: 26.6 (5.75) years<br>o Mean (SD) maternal BMI: 28.61 (6.58) kg/m^2<br>o Smoking status: 9/60<br>o Mean (SD) parity: 0.47 (0.96)<br>o Diabetes: 10/60<br>o Gestational age: Not reported<br>o Numbers with caesarean section at full (10cm) cervical dilatation: Not explicitly stated, but presumably 60 as per the inclusion criteria<br>o Numbers with caesarean prior to (<10cm) full cervical dilatation: Not explicitly state, but presumably 0 as per the inclusion criteria
**Intervention & comparator**	**Intervention:** Fetal pillow: "Before commencing a cesarean delivery, the uninflated pillow device is positioned into the vagina. It is then filled with sterile saline with the goal of elevating the fetal head to assist with disimpaction when attempting to deliver it through a lower abdominal incision" (p. 229).<br>**Comparator:** No fetal pillow<br><br>"All patients received intravenous antibiotics (Cephazolin or Lincomycin) before skin incision and all patients received both mechanical and chemical deep vein thrombosis prophylaxis (heparin or enoxaparin) after casesarean" (p. 229).

**Primary outcomes and results**	**Maternal:**<br>• Uterine incision extension (angle extensions on lower segments and / or into broad ligaments):<br>○ Incision extension on lower segment (at angles or towards cervix): Fetal pillow: 13/114; No fetal pillow: 11/60<br>○ Angle extensions into broad ligaments: Not reported<br>• Operative blood loss / post-partum haemorrhage (operative blood loss >500ml): Study reported estimated blood loss (ml): Fetal pillow (N=113): Mean (SD) = 730 (400) ml; No-fetal pillow (N=59): Mean (SD) = 725 (374) ml; adjusted coefficient (95% CI): 1.76 (-123.75 to 127.27; p=0.98); estimated blood loss >1000 ml: Fetal pillow: 15/114; No-fetal pillow: 7/60; adjusted OR (95% CI): 1.15 (0.44 to 3; p=0.77)<br>• Operative time (duration of surgery): Not reported<br>**Perinatal:**<br>• Infant birth trauma (skull fracture / intracranial haemorrhage / other bony fracture / nerve injury): Not reported<br>• Apgar score at five minutes / Apgar score < 7 at five minutes: Mean (SD) Apgar score at 5 minutes: Fetal pillow (N=113): Mean (SD) = 8.65 (1.08); No-fetal pillow (N=60): Mean (SD) = 8.63 (1.02); adjusted coefficient (95% CI): 0.27 (-0.31 to 0.36; p=0.87): Apgar score < 7 at five minutes: Fetal pillow: 105/113; No-fetal pillow: 56/60; adjusted OR (95% CI): 1.07 (0.31 to 3.37; p=0.91)
**Secondary outcomes and results**	**Maternal:**<br>• Blood transfusion: Fetal pillow: 1/114; No-fetal pillow: 3/58; adjusted OR (95% CI): 0.22 (0.03 to 1.64; p=0.14)<br>• Inverted T or J incision: Study reported addition of vertical component to hysterotomy: Fetal pillow: 3/114; No-fetal pillow: 2/60<br>• Visceral injury (ureteral / bladder / cervical) or hysterectomy:<br>○ Uterine incision extension into cervix / vagina: Study reported vaginal trauma: Fetal pillow: 0/114; No-fetal pillow: 0/60<br>○ Injury to urinary tract (including ureteric injury and bladder injury): Study reported cystotomy: Fetal pillow: 2/114; No-fetal pillow: 2/60<br>○ Hysterectomy: Not reported<br>• Infection (wound infection / endometritis / maternal sepsis):<br>○ Wound infection: Not reported<br>○ Endometritis: Not reported<br>○ Urinary tract infection: Not reported<br>○ Post-partum pyrexia / maternal sepsis: Not reported<br>• Duration of hospital stay: Fetal pillow (N=114): Mean (SD) = 65.75 (24.27) hours; No-fetal pillow (N=59): Mean (SD) = 72.56 (28.21) hours; adjusted coefficient (95% CI): -7.5 (-15.65 to 0.64; p=0.71)<br>• Decision-to-delivery interval: Not reported<br>• Incision-to-delivery interval: Not reported<br>**Perinatal:**

	• NICU (Neonatal Intensive Care Unit) admission: Fetal pillow: 37/112; No-fetal pillow: 24/60; adjusted OR (95% CI): 0.76 (0.39 to 1.47; p=0.41)<br>• Umbilical artery pH: Fetal pillow (N=98): Mean (SD) = 7.25 (0.08); No-fetal pillow (N=49): Mean (SD) = 7.19 (0.10); adjusted coefficient (95% CI): 0.06 (0.03 to 0.09; p=0.0001)<br>• Neonatal death (defined as death within the first 28 days of life): Not reported<br>Cost: Not reported
**Risk of bias**	**Assessed by ROBINS-I:**<br>• Bias due to confounding: Serious risk of bias - more women in the 'No fetal pillow' intervention arm had an attempted instrumental delivery before they were taken for caesarean section, than women in the 'Fetal pillow' arm (although this was not statistically significant); there were statistically significantly more women in the 'No fetal pillow' intervention arm who had use of 'hand push' at caesarean section; the authors used logistic regression models (including the fetal pillow, age, and BMI variables) to adjust for confounding; data were not adjusted for time trends.<br>• Bias in selection of participants into study: Moderate risk of bias –the authors stated that the significant finding of improved arterial pH with the use of the fetal pillow may potentially be explained by selection bias; the authors acknowledged that Obstetrician preference or situation-specific factors (such as urgency of delivery) affects whether fetal pillow is used and this leads to selection bias which cannot be adjusted for; initial follow-up time appears to be similar for all women.<br>• Bias in classification of interventions: Moderate risk of bias – clear definition provided for fetal pillow group but not for the no-fetal pillow group.<br>• Bias due to deviations from intended intervention: Low risk of bias – no deviations from the intended intervention reported.<br>• Bias due to missing data: Serious risk of bias – incomplete health records were removed and excluded if data were missing from the final vaginal examination or information from the operating theatre (Fetal pillow: N=25 (15%); No-fetal pillow: N=18 (22%)); some outcomes did not have data reported for all included participants.<br>• Bias on measurement of outcomes: Low risk of bias - outcomes measured using objective measurement tools; comparable outcome detection methods and thresholds used, and same definitions and measurements.<br>• Bias in selection of the reported result: Moderate risk of bias - no mention of pre-registered protocol or statistical analysis plan.<br>Overall risk of bias: Serious risk of bias.
**Source of funding**	Not reported

11. Javed 2022

**Study details**	**Full citation:** Javed A., Noreen H., Batool I., Arshad N.<br>Comparison of push and pull methods of delivery for deeply engaged fetal head during cesarean section for prolong second stage of labor in preventing extension of uterine incision. Rawal Medical Journal: 47 (1), Jan-Mar 2022<br>**Study type:** Randomised controlled trial<br>**Country of study:** Pakistan<br>**Study dates**: December 2018 to June 2019
**Participants**	**Inclusion criteria:** Women with singleton pregnancy with prolonged second stage of labor, gestational age ≥ 37 weeks (assessed on LMP), parity status 1-5, BMI ≤ 30 kg/m2 and age 18 to 40 years.<br>**Exclusion criteria:** Patients with multiple pregnancy, non-cephalic presentation and previous uterine scar were excluded from the study<br>**Participant characteristics:**<br>- Vaginal push up group (Group B):<br>○ N women: 43<br>○ Mean (SD) maternal age: 28.21 (+/- 3.85 (not reported if SD, SE etc.)<br>○ Mean (SD) maternal BMI: 23.08 ± 3.66 kg/m2 (across both groups); in vaginal push-up group, n=31 had BMI </= 25 kg/m^2 and n=12 had BMI > 25 kg/m^2.<br>○ Smoking status: Not reported<br>○ Parity: Mean parity 2.55 ± 0.84 (across both groups); in vaginal push-up group, n=38 had parity 1-2 and n=5 had parity 3-5.<br>○ Diabetes: Not reported<br>○ Mean (SD) gestational age: 38.65 (1.04) weeks<br>○ Numbers with caesarean section at full (10cm) cervical dilatation: Not explicitly stated, but presumably 43 as women in second stage of labour<br>○ Numbers with caesarean prior to (<10cm) full cervical dilatation: Not explicitly stated, but presumably 0<br>- Reverse breech extraction (pull) group (Group A):<br>○ N women: 43<br>○ Mean (SD) maternal age: 28.4 (+/- 3.22) years<br>○ Mean (SD) maternal BMI: 23.08 ± 3.66 kg/m2 (across both groups); in reverse breech extraction group, n=34 had BMI </= 25 kg/m^2 and n=9 had BMI > 25 kg/m^2.<br>○ Smoking status: Not reported<br>○ Parity: Mean parity 2.55 ± 0.84 (across both groups); in reverse breech extraction group, n=38 had parity 1-2 and n=5 had parity 3-5.<br>○ Diabetes: Not reported<br>○ Mean (SD) gestational age: 38.21 (2.0) weeks

Commented [SK1]: Dilatation not stated but does state that women are in second stage of labour, so have used the same wording as previous studies where this is the case.

Commented [JvdS2R1]: TY - will verify when I do my 2nd reviewer check

	○ Numbers with caesarean section at full (10cm) cervical dilatation: Not explicitly stated, but presumably 43 as women in second stage of labour<br>○ Numbers with caesarean prior to (<10cm) full cervical dilatation: Not explicitly stated, but presumably 0
**Intervention & comparator**	**Intervention:** Vaginal push up: 'in push method, fetal head was pushed upward from vagina by assistant and baby delivered as cephalic by surgeon. It is described as abdomino-vaginal delivery in which patient legs were abducted by using a modified lithotomy position and a cupped hand gently pushed up fetal head through vagina and delivered through the uterine incision.'<br>**Comparator:** Reverse breech extraction: 'In pull method, also known as reverse breech extraction, the obstetrician introduced a hand through the uterine incision towards the upper segment to grasp both feet as baby is in cephalic position and gently delivers the fetus up as breech.'
**Primary outcomes and results**	**Maternal:**<br>• Uterine incision extension (angle extensions on lower segments and / or into broad ligaments): Study reported: 'Extension of uterine incision was assessed subjectively by surgeon as presence or absence of inadvertent extension of uterine incision beyond 1-2cm from normal lower segment uterine incision or tear/laceration more than 2cm in lower uterine segment.'<br>○ Incision extension on lower segment: Vaginal push up (push): 19/43; Reverse breech extraction (pull): 5/43 (p=0.001)<br>○ Angle extensions into broad ligaments: not reported<br>• Operative blood loss / post-partum haemorrhage (operative blood loss >1000 ml): Study reported operative blood loss (ml): 'Vaginal push up' (push): Mean (SD) = 515 (97.2) ml (*not stated, seems to be SD*); Reverse breech extraction (pull): Mean (SD) = 438 (72.4) ml (p=0.0001); Post-partum haemorrhage: Not reported<br>• Operative time (duration of surgery): 'Vaginal push up' (push): Mean (SD) = 46.37(7.44) (*not stated, seems to be SD*); minutes; Reverse breech extraction (pull): Mean (SD) = 36.54 (8.56) ml (p=0.0001)<br>• Infant birth trauma (skull fracture / intracranial haemorrhage / other bony fracture / nerve injury): The study reported 'fetal injury' without specifying the type of injury. 'Vaginal push up' (push): 0/43; Reverse breech extraction (pull) : 1/43.<br>• Apgar score at five minutes / Apgar score < 7 at five minutes: Study reports 'Apgar score' but does not state the time period): Mean (SD) Apgar score: Vaginal push up' (push): (N=43): Mean (SD) = 9.24 (0.78) (*not stated, seems to be SD*); Reverse breech extraction (pull) (N=43): Mean (SD) = 9.08 (0.76); Apgar score < 7 at five minutes: Not reported.
**Secondary outcomes and results**	**Maternal:**<br>• Blood transfusion: Not reported<br>• Inverted T or J incision: Not reported<br>○ Visceral injury (ureteral / bladder / cervical) or hysterectomy: Not reported<br>○ Injury to urinary tract (including ureteric injury and bladder injury): The study reported 'bladder injury' only: 'Vaginal push up' (push): 0/43; Reverse breech extraction (pull) : 0/43.

Commented [SK3]: Dilatation not stated but does state that women are in second stage of labour, so have used the same wording as previous studies where this is the case.

Commented [JvdS4R3]: TY - will verify when I do my 2nd reviewer check

	○ Hysterectomy: Not reported<br>• Infection (wound infection / endometritis / maternal sepsis):<br>○ Wound infection: Study reported ‘wound complication’. ‘Vaginal push up’ (push): 2/43; Reverse breech extraction (pull) : 1/43, (p=0.544)<br>○ Endometritis: Not reported<br>○ Urinary tract infection: Not reported<br>○ Post-partum pyrexia / maternal sepsis: Study reported ‘post-partum fever’. ‘Vaginal push up’ (push): 5/43; Reverse breech extraction (pull): 3/43, (p=0.132)<br>• Duration of hospital stay: Not reported<br>• Decision-to-delivery interval: Not reported<br>• Incision-to-delivery interval: Not reported<br>**Perinatal:**<br>• NICU (Neonatal Intensive Care Unit) admission: ‘Vaginal push up’ (push): 0/43; Reverse breech extraction (pull): 1/43.<br>• Umbilical artery pH / cord pH < 7.10: Not reported<br>• Neonatal death (defined as death within the first 28 days of life): Not reported<br>• Cost: Not reported
**Risk of bias**	**Assessed by RoB2:**<br>• Risk of bias arising from the randomisation process: Low risk of bias – method of randomisation and allocation concealment clearly described.<br>• Risk of bias due to deviations from the intended interventions (effect of assignment to intervention): Low risk of bias – no deviations from intended intervention.<br>• Risk of bias due to missing outcome data: Low risk of bias – no reported missing outcome data.<br>• Risk of bias in measurement of the outcome: Low risk of bias – outcomes measured using objective measurement tools; comparable outcome detection methods and thresholds used, and same definitions and measurements.<br>• Risk of bias in selection of the reported result: Some concerns - no details provided.<br>• Overall risk of bias: Some concerns.
**Source of funding**	Not reported

12. Keepanasseril 2019

**Study details**	**Full citation:** Keepanasseril A., Shaik N., Kubera N.S., Adhisivam B., Maurya D.K. Comparison of' push method' with' Patwardhan's method' on maternal and perinatal outcomes in women undergoing caesarean section in second stage. J Obstet Gynaecol. 2019; 39: 606-611<br>**Study type:** Non-randomised comparative retrospective cohort study<br>**Country of study:** South India<br>**Study dates**: January 2014 to June 2016
**Participants**	**Inclusion criteria:** Pregnant women who were at full dilation with the fetal head at or below the ischial spines, and who underwent caesarean section where delivery of the fetal head was undertaken using the Push or Patwardhan's method<br>**Exclusion criteria:** Women with multiple pregnancies, women with non-vertex presentation, and delivery by the 'Pull method'<br>**Participant characteristics:**<br>- Vaginal push up group:<br>o N women: 221<br>o Mean (SD) maternal age: 25.7 (3.9) years<br>o Mean (SD) maternal BMI: 26.8 (4.2) kg/m^2<br>o Smoking status: Not reported<br>o Parity: N=157 (71.0%) were nulliparous, no further details reported<br>o Diabetes: Not reported<br>o Mean (SD) gestational age: 39.5 (1.8) weeks<br>o Numbers with caesarean section at full (10cm) cervical dilatation: 221 (100%) as per the inclusion criteria<br>o Numbers with caesarean section prior to (<10cm) full cervical dilatation: 0 as per the inclusion criteria<br>- Patwardhan method group:<br>o N women: 77<br>o Mean (SD) maternal age: 25.0 (4.0) years<br>o Mean (SD) maternal BMI: 26.0 (3.6) kg/m^2<br>o Smoking status: Not reported<br>o Parity: N=57 (74.0%) were nulliparous, no further details reported<br>o Diabetes: Not reported<br>o Mean (SD) gestational age: 39.4 (1.3) weeks<br>o Numbers with caesarean section at full (10cm) cervical dilatation: 77 (100%) as per the inclusion criteria<br>o Numbers with caesarean prior to (<10cm) full cervical dilatation: 0 as per the inclusion criteria
**Intervention & comparator**	**Intervention:** 'Vaginal push up': "In the 'Push method', an assistant pushes the foetal head cephalad from below resulting in dis-impaction, and a further delivery is accomplished similar to the traditional cephalad delivery, as in a routine caesarean section" (p. 607).

	**Comparator:** 'Patwardhan method': "In the 'Patwardhan's method', uterine incision is placed at the level of anterior shoulder in a deeply impacted head, which is then delivered. Applying gentle traction on this shoulder, the other shoulder is also delivered. With the help of an assistant applying fundal pressure, the operator, by hooking and giving gentle traction to both axillae, delivers the body of the foetus. This is followed by the delivery of the feet, which is delivered by the combined action of traction and application of fundal pressure. The head is then delivered by traction on the legs" (p. 607 to 608).
**Primary outcomes and results**	**Maternal:**<br>• Uterine incision extension (angle extensions on lower segments and / or into broad ligaments): Study reported extension of uterine incision involving the lower segment, uterine vessels or broad ligament: 'Vaginal push up': 55/221; 'Patwardhan method': 20/77 (p=0.850)<br>○ Incision extension on lower segment (at angles or towards cervix): 'Vaginal push up': 52/221 (Extensions to the lower uterine flap: 42/221; extensions to the uterine artery: 10/221); 'Patwardhan method': 14/77 (Extensions to the lower uterine flap: 9/77; extensions to the uterine artery: 5/77)<br>○ Angle extensions into broad ligaments: 'Vaginal push up': 6/221; 'Patwardhan method': 6/77<br>• Operative blood loss / post-partum haemorrhage (operative blood loss >1000 ml): Study reported operative blood loss >1000 ml: 'Vaginal push up': Mean (SD) = 647.1 (250.3) mL; 'Patwardhan method': Mean (SD) = 653.2 (285.08) mL (p=0.857); Post-partum haemorrhage: 'Vaginal push up': 26/221; 'Patwardhan method': 8/77 (p=0.744)<br>• Operative time (duration of surgery): 'Vaginal push up': Mean (SD) = 69.0 (22.3) minutes; 'Patwardhan method': Mean (SD) = 67.3 (23.7) minutes (p=0.583)<br>**Perinatal:**<br>• Infant birth trauma (skull fracture / intracranial haemorrhage / other bony fracture / nerve injury): The study reported this outcome as "injuries to the visceral organ injury or the fracture of long bones or the skull during the attempt to deliver the baby" (p. 608) 'Vaginal push up': 8/221; 'Patwardhan method': 4/77 Birth injuries included: subgaleal haemorrhage ('Vaginal push up': 2/221; 'Patwardhan method': 1/77); laceration over the forehead/scalp ('Vaginal push up': 6/221; 'Patwardhan method': 1/77); fracture of a rib and humerus ('Vaginal push up': 0/221; 'Patwardhan method': 2/77).<br>• Apgar score at five minutes / Apgar score < 7 at five minutes: Not reported
**Secondary outcomes and results**	**Maternal:**<br>• Blood transfusion: 'Vaginal push up': 13/221; 'Patwardhan method': 7/77 (p=0.333)<br>• Inverted T or J incision: Not reported<br>• Visceral injury (ureteral / bladder / cervical) or hysterectomy:<br>○ Uterine incision extension into cervix / vagina: The study reported extensions into the vagina: 'Vaginal push up': 6/221; 'Patwardhan method': 3/77<br>○ Injury to urinary tract (including ureteric injury and bladder injury): This outcome is not fully reported, but intraoperative bowel or bladder injury is: 'Vaginal push up': 4/221; 'Patwardhan method': 0/77 (p=0.235)<br>○ Hysterectomy: Not reported

	• Infection (wound infection / endometritis / maternal sepsis):<br>○ Wound infection: Study reported this outcome as surgical site infection (the most common type of sepsis [defined as a fever >100.4 degrees Fahrenheit, which occurred on 2 separate occasions 24 hours after delivery, with bacteraemia resulting in systemic infection or surgical site infection]): ‘Vaginal push up’: 14/221; ‘Patwardhan method’: 12/77 (p=0.012)<br>○ Endometritis: Not reported<br>○ Urinary tract infection: Not reported<br>○ Post-partum pyrexia / maternal sepsis: Not reported<br>• Duration of hospital stay: ‘Vaginal push up’: Median (range) = 7 (3 to 45) days; ‘Patwardhan method’: Median (range) = 8 (3 to 37) days (p=0.233)<br>• Decision-to-delivery interval: Not reported<br>• Incision-to-delivery interval: Not reported<br>**Perinatal:**<br>• NICU (Neonatal Intensive Care Unit) admission: ‘Vaginal push up’: 80/221; ‘Patwardhan method’: 45/77 (p<0.001)<br>• Umbilical artery pH / cord pH < 7.10: Not reported<br>• Neonatal death (defined as death within the first 28 days of life): Study reported death within 7 days of birth: ‘Vaginal push up’: 3/221; ‘Patwardhan method’: 3/77 (p=0.172)<br>Cost: Not reported
**Risk of bias**	**Assessed by ROBINS-I:**<br>• Bias due to confounding: Serious risk of bias - more women in the ‘Vaginal push up’ intervention arm had an attempted instrumental delivery before they were taken for caesarean section, than women in the ‘Patwardhan method’ arm; the authors did not adjust for confounding in any of the analyses.<br>• Bias in selection of participants into study: Low risk of bias – selection of participants does not appear to be related to both intervention and outcomes; initial follow-up time appears to be similar for all women.<br>• Bias in classification of interventions: Low risk of bias – clear definitions provided for ‘Vaginal push up’ and ‘Patwardhan method’.<br>• Bias due to deviations from intended intervention: Low risk of bias – no deviations from the intended intervention reported.<br>• Bias due to missing data: Low risk of bias – outcomes reported for all women.<br>• Bias on measurement of outcomes: Low risk of bias - outcomes measured using objective measurement tools; comparable outcome detection methods and thresholds used, and same definitions and measurements.<br>• Bias in selection of the reported result: Moderate risk of bias - no mention of pre-registered protocol or statistical analysis plan.<br>• Overall risk of bias: Serious risk of bias.
**Source of funding**	Not reported

13. Lal 2018

Study details	**Full citation:** Lal, M., Goyal, P. and Shamim, S., 2018. Evaluation of Patwardhan technique in second stage Caesarean Section. Int Arch BioMed Clin Res. 2018;4(1):47-49.<br>**Study type:** Non-randomised comparative retrospective cohort study<br>**Country of study:** India<br>**Study dates**: 2014 to Dec 2016.
**Participants**	**Inclusion criteria:** Second stage caesarean section, women with single fetus, with anterior vertex, at term with a deeply impacted fetal head into pelvis, in whom decision of caesarean section was already taken.<br>**Exclusion criteria:** Intrauterine fetal death, congenital fetal anomaly, multiple pregnancy, preterm caesarean section, previous caesarean section.<br><br>**Participant characteristics:**<br>- Vaginal push up group (Group B):<br>o N women: 64<br>o Mean (SD) maternal age: Not reported<br>o Mean (SD) maternal BMI: Not reported<br>o Smoking status: Not reported<br>o Parity: Not reported<br>o Diabetes: Not reported<br>o Mean (SD) gestational age: 39.14 (1.0) weeks<br>o Numbers with caesarean section at full (10cm) cervical dilatation: Not explicitly stated, but presumably 56 as women in second stage of labour (introduction focuses on women at full dilatation).<br>o Numbers with caesarean section prior to (<10cm) full cervical dilatation: Not explicitly stated, but presumably 0<br>- Patwardhan method group (Group A):<br>o N women: 56<br>o Mean (SD) maternal age: Not reported<br>o Mean (SD) maternal BMI: Not reported<br>o Smoking status: Not reported<br>o Parity: Not reported<br>o Diabetes: Not reported<br>o Mean (SD) gestational age: 38.85 (0.85) weeks<br>o Numbers with caesarean section at full (10cm) cervical dilatation: Not explicitly stated, but presumably 56 as women in second stage of labour (introduction focuses on women at full dilatation)<br>o Numbers with caesarean prior to (<10cm) full cervical dilatation: Not explicitly stated, but presumably 0

**Intervention & comparator**	**Intervention:** “Patwardhan method” or “modified Patwardhan method”: *Patwardhan method:* “In cases of occipito-anterior and transverse positions, with the head deeply impacted in the pelvis, incision is made in the lower uterine segment, shoulders are present usually at incision level. The anterior shoulder is delivered out by hooking the arm first. With gentle traction on this shoulder, the posterior shoulder is also delivered out. Next the surgeon holds the trunk of the baby gently with both thumbs parallel to spine and with fundal pressure given by the assistant the buttocks are delivered followed by legs.” *Modified Patwardhan method:* “In case of occipito–posteior position with the head deeply into the pelvis, the anterior shoulder is delivered of first followed by delivering the same side leg § In other side leg is then delivered gently followed by same side arm. By gently pulling baby legs buttocks and the trunk of baby and are delivered. Lastly the baby head is delivered.”<br><br>**Comparator:** “Vaginal push-up” (described as ‘conventional push method’): “After opening the uterus, the wedged fetal head is pushed up by an assistant′s hand introduced through vagina. The surgeon then introduces his hand into the uterus, between the baby′s head & uterine wall, to get beneath the fetal head as the assistant pushes the fetal head up from down below. The surgeon then delivers the fetal head and rest of the body as in routine caesarean sections.”
**Primary outcomes and results**	**Maternal:**<br>• Uterine incision extension (angle extensions on lower segments and / or into broad ligaments): Study appears to report data for ‘uterine incision extension’ and ‘uterine artery injury’ together: Vaginal push up’ (Group B): 15/64; ‘Patwardhan method’ (Group A): 2/56 (p=0.0049).<br>o Incision extension on lower segment (at angles or towards cervix): Not reported separately<br>o Angle extensions into broad ligaments: Not reported separately<br>• Operative blood loss / post-partum haemorrhage (operative blood loss >1000 ml): Study reported ‘traumatic PPH’ and ‘atonic PPH’ without any definitions:’ traumatic PPH’: Vaginal push up’ (Group B): 16/64; ‘Patwardhan method’ (Group A): 2/56 (p=0.0049). ‘Atonic PPH’: Vaginal push up’ (Group B): 17/64; ‘Patwardhan method’ (Group A): 3/56 (p=0.4423).<br>• Operative time (duration of surgery): Not reported<br>**Perinatal:**<br>• Infant birth trauma (skull fracture / intracranial haemorrhage / other bony fracture / nerve injury: The study reported ‘fetal injury’ without specifying the type of injury. ‘Vaginal push up’ (Group B): 0/64; ‘Patwardhan method’ (Group A) 2/56 (p=0.4967).<br>• Apgar score at five minutes / Apgar score < 7 at five minutes: Not reported (study only reported Apgar score at 1 minute).
**Secondary outcomes and results**	**Maternal:**<br>• Blood transfusion: ‘Vaginal push up’ (Group B): 21/64; ‘Patwardhan method’ (Group A): 8/56 (p=0.0259)<br>• Inverted T or J incision: Not reported<br>• Visceral injury (ureteral / bladder / cervical) or hysterectomy:<br>o Uterine incision extension into cervix / vagina: Not reported

	○ Injury to urinary tract (including ureteric injury and bladder injury): The study only reported 'bladder injury' without further details: 'Vaginal push up' (Group B): 3/64; 'Patwardhan method' (Group A): 0/56 (p=0.4967).<br>○ Hysterectomy: 'Vaginal push up' (Group B): 2/64; 'Patwardhan method' (Group A): 0/56 (p=0.4967).<br>• Infection (wound infection / endometritis / maternal sepsis):<br>○ Wound infection: Not reported<br>○ Endometritis: Not reported<br>○ Urinary tract infection: Not reported<br>○ Post-partum pyrexia / maternal sepsis: Not reported<br>• Duration of hospital stay: Not reported<br>• Decision-to-delivery interval: Not reported<br>• Incision-to-delivery interval: Not reported<br>**Perinatal:**<br>• NICU (Neonatal Intensive Care Unit) admission: 'Vaginal push up' (Group B): 13/64; 'Patwardhan method' (Group A): 10/56 (p=1.0000).<br>• Umbilical artery pH / cord pH < 7.10: Not reported<br>• Neonatal death (defined as death within the first 28 days of life): Study reported 'stillbirth' only: 'Vaginal push up' (Group B): 2/64; 'Patwardhan method' (Group A): 0/56 (p=1.0000).<br>• Cost: Not reported
**Risk of bias**	**Assessed by ROBINS-I:**<br>• Bias due to confounding: Serious risk of bias – only limited baseline parameters reported so limited comparisons between baseline groups possible; the authors did not adjust for confounding in any of the analyses.<br>• Bias in selection of participants into study: Serious risk of bias – criteria for selection for each technique unclear.<br>• Bias in classification of interventions: Low risk of bias – clear definitions provided for 'Vaginal push up' and 'Patwardhan method'.<br>• Bias due to deviations from intended intervention: Unclear risk of bias – no deviations from the intended intervention reported.<br>• Bias due to missing data: Unclear risk of bias – outcomes reported for all women, but only limited outcomes reported.<br>• Bias on measurement of outcomes: Moderate risk of bias - unclear definition of some outcomes e.g. position of uterine incision extension not reported, criteria not reported.<br>• Bias in selection of the reported result: Critical risk of bias - no mention of pre-registered protocol or statistical analysis plan, and published in a potential predatory journal according to Beall's List of Potential Predatory Journals and Publishers.<br>• Overall risk of bias: Critical risk of bias.
**Source of funding**	Reported as 'Nil'

14. Lassey 2020

**Study details**	**Full citation:** Lassey S.C., Little S.E., Saadeh M., Patton N., Farber M.K., Bateman B.T., et al. Cephalic Elevation Device for Second-Stage Cesarean Delivery: A Randomized Controlled Trial. Obstetrics Gynecol 2020;135:879–84<br>**Study type:** Randomised controlled trial<br>**Country of study:** United States<br>**Study dates**: January 2018 to July 2019
**Participants**	**Inclusion criteria:** Women aged ≥18 years with a single, full-term (≥37 weeks gestation) fetus in cephalic presentation; nulliparous women<br>**Exclusion criteria:** Women contraindicated to a vaginal delivery, prior caesarean delivery or caesarean delivery during first state of labour, non-English speaking, or presence of congenital fetal anomaly<br>**Participant characteristics:**<br>- Inflated fetal pillow group:<br>o N women: 30<br>o Median (interquartile range) maternal age: 33 (31 to 36) years<br>o Median (interquartile range) maternal BMI: 28.9 (27 to 34) kg/m^2<br>o Smoking status: Not reported<br>o Parity: N=30 (100%) women were nulliparous, as per the inclusion criteria<br>o Diabetes: 2/30<br>o Median (interquartile range) gestational age: 40 (39.1 to 40.6) weeks<br>o Numbers with caesarean section at full (10cm) cervical dilatation: Not explicitly stated, but presumably 30 as women in second stage of labour<br>o Numbers with caesarean section prior to (<10cm) full cervical dilatation: Not explicitly stated, but presumably 0<br>- Non-inflated fetal pillow group:<br>o N women: 30<br>o Median (interquartile range) maternal age: 30.5 (26 to 33) years<br>o Median (interquartile range) maternal BMI: 29.7 (26.6 to 32.5) kg/m^2<br>o Smoking status: Not reported<br>o Parity: N=30 (100%) women were nulliparous, as per the inclusion criteria<br>o Diabetes: 2/30<br>o Median (interquartile range) gestational age: 40 (39.3 to 40.3) weeks<br>o Numbers with caesarean section at full (10cm) cervical dilatation: Not explicitly stated, but presumably 30 as women in second stage of labour<br>o Numbers with caesarean prior to (<10cm) full cervical dilatation: Not explicitly stated, but presumably 0
**Intervention & comparator**	**Intervention:** Inflated fetal pillow: "At the time of cesarean delivery, the cephalic elevation device was inserted vaginally by the obstetrician after catheterization of the bladder and after vaginal preparation with betadine, per our

	current labor and delivery guidelines. Once the cephalic elevation device was inserted, the patient's legs were laid flat on the operating table in accordance with the guidelines for use of this device." (p. 881).<br>**Comparator:** Fetal pillow inserted but not inflated.<br><br>"The cephalic elevation device used in this trial is a soft silicone balloon device that is inserted into the vagina and placed beneath the fetal head and then inflated to help lift the head from the pelvis. Once the cephalic elevation device is inserted, the patient's legs are laid flat and adducted on the operating room table, with the patient in the supine position with left lateral tilt. The cephalic elevation device is inflated with 180 mL of sterile saline" (p. 880). "The circulating nurse accessed the device (both groups) by the catheter to the side of the patient's legs and either deflated it or carried out a mock deflation after delivery of the neonate (inflated vs not-inflated group, respectively). The cephalic elevation device was removed by the delivering provider at the end of the procedure in both groups" (p. 882).
**Primary outcomes and results**	**Maternal:**<br>• Uterine incision extension (angle extensions on lower segments and / or into broad ligaments):<br>○ Incision extension on lower segment (at angles or towards cervix): Inflated fetal pillow: 6/30; Non-inflated fetal pillow: 9/30<br>○ Angle extensions into broad ligaments: Not reported<br>• Operative blood loss / post-partum haemorrhage (operative blood loss > 500ml): Study reported blood loss: Inflated fetal pillow: Median (interquartile range) = 800 (700 to 900) ml; Non-inflated fetal pillow: Median (interquartile range) = 900 (750 to 1050) ml; MD (95% CI): -191.7 ml (-370.3 to -13; p=0.09)<br>• Operative time (duration of surgery): Inflated fetal pillow: Median (interquartile range) = 56 (50 to 62) minutes; Non-inflated fetal pillow: Median (interquartile range) = 59 (52 to 70) minutes; MD (95% CI): -8.7 minutes (-18.5 to 1.2; p=0.14)<br>**Perinatal:**<br>• Infant birth trauma (skull fracture / intracranial haemorrhage / other bony fracture / nerve injury): Not clearly reported<br>• Apgar score at five minutes: Inflated fetal pillow: Median (interquartile range) = 9 (9 to 9); Non-inflated fetal pillow: Median (interquartile range) = 9 (9 to 9); MD (95% CI): -0.1 (-0.5 to 0.3; p=0.84)
**Secondary outcomes and results**	**Maternal:**<br>• Blood transfusion: Inflated fetal pillow: 0/30; Non-inflated fetal pillow: 3/30; RD (95% CI): -0.1 (-0.21 to 0.01; p=0.24)<br>• Inverted T or J incision: Not reported<br>• Visceral injury (ureteral / bladder / cervical) or hysterectomy:<br>○ Uterine incision extension into cervix / vagina: The study reported extension into the cervix, vagina or bladder: Inflated fetal pillow: 0/30; Non-inflated fetal pillow: 4/30<br>○ Injury to urinary tract (including ureteric injury and bladder injury): Not reported<br>○ Hysterectomy: Not reported<br>• Infection (wound infection / endometritis / maternal sepsis):

	○ Wound infection: Not reported<br>○ Endometritis: Not reported<br>○ Urinary tract infection: Not reported<br>○ Post-partum pyrexia / maternal sepsis: Study reported maternal fever: Inflated fetal pillow: 6/30; Non-inflated fetal pillow: 5/30; RD (95% CI): 0.10 (-0.16 to 2.3; p=1.0)<br>• Duration of hospital stay: Inflated fetal pillow: Median (interquartile range) = 4 (4 to 4) days; Non-inflated fetal pillow: Median (interquartile range) = 4 (4 to 4) days; MD (95% CI): -0.1 days (-0.2 to 0.1; p=0.43)<br>• Decision-to-delivery interval: Not reported<br>• Incision-to-delivery interval: Study reported hysterotomy-to-delivery interval: Inflated fetal pillow: Median (interquartile range) = 31 (24 to 37) seconds; Non-inflated fetal pillow: Median (interquartile range) = 54 (41 to 72) seconds; MD (95% CI): -38.2 seconds (-56.1 to -20.3; p<0.01)<br>**Perinatal:**<br>• NICU (Neonatal Intensive Care Unit) admission: Data were not reported for this outcome, but the authors did report NICU length of stay: Inflated fetal pillow: Median (interquartile range) = 0 (0 to 0) days; Non-inflated pillow: Median (interquartile range) = 0 (0 to 0) days; MD (95% CI): -1.1 days (-2.9 to 0.8; p=0.91)<br>• Umbilical artery pH / cord pH < 7.10: Not reported<br>• Neonatal death (defined as death within the first 28 days of life): Not reported<br>Cost: Not reported
**Risk of bias**	**Assessed by RoB2**<br>• Risk of bias arising from the randomisation process: Low risk of bias - an independent consultant used computer-generated randomisation sequence to balance treatment allocation in blocks of ten; allocation kept in sealed, opaque envelopes until time of randomisation; women in the inflated fetal pillow group were older than women in the non-inflated group.<br>• Risk of bias due to deviations from the intended interventions (effect of assignment to intervention): Low risk of bias – delivering provider and other members of the obstetric team were blinded to whether the device was inflated or not; group allocation was revealed to the anaesthesiologist who inflated the device; one device was unsuccessfully inflated for one woman in the inflated fetal pillow group.<br>• Risk of bias due to missing outcome data: Low risk of bias – no missing outcome data.<br>• Risk of bias in measurement of the outcome: Low risk of bias – outcomes measured using objective measurement tools; comparable outcome detection methods and thresholds used, and same definitions and measurements.<br>• Risk of bias in selection of the reported result: Low risk of bias - study approved by review board and registered with ClinicalTrials.gov.<br>Overall risk of bias: Low risk of bias (authors acknowledged that study was underpowered to assess some outcomes)
**Source of funding**	The authors reported that the cephalic elevation devices used in this study were donated by Safe Obstetrics Systems, a medical device company acquired in 2021 by CooperCompanies, a global medical device company.

15. Lenz 2019

**Study details**	**Full citation**: Lenz F., Kimmich N., Zimmermann R., Kreft M. Maternal and neonatal outcome of reverse breech extraction of an impacted fetal head during caesarean section in advanced stage of labour: a retrospective cohort study 2019:1–8<br>**Study type**: Non-randomised comparative retrospective cohort study<br>**Country of study**: Switzerland<br>**Study dates**: December 2012 to December 2016
**Participants**	**Inclusion criteria**: Women at term (≥37+0 weeks pregnancy) with a singleton pregnancy with cephalic presentation and requiring an intrapartum caesarean section at cervical dilation ≥7 cm. In addition, the interventions were only compared in women with difficult fetal extraction, "defined to be difficult when the T-incision or transvaginal head pushing manoeuvres were performed, if the reverse breech extraction was required due to a failed fetal extraction caused by an impacted fetal head, or if declared as a difficult extraction in the surgical report (completed by the surgeon just after performing the caesarean section)" (p. 2).<br>**Exclusion criteria**: Women with multiple pregnancies, fetal anomalies, preterm delivery and non-cephalic presentation.<br>**Participant characteristics**:<br>- Vaginal push up group:<br>○ N women: 82<br>○ Mean (SD) maternal age: 31.7 (5.0) years<br>○ Mean (SD) maternal BMI: BMI before pregnancy 23.9 (5.4) kg/m^2<br>○ Smoking status: Not reported<br>○ Parity: Not reported<br>○ Diabetes: Not reported<br>○ Mean (SD) gestational age: 39.7 (1.0) weeks<br>○ Numbers with caesarean section at full (10cm) cervical dilatation: 58<br>○ Numbers with caesarean section prior to (<10cm) full cervical dilatation: Not directly reported, but presumably 24<br>- Patwardhan method group:<br>○ N women: 55<br>○ Mean (SD) maternal age: 31.6 (5.1) years<br>○ Mean (SD) maternal BMI: BMI before pregnancy: 22.7 (2.9) kg/m^2<br>○ Smoking status: Not reported<br>○ Parity: Not reported<br>○ Diabetes: Not reported<br>○ Mean (SD) gestational age: 39.5 (1.1) weeks<br>○ Numbers with caesarean section at full (10cm) cervical dilatation: 45

	○ Numbers with caesarean prior to (<10cm) full cervical dilatation: Not directly reported, but presumably 10
**Intervention & comparator**	**Intervention**: 'Vaginal push up': "The conventional head pushing method during caesarean section was performed by lifting the fetal head out of the maternal pelvis by the surgeon's hand. If the surgeon was not able to lift the head, vaginal dislodge by the help of an assistant's hand or an inserted silicone cup (normally used for vacuum deliveries) was additionally performed, so the surgeon could finally deliver the fetal head through the uterine incision" (p. 2).<br>**Comparator**: 'Patwardhan method' ('Pull method') for the fetus in occiput posterior position. After uterine incision the fetal arms are extracted and then the feet and both legs are delivered from the fundal uterine region. After extraction of the fetal body by pulling symmetrically on both legs, sometimes accompanied by fundal pressure, the head is disengaged from the maternal pelvis by using an unscrewing manoeuvre (unscrewing by grasping the body and shoulders simultaneously and rotating the fetus very carefully).<br>'Patwardhan method' for the fetus in occiput anterior position involves the surgeon grasping the babies back with both hands and pulling them to reach the breech. Fundal pressure may be helpful to extract the body, and then the unscrewing manoeuvre (as described for occiput posterior position) is similarly applied.
**Primary outcomes and results**	**Maternal:**<br>• Uterine incision extension (angle extensions on lower segments and / or into broad ligaments):<br>○ Incision extension on lower segment (at angles or towards cervix): 'Vaginal push up': 29/82; 'Patwardhan method': 5/55 (p=<0.001)<br>○ Angle extensions into broad ligaments: Not reported<br>• Operative blood loss / post-partum haemorrhage (operative blood loss > 500 ml): Study reported operative blood loss: 'Vaginal push up': Mean (SD) = 712.2 (375.0) mL; 'Patwardhan method': Mean (SD) = 562.7 (195.1) mL (p=0.009)<br>• Operative time (duration of surgery): 'Vaginal push up': Mean (SD) = 44.8 (16.7) minutes; 'Patwardhan method': Mean (SD) = 38.3 (18.4) minutes (p=0.006)<br>**Perinatal:**<br>• Infant birth trauma (skull fracture / intracranial haemorrhage / other bony fracture / nerve injury): 'Vaginal push up': Number = 2/82 (both events were due to perinatal skull fractures that resulted in neonatal admissions); 'Patwardhan method': Number = 1/55 (fetal humours fracture)<br>• Apgar score at five minutes not reported, but Apgar score < 7 at five minutes was: 'Vaginal push up': 3/82; 'Patwardhan method': 0/55 (p=0.274)
**Secondary outcomes and results**	**Maternal:**<br>• Blood transfusion: Not reported<br>• Inverted T or J incision: Study reported T incisions: 'Vaginal push up': 5/82; 'Patwardhan method': 2/55 (p=0.702)<br>• Visceral injury (ureteral / bladder / cervical) or hysterectomy:<br>○ Uterine incision extension into cervix / vagina: Not reported<br>○ Injury to urinary tract (including ureteric injury and bladder injury): Not reported<br>○ Hysterectomy: Not reported

	• Infection (wound infection / endometritis / maternal sepsis):<br>○ Wound infection: Not reported<br>○ Endometritis: Not reported<br>○ Urinary tract infection: Not reported<br>○ Post-partum pyrexia / maternal sepsis: Not reported<br>• Duration of hospital stay: Not reported<br>• Decision-to-delivery interval: Not reported<br>• Incision-to-delivery interval: ‘Vaginal push up’: Mean (SD) = 7 (3.2) minutes; ‘Patwardhan method’: Mean (SD) = 6.7 (2.5) minutes (p=0.979)<br>**Perinatal:**<br>• NICU (Neonatal Intensive Care Unit) admission: Study reported transmission to neonatology unit: ‘Vaginal push up’: 2/82; ‘Patwardhan method’: 1/55 (p=1.0)<br>• Umbilical artery pH / cord pH < 7.10: Not reported but Umbilical artery pH < 7.15 was: ‘Vaginal push up’: 8/82; ‘Patwardhan method’: 4/55 (p=0.768)<br>• Neonatal death (defined as death within the first 28 days of life): Study reported neonatal death (no further information): ‘Vaginal push up’: 1/82; ‘Patwardhan method’: 0/55 (p=1.0)<br>Cost: Not reported
**Risk of bias**	**Assessed by ROBINS-I:**<br>• Bias due to confounding: Serious risk of bias - the authors acknowledged that the risk of bias due to potential differences in experience, skills, and knowledge of the surgeons have to be taken into consideration; more women in the 'Patwardhan method' had cephalic malpresentation compared to women in the 'Vaginal push up'; the authors did not adjust for confounding in any of the analyses.<br>• Bias in selection of participants into study: Low risk of bias – selection of participants does not appear to be related to both intervention and outcomes; initial follow-up time appears to be similar for all women.<br>• Bias in classification of interventions: Low risk of bias - clear definitions provided for ‘Vaginal push up’ and ‘Patwardhan method’.<br>• Bias due to deviations from intended intervention: Low risk of bias - no deviations from the intended intervention reported.<br>• Bias due to missing data: Unclear risk of bias – no information provided.<br>• Bias on measurement of outcomes: Low risk of bias - outcomes measured using objective measurement tools; comparable outcome detection methods and thresholds used, and same definitions and measurements.<br>• Bias in selection of the reported result: Moderate risk of bias - no mention of pre-registered protocol or statistical analysis plan.<br>• Overall risk of bias: Serious risk of bias.
**Source of funding**	None

16. Nooh 2017

Study details	Full citation: Nooh A.M., Abdeldayem H.M., Ben-Affan O. Reverse breech extraction versus the standard approach of pushing the impacted fetal head up through the vagina in caesarean section for obstructed labour: A randomised controlled trial. Journal of Obstetrics & Gynaecology 2017;37:459–63<br>**Study type:** Randomised controlled trial<br>**Country of study:** Egypt<br>**Study dates:** June 2012 to November 2013
**Participants**	**Inclusion criteria:** Women with single, term pregnancy (37 to 42 weeks) in cephalic presentation who were actively pushing with uterine contractions in the second stage of labour for ≥2 hours (multipara) or ≥3 hours (primipara); fetal head at ≥1+ station with obstructed labour and in need of abdominal delivery<br>**Exclusion criteria:** Women with multiple pregnancy; preterm labour (<37 weeks gestation); non-cephalic presentation; previous uterine scar<br>**Participant characteristics:**<br>- Vaginal push up group:<br>o N women: 96<br>o Mean (SD) maternal age: 22.1 (4.3) years<br>o Mean (SD) maternal BMI: Not reported<br>o Smoking status: Not reported<br>o Parity: Mean/median: 3 (range 0 to 6)<br>o Diabetes: Not reported<br>o Mean (SD) gestational age: 39.5 (2.3) weeks<br>o Numbers with caesarean section at full (10cm) cervical dilatation: Not explicitly stated, but presumably 96 as per the inclusion criteria<br>o Numbers with caesarean prior to (<10cm) full cervical dilatation: Not explicitly stated, but presumably 0 as per the inclusion criteria<br>- Reverse breech extraction group:<br>o N women: 96<br>o Mean (SD) maternal age: 21.8 (4.4) years<br>o Mean (SD) maternal BMI: Not reported<br>o Smoking status: Not reported<br>o Parity: Mean/median: 3 (range 0 to 5)<br>o Diabetes: Not reported<br>o Mean (SD) gestational age: 39.4 (2.4) weeks<br>o Numbers with caesarean section at full (10cm) cervical dilatation: Not explicitly stated, but presumably 96 as per the inclusion criteria

	○ Numbers with caesarean section prior to (<10cm) full cervical dilatation: Not explicitly stated, but presumably 0 as per the inclusion criteria
**Intervention & comparator**	**Intervention:** 'Vaginal push up': "In the 'standard approach' group, an assistant, inserting one hand into the vagina, pushed the fetal head up until it was easily grasped by hand of the operating Obstetrician. The later then dislodged the fetal head from the maternal pelvis and delivered it through the CS incision. The rest of baby's body followed in the usual way" (p. 460).<br><br>**Comparator:** 'Reverse breech extraction': "In the 'reverse breech extraction approach' group, the main operating hand of the obstetric surgeon was inserted upward inside the uterine cavity to find and grasp a baby's foot and deliver it along with the ipsilateral leg through the uterine incision. This in turn brought the other leg into the operative field. The surgeon then grasped both feet and proceeded in a manner similar to that practiced in vaginal breech extraction" (p. 460).<br><br>After delivery, all women received antibiotics, and if required, women received anti-thromboembolic treatment, in accordance with the hospital protocol.
**Primary outcomes and results**	**Maternal:**<br>• Uterine incision extension (angle extensions on lower segments and / or into broad ligaments):<br>○ Incision extension on lower segment (at angles or towards cervix): Study reported "inadvertent increase in the length of incision beyond normal limits: any extra length beyond the initial incision made by the scalpel, with or without the use of scissors and/or digital widening, which can go laterally further than the anterior surface of the uterus or downwards, that required anything more than the standard uterine closure" (p. 460): 'Vaginal push up': 46/96; 'Reverse breech extraction': 18/96 (p=0.0003)<br>○ Angle extensions into broad ligaments: Not reported<br>• Operative blood loss / post-partum haemorrhage (operative blood loss > 500ml): Study reported operative blood : 'Vaginal push up': Mean (SD) = 1367 (322) ml; 'Reverse breech extraction': Mean (SD) = 832 (221) ml (p=0.00); Post-partum haemorrhage: 'Vaginal push up': 5/96; 'Reverse breech extraction': 2/96 (p=0.25)<br>• Operative time (duration of surgery): 'Vaginal push up': Mean (SD) = 71.2 (5.4) minutes; 'Reverse breech extraction': Mean (SD) = 56.3 (4.7) minutes (p=0.00)<br>**Perinatal:**<br>• Infant birth trauma (skull fracture / intracranial haemorrhage / other bony fracture / nerve injury): Not reported<br>• Apgar score at five minutes / Apgar score < 7 at five minutes: Study reported Apgar score < 7 at five minutes: 'Vaginal push up': 21/96; 'Reverse breech extraction': 8/96 (p=0.015)
**Secondary outcomes and results**	**Maternal:**<br>• Blood transfusion: Intra-operative blood transfusion: 'Vaginal push up': 11/96; 'Reverse breech extraction': 2/96 (p=0.012); Post-operative blood transfusion: 'Vaginal push up': 3/96; 'Reverse breech extraction': 1/96 (p=0.29)<br>• Inverted T or J incision: Not reported

	• Visceral injury (ureteral / bladder / cervical) or hysterectomy:<br>o Uterine incision extension into cervix / vagina: Not reported<br>o Injury to urinary tract (including ureteric injury and bladder injury): bladder injury: 'Vaginal push up': 5/96; 'Reverse breech extraction': 2/96 (p=0.25<br>o Hysterectomy: Study reported Caesarean hysterectomy: 'Vaginal push up': 1/96; 'Reverse breech extraction': 0/96 (p=0.31)<br>• Infection (wound infection / endometritis / maternal sepsis):<br>o Wound infection: 'Vaginal push up': 12/96; 'Reverse breech extraction': 2/96 (p=0.007)<br>o Endometritis: Not reported<br>o Urinary tract infection: Not reported<br>o Post-partum pyrexia / maternal sepsis: 'Vaginal push up': 19/96; 'Reverse breech extraction': 3/96 (p=0.0006)<br>• Duration of hospital stay: 'Vaginal push up': Mean (SD) = 3.9 (1.2) days; 'Reverse breech extraction': Mean (SD) = 3.1 (1.5) days (p=0.00)<br>• Decision-to-delivery interval: Not reported separately for treatment arms (range between 30 and 75 minutes)<br>• Incision-to-delivery interval: Not reported<br>**Perinatal:**<br>• NICU (Neonatal Intensive Care Unit) admission: Study reported special Care Baby Unit admission: 'Vaginal push up': 15/96; 'Reverse breech extraction': 7/96 (p=0.088)<br>• Umbilical artery pH / cord pH < 7.10: Not reported<br>• Neonatal death (defined as death within the first 28 days of life): Study reported neonatal death (no further information): 'Vaginal push up': 3/96; 'Reverse breech extraction': 0/96 (p=0.08)<br>Cost: Not reported
**Risk of bias**	**Assessed by RoB2:**<br>• Risk of bias arising from the randomisation process: Low risk of bias - computer-generated randomisation sequence using serially numbered, opaque, sealed envelopes; envelope opened after recruitment; similar baseline characteristics between intervention arms.<br>• Risk of bias due to deviations from the intended interventions (effect of assignment to intervention): Low risk of bias – surgeons and participants were not aware of allocation prior to intended caesarean section; no deviations from intended intervention.<br>• Risk of bias due to missing outcome data: Low risk of bias – no reported missing outcome data.<br>• Risk of bias in measurement of the outcome: Low risk of bias – outcomes measured using objective measurement tools; comparable outcome detection methods and thresholds used, and same definitions and measurements.<br>• Risk of bias in selection of the reported result: Some concerns – no information provided.<br>Overall risk of bias: Some concerns.
**Source of funding**	Not reported

17. Rakholia 2019

**Study details**	**Full citation:** Rakholia R., Jain G. Comparison of Patwardhan and Push method for impacted fetal head extraction during Caesarean section. Int. J. Adv. Res. 2019; 7(4), 1203-1206.<br>**Study type:** Non-randomised comparative retrospective cohort study<br>**Country of study:** India<br>**Study dates**: Not reported
**Participants**	**Inclusion criteria:** Women with single fetus at term in vertex presentation with full cervical dilatation with deeply impacted fetal head in maternal pelvis.<br>**Exclusion criteria:** Multiple pregnancies, previous caesarean section/myomectomy, antepartum haemorrhage, pregnancy less than 37 weeks<br>**Participant characteristics:**<br>- Vaginal push up ('push') group:<br>○ N women: 54<br>○ Mean (SD) maternal age: Not reported but study states age was comparable between groups.<br>○ Mean (SD) maternal BMI: Not reported<br>○ Smoking status: Not reported<br>○ Parity: Not reported but study states parity was comparable between groups.<br>○ Diabetes: Not reported<br>○ Mean (SD) gestational age: Not reported<br>○ Numbers with caesarean section at full (10cm) cervical dilatation: 54 (100%) as per the inclusion criteria<br>○ Numbers with caesarean section prior to (<10cm) full cervical dilatation: 0 as per the inclusion criteria<br>- Patwardhan method group:<br>○ N women: 62<br>○ Mean (SD) maternal age: Not reported but study states age was comparable between groups.<br>○ Mean (SD) maternal BMI: Not reported<br>○ Smoking status: Not reported<br>○ Parity: Not reported but study states age was comparable between groups.<br>○ Diabetes: Not reported<br>○ Mean (SD) gestational age: Not reported<br>○ Numbers with caesarean section at full (10cm) cervical dilatation: 62 (100%) as per the inclusion criteria<br>○ Numbers with caesarean prior to (<10cm) full cervical dilatation: 0 as per the inclusion criteria
**Intervention & comparator**	**Intervention:** 'Vaginal push up' ('push' method): 'fetal head is dislodged by pushing it through vagina' (no further details given.<br>**Comparator:** 'In case of occipito-anterior and transverse positions with the head deeply impacted in the pelvis, incision is made in the lower uterine segment, shoulders are present usually at incision level in deeply engaged head ,the anterior shoulder is delivered out by hooking the arm. With gentle traction on shoulder, the posterior shoulder is also

	delivered out. Next, the surgeon holds the trunk of baby gently with both thumbs parallel to spine and with fundal pressure given by assistant the buttocks are delivered followed by legs. Now the baby's head which is the only part of the foetus which is still inside the uterus, is gently lifted out of the pelvis by making an arc'.
**Primary outcomes and results**	**Maternal:**<br>• Uterine incision extension (angle extensions on lower segments and / or into broad ligaments): Study reported 'extension of uterine incision' without giving further details: 'Vaginal push up': 10/54; 'Patwardhan method': 1/62 (p=0.002).<br>○ Incision extension on lower segment (at angles or towards cervix): Not specifically reported<br>○ Angle extensions into broad ligaments: Not specifically reported<br>• Operative blood loss / post-partum haemorrhage (operative blood loss >1000 ml): Study reported 'traumatic PPH' without giving a definition: 'Vaginal push up': 8/54; 'Patwardhan method': 1/62 (p=0.009).<br>• Operative time (duration of surgery): Not reported<br>**Perinatal:**<br>• Infant birth trauma (skull fracture / intracranial haemorrhage / other bony fracture / nerve injury): Not reported<br>• Apgar score at five minutes / Apgar score < 7 at five minutes: Study reported Apgar score <7 at five minutes: 'Vaginal push up': 5/54; 'Patwardhan method': 3/62 (p=0.18).
**Secondary outcomes and results**	**Maternal:**<br>• Blood transfusion: 'Vaginal push up': 16/54; 'Patwardhan method': 1/62 (p=<0.001)<br>• Inverted T or J incision: Not reported<br>• Visceral injury (ureteral / bladder / cervical) or hysterectomy:<br>○ Uterine incision extension into cervix / vagina: Not reported<br>○ Injury to urinary tract (including ureteric injury and bladder injury): Not reported<br>○ Hysterectomy: Not reported<br>• Infection (wound infection / endometritis / maternal sepsis):<br>○ Wound infection: Not reported<br>○ Endometritis: Not reported<br>○ Urinary tract infection: Not reported<br>○ Post-partum pyrexia / maternal sepsis: Not reported<br>• Duration of hospital stay: Not reported<br>• Decision-to-delivery interval: Not reported<br>• Incision-to-delivery interval: Not reported<br>**Perinatal:**<br>• NICU (Neonatal Intensive Care Unit) admission: 'Vaginal push up': 9/54; 'Patwardhan method': 5/62 (p=0.15)<br>• Umbilical artery pH / cord pH < 7.10: Not reported<br>• Neonatal death (study reported 'neonatal death' without giving a definition: 'Vaginal push up': 3/54; 'Patwardhan method': 1/62 (p=0.23)

	Cost: Not reported
**Risk of bias**	**Assessed by ROBINS-I:**<br>• Bias due to confounding: Serious risk of bias – only limited baseline parameters reported so limited comparisons between baseline groups possible; the authors did not adjust for confounding in any of the analyses.<br>• Bias in selection of participants into study. Serious risk of bias – criteria for selection for each technique unclear.<br>• Bias in classification of interventions: Low risk of bias – clear definitions provided for 'Vaginal push up' and 'Patwardhan method'.<br>• Bias due to deviations from intended intervention: Unclear risk of bias – not reported.<br>• Bias due to missing data: Unclear risk of bias – outcomes reported for all women, but limited outcomes reported.<br>• Bias on measurement of outcomes: Moderate risk of bias - unclear definition of some outcomes e.g. position of uterine incision extension not reported, criteria not reported.<br>• Bias in selection of the reported result: Critical risk of bias - no mention of pre-registered protocol or statistical analysis plan, and published in a potential predatory journal according to Beall's List of Potential Predatory Journals and Publishers.<br>• Overall risk of bias: Critical risk of bias.
**Source of funding**	Not reported

18. Sacre 2021

**Study details**	**Full citation:** Sacre H., Bird A., Clement-Jones M., Sharp A. Effectiveness of the fetal pillow to prevent adverse maternal and fetal outcomes at full dilatation cesarean section in routine practice. Acta Obstet Gyn Scan 2021;100:949–54<br>**Study type:** Non-randomised comparative retrospective cohort study<br>**Country of study:** UK<br>**Study dates**: September 2014 to March 2018
**Participants**	**Inclusion criteria:** Women with a live single pregnancy at full dilatation<br>**Exclusion criteria:** Women with multiple pregnancy, breech presentations, not at full dilation or stillbirths<br>**Participant characteristics:**<br>- Fetal pillow group:<br>o N women: 170<br>o Median (interquartile range) maternal age: 30 (27 to 33) years<br>o Median (interquartile range) maternal BMI: 25.7 (22.9 to 28.5) kg/m^2<br>o Smoking status: Not reported<br>o Parity: N=148 (87.1%) women were nulliparous, no further details provided<br>o Diabetes: Not reported<br>o Mean (SD) gestational age: Not reported<br>o Numbers with caesarean section at full (10cm) cervical dilatation: Not specifically stated, but presumably 170 as all women at full dilatation<br>o Numbers with caesarean section prior to (<10cm) full cervical dilatation: Not specifically stated, but presumably 0<br>- No-fetal pillow group:<br>o N women: 221<br>o Median (interquartile range) maternal age: 30 (27 to 34) years<br>o Median (interquartile range) maternal BMI: 25.7 (23.1 to 29.5) kg/m^2<br>o Smoking status: Not reported<br>o Parity: N=161 (72.9%) women were nulliparous, no further details provided<br>o Diabetes: Not reported<br>o Mean (SD) gestational age: Not reported<br>o Numbers with caesarean section at full (10cm) cervical dilatation: Not specifically stated, but presumably 221 as all women at full dilatation<br>o Numbers with caesarean prior to (<10cm) full cervical dilatation: Not specifically stated, but presumably 0
**Intervention & comparator**	**Intervention:** Fetal pillow: “The fetal pillow is a one-use disposable silicone device consisting of a soft flat base with a balloon compartment, which can be inserted into the vagina and then inflated in order to elevate the fetal head before FDCS [caesarean section at full cervical dilatation]” (p. 950).

	**Comparator:** No fetal pillow
**Primary outcomes and results**	**Maternal:**<br>• Uterine incision extension (angle extensions on lower segments and / or into broad ligaments):<br>○ Incision extension on lower segment (at angles or towards cervix): Study reported this outcome as defined by the attending clinician: Fetal pillow: 37/170; No-fetal pillow: 47/221; RR (95% CI) 1.02 (0.70 to 1.50; p=0.91)<br>○ Angle extensions into broad ligaments: Not reported<br>• Operative blood loss / post-partum haemorrhage (operative blood loss > 500ml): Study reported estimated blood loss: >1000 ml: Fetal pillow: 39/170; No-fetal pillow: 41/221, RR (95% CI): 1.24 (0.84 to 1.83; p=0.29); estimated blood loss >1500 ml: Fetal pillow: 15/170; No-fetal pillow: 14/221, RR (95% CI) 1.39 (0.69 to 2.81; p=0.35); estimated blood loss (mL): Fetal pillow: Median (interquartile range) = 600 (500 to 900); No-fetal pillow: Median (interquartile range) = 600 (500 to 800)<br>• Operative time (duration of surgery): Not reported<br>**Perinatal:**<br>• Infant birth trauma (skull fracture / intracranial haemorrhage / other bony fracture / nerve injury): Not reported<br>• Apgar score at five minutes / Apgar score < 7 at five minutes: Study reported Apgar score <7 at five minutes: Fetal pillow: 12/170; No-fetal pillow: 12/221; RR (95% CI) 1.30 (0.60 to 2.82; p=0.51)
**Secondary outcomes and results**	**Maternal:**<br>• Blood transfusion: Fetal pillow: 8/170; No-fetal pillow: 9/221; RR (95% CI) 1.16 (0.46 to 2.9; p=0.76)<br>• Inverted T or J incision: Not reported<br>• Visceral injury (ureteral / bladder / cervical) or hysterectomy:<br>○ Uterine incision extension into cervix / vagina: Not reported<br>○ Injury to urinary tract (including ureteric injury and bladder injury): Not reported<br>○ Hysterectomy: Not reported<br>• Infection (wound infection / endometritis / maternal sepsis):<br>○ Wound infection: Not reported<br>○ Endometritis: Not reported<br>○ Urinary tract infection: Not reported<br>○ Post-partum pyrexia / maternal sepsis: Not reported<br>• Duration of hospital stay (delivery to discharge): Fetal pillow: Median (interquartile range) = 2 (2 to 3) days; No-fetal pillow: Median (interquartile range) = 2 (2 to 3) days<br>• Decision-to-delivery interval: Not reported<br>• Incision-to-delivery interval: Not reported<br>**Perinatal:**<br>• NICU (Neonatal Intensive Care Unit) admission: Fetal pillow: 15/170; No-fetal pillow: 27/221; RR (95% CI) 0.72 (0.40 to 1.31; p=0.29)

	• Umbilical artery pH / cord pH < 7.10: Fetal pillow: 12/170; No-fetal pillow: 29/221; RR (95% CI) 0.54 (0.28 to 1.02; p=0.06)<br>• Neonatal death (defined as death within the first 28 days of life): Not reported<br>Cost: Not reported
**Risk of bias**	**Assessed by ROBINS-I:**<br>• Bias due to confounding: Serious risk of bias - there were statistically significantly fewer women in the Fetal pillow group with station above the ischial spines (p<0 .0001) and more below the ischial spines (p<0.0001); there were more nulliparous women managed with fetal pillow (p=0.0009); the decision to use fetal pillow or not was at the clinicians discretion; the authors did not adjust for confounding in any of the analyses.<br>• Bias in selection of participants into study: Low risk of bias – the authors acknowledged that it was possible that patient selection for women who received the fetal pillow had an influence on the findings; initial follow-up time appears to be similar for all women.<br>• Bias in classification of interventions: Moderate risk of bias – clear definitions were not provided for either method.<br>• Bias due to deviations from intended intervention: Low risk of bias – no deviations from the intended intervention reported.<br>• Bias due to missing data: Low risk of bias – no reported missing data.<br>• Bias on measurement of outcomes: Low risk of bias - outcomes measured using objective measurement tools; comparable outcome detection methods and thresholds used, and same definitions and measurements.<br>• Bias in selection of the reported result: Moderate risk of bias - no mention of pre-registered protocol or statistical analysis plan.<br>Overall risk of bias: Serious risk of bias.
**Source of funding**	Not reported

19. Safa 2016

**Study details**	**Full citation:** Safa H., Beckmann M. Comparison of maternal and neonatal outcomes from full-dilatation cesarean deliveries using the Fetal Pillow or hand-push method. International Journal of Gynecology & Obstetrics 2016;135:281–4<br>**Study type:** Non-randomised comparative retrospective cohort study<br>**Country of study:** Australia<br>**Study dates**: May 2013 to March 2015
**Participants**	**Inclusion criteria:** Women with single pregnancy at 37+0 weeks or more of pregnancy and at full dilatation<br>**Exclusion criteria:** Women with multiple pregnancy, pregnancies resulting in fetal death in utero, and major fetal congenital anomalies<br>**Participant characteristics:**<br>- Fetal pillow group:<br>o N women: 91<br>o Mean (SD) maternal age: 29.94 (4.5) years<br>o Mean (SD) maternal BMI: 24.7 (6.1) kg/m^2<br>o Smoking status: Not reported<br>o Parity: N=75 (82%) women were nulliparous, no further details provided<br>o Diabetes: Not reported<br>o Mean (SD) gestational age: 39.7 (1.1) weeks<br>o Numbers with caesarean section at full (10cm) cervical dilatation: Not specifically reported, but presumably 91 as all women at full dilatation as per inclusion criteria<br>o Numbers with caesarean section prior to (<10cm) full cervical dilatation: Not specifically reported, but presumably 0 as per inclusion criteria<br>- ‘Vaginal push up’ group:<br>o N women: 69<br>o Mean (SD) maternal age: 31 (4.9) years<br>o Mean (SD) maternal BMI: 24 (4.5) kg/m^2<br>o Smoking status: Not reported<br>o Parity: N=45 (65%) women were nulliparous, no further details provided<br>o Diabetes: Not reported<br>o Mean (SD) gestational age: 39.8 (1.1) weeks<br>o Numbers with caesarean section at full (10cm) cervical dilatation: Not specifically reported, but presumably 69 as all women at full dilatation as per inclusion criteria<br>o Numbers with caesarean prior to (<10cm) full cervical dilatation: Not specifically reported, but presumably 0 as per inclusion criteria

**Intervention & comparator**	**Intervention:** Fetal pillow: "use a Fetal Pillow to elevate the fetal head and facilitate delivery during cesarean deliveries at full dilatation. The Fetal Pillow is a soft silicone balloon attached to 100-cm tubing with a two way tap that controls the inflation/deflation of the device. Prior to scrubbing, the device is inserted vaginally by the surgeon by folding the base plate with the balloon surface in contact with the fetal head; the device is then advanced posteriorly in the same manner as inserting a posterior ventouse cup. The maternal legs are then laid flat. A total of 180 mL of saline is used for inflation of the device using the provided 60-mL syringe prior to skin incision. Upon inflation, the base plate opens flat against the pelvic floor and gently elevates the fetal head 3–4 cm from its original position" (p. 282).<br>**Comparator:** 'Vaginal push up': No further details.
**Primary outcomes and results**	**Maternal:**<br>• Uterine incision extension (angle extensions on lower segments and / or into broad ligaments):<br>○ Incision extension on lower segment (at angles or towards cervix): Study reported uterine angle extension: Fetal pillow: 18/91; 'Vaginal push up': 24/69 (p=0.061)<br>○ Angle extensions into broad ligaments: Not reported<br>• Operative blood loss / post-partum haemorrhage (operative blood loss > 500 ml): Study reported estimated blood loss: Fetal pillow: Mean (SD) = 273 (145) ml; 'Vaginal push up': Mean (SD) = 403 (199) ml (p=0.026)<br>• Operative time (duration of surgery): Not reported<br>**Perinatal:**<br>• Infant birth trauma (skull fracture / intracranial haemorrhage / other bony fracture / nerve injury): Not reported<br>• Apgar score at five minutes / Apgar score < 7 at five minutes: Study reported Apgar score < 7 at five minutes: Fetal pillow: 3/91; 'Vaginal push up': 4/69 (p=0.444)
**Secondary outcomes and results**	**Maternal:**<br>• Blood transfusion: Fetal pillow: 3/91; 'Vaginal push up': 2/69 (p=0.753)<br>• Inverted T or J incision: Not reported<br>• Visceral injury (ureteral / bladder / cervical) or hysterectomy:<br>○ Uterine incision extension into cervix / vagina: Not reported<br>○ Injury to urinary tract (including ureteric injury and bladder injury): Not reported<br>○ Hysterectomy: Not reported<br>• Infection (wound infection / endometritis / maternal sepsis):<br>○ Wound infection: Not reported<br>○ Endometritis: Not reported<br>○ Urinary tract infection: Not reported<br>○ Post-partum pyrexia / maternal sepsis: Not reported<br>• Duration of hospital stay (postpartum hospital stay): Fetal pillow: Mean (SD) = 77.9 (19.6) hours; 'Vaginal push up': Mean (SD) = 97.8 (27.6) hours (p=0.002)<br>• Decision-to-delivery interval: Not reported<br>• Incision-to-delivery interval: Not reported

	**Perinatal:**<br>• NICU (Neonatal Intensive Care Unit) admission: Fetal pillow: 14/91; 'Vaginal push up': 17/69 (p=0.142)<br>• Umbilical artery pH / cord arterial pH: Fetal pillow: Mean (SD) = 7.24 (0.06); 'Vaginal push up': Mean (SD) = 7.19 (0.09) (p=0.003)<br>• Neonatal death (defined as death within the first 28 days of life): Not reported<br>Cost: Not reported
**Risk of bias**	**Assessed by ROBINS-I:**<br>• Bias due to confounding: Serious risk of bias – more women in the 'hand-push method' group had previous failed instrumental delivery compared to the 'fetal pillow' group but this was not statistically significant; the authors did not adjust for confounding in any of the analyses.<br>• Bias in selection of participants into study: Low risk of bias – selection of participants does not appear to be related to both intervention and outcomes; initial follow-up time appears to be similar for all women.<br>• Bias in classification of interventions: Moderate risk of bias – clear definitions provided for 'Fetal pillow' but limited details provided on 'Vaginal push up'.<br>• Bias due to deviations from intended intervention: Low risk of bias – no deviations from the intended intervention reported.<br>• Bias due to missing data: Low risk of bias – data appear to be reported for all included women.<br>• Bias on measurement of outcomes: Low risk of bias - outcomes measured using objective measurement tools; comparable outcome detection methods and thresholds used, and same definitions and measurements.<br>• Bias in selection of the reported result: Moderate risk of bias - no mention of pre-registered protocol or statistical analysis plan.<br>Overall risk of bias: Serious risk of bias.
**Source of funding**	Not reported

20. Saha 2014

**Study details**	**Full citation:** Saha P.K. Second Stage Caesarean Section Evaluation of Patwardhan Technique. Journal of Clinical and Diagnostic Research, 2014:1-3<br>**Study type:** Non-randomised comparative retrospective cohort study<br>**Country of study:** India<br>**Study dates:** 2004 to 2006
**Participants**	**Inclusion criteria:** Women undergoing caesarean sections performed in full dilatation of cervix<br>**Exclusion criteria:** Not reported<br>**Participant characteristics:**<br>- Vaginal push up or Reverse breech extraction group:<br>o N women: 44<br>o Maternal age: Not reported<br>o Maternal BMI: Not reported<br>o Smoking status: Not reported<br>o Parity: Not reported<br>o Diabetes: Not reported<br>o Mean (SD) gestational age: 39.32 (SD: not reported) weeks<br>Period of gestation <37 weeks: 2/44; 37 to 40 weeks: 28/44; >40 weeks: 14/44<br>o Numbers with caesarean section at full (10cm) cervical dilatation: 44, all women at full dilatation of cervix<br>o Numbers with caesarean prior to (<10cm) full cervical dilatation: 0<br>- Patwardhan method group:<br>o N women: 35<br>o Maternal age: Not reported<br>o Maternal BMI: Not reported<br>o Smoking status: Not reported<br>o Parity: Not reported<br>o Diabetes: Not reported<br>o Mean (SD) gestational age: 38.69 (SD: not reported) weeks<br>Period of gestation <37 weeks: 4/35; 37 to 40 weeks: 25/35; >40 weeks: 6/35<br>o Numbers with caesarean section at full (10cm) cervical dilatation: 35, all women at full dilatation of cervix<br>o Numbers with caesarean section prior to (<10cm) full cervical dilatation: 0
**Intervention & comparator**	**Intervention:** 'Vaginal push up or Reverse breech extraction': "Extraction of the impacted foetal head may be done by 'push method', i.e., pushing through the vagina or by "pull" method, i.e., a reverse breech technique" (p.93).<br><br>**Comparator:** 'Patwardhan method': "In case of occipito-transverse or occipito-anterior positions with the head deeply impacted in the pelvis, incision is made in the lower uterine segment, at the level of the anterior shoulder, which is

	delivered out. With gentle traction on this shoulder, the posterior shoulder is also delivered out. Next, the surgeon hooks the fingers through both the axillae and with gentle traction, aided by fundal pressure applied by assistant, the body of the foetus is brought out of the uterus. Now the baby's head which is the only part of the foetus which is still inside the uterus, is gently lifted out of the pelvis" (p. 93).
**Primary outcomes and results**	**Maternal:**<br>• Uterine incision extension (angle extensions on lower segments and / or into broad ligaments):<br>○ Incision extension on lower segment (at angles or towards cervix): 'Vaginal push up or Reverse breech extraction': 10/44; 'Patwardhan method': 0/35 (p=0.002)<br>○ Angle extensions into broad ligaments: Not reported<br>• Operative blood loss / post-partum haemorrhage (operative blood loss > 500ml): Not reported<br>• Operative time (duration of surgery): Not reported<br>**Perinatal:**<br>• Infant birth trauma (skull fracture / intracranial haemorrhage / other bony fracture / nerve injury): Authors report in the discussion that "there was no increased risk of neonatal injuries or asphyxia with this technique, as was compared to that seen in vertex or breech extractions" (p. 94) but no further or numerical details provided.<br>• Apgar score at five minutes / Apgar score < 7 at five minutes: Study reported Apgar score < 7 at five minutes: 'Vaginal push up or Reverse breech extraction': 6/44; 'Patwardhan method': 4/35 (p=0.732)
**Secondary outcomes and results**	**Maternal:**<br>• Blood transfusion: 'Vaginal push up or Reverse breech extraction': 12/44; 'Patwardhan method': 3/35 (p=0.32)<br>• Inverted T or J incision: Not reported<br>• Visceral injury (ureteral / bladder / cervical) or hysterectomy:<br>○ Uterine incision extension into cervix / vagina: Not reported<br>○ Injury to urinary tract (including ureteric injury and bladder injury): Not reported<br>○ Hysterectomy: Not reported<br>• Infection (wound infection / endometritis / maternal sepsis):<br>○ Wound infection: Not reported<br>○ Endometritis: Not reported<br>○ Urinary tract infection: Not reported<br>○ Post-partum pyrexia / maternal sepsis: Not reported<br>• Duration of hospital stay: Not reported<br>• Decision-to-delivery interval: Not reported<br>• Incision-to-delivery interval: Not reported<br>**Perinatal:**<br>• NICU (Neonatal Intensive Care Unit) admission: 'Vaginal push up or Reverse breech extraction': 9/44; 'Patwardhan method': 7/35 (p=0.594)

	• Umbilical artery pH / cord pH < 7.10: Not reported<br>• Neonatal death (defined as death within the first 28 days of life): Not reported<br>Cost: Not reported
**Risk of bias**	**Assessed by ROBINS-I:**<br>• Bias due to confounding: Serious risk of bias - caesarean sections were performed by third year registrars or consultants; the authors did not adjust for confounding in any of the analyses.<br>• Bias in selection of participants into study: Low risk of bias – selection of participants does not appear to be related to both intervention and outcomes; initial follow-up time appears to be similar for all women.<br>• Bias in classification of interventions: Moderate risk of bias – clear definitions provided for 'Patwardhan method' but limited details provided on 'Vaginal push up or Reverse breech extraction'.<br>• Bias due to deviations from intended intervention: Low risk of bias – no deviations from the intended intervention reported.<br>• Bias due to missing data: Unclear risk of bias – no information provided.<br>• Bias on measurement of outcomes: Low risk of bias - outcomes measured using objective measurement tools; comparable outcome detection methods and thresholds used, and same definitions and measurements.<br>• Bias in selection of the reported result: Moderate risk of bias - no mention of pre-registered protocol or statistical analysis plan.<br>Overall risk of bias: Serious risk of bias.
**Source of funding**	Not reported

21. Saleh 2014

**Study details**	**Full citation:** Saleh H.S., Kassem G.A., Mohamed M.S. et al. Pull breech out versus push impacted head up in emergency caesarean section: a comparative study. Open J Obstet Gynec. 2014;4:260-5<br>**Study type:** Randomised controlled trial<br>**Country of study:** Egypt<br>**Study dates:** April 2011 to May 2012
**Participants**	**Inclusion criteria:** Women in advanced labour with cervical dilatation ≥7 cm, single term pregnancy (according to the date of last normal menstrual period or early first trimester ultrasonography), cephalic presentation, and deeply impacted fetal head in maternal pelvis<br>**Exclusion criteria:** Women with multiple pregnancy, non-cephalic presentation, previous uterine scar, or gestational age <37 weeks<br>**Participant characteristics:**<br>- Vaginal push up group:<br>o N women: 40<br>o Mean (SD) maternal age: 22.7 (4.7) years<br>o Mean (SD) maternal weight: Not reported<br>o Smoking status: Not reported<br>o Mean (SD; range) parity: 2.4 (1.2; 0 to 5)<br>o Diabetes: Not reported<br>o Mean (SD) gestational age: 39.42 (1.1; 37 to 41) weeks<br>o Numbers with caesarean section at full (10cm) cervical dilatation: Not reported (women were required to have cervical dilatation ≥7 cm, as per the inclusion criteria)<br>o Numbers with caesarean prior to (<10cm) full cervical dilatation: Not reported (women were required to have cervical dilatation ≥7 cm, as per the inclusion criteria)<br>- Reverse breech extraction group:<br>o N women: 40<br>o Mean (SD) maternal age: 23.5 (4.6) years<br>o Mean (SD) maternal weight: Not reported<br>o Smoking status: Not reported<br>o Mean (SD; range) parity: 2.2 (1; 0 to 5)<br>o Diabetes: Not reported<br>o Mean (SD; range) gestational age: 39.51 (1.2; 37 to 41) weeks<br>o Numbers with caesarean section at full (10cm) cervical dilatation: Not reported (women were required to have cervical dilatation ≥7 cm, as per the inclusion criteria)<br>o Numbers with caesarean section prior to (<10cm) full cervical dilatation: Not reported (women were required to have cervical dilatation ≥7 cm, as per the inclusion criteria)

**Intervention & comparator**	**Intervention:** 'Vaginal push up': "an assistant was used to push the fetal head vaginally while the surgeon attempts to pass his/her hand below the head to dislodge the head from pelvis" (p. 261).<br><br>**Comparator:** 'Reverse breech extraction': "A high transverse incision was set over stretched lower uterine segment where loose fold of visceral peritoneum is attached, at the level of the anterior shoulder of the fetus as the head is deeply impacted. As soon as the incision was given anterior shoulder came out spontaneously or it was gently pulled out of the uterus as space was created for the surgeon to enter his/her hand or two fingers inside the uterine cavity and examine for the anterior foot. Once the foot was grasped, traction was applied on the foot with fundal pressure to bring the foot and subsequently the whole lower limb, the other limb, trunk and head outside the cavity" (p. 261).
**Primary outcomes and results**	**Maternal:**<br>• Uterine incision extension (angle extensions on lower segments and / or into broad ligaments):<br>o Incision extension on lower segment (at angles or towards cervix): Study reported unintended extension of uterine incision beyond normal limits: 'Vaginal push up': 20/40; 'Reverse breech extraction': 8/40 (p=0.001)<br>o Angle extensions into broad ligaments: Not reported<br>• Operative blood loss / postpartum haemorrhage (operative blood loss > 500ml): Study reported operative blood loss (ml): 'Vaginal push up': Mean (SD) = 1321 (572) ml; 'Reverse breech extraction': Mean (SD) = 878 (674) ml (p=0.001); Postpartum haemorrhage: 'Vaginal push up': 5/40; 'Reverse breech extraction': 2/40 (p=0.06)<br>• Operative time (duration of surgery): 'Vaginal push up': Mean (SD) = 75.2 (6.1) minutes; 'Reverse breech extraction': Mean (SD) = 59.7 (4.2) minutes (p=0.001)<br>**Perinatal:**<br>• Infant birth trauma (skull fracture / intracranial haemorrhage / other bony fracture / nerve injury): Not reported<br>• Apgar score at five minutes / Apgar score < 7 at five minutes: The authors defined this outcome as "Apgar score five minutes" and then reported "'Vaginal push up': 9/40; 'Reverse breech extraction': 5/40 (p=0.312)". We have not included these data in the analyses as these data are reported as dichotomous data (not continuous), but it is not reported what the Apgar score cut-off is.
**Secondary outcomes and results**	**Maternal:**<br>• Blood transfusion: Intraoperative blood transfusion: 'Vaginal push up': 10/40; 'Reverse breech extraction': 2/40 (p=0.02); Postoperative blood transfusion: 'Vaginal push up': 7/40; 'Reverse breech extraction': 4/40 (p=0.07)<br>• Inverted T or J incision: Not reported<br>• Visceral injury (ureteral / bladder / cervical) or hysterectomy:<br>o Uterine incision extension into cervix / vagina: Not reported<br>o Injury to urinary tract (including ureteric injury and bladder injury): Study reported this outcome defined as injury to urinary bladder: 'Vaginal push up': 2/40; 'Reverse breech extraction': 0/40 (p=0.41)<br>o Hysterectomy: Not reported<br>• Infection (wound infection / endometritis / maternal sepsis):

	○ Wound infection: 'Vaginal push up': 2/40; 'Reverse breech extraction': 1/40 (p=0.52)<br>○ Endometritis: Not reported<br>○ Urinary tract infection: Not reported<br>○ Post-partum pyrexia / maternal sepsis: Not reported<br>• Duration of hospital stay: The authors did not explicitly state the unit of measure for this outcome but it was assumed that the outcome refers to days: 'Vaginal push up': Mean (SD) = 3.8 (1.3) days; 'Reverse breech extraction': Mean (SD) = 3.2 (1.4) days (p=0.72)<br>• Decision-to-delivery interval: Not reported<br>• Incision-to-delivery interval: Not reported<br>**Perinatal:**<br>• NICU (Neonatal Intensive Care Unit) admission: ' 'Vaginal push up': 7/40; Reverse breech extraction': 4/40 (p=0.176)<br>• Umbilical artery pH: Not reported<br>• Neonatal death (defined as death within the first 28 days of life): Not reported<br>Cost: Not reported
**Risk of bias**	**Assessed by RoB2:**<br>• Risk of bias arising from the randomisation process: Some concerns – women were randomly assigned on a 1:1 basis and randomisation started by rotary, no other details provided.<br>• Risk of bias due to deviations from the intended interventions (effect of assignment to intervention): Low risk of bias – no mention of blinding; no deviations from intended intervention.<br>• Risk of bias due to missing outcome data: Low risk of bias – no reported missing outcome data.<br>• Risk of bias in measurement of the outcome: Low risk of bias – outcomes measured using objective measurement tools; comparable outcome detection methods and thresholds used, and same definitions and measurements.<br>• Risk of bias in selection of the reported result: High risk of bias – no details provided, and published in a potential predatory journal according to Beall's List of Potential Predatory Journals and Publishers.<br>Overall risk of bias: High risk of bias.
**Source of funding**	Not reported

22. Seal 2014

**Study details**	**Full citation:** Seal S.L., Dey A., Barman S.C., Kamilya G., Mukherji J. Does elevating the fetal head prior to delivery using a fetal pillow reduce maternal and fetal complications in a full dilatation caesarean section? A prospective study with historical controls. J Obstet Gynaecol 2014;34:241–4<br>**Study type:** Non-randomised comparative prospective cohort with unmatched historical controls<br>**Country of study:** India

	**Study dates**: October 2011 to February 2012
**Participants**	**Inclusion criteria:** Women with single pregnancy at full dilation requiring caesarean section, including women with failed attempt at instrumental delivery (fetal pillow group); “The unmatched historical controls were 124 patients who underwent a second stage CS from our previously reported study of second vs first stage caesarean delivery” (p. 242; no fetal pillow)<br>**Exclusion criteria:** Women with active genital infection; major fetal abnormalities; pregnancy induced hypertension; intrauterine growth restriction; diabetes<br>**Participant characteristics:**<br>- Fetal pillow group:<br>o N women: 50<br>o Mean (SD) maternal age: 23.1 (3.7)<br>o Mean (SD) maternal weight: 55.7 (8.7) kg<br>o Smoking status: Not reported<br>o Parity: Not reported<br>o Diabetes: 0, as per exclusion criteria<br>o Mean (SD) gestational age: 39.1 (1) weeks<br>o Numbers with caesarean section at full (10cm) cervical dilatation: 50, all women at full dilation as per inclusion criteria<br>o Numbers with caesarean section prior to (<10cm) full cervical dilatation: 0<br>- No fetal pillow group:<br>o N women: 124<br>o Mean (SD) maternal age: 24.2 (3.1)<br>o Mean (SD) maternal weight: 55.6 (9.3) kg<br>o Smoking status: Not reported<br>o Parity: Not reported<br>o Diabetes: 0, as per exclusion criteria<br>o Mean (SD) gestational age: 39.4 (0.88) weeks<br>o Numbers with caesarean section at full (10cm) cervical dilatation: 124, all women at full dilation as per inclusion criteria<br>o Numbers with caesarean prior to (<10cm) full cervical dilatation: 0
**Intervention & comparator**	**Intervention:** ‘Fetal pillow’: “A fetal pillow is a device designed to elevate the fetal head atraumatically prior to a CSFD. It is a soft silicone balloon that is inserted vaginally prior to performing a CSFD. After insertion, the balloon is inflated with 180 ml of sterile saline via a tube connected to a two-way tap. The balloon inflates only in an upward direction; the base plate rests on the least distensible part of pelvic floor, the anococcygeal ligament, and it is designed so that it does not change position during inflation… The device is deflated as soon as the delivery is achieved, by opening the two-way tap and it is removed at the end of the procedure by gently pulling at the tubing” (p. 241-242).<br>**Comparator:** “No fetal pillow’: “In our previous study, the main method of delivery when there was severe difficulty encountered was by pushing from below in half of the patients and a reverse breech extraction technique in

	the others (Seal et al. 2010)" (p. 243).
**Primary outcomes and results**	**Maternal:**<br>○ Uterine incision extension (angle extensions on lower segments and / or into broad ligaments):<br>○ Incision extension on lower segment (at angles or towards cervix): Not reported<br>○ Angle extensions into broad ligaments: Not reported<br>○ Operative blood loss / postpartum haemorrhage (operative blood loss > 500ml): Study reported blood loss > 1000 ml: 'Fetal pillow': 1/50; 'No fetal pillow': 10/124 (p=0.18)<br>○ Operative time (duration of surgery): Study reported total caesarean time: 'Fetal pillow': Mean (SD) = 31.8 (4.6) minutes; 'No fetal pillow': Mean (SD) = 52.1 (10.7) minutes (p<0.001)<br>**Perinatal:**<br>○ Infant birth trauma (skull fracture / intracranial haemorrhage / other bony fracture / nerve injury): Study reported neonatal injury: 'Fetal pillow': 0/50; 'No fetal pillow': 6/124 (p=0.12)<br>○ Apgar score at five minutes / Apgar score < 7 at five minutes: Study reported Apgar score < 3 at five minutes: 'Fetal pillow': 2/50; 'No fetal pillow': 4/124 (p=0.55)
**Secondary outcomes and results**	**Maternal:**<br>• Blood transfusion: 'Fetal pillow': 1/50; 'No fetal pillow': 6/124 (p=0.36)<br>• Inverted T or J incision: Not reported<br>• Visceral injury (ureteral / bladder / cervical) or hysterectomy:<br>○ Uterine incision extension into cervix / vagina: Study reported uterine extension Grades II (extension that increases the operating time and blood loss) and III (extension that involves one or both uterine arteries, cervix, vagina or other organs): 'Fetal pillow': 2/50; 'No fetal pillow': 19/124 (p=0.03)<br>○ Injury to urinary tract (including ureteric injury and bladder injury): Not reported (although the study did report second degree tear during device insertion: 'Fetal pillow': 1/50; 'No fetal pillow':0/124)<br>○ Hysterectomy: Not reported<br>• Infection (wound infection / endometritis / maternal sepsis):<br>○ Wound infection: Not reported<br>○ Endometritis: Not reported<br>○ Urinary tract infection: Not reported<br>○ Post-partum pyrexia / maternal sepsis: Not reported<br>• Duration of hospital stay: 'Fetal pillow': Mean (SD not reported) = 4.1 days; 'No fetal pillow': Mean (SD not reported) = 6.4 days (p<0.001)<br>• Decision-to-delivery interval: Not reported<br>• Incision-to-delivery interval: 'Fetal pillow': Mean (SD) = 2.79 (0.4) minutes; 'No fetal pillow': Mean (SD) = 8.43 (1.7) minutes (p<0.001)<br>**Perinatal:**<br>• NICU (Neonatal Intensive Care Unit) admission: 'Fetal pillow': 3/50; 'No fetal pillow': 12/124 (p=0.33)

	• Umbilical artery pH / cord pH < 7.10: Not reported<br>• Neonatal death: Study did not further define this outcome: ‘Fetal pillow’: 0/50; ‘No fetal pillow’: 2/124 (p=0.51)<br>Cost: Not reported
**Risk of bias**	**Assessed by ROBINS-I:**<br>• Bias due to confounding: Serious risk of bias – all clinicians were instructed in the use of the device by observing a training animation video; historical controls were unmatched and the authors did not adjust for confounding in any of the analyses.<br>• Bias in selection of participants into study: No information – no information provided on unmatched controls.<br>• Bias in classification of interventions: Moderate risk of bias – clear definitions provided for ‘Fetal pillow’ group but limited details provided on the interventions used for the unmatched historical controls.<br>• Bias due to deviations from intended intervention: Low risk of bias – no deviations from the intended intervention reported.<br>• Bias due to missing data: Unclear risk of bias – no information provided.<br>• Bias on measurement of outcomes: Low risk of bias - outcomes measured using objective measurement tools; comparable outcome detection methods and thresholds used, and same definitions and measurements.<br>• Bias in selection of the reported result: Critical risk of bias - no mention of pre-registered protocol or statistical analysis plan, and published with authors and using data similar to that of a recently retracted RCT.<br>Overall risk of bias: Critical risk of bias.
**Source of funding**	The authors reported that the cephalic elevation devices were provided by Safe Obstetrics Systems, a medical device company acquired in 2021 by CooperCompanies, a global medical device company.

23. Tahir 2020

**Study details**	**Full citation:** Tahir N, Shahid G, Adil M, Fatima S. Reverse Breech Extraction Vs Head Pushing For Delivery Of Deeply Impacted Fetal Head In Emergency Caesarean Section. Journal of Ayub Medical College, Abbottabad : JAMC. 2020;32(4):497-501<br>**Study type:** Randomised controlled study<br>**Country of study:** Pakistan<br>**Study dates**: May to November 2014
**Participants**	**Inclusion criteria:** Women who developed obstructed labour with a single pregnancy in cephalic presentation, at least 37 weeks of gestation (calculated by the date of the last menstrual period or ultrasonography performed in early first trimester) and requiring caesarean section<br>**Exclusion criteria:** Women with multiple pregnancy, non-cephalic presentation, previous caesarean section scar, or pre-term labour<br>**Participant characteristics:**<br>- 'Vaginal push up' group:<br>o N women: 55<br>o Mean (SD) maternal age: 27.91 (6.85) years (15 to 25 years: 26/55; 26 to 35 years: 22/55; 36 to 45 years: 7/55)<br>o Maternal BMI: Not reported<br>o Smoking status: Not reported<br>o Parity: Primigravida: 26 (47.3%); Multigravida: 29 (52.7%)<br>o Diabetes: Not reported<br>o Mean (SD) gestational age: 40.05 (0.62) weeks (38 to 39 weeks: 7/55; 40 to 41 weeks: 48/55)<br>o Numbers with caesarean section at full (10cm) cervical dilatation: Not reported<br>o Numbers with caesarean section prior to (<10cm) full cervical dilatation: Not reported<br>- 'Reverse breech extraction' group:<br>o N women: 55<br>o Mean (SD) maternal age: 27.51 (6.6) years (15 to 25 years: 24/55; 26 to 35 years: 25/55; 36 to 45 years: 6/55)<br>o Maternal BMI: Not reported<br>o Smoking status: Not reported<br>o Parity: Primigravida: 27 (49.1%); Multigravida: 28 (50.9%)<br>o Diabetes: Not reported<br>o Mean (SD) gestational age: 39.93 (0.87) weeks (38 to 39 weeks: 19/55; 40 to 41 weeks: 36/55)<br>o Numbers with caesarean section at full (10cm) cervical dilatation: Not reported<br>o Numbers with caesarean prior to (<10cm) full cervical dilatation: Not reported

**Intervention & comparator**	**Intervention:** ‘Vaginal push up’: “An assistant pushed the foetal head vaginally while the surgeon tried to dislodge the head from the pelvis by passing a hand below the head” (p. 498).<br><br>**Comparator:** ‘Reverse breech extraction’: “Reverse breech extraction was performed by giving a high transverse incision over the lower stretched uterine segment where loose fold of visceral peritoneum is attached, at the level of the anterior shoulder of the baby” (p. 498).<br><br>“A single dose of Ceftriaxone 1gm IV was given prophylactically to all women before the incision. All caesarean deliveries were performed under spinal anaesthesia” (p. 498).
**Primary outcomes and results**	**Maternal:**<br>• Uterine incision extension (angle extensions on lower segments and / or into broad ligaments):<br>○ Incision extension on lower segment (at angles or towards cervix): ‘Vaginal push up’: 25/55; ‘Reverse breech extraction’: 5/55 ($p<0.001$)<br>○ Angle extensions into broad ligaments: Not reported<br>• Operative blood loss / post-partum haemorrhage (operative blood loss > 500ml): Study reported operative blood loss (ml): ‘Vaginal push up’: Mean (SD) = 1542.36 (188.27) ml; ‘Reverse breech extraction’: Mean (SD) = 1090 (130.08) ml ($p<0.001$)<br>• Operative time (duration of surgery): ‘Vaginal push up’: Mean (SD) = 51.73 (2.14) minutes; ‘Reverse breech extraction’: Mean (SD) = 42.47 (3) minutes ($p<0.001$)<br>**Perinatal:**<br>• Infant birth trauma (skull fracture / intracranial haemorrhage / other bony fracture / nerve injury): Not reported<br>• Apgar score at five minutes / Apgar score < 7 at five minutes: Not reported
**Secondary outcomes and results**	**Maternal:**<br>• Blood transfusion: ‘Not reported<br>• Inverted T or J incision: Not reported<br>• Visceral injury (ureteral / bladder / cervical) or hysterectomy:<br>○ Uterine incision extension into cervix / vagina: Not reported<br>○ Injury to urinary tract (including ureteric injury and bladder injury): Not reported<br>○ Hysterectomy: Not reported<br>• Infection (wound infection / endometritis / maternal sepsis):<br>○ Wound infection: Not reported<br>○ Endometritis: Not reported<br>○ Urinary tract infection: Not reported<br>○ Post-partum pyrexia / maternal sepsis: Not reported<br>• Duration of hospital stay: Not reported<br>• Decision-to-delivery interval: Not reported

	• Incision-to-delivery interval: Not reported<br>**Perinatal:**<br>• NICU (Neonatal Intensive Care Unit) admission: Not reported<br>• Umbilical artery pH / cord pH < 7.10: Not reported<br>• Neonatal death (defined as death within the first 28 days of life): Not reported<br>Cost: Not reported
**Risk of bias**	**Assessed by RoB2:**<br>• Risk of bias arising from the randomisation process: Some concerns – the authors reported that women were randomised using block randomisation methods.<br>• Risk of bias due to deviations from the intended interventions (effect of assignment to intervention): Low risk of bias – no information provided relating to blinding to treatment assignment; no deviations from intended intervention.<br>• Risk of bias due to missing outcome data: Low risk of bias – no reported missing outcome data.<br>• Risk of bias in measurement of the outcome: Low risk of bias – outcomes measured using objective measurement tools; comparable outcome detection methods and thresholds used, and same definitions and measurements.<br>• Risk of bias in selection of the reported result: Some concerns - outcome data recorded using a pre-designed proforma, but no further details provided.<br>Overall risk of bias: Some concerns.
**Source of funding**	Not reported

24. Veisi 2012

**Study details**	**Full citation:** Veisi F., Zangeneh M., Malekkhosravi S., Rezavand N. Comparison of “push” and “pull” methods for impacted fetal head extraction during cesarean delivery. International Journal of Gynecology & Obstetrics. 2012 Jul 1;118(1):4–6<br>**Study type:** Randomised controlled trial<br>**Country of study:** Iran<br>**Study dates:** April 2006 to March 2008
**Participants**	**Inclusion criteria:** Women with confirmed single, term pregnancy (37 to 42 weeks) in cephalic presentation, and reactive fetal heart rate pattern. Women had obstructed dystocia with full cervical dilation and fetal head impacted in the pelvis<br>**Exclusion criteria:** Women with multiple pregnancy; estimated fetal weight >4000 g; intrauterine fetal death; previous caesarean or myomectomy, chorioamnionitis; or third-trimester haemorrhage<br>**Participant characteristics:**<br>- Vaginal push up group:<br>o N women: 35<br>o Mean (SD) maternal age: 24.05 (3.42) years<br>o Maternal BMI: Not reported<br>o Smoking status: Not reported<br>o Mean (SD) , range parity: 1.42 (0.92), 0 to 5<br>o Diabetes: Not reported<br>o Mean (SD) gestational age: 38.68 (1.31) weeks<br>o Numbers with caesarean section at full (10cm) cervical dilatation: Not explicitly stated, but presumably 37 as per the inclusion criteria stating full cervical dilatation<br>o Numbers with caesarean prior to (<10cm) full cervical dilatation: Not explicitly stated, but presumably 0<br>- Reverse breech extraction group:<br>o N women: 37<br>o Mean (SD) maternal age: 23.46 (3.73) years<br>o Maternal BMI: Not reported<br>o Smoking status: Not reported<br>o Mean (SD), range parity: 1.54 (0.83), 0 to 4<br>o Diabetes: Not reported<br>o Mean (SD) gestational age: 38.94 (1.29) weeks<br>o Numbers with caesarean section at full (10cm) cervical dilatation: Not explicitly stated, but presumably 35 as per the inclusion criteria stating full cervical dilatation<br>o Numbers with caesarean section prior to (<10cm) full cervical dilatation: Not explicitly stated, but presumably 0

**Intervention & comparator**	**Intervention:** 'Vaginal push up': "In the push method, after opening into the uterus, the patient's knees were flexed and her legs abducted. An assistant then introduced their hand into the patient's vagina and exerted pressure to dislodge the fetal head and elevate it toward the surgeon's hand. The surgeon then delivered the fetus via routine cesarean" (p. 5).<br><br>**Comparator:** 'Reverse breech extraction': "In the pull method, after opening into the uterus, the surgeon introduced their hand through the uterine incision toward the upper segment, grasped both fetal legs, and gently pulled the fetus up to extract it" (p. 5).<br><br>All caesarean sections were undertaken by the same obstetric chief resident under the direct supervision of one of the co-authors. All women received antibiotic prophylaxis (1 g intravenous cefazolin every 8 hours) after delivery and clamping of the umbilical cord. Women without complications were discharged on the third or fourth post-operative day.
**Primary outcomes and results**	**Maternal:**<br>• Uterine incision extension (angle extensions on lower segments and / or into broad ligaments):<br>○ Incision extension on lower segment (at angles or towards cervix): 'Vaginal push up': 24/35; 'Reverse breech extraction': 3/37 ($p<0.05$)<br>○ Angle extensions into broad ligaments: Not reported<br>• Operative blood loss / post-partum haemorrhage (operative blood loss > 500ml): Study reported operative blood loss: 'Vaginal push up': Mean (SD) = 571 (106) ml; 'Reverse breech extraction': Mean (SD) = 457 (68) ml ($p<0.05$)<br>• Operative time (duration of surgery): 'Vaginal push up': Mean (SD) = 45.29 (8.74) minutes; 'Reverse breech extraction': Mean (SD) = 33.38 (6.77) minutes ($p<0.05$)<br>**Perinatal:**<br>• Infant birth trauma (skull fracture / intracranial haemorrhage / other bony fracture / nerve injury): 'Vaginal push up': 0/35; 'Reverse breech extraction': 1/37 (femoral fracture)<br>• Apgar score at five minutes / Apgar score < 7 at five minutes: Study reported Apgar score at five minutes: 'Vaginal push up': Mean (SD) 9.54 (0.63); 'Reverse breech extraction': Mean (SD) = 9.51 (0.69) ($p=0.949$)
**Secondary outcomes and results**	**Maternal:**<br>• Blood transfusion: Not reported<br>• Inverted T or J incision: Not reported<br>• Visceral injury (ureteral / bladder / cervical) or hysterectomy:<br>○ Uterine incision extension into cervix / vagina: Not reported<br>○ Injury to urinary tract (including ureteric injury and bladder injury): Study reported bladder injury: 'Vaginal push up': 0/35; 'Reverse breech extraction': 0/37<br>○ Hysterectomy: Not reported<br>• Infection (wound infection / endometritis / maternal sepsis):<br>○ Wound infection: Study reported wound complication: 'Vaginal push up': 1/35; 'Reverse breech extraction': 1/37

	○ Endometritis: Not reported<br>○ Urinary tract infection: Not reported<br>○ Post-partum pyrexia / maternal sepsis: Study reported postpartum fever: ‘Vaginal push up’: 3/35 ; ‘Reverse breech extraction’: 3/37 (p=0.943)<br>• Duration of hospital stay: Not reported<br>• Decision-to-delivery interval: Not reported<br>• Incision-to-delivery interval: Not reported<br>**Perinatal:**<br>• NICU (Neonatal Intensive Care Unit) admission: ‘Vaginal push up’: 0/35; ‘Reverse breech extraction’: 0/37<br>• Umbilical artery pH / cord pH < 7.10: Not reported<br>• Neonatal death (defined as death within the first 28 days of life): Not reported<br>Cost: Not reported
**Risk of bias**	**Assessed by RoB2:**<br>• Risk of bias arising from the randomisation process: Some concerns – the authors state that women were randomised on a 1:1 ratio, but no other details provided; similar baseline characteristics between intervention arms.<br>• Risk of bias due to deviations from the intended interventions (effect of assignment to intervention): Low risk of bias – blinding not mentioned; no deviations from intended intervention.<br>• Risk of bias due to missing outcome data: Low risk of bias – no reported missing outcome data.<br>• Risk of bias in measurement of the outcome: Low risk of bias – outcomes measured using objective measurement tools; comparable outcome detection methods and thresholds used, and same definitions and measurements.<br>• Risk of bias in selection of the reported result: Some concerns - no information provided.<br>Overall risk of bias: Some concerns.
**Source of funding**	Not reported

Abbreviations

BMI: body mass index; CDFD: caesarean delivery at full dilatation; CI: confidence interval; CS: caesarean section; CSFD: caesarean section at full cervical dilatation; FDCS: caesarean section at full cervical dilatation; MD: mean difference; NICU: Neonatal Intensive Care Unit; NS: non-significant; OR: odds ratio; RD: risk difference; RoB2: Risk of Bias version 2; ROBINS-I: Risk of Bias in Non-Randomised Studies – of Interventions; RR: risk ratio;
