# Supplementary material for: Management of impacted fetal head at cesarean birth: A systematic review and meta‐analysis
Source: Acta Obstet Gynecol Scand. 2024 May 24;103(9):1702–13. doi: 10.1111/aogs.14873 (PMC11324922; doi:10.1111/aogs.14873)
Supplement: Supplementary file 6 — Table S3. [file AOGS-103-1702-s008.pdf]

Table S3: Risk of bias assessment for individual studies

Risk of bias assessed using RoB2 scores (i.e. low risk of bias, some concerns, high risk of bias), and for non-randomised studies assessed using ROBINS-I scores (i.e. low risk of bias, moderate risk of bias, serious risk of bias, critical risk of bias).[1, 2]

See **Table S2** for details on assessment for each individual study

**Author & year**	**RoB2 (RCTs)**						**ROBINS-I (non-randomised studies)**							
	Randomisation process	***Deviations from the intended interventions***	***Missing outcome data***	***Measurement of the outcome***	***Selection of the reported result***	***OVERALL RISK OF BIAS***	***Bias due to confounding***	***Bias in selection of participants into the study***	***Bias in classification of interventions***	***Bias due to deviations from intended interventions***	***Bias due to missing data***	***Bias in measurement of outcomes***	***Bias in selection of the reported result***	***OVERALL RISK OF BIAS***
**Vaginal disimpaction vs reverse breech extraction**														
Bastani 2012	Some	Low	Low	Low	Some	**Some**	-	-	-	-	-	-	-	-
Fasubaa 2002	Low	Low	Low	Low	Some	**Some**	-	-	-	-	-	-	-	-
Frass 2011	Some	Low	Low	Low	Some	**Some**	-	-	-	-	-	-	-	-
Javed 2022	Low	Low	Low	Low	Some	**Some**	-	-	-	-	-	-	-	-
Nooh 2017	Low	Low	Low	Low	Some	**Some**	-	-	-	-	-	-	-	-
Saleh 2014	Some	Low	Low	Low	High	**High**	-	-	-	-	-	-	-	-
Tahir 2020	Some	Low	Low	Low	Some	**Some**	-	-	-	-	-	-	-	-
Veisi 2012	Some	Low	Low	Low	Some	**Some**	-	-	-	-	-	-	-	-

Author & year	RoB2 (RCTs)						ROBINS-I (non-randomised studies)							
	Randomisation process	*Deviations from the intended interventions*	*Missing outcome data*	*Measurement of the outcome*	*Selection of the reported result*	*OVERALL RISK OF BIAS*	*Bias due to confounding*	*Bias in selection of participants into the study*	*Bias in classification of interventions*	*Bias due to deviations from intended interventions*	*Bias due to missing data*	*Bias in measurement of outcomes*	*Bias in selection of the reported result*	*OVERALL RISK OF BIAS*
**Vaginal disimpaction vs Patwardhan**														
Beeresh 2016	-	-	-	-	-	-	Serious	Serious	Low	Unclear	Unclear	Moderate	Critical	**Critical**
Bhattacharya 2020	-	-	-	-	-	-	Serious	Serious	Low	Unclear	Unclear	Moderate	Critical	**Critical**
Keepanasseril 2019	-	-	-	-	-	-	Serious	Low	Low	Low	Low	Low	Moderate	**Serious**
Lal 2018	-	-	-	-	-	-	Serious	Serious	Low	Unclear	Unclear	Moderate	Critical	**Critical**
Lenz 2019	-	-	-	-	-	-	Serious	Low	Low	Low	Unclear	Low	Moderate	**Serious**
Rakholia 2019	-	-	-	-	-	-	Serious	Serious	Low	Unclear	Unclear	Moderate	Critical	**Critical**
**Vaginal disimpaction or reverse breech extraction vs Patwardhan**														
Bansiwal 2017	-	-	-	-	-	-	Serious	Low	Moderate	Low	Low	Unclear	Critical	**Critical**
Bhoi 2019	High	Low	Low	Low	Some	**High**	-	-	-	-	-	-	-	-
Saha 2014	-	-	-	-	-	-	Serious	Low	Moderate	Low	Unclear	Low	Moderate	**Serious**
**Fetal Pillow® vs no pillow**														
Chooi 2022	-	-	-	-	-	-	Serious	Moderate	Moderate	Low	Low	Moderate	Moderate	**Serious**
Hanley 2020	-	-	-	-	-	-	Serious	Moderate	Moderate	Low	Serious	Low	Moderate	**Serious**

**Author & year**	**RoB2 (RCTs)**						**ROBINS-I (non-randomised studies)**							
	Randomisation process	***Deviations from the intended interventions***	***Missing outcome data***	***Measurement of the outcome***	***Selection of the reported result***	***OVERALL RISK OF BIAS***	***Bias due to confounding***	***Bias in selection of participants into the study***	***Bias in classification of interventions***	***Bias due to deviations from intended interventions***	***Bias due to missing data***	***Bias in measurement of outcomes***	***Bias in selection of the reported result***	***OVERALL RISK OF BIAS***
Sacre 2021	-	-	-	-	-	-	Serious	Low	Moderate	Low	Low	Low	Moderate	**Serious**
Seal 2014	-	-	-	-	-	-	Serious	Unclear	Moderate	Low	Unclear	Low	Critical	**Critical**
**Inflated Fetal Pillow® vs non-inflated pillow**														
Lassey 2020	Low	Low	Low	Low	Low	**Low**	-	-	-	-	-	-	-	-
**Fetal Pillow® vs vaginal disimpaction**														
Safa 2016	-	-	-	-	-	-	Serious	Low	Moderate	Low	Low	Low	Moderate	**Serious**
**Fetal Pillow® vs Patwardhan**														
Dutta 2019	Low	Low	Low	Low	High	**High**	-	-	-	-	-	-	-	-

1. Sterne JAC, Savović J, Page MJ, Elbers RG, Blencowe NS, Boutron I, et al. RoB 2: a revised tool for assessing risk of bias in randomised trials. BMJ. 2019:l4898.
2. Sterne JA, Hernán MA, Reeves BC, Savović J, Berkman ND, Viswanathan M, et al. ROBINS-I: a tool for assessing risk of bias in non-randomised studies of interventions. bmj. 2016;355.
