# Supplementary material for: Management of impacted fetal head at cesarean birth: A systematic review and meta‐analysis
Source: Acta Obstet Gynecol Scand. 2024 May 24;103(9):1702–13. doi: 10.1111/aogs.14873 (PMC11324922; doi:10.1111/aogs.14873)
Supplement: Supplementary file 7 — Table S4. [file AOGS-103-1702-s001.pdf]

Table S4: GRADE assessment of certainty of evidence for each outcome in the seven different comparisons identified across the 24 eligible studies

Table of contents

1. GRADE summary of findings table for vaginal disimpaction vs reverse breech extraction (**page 2**)
2. GRADE summary of findings table for vaginal disimpaction vs Patwardhan method (**page 7**)
3. GRADE summary of findings table for vaginal disimpaction or reverse breech extraction vs Patwardhan method (**page 10**)
4. GRADE summary of findings table for Fetal Pillow® vs no pillow (**page 11**)
5. GRADE summary of findings table for inflated Fetal Pillow vs non-inflated Fetal Pillow (**page 14**)
6. GRADE summary of findings table for Fetal Pillow® vs vaginal disimpaction (**page 16**)
7. GRADE summary of findings table for Fetal Pillow® vs Patwardhan method (**page 17**)

1. GRADE summary of findings table for vaginal disimpaction vs reverse breech extraction

Quality assessment							No of patients		Effect		Certainty
No of studies	Design	Risk of bias	Inconsistency	Indirectness	Imprecision[c]	Publication bias	Vaginal push up	Reverse breech extraction	Relative risk (95% CI)	Absolute	
**Uterine incision extension: Combined – RCTs**											
8	Randomised trials (Bastani 2012, Fasubaa 2002, Frass 2011, Nooh 2017, Saleh 2014, Tahir 2020, Veisi 2012, Javed 2022)	Very serious[a]	No serious inconsistency[b] (I^2=17%)	No serious indirectness	Serious[c]	Yes*	189/412 (45.9%)	53/413 (12.8%)	RR 3.41 (2.50 to 4.66)	309 more per 1,000 (from 192 more to 470 more)	V LOW
**Uterine incision extension: Incision extension on lower segment – RCTs**											
6	Randomised trials (Frass 2011, Nooh 2017, Saleh 2014, Tahir 2020, Veisi 2012, Javed 2022)	Very serious[a]	Serious[b] (I^2=39%)	No serious indirectness	Serious[c]	Yes*	158/328 (48.2%)	42/330 (12.7%)	RR 3.82 (2.51 to 5.82)	359 more per 1000 (from 192 more to 613 more)	VERY LOW
**Uterine incision extension: Angle extensions into broad ligaments – RCTs**											
2	Randomised trials (Bastani 2012, Fasubaa 2002)	Serious[a]	No serious inconsistency[b] (I^2=0%)	No serious indirectness	Very serious[c]	None	31/84 (36.9%)	11/83 (13.3%)	RR 2.78 (1.51 to 5.13)	236 more per 1000 (from 68 more to 547 more)	VERY LOW
**Maternal operative blood loss (ml) - RCTs (Better indicated by lower values)**											
7	Randomised trials (Fasubaa 2002, Frass 2011, Nooh 2017, Saleh 2014, Tahir 2020, Veisi 2012, Javed 2022)	Very serious[a]	Very serious[b] (I^2=98%)	No serious indirectness	No serious imprecision	Yes*	Fasubaa 2002: 54<br>Frass 2011: 59<br>Nooh 2017: 96<br>Saleh 2014:40<br>Tahir 2020: 55<br>Veisi 2012:35<br>Javed 2022: 43	Fasubaa 2002: 54<br>Frass 2011: 59<br>Nooh 2017: 96<br>Saleh 2014:40<br>Tahir 2020: 55<br>Veisi 2012:37<br>Javed 2022: 43	Not applicable	Fasubaa 2002: MD 358.1 higher (340.84 to 375.36 higher)<br>Frass 2011: MD 444 higher (265.17 to 622.83 higher)<br>Nooh 2017: MD 535 higher (456.88 to 613.12 higher)<br>Saleh 2014: MD 443 higher (169.05 to 716.95 higher)<br>Tahir 2020: MD 452.36 higher (391.88 to 512.84 higher)<br>Veisi 2012: MD 114 higher (72.61 to 155.39 higher)<br>Javed 2022: MD 77 (40.77 to 113.23 higher)	VERY LOW

Quality assessment							No of patients		Effect		Certainty
No of studies	Design	Risk of bias	Inconsistency	Indirectness	Imprecision[c]	Publication bias	Vaginal push up	Reverse breech extraction	Relative risk (95% CI)	Absolute	
**Post-partum haemorrhage – RCTs**											
3	Randomised trials (Frass 2011, Nooh 2017, Saleh 2014)	Very serious[a]	No serious inconsistency[b] (I^2=0%)	No serious indirectness	Very serious[c]	Yes*	20/195 (10.3%)	9/195 (4.6%)	RR 2.21 (1.04 to 4.69)	56 more per 1000 (from 2 more to 170 more)	VERY LOW
**Operative time (duration of surgery) - minutes RCTs (Better indicated by lower values)**											
8	Randomised trials (Bastani 2012, Fasubaa 2002, Frass 2011, Nooh 2017, Saleh 2014, Tahir 2020, Veisi 2012, Javed 2022)	Very serious[a]	Very serious[b] (I^2=99%)	No serious indirectness	No serious imprecision	Yes*	Bastani 2012: 30<br>Fasubaa 2002: 54<br>Frass 2011: 59<br>Nooh 2017: 96<br>Saleh 2014:40<br>Tahir 2020: 55<br>Veisi 2012:35<br>Javed 2022:43	Bastani 2012: 29<br>Fasubaa 2002: 54<br>Frass 2011: 59<br>Nooh 2017: 96<br>Saleh 2014:40<br>Tahir 2020: 55<br>Veisi 2012: 37<br>Javed 2022:43	Not applicable	Bastani 2012: MD 0.1 higher (5.77 lower to 5.97 higher)<br>Fasubaa 2002: MD 32.9 higher (31.00 to 34.8 higher)<br>Frass 2011: MD 14.3 higher (12.53 to 16.07 higher)<br>Nooh 2017: MD 14.9 higher (13.47 to 16.33 higher)<br>Saleh 2014: MD 15.5 higher (13.2 to 17.8 higher)<br>Tahir 2020: MD 9.26 higher (8.29 to 10.23 higher)<br>Veisi 2012: MD 11.91 higher (8.28 to 15.54 higher)<br>Javed 2022: MD 9.83 higher (6.44 to 13.22 higher)	VERY LOW
**Infant birth trauma – RCTs**											
4	Randomised trials (Bastani 2012, Fasubaa 2002, Veisi 2012, Javed 2022)	Serious[a]	No serious inconsistency[b] (I^2=0%)	No serious indirectness	Very serious[c]	None	3/162 (1.9%)	6/163 (3.7%)	RD -0.02 (-0.05 to 0.02)	19 fewer per 1000 (from 48 fewer to 19 more)	VERY LOW
**Apgar score at five minutes - RCTs (Better indicated by lower values)**											
3	Randomised trials (Bastani 2012, Fasubaa 2002, Veisi 2012)	Serious[a]	Very serious[b] (I^2=98%)	No serious indirectness	Serious[c]	None	Bastani 2012: 30<br>Fasubaa 2002: 54<br>Veisi 2012: 35	Bastani 2012: 29<br>Fasubaa 2002: 54<br>Veisi 2012: 37	Not applicable	Bastani 2012: MD 0.1 higher (0.18 lower to 0.38 higher)	VERY LOW

Quality assessment							No of patients		Effect		Certainty
No of studies	Design	Risk of bias	Inconsistency	Indirectness	Imprecision[c]	Publication bias	Vaginal push up	Reverse breech extraction	Relative risk (95% CI)	Absolute	
										Fasubaa 2002: MD 1.2 lower (1.28 to 1.12 lower) Veisi 2012: MD 0.03 higher (0.27 lower to 0.33 higher)	
**Apgar score <7 at five minutes – RCTs**											
2	Randomised trials (Frass 2011, Nooh 2017)	Serious[a]	Serious[b] (I^2=59%)	No serious indirectness	Serious[c]	None	35/155 (22.6%)	20/155 (12.9%)	RR 1.72 (0.77 to 3.82)	93 more per 1000 (from 30 fewer to 364 more)	VERY LOW
**Maternal blood transfusion – RCTs**											
4	Randomised trials (Bastani 2012, Frass 2011, Nooh 2017, Saleh 2014)	Very serious[a]	No serious inconsistency[b] (I^2=0%)	No serious indirectness	Very serious[c]	Yes*	40/225 (17.8%)	14/224 (6.3%)	RR 2.75 (1.55 to 4.88)	109 more per 1000 (from 34 more to 243 more)	VERY LOW
**Inverted T or J incision – RCTs**											
1	Randomised trials (Frass 2011)	Serious[a]	No serious inconsistency	No serious indirectness	Very serious[c]	None	0/59 (0%)	4/59 (6.8%)	Peto OR 0.13 (0.02 to 0.94)	59 fewer per 1000 (from 4 fewer to 66 fewer)	VERY LOW
**Uterine incision extension into the cervix or vagina – RCTs**											
2	Randomised trials (Bastani 2012, Fasubaa 2002)	Serious[a]	Serious[b] (I^2=68%)	No serious indirectness	Very serious[c]	None	12/84 (14.3%)	6/83 (7.2%)	RR 1.78 (0.29 to 10.85)	56 more per 1000 (from 51 fewer to 712 more)	VERY LOW
**Injury to the urinary tract – RCTs**											
6	Randomised trials (Bastani 2012, Frass 2011, Nooh 2017, Saleh 2014, Veisi 2012, Javed 2022)	Very serious[a]	No serious inconsistency[b] (I^2=0%)	No serious indirectness	Very serious[c]	Yes*	7/303 (2.3%)	2/304 (0.7%)	RD 0.01 (-0.01 to 0.03)	8 more per 1000 (from 8 fewer to 24 more)	VERY LOW
**Hysterectomy – RCTs**											
1	Randomised trials (Nooh 2017)	Serious[a]	No serious inconsistency	No serious indirectness	Very serious[c]	None	1/96 (1%)	0/96 (0%)	Peto OR 7.39 (0.15 to 372.38)	10 more per 1000 (from 18 fewer to 39 more)[d]	VERY LOW
**Wound infection – RCTs**											
7	Randomised trials (Bastani 2012, Fasubaa 2002, Frass 2011, Nooh 2017, Saleh 2014, Veisi 2012, Javed 2022)	Very serious[a]	No serious inconsistency[b] (I^2=28%)	No serious indirectness	Very serious[c]	Yes*	40/357 (11.2%)	27/358 (7.5%)	RR 1.62 (0.81 to 3.23)	47 more per 1000 (from 14 fewer to 168 more)	VERY LOW
**Endometritis – RCTs**											
2	Randomised trials (Fasubaa 2002, Frass 2011)	Serious[a]	No serious inconsistency[b] (I^2=0%)	No serious indirectness	Very serious[c]	None	39/113 (34.5%)	26/113 (23%)	RR 1.54 (1.04 to 2.27)	124 more per 1000 (from 9 more to 292 more)	VERY LOW

Quality assessment							No of patients		Effect		Certainty
No of studies	Design	Risk of bias	Inconsistency	Indirectness	Imprecision[c]	Publication bias	Vaginal push up	Reverse breech extraction	Relative risk (95% CI)	Absolute	
**Urinary tract infection – RCTs**											
1	Randomised trials (Bastani 2012)	Serious[a]	No serious inconsistency	No serious indirectness	Very serious[c]	None	10/30 (33.3%)	0/29 (0%)	Peto OR 10.26 (2.66 to 39.52)	333 more per 1000 (from 161 more to 506 more)[d]	VERY LOW
**Post-partum pyrexia / maternal sepsis – RCTs**											
4	Randomised trials (Bastani 2012, Nooh 2017, Veisi 2012, Javed 2022)	Serious[a]	No serious inconsistency[b] (I^2=39%)	No serious indirectness	Very serious[c]	None	43/204 (21.1%)	12/205 (5.9%)	RR 3.06 (1.34 to 6.98)	121 more per 1000 (from 20 more to 350 more)	VERY LOW
**Maternal duration of hospital stay - days RCTs (Better indicated by lower values)**											
5	Randomised trials (Bastani 2012, Fasubaa 2002, Frass 2011, Nooh 2017, Saleh 2004)	Very serious[a]	Very serious[b] (I^2=94%)	No serious indirectness	No serious imprecision	Yes*	Bastani 2012: 30<br>Fasubaa 2002: 54<br>Frass 2011: 59<br>Nooh 2017: 96<br>Saleh 2014:40	Bastani 2012: 29<br>Fasubaa 2002: 54<br>Frass 2011: 59<br>Nooh 2017: 96<br>Saleh 2014:40	Not applicable	Bastani 2012: MD 0.1 lower (0.41 lower to 0.21 higher)<br>Fasubaa 2002: MD 3.1 higher (2.36 to 3.84 higher)<br>Frass 2011: MD 0.5 higher (0.03 lower to 1.03 higher)<br>Nooh 2017: MD 0.8 higher (0.42 to 1.18 higher)<br>Saleh 2014: MD 0.6 (0.01 to 1.19 higher)	VERY LOW
**NICU admission – RCTs**											
7	Randomised trials (Bastani 2012, Fasubaa 2002, Frass 2011, Nooh 2017, Saleh 2004, Veisi 2012, Javed 2022)	Very serious[a]	Very serious[b] (I^2=90%)	No serious indirectness	Very serious[c]	Yes*	Bastani 2012: 0/30 (0%)<br>Fasubaa 2002: 39/54 (72.2%)<br>Frass 2011: 13/59 (22.0%)<br>Nooh 2017: 15/96 (15.6%)<br>Saleh 2014: 7/40 (17.5%)<br>Veisi 2012: 0/35 (0%)	Bastani 2012: 0/29 (0%)<br>Fasubaa 2002: 14/54 (25.9%)<br>Frass 2011: 11/59 (18.6%)<br>Nooh 2017: 7/96 (7.3%)<br>Saleh 2014: 4/40 (10%)<br>Veisi 2012: 0/37 (0%)	Bastani 2012: RD 0 (-0.06 to 0.06)<br>Fasubaa 2002: RD 0.46 (0.03 to 0.63)<br>Frass 2011: RD 0.03 (-0.11 to 0.18)<br>Nooh 2017: RD 0.08 (-0.01 to 0.17)<br>Saleh 2014: RD 0.07 (-0.08 to 0.23)	Bastani 2012: 0 fewer per 1000 (from 64 fewer to 64 more)[d]<br>Fasubaa 2002: 140 fewer per 1000 (from 96 fewer to 267 more)<br>Frass 2011: 181 fewer per 1000 (from 153 fewer to 207 fewer)<br>Nooh 2017: 172 fewer per 1000 (from 155 fewer to 188 fewer)<br>Saleh 2014: 173 fewer per 1000 (from 144 fewer to 201 fewer)	VERY LOW

Quality assessment							No of patients		Effect		Certainty
**No of studies**	**Design**	**Risk of bias**	**Inconsistency**	**Indirectness**	**Imprecision[c]**	**Publication bias**	**Vaginal push up**	**Reverse breech extraction**	**Relative risk (95% CI)**	**Absolute**	
									Veisi 2012: RD 0 (-0.05 to 0.05)	Veisi 2012: 0 fewer per 1000 (from 53 fewer to 53 more)[d]	
**Umbilical artery pH - RCTs (Better indicated by lower values)**											
1	Randomised trials (Bastani 2012)	Serious[a]	No serious inconsistency	No serious indirectness	Very serious[c]	None	30	29	Not applicable	MD 0.01 lower (0.05 lower to 0.03 higher)	VERY LOW
**Neonatal death – RCTs**											
3	Randomised trials (Bastani 2012, Fasubaa 2002, Nooh 2017)	Serious[a]	No serious inconsistency[b] (I^2=49%)	No serious indirectness	Very serious[c]	None	16/180 (8.9%)	7/179 (3.9%)	RD 0.03 (-0.03 to 0.09)	38 fewer per 1000 (from 36 fewer to 40 fewer)	VERY LOW

CI: confidence interval; RD = risk difference; MD: mean difference; OR: odds ratio; RCT: randomised controlled trial; RR: risk ratio.

[a] Risk of bias assessed using Cochrane RoB2 for randomised trials and the studies had some concerns because of risk of bias arising from the randomisation process and selection of the reported results (no information provided on pre-specified protocol and statistical analysis plan). For Saleh (2014), there was high risk of bias due to the study being published in a potential predatory journal according to Beall's List of Potential Predatory Journals and Publishers.
[b] Heterogeneity (consistency) was assessed using I^2: The certainty of the evidence was not downgraded if I^2 < 50%, but it was downgraded by one level if I^2 = 50-80%, and by two levels if I^2 >80%.
[c] The imprecision ratings were undertaken by using the optimum information size so that for dichotomous outcomes if the total event rate was ≥300, then the certainty was not downgraded, if the event rate was 150-299, then the certainty was downgraded by one level and if the event rate was <150, then the certainty was downgraded by two levels; and for continuous outcomes if the total n≥400, then the certainty was not downgraded; if the total n=200–399, then the certainty was downgraded by one level; and if the total n<200, then the certainty was downgraded by two levels.
[d] Calculated using risk difference calculator in Review Manager.
*One study was published in a potential predatory journal according to Beall's List of Potential Predatory Journals and Publishers.

2. GRADE summary of findings table for vaginal disimpaction vs Patwardhan method

Quality assessment							No of patients		Effect		Certainty
**No of studies**	**Design**	**Risk of bias**	**Inconsistency**	**Indirectness**	**Imprecision[c]**	**Publication bias**	**Vaginal push up**	**Patwardhan method**	**Relative risk (95% CI)**	**Absolute**	
**Uterine incision extension: Combined - NRS**											
6	Non-randomised studies (Keepanasseril 2019, Lenz 2019, Beeresh 2016, Bhattacharya 2020, Lal 2018, Rakholia 2019)	Very serious[a]	Serious[b] (I^2=79%)	No serious indirectness	Very serious[c]	Yes*	135/523	33/346	3.79 (1.46, 9.86)	218 more per 1000 (from 36 more to 691 more)	VERY LOW
**Uterine incision extension: Incision extension on lower segment - NRS**											
6	Non-randomised studies (Keepanasseril 2019, Lenz 2019, Beeresh 2016, Bhattacharya 2020, Lal 2018, Rakholia 2019)	Very serious[a]	Serious[b] (I^2=63%)	No serious indirectness	Very serious[c]	Yes*	132/523 (25.2%)	26/346 (7.8%)	RR 3.75 (1.76 to 7.96)	215 more per 1000 (from 59 more to 543 more)	VERY LOW
**Uterine incision extension: Angle extension into broad ligaments - NRS**											
1	Non-randomised studies (Keepanasseril 2019)	Very serious[a]	No serious inconsistency	No serious indirectness	Very serious[c]	None	6/221 (2.7%)	6/77 (7.8%)	RR 0.35 (0.12 to 1.05)	51 fewer per 1000 (from 69 fewer to 4 more)	VERY LOW
**Maternal operative blood loss (ml) - NRS (Better indicated by lower values)**											
2	Non-randomised studies (Keepanasseril 2019, Lenz 2019)	Very serious[a]	Very serious[b] (I^2=85%)	No serious indirectness	No serious imprecision	None	Keepanasseril 2019: 221 Lenz 2019: 82	Keepanasseril 2019: 77 Lenz 2019: 55	Not applicable	Keepanasseril 2019: MD 6.10 lower (77.82 lower to 65.62 higher) Lenz 2019: MD 149.50 higher (53.34 to 245.66 higher)	VERY LOW
**Post-partum haemorrhage (operative blood loss >1000 ml) - NRS**											
4	Non-randomised studies (Keepanasseril 2019, Beeresh 2016, Lal 2018, Rakholia 2019,)	Very serious[a]	Serious inconsistency (I^2=70%)	No serious indirectness	Very serious[c]	Yes*	84/391 (21.5%)	18/241 (7.5%)	RR 3.35 (1.29 to 8.66)	139 more per 1000 (from 54 more to 359 more)	VERY LOW
**Operative time (duration of surgery) - minutes - NRS (Better indicated by lower values)**											
2	Non-randomised studies Keepanasseril 2019, Lenz 2019)	Very serious[a]	No serious inconsistency[b] (I^2=17%)	No serious indirectness	No serious imprecision	None	303	132	Not applicable	MD 4.1 higher (0.61 lower to 8.8 higher)	LOW
**Infant birth trauma - NRS**											
5	Non-randomised studies (Keepanasseril 2019, Lenz 2019, Beeresh 2016, Bhattacharya 2020, Lal 2018)	Very serious[a]	No serious inconsistency[b] (I^2=0%)	No serious indirectness	Very serious[c]	Yes*	12/469 (2.6%)	12/284 (4.2%)	RR 0.52 (0.22 to 1.24)	20 fewer per 1000 (from 33 fewer to 10 more)	VERY LOW

Quality assessment							No of patients		Effect		Certainty
No of studies	Design	Risk of bias	Inconsistency	Indirectness	Imprecision[c]	Publication bias	Vaginal push up	Patwardhan method	Relative risk (95% CI)	Absolute	
**Apgar score <7 at five minutes - NRS**											
2	Non-randomised studies (Lenz 2019, Beeresh 2016)	Very serious[a]	No serious inconsistency	No serious indirectness	Very serious[c]	Yes*	10/134 (7.5%)	5/101 (5.0%)	Peto OR 1.73 (0.59 to 5.03)	25 more per 1000 (from 6 fewer to 50 more)[e]	VERY LOW
**Maternal blood transfusion - NRS**											
5	Non-randomised studies (Keepanasseril 2019, Beeresh 2016, Bhattacharya 2020, Lal 2018, Rakholia 2019)	Very serious[a]	Serious inconsistency (I^2=64%)[b]	No serious indirectness	Very serious[c]	Yes*	89/441 (20.2%)	36/281 (12.8%)	RR 1.85 (0.99 to 3.47)	109 more per 1000 (from 1 fewer to 316 more)	VERY LOW
**Inverted T or J incision - NRS**											
1	Non-randomised studies (Lenz 2019)	Very serious[a]	No serious inconsistency	No serious indirectness	Very serious[c]	None	5/82 (6.1%)	2/55 (3.6%)	RR 1.68 (0.34 to 8.34)	25 more per 1000 (from 24 fewer to 267 more)	VERY LOW
**Uterine incision extension into cervix or vagina - NRS**											
1	Non-randomised studies (Keepanasseril 2019)	Very serious[a]	No serious inconsistency	No serious indirectness	Very serious[c]	None	6/221 (2.7%)	3/77 (3.9%)	RR 0.70 (0.18 to 2.72)	12 fewer per 1000 (from 32 fewer to 67 more)	VERY LOW
**Injury to the urinary tract - NRS**											
4	Non-randomised studies (Keepanasseril 2019, Beeresh 2016, Bhattacharya 2020, Lal 2018)	Very serious[a]	No serious inconsistency (I^2=0%)	No serious indirectness	Very serious[c]	Yes*	12/387 (3.1%)	1/229 (0.4%)	Peto OR 4.39 (1.40 to 13.73)	27 more per 1000 (from 13 fewer to 54 more)[e]	VERY LOW
**Wound infection - NRS**											
1	Non-randomised studies (Keepanasseril 2019)	Very serious[a]	No serious inconsistency	No serious indirectness	Very serious[c]	None	14/221 (6.3%)	12/77 (15.6%)	RR 0.41 (0.2 to 0.84)	92 fewer per 1000 (from 25 fewer to 125 fewer)	VERY LOW
**Maternal duration of hospital stay (days) - NRS (Better indicated by lower values)**											
1	Non-randomised studies (Keepanasseril 2019)	Very serious[a]	No serious inconsistency	No serious indirectness	Serious[c]	None	221	77	Median (range): Vaginal push up: = 7 (3 to 45); Patwardhan method: 8 (3 to 7) p=0.233[d]		VERY LOW
**Incision-to-delivery interval (minutes) - NRS (Better indicated by lower values)**											
1	Non-randomised studies (Lenz 2019)	Very serious[a]	No serious inconsistency	No serious indirectness	Very serious[c]	None	82	55	Not applicable	MD 0.3 higher (0.66 lower to 1.26 higher)	VERY LOW
**NICU admission - NRS**											
6	Non-randomised studies (Keepanasseril 2019, Lenz 2019, Beeresh 2016, Bhattacharya 2020, Lal 2018, Rakholia 2019)	Very serious[a]	No serious inconsistency[b] (I^2=49%)	No serious indirectness	Serious[c]	Yes*	132/523 (25.2%)	87/346 (25.1%)	RR 0.96 (0.05 to 1.42)	10 fewer per 1000 (from 239 fewer to 106 more)	VERY LOW

Quality assessment							No of patients		Effect		Certainty
**No of studies**	**Design**	**Risk of bias**	**Inconsistency**	**Indirectness**	**Imprecision**[c]	**Publica-tion bias**	**Vaginal push up**	**Patwardhan method**	**Relative risk (95% CI)**	**Absolute**	
**Umbilical artery pH/cord pH <7.15 - NRS**											
1	Non-randomised studies (Lenz 2019)	Very serious[a]	No serious inconsistency	No serious indirectness	Very serious[c]	None	8/82 (9.8%)	4/55 (7.3%)	RR 1.34 (0.42 to 4.24)	25 more per 1000 (from 42 fewer to 236 more)	VERY LOW
**Neonatal death - NRS**											
4	Non-randomised studies (Keepanasseril 2019, Lenz 2019, Bhattacharya 2020, Rakholia 2019)	Very serious[a]	No serious inconsistency (I^2=23%)	No serious indirectness	Serious[c]	Yes*	14/407 (3.4%)	10/244 (4.1%)	Peto OR 1.12 (0.47 to 2.65)	7 fewer per 1000 (from 3 fewer to 16 more)	VERY LOW

CI: confidence interval; RD = risk difference; MD: mean difference; NRS: non-randomised study; OR: odds ratio; RR: risk ratio.

[a] Risk of bias assessed using ROBINS-I for non-randomised studies and the overall risk of bias of this study was serious because of serious risk of bias due to lack of adjustment for confounding in the analyses and moderate/critical risk of bias because no mention of pre-registered protocol or statistical analysis plan and/or published in a potential predatory journal according to Beall's List of Potential Predatory Journals and Publishers.
[b] Heterogeneity (consistency) was assessed using I^2: The certainty of the evidence was not downgraded if I^2 < 50%, but it was downgraded by one level if I^2 = 50-80%, and by two levels if I^2 >80%.
[c] The imprecision ratings were undertaken by using the optimum information size so that for dichotomous outcomes if the total event rate was ≥300, then the certainty was not downgraded, if the event rate was 150-299, then the certainty was downgraded by one level and if the event rate was <150, then the certainty was downgraded by two levels; and for continuous outcomes if the total n≥400, then the certainty was not downgraded; if the total n=200–399, then the certainty was downgraded by one level; and if the total n<200, then the certainty was downgraded by two levels.
[d] Data are only reported as medians and ranges and the p value was obtained using the Fischer's exact test.
[e] Calculated using risk difference calculator in Review Manager as absolute risk difference could not be calculated.
*One or more studies published in a potential predatory journal according to Beall's List of Potential Predatory Journals and Publishers.

3. GRADE summary of findings table for vaginal disimpaction or reverse breech extraction vs Patwardhan method

Quality assessment							No of patients		Effect		Certainty
**No of studies**	**Design**	**Risk of bias**	**Inconsistency**	**Indirectness**	**Imprecision[c]**	**Publication bias**	**Vaginal push up or reverse breech extraction**	**Patwardhan method**	**Relative risk (95% CI)**	**Absolute**	
**Uterine incision extension: Incision extension on lower segment – RCTs and NRS**											
3	Randomised trials (Bhoi 2019) and non-randomised studies (Bansiwal 2017, Saha 2014)	Very serious[a, b]	No serious inconsistency[d] (I^2=0%)	No serious indirectness	Very serious[c]	Yes*	61/406 (15%)	2/228 (0.9%)	Peto OR 5.42 (3.14 to 9.34)	37 more per 1000 (from 18 more to 68 more)	VERY LOW
**Post-partum haemorrhage - NRS**											
1	Non-randomised studies (Bansiwal 2017)	Very serious[b]	No serious inconsistency	No serious indirectness	Very serious[c]	Yes*	16/71 (22.5%)	1/64 (1.6%)	RR 14.42 (1.97 to 105.7)	210 more per 1000 (from 15 more to 1000 more)	VERY LOW
**Operative time (duration of surgery) - minutes - RCTs (Better indicated by lower values)**											
1	Randomised trials (Bhoi 2019)	Very serious[a]	No serious inconsistency	No serious indirectness	No serious imprecision	None	291	129	Not applicable	MD 6.58 higher (3.27 to 9.89 higher)	LOW
**Apgar score <7 at five minutes - NRS**											
1	Non-randomised studies (Saha 2014)	Very serious[b]	No serious inconsistency	No serious indirectness	Very serious[c]	None	6/44 (13.6%)	4/35 (11.4%)	RR 1.19 (0.36 to 3.90)	22 more per 1000 (from 73 fewer to 331 more)	VERY LOW
**Apgar score <3 at 5 minutes - RCTs**											
1	Randomised trials (Bhoi 2019)	No serious risk of bias	No serious inconsistency	No serious indirectness	Very serious[c]	None	38/291 (13.1%)	10/129 (7.8%)	RR 1.68 (0.87 to 3.28)	53 more per 1000 (from 10 fewer to 177 more)	LOW
**Maternal blood transfusion - RCTs and NRS**											
3	Randomised trials (Bhoi 2019) and non-randomised studies (Bansiwal 2017, Saha 2014)	Very serious[a, b]	No serious inconsistency[d] (I^2=12%)	No serious indirectness	Very serious[c]	Yes*	67/406 (16.5%)	10/228 (4.4%)	RR 3.62 (1.77 to 7.41)	115 more per 1000 (from 34 more to 281 more)	VERY LOW
**Maternal duration of hospital stay (days) - RCTs (Better indicated by lower values)**											
1	Randomised trials (Bhoi 2019)	Very serious[a]	No serious inconsistency	No serious indirectness	No serious imprecision	None	291	129	Not applicable	MD 0.48 higher (0.51 lower to 1.47 higher)	LOW
**NICU admission – RCTs and NRS**											
3	Randomised trials (Bhoi 2019) and non-randomised studies (Bansiwal 2017, Saha 2014)	Very serious[a, b]	No serious inconsistency[d] (I^2=0%)	No serious indirectness	Serious[c]	Yes*	112/406 (27.6%)	56/228 (24.6%)	RR 1.11 (0.84 to 1.48)	27 more per 1000 (from 39 fewer to 118 more)	LOW

CI: confidence interval; RD = risk difference; MD: mean difference; NRS: non-randomised study; OR: odds ratio; RCT: randomised controlled trial; RR: risk ratio.

[a] Risk of bias assessed using the Cochrane RoB2 for randomised trials and overall this study had some concerns due to risk of bias in the randomisation process and selection of the reported result.
[b] Risk of bias assessed using ROBINS-I for non-randomised studies and overall the studies were at serious risk of bias because of confounding, or at critical risk of bias due to being published in a potential predatory journal according to Beall's List of Potential Predatory Journals and Publishers.
[c] The imprecision ratings were undertaken by using the optimum information size so that for dichotomous outcomes if the total event rate was ≥300, then the certainty was not downgraded, if the event rate was 150-299, then the certainty was downgraded by one level and if the event rate was <150, then the certainty was downgraded by two levels; and for continuous outcomes if the total n≥400, then the certainty was not downgraded; if the total n=200–399, then the certainty was downgraded by one level; and if the total n<200, then the certainty was downgraded by two levels.
[d] Heterogeneity (consistency) was assessed using I^2: The certainty of the evidence was not downgraded if $I^2 < 50\%$, but it was downgraded by one level if $I^2 = 50-80\%$, and by two levels if $I^2 > 80\%$.
*One study was published in a potential predatory journal according to Beall's List of Potential Predatory Journals and Publishers.

4. GRADE summary of findings table for Fetal Pillow® vs no pillow

Quality assessment							No of patients		Effect		Certainty
No of studies	Design	Risk of bias	Inconsistency[a]	Indirectness	Imprecision[b]	Publication bias	Fetal pillow	No fetal pillow	Relative risk (95% CI)	Absolute	
**Uterine incision extension: Combined – NRS**											
4	Non-randomised studies (Chooi 2022, Hanley 2020, Sacre 2021, Seal 2014)	Very serious[c,]	No serious inconsistency[a] (I^2=31%)	No serious indirectness	Serious[b]	Yes*	63/387 (16.3%)	89/453 (19.6%)	RR 0.81 (0.60 to 1.10)	37 fewer per 1000 (from 79 fewer to 20 more)	VERY LOW
**Uterine incision extension: Incision extension on lower segment – NRS**											
2	Non-randomised studies (Hanley 2020, Sacre 2021)	Very serious[c]	No serious inconsistency[a] (I^2=0%)	No serious indirectness	Very serious[b]	None	50/284 (17.6%)	58/281 (20.6%)	RR 0.89 (0.57 to 1.38)	23 fewer per 1000 (from 89 fewer to 78 more)	VERY LOW
**Maternal operative blood loss (ml) - NRS (Better indicated by lower values)**											
2	Non-randomised studies (Chooi 2022, Hanley 2020)	Very serious[c]	No serious inconsistency (I^2=0%)	No serious indirectness	Serious[b]	None	166	107	Not applicable	MD 28.67 lower (127.82 lower to 70.48 higher)	VERY LOW
**Maternal operative blood loss >1000 ml - NRS**											
4	Non-randomised studies (Chooi 2022, Hanley 2020, Sacre 2021, Seal 2014)	Very serious[c]	No serious inconsistency[a] (I^2=0%)	No serious indirectness	Very serious[b]	Yes*	69/387 (17.8%)	72/453 (15.9%)	RR 1.10 (0.81 to 1.49)	13 more per 1000 (from 35 fewer to 79 more)	VERY LOW
**Operative time (minutes) – NRS (Better indicated by lower values)**											
1	Non-randomised study (Seal 2014)	Very serious[c, d]	No serious inconsistency[a]	No serious indirectness	Serious[b]	Yes*	50	124	Not applicable	MD 20.30 lower (22.57 to 18.03 lower)	VERY LOW
**Infant birth trauma – NRS**											
2	2 non-randomised studies (Chooi 2022, Seal 2014)	Very serious[c]	Serious[a] (I^2=68%)	No serious indirectness	Very serious[b]	Yes*	16/103 (15.5%)	17/172 (9.9%)	Peto OR 1.02 (0.46 to 2.24)	4 more per 1000 (from 44 fewer to 95 more)	VERY LOW
**Apgar score at five minutes – NRS**											
1	Non-randomised studies (Hanley 2020)	Serious[c]	No serious inconsistency	No serious indirectness	Very serious[b]	None	113	60	Not applicable	MD 0.02 higher (0.31 lower to 0.35 higher)	VERY LOW
**Apgar score <7 at five minutes – NRS**											
3	Non-randomised studies (Chooi 2022, Hanley 2020, Sacre 2021)	Very serious[c]	No serious inconsistency[a] (I^2=0%)	No serious indirectness	Serious[b]	None	119/336 (35.4%)	71/329 (21.6%)	RR 1.00 (0.92 to 1.09)	0 more per 1000 (from 35 fewer to 45 more)	VERY LOW
**Apgar score ≤ 3 at five minutes – NRS**											
1	Non-randomised study (Seal 2014)	Very serious[c,]	No serious inconsistency[a]	No serious indirectness	Very serious[b]	Yes*	2/50 (4.0%)	4/124 (3.2%)	RR 1.24 (0.23 to 6.56)	8 more per 1000 (from 25 fewer to 179 more)	VERY LOW
**Blood transfusion – RCTs and NRS**											
4	Non-randomised studies (Chooi 2022, Hanley 2020, Sacre 2021, Seal 2014)	Serious[c, d]	No serious inconsistency[a] (I^2=9%)	No serious indirectness	Very serious[b]	Yes*	12/387 (3.1%)	19/451 (4.2%)	RR 0.81 (0.35 to 1.87)	8 fewer per 1000 (from 26 fewer to 27 more)	VERY LOW
**Inverted T or J incision – NRS**											
1	Non-randomised studies (Hanley 2020)	Very serious[c]	No serious inconsistency	No serious indirectness	Very serious[b]	None	3/114 (2.6%)	2/60 (3.3%)	RR 0.79 (0.14 to 4.6)	7 fewer per 1000 (from 29 fewer to 120 more)	VERY LOW

Quality assessment							No of patients		Effect		Certainty
No of studies	Design	Risk of bias	Inconsistency[a]	Indirectness	Imprecision[b]	Publication bias	Fetal pillow	No fetal pillow	Relative risk (95% CI)	Absolute	
**Uterine incision extension into cervix / vagina – NRS**											
2	Non-randomised studies (Hanley 2020, Seal 2014)	Very serious[c]	Very serious[a] (I^2=95%)	No serious indirectness	Very serious[b]	Yes*	Hanley 2020: 0/114 (0%) Seal 2014: 2/50 (0.04%)	Hanley 2020: 0/60 (0%) Seal 2014: 19/124 (15.3%)	Hanley 2020: RD 0.00 (-0.03 to 0.03) Seal 2014: RD -0.11 (-0.24 to 0.14)	Hanley 2020: 0 fewer per 1000 (from 26 fewer to 26 more)[g] Seal 2014: 170 fewer per 1000 (from 132 fewer to 190 fewer)	VERY LOW
**Injury to the urinary tract (including ureteric injury and bladder injury) – NRS**											
1	Non-randomised studies (Hanley 2020)	Very serious[c]	No serious inconsistency	No serious indirectness	Very serious[b]	None	2/114 (1.8%)	2/60 (3.3%)	RR 0.53 (0.08 to 3.64)	16 fewer per 1000 (from 31 fewer to 88 more)	VERY LOW
**Maternal duration of hospital stay (days) - NRS (Better indicated by lower values)**											
3	Non-randomised studies (Chooi 2022, Hanley 2020, Seal 2014)	Very serious[c]	No serious inconsistency (for Chooi 2022 and Hanley 2020 pooled) (I^2=0%)[f]<br><br>Very serious inconsistency (with Seal 2014) [f]	No serious indirectness	Serious (for Chooi 2022, Hanley 2020 pooled) [b,] Very serious (with Seal 2014) [b, f]	Yes*	Chooi 2022:53 Hanley 2020: 114 Seal 2014: 50	Chooi 2022:48 Hanley 2020: 59 Seal 2014: 124	Not applicable	Chooi 2022, Hanley 2020 (2 studies) pooled: MD 0.26 lower (0.56 lower to 0.03 higher) Seal 2014: Fetal pillow mean 4.1; no fetal pillow mean 6.4[f]	VERY LOW
**Maternal duration of hospital stay (delivery to discharge) - NRS (Better indicated by lower values)**											
1	Non-randomised studies (Sacre 2021)	Very serious[c]	No serious inconsistency	No serious indirectness	Serious[b]	None	170	221	Median (interquartile range): Fetal pillow: = 2 (2 to 3); No-fetal pillow: 2 (2 to 3) p value not reported[e]		VERY LOW
**Incision-to-delivery interval (seconds) - NRS (Better indicated by lower values)**											
1	Non-randomised studies (Seal 2014)	Very serious[c]	No serious inconsistency	No serious indirectness	Very serious[b]	Yes*	50	124	Not applicable	MD 338.4 lower (357.55 to 319.25 lower)	VERY LOW
**NICU admission - NRS**											
3	Non-randomised studies (Hanley 2020, Sacre 2021, Seal 2014)	Very serious[c, d]	No serious inconsistency[a] (I^2=0%)	No serious indirectness	Very serious[b]	Yes*	55/332 (16.6%)	63/405 (15.6%)	RR 0.78 (0.55 to 1.08)	37 fewer per 1000 (from 70 fewer to 9 higher	VERY LOW
**Umbilical artery pH - NRS (Better indicated by lower values)**											
2	Non-randomised studies (Chooi 2022, Hanley 2020)	Very serious[c]	Very serious inconsistency (I^2=81%)	No serious indirectness	Serious[b]	None	Chooi 2022: 53 Hanley 2020: 98	Chooi 2020: 48 Hanley 2020:49	Not applicable	Chooi 2022: MD 0.01 higher (0.02 lower to 0.04 higher)	VERY LOW

Quality assessment							No of patients		Effect		Certainty
No of studies	Design	Risk of bias	Inconsistency[a]	Indirectness	Imprecision[b]	Publication bias	Fetal pillow	No fetal pillow	Relative risk (95% CI)	Absolute	
										Hanley 2020: MD 0.06 higher (0.03 to 0.09 higher)	
**Umbilical artery pH <7.10 – NRS**											
1	Non-randomised studies (Sacre 2021)	Very serious[c]	No serious inconsistency	No serious indirectness	Very serious[b]	None	12/170 (7.1%)	29/221 (13.1%)	RR 0.54 (0.28 to 1.02)	60 fewer per 1000 (from 94 fewer to 3 more)	VERY LOW
**Neonatal death – NRS**											
1	Non-randomised study (Seal 2014)	Very serious[c, d]	No serious inconsistency[a]	No serious indirectness	Very serious[b]	Yes*	0/50 (0%)	2/124 (0.6%)	Peto OR 0.24 (0.01 to 5.26)	8 fewer per 1000 (from 16 fewer to 146 more)	VERY LOW

CI: confidence interval; RD = risk difference; RD = risk difference; MD: mean difference; NRS: non-randomised study; OR: odds ratio; RCT: randomised controlled trial; RR: risk ratio.

[a] Heterogeneity (consistency) was assessed using I^2: The certainty of the evidence was not downgraded if I^2 < 50%, but it was downgraded by one level if I^2 = 50-80%, and by two levels if I^2 >80%.
[b] The imprecision ratings were undertaken by using the optimum information size so that for dichotomous outcomes if the total event rate was ≥300, then the certainty was not downgraded, if the event rate was 150-299, then the certainty was downgraded by one level and if the event rate was <150, then the certainty was downgraded by two levels; and for continuous outcomes if the total n≥400, then the certainty was not downgraded; if the total n=200–399, then the certainty was downgraded by one level; and if the total n<200, then the certainty was downgraded by two levels.
[c] Risk of bias assessed using ROBINS-I for non-randomised studies and overall the studies were at serious risk of bias because of confounding or missing data, or at critical risk of bias due to a retraction of an article with the same authors and similar data.
[d] Risk of bias assessed using the Cochrane RoB2 for randomised trials and overall this study had some concerns due to risk of bias in selection of the reported result.
[e] Data are only reported as medians and interquartile ranges.
[f] Seal 2014 did not report standard deviations so I^2 and MD are not estimable
[g] Calculated using risk difference calculator in Review Manager.
* Due to retraction of an article with the same authors and similar data.

5. GRADE summary of findings table for inflated Fetal Pillow® vs non-inflated pillow

Quality assessment							No of patients		Effect		Certainty
No of studies	Design	Risk of bias	Inconsistency	Indirectness	Imprecision[a]	Publication bias	Fetal pillow (inflated)	Non-inflated fetal pillow	Relative risk (95% CI)	Absolute	
**Uterine incision extension: Combined – RCTs**											
1	Randomised trials (Lassey 2020)	No serious risk of bias	No serious inconsistency	No serious indirectness	Very serious[a]	None	6/30 (20%)	13/30 (43.3%)	RR 0.46 (0.2 to 1.05)	234 fewer per 1000 (from 347 fewer to 22 more)	LOW
**Uterine incision extension: Incision extension on lower segment – RCTs**											
1	Randomised trials (Lassey 2020)	No serious risk of bias	No serious inconsistency	No serious indirectness	Very serious[a]	None	6/30 (20%)	9/30 (30%)	RR 0.67 (0.27 to 1.64)	99 fewer per 1000 (from 219 fewer to 192 more)	LOW
**Maternal operative blood loss (ml) - RCTs (Better indicated by lower values)**											
1	Randomised trials (Lassey 2020)	No serious risk of bias	No serious inconsistency	No serious indirectness	Very serious[a]	None	30	30	Median (interquartile range):<br>Fetal pillow (inflated): 800 (700 to 900)<br>Non-inflated fetal pillow: 900 (750 to 1050)<br>p=.09[b]		LOW
**Operative time (minutes) - RCTs (Better indicated by lower values)**											
1	Randomised trials (Lassey 2020)	No serious risk of bias	No serious inconsistency	No serious indirectness	Very serious[a]	None	30	30	Median (interquartile range):<br>Fetal pillow (inflated): 56 (50 to 62)<br>Non-inflated fetal pillow: 59 (52 to 70)<br>p=.14[b]		LOW
**Apgar score at 5 minutes - RCTs (Better indicated by higher values)**											
1	Randomised trials (Lassey 2020)	No serious risk of bias	No serious inconsistency	No serious indirectness	Very serious[a]	None	30	30	Median (interquartile range):<br>Fetal pillow (inflated): 9 (9 to 9)<br>Non-inflated fetal pillow: 9 (9 to 9)<br>p=.84[b]		LOW
**Blood transfusion – RCTs**											
1	Randomised trials (Lassey 2020)	No serious risk of bias	No serious inconsistency	No serious indirectness	Very serious[a]	None	0/30 (0%)	3/30 (10%)	Peto OR 0.13 (0.01 to 1.26)	87 fewer per 1000 (from 99 fewer to 26 more)	LOW
**Uterine incision extension into cervix or vagina – RCTs**											
1	Randomised trials (Lassey 2020)	No serious risk of bias	No serious inconsistency	No serious indirectness	Very serious[a]	None	0/30 (0%)	4/30 (13.3%)	Peto OR 0.12 (0.02 to 0.91)	117 fewer per 1000 (from 12 fewer to 131 fewer)	LOW
**Postpartum pyrexia or maternal sepsis – RCTs**											
1	Randomised trials (Lassey 2020)	No serious risk of bias	No serious inconsistency	No serious indirectness	Very serious[a]	None	6/30 (20%)	5/30 (16.7%)	RR 1.2 (0.41 to 3.51)	33 more per 1000 (from 98 fewer to 418 more)	LOW

Quality assessment							No of patients		Effect		Certainty
No of studies	Design	Risk of bias	Inconsistency	Indirectness	Imprecision[a]	Publication bias	Fetal pillow (inflated)	Non-inflated fetal pillow	Relative risk (95% CI)	Absolute	
**Maternal duration of hospital stay (days) - RCTs (Better indicated by lower values)**											
1	Randomised trials (Lassey 2020)	No serious risk of bias	No serious inconsistency	No serious indirectness	Very serious[a]	None	30	30	Median (interquartile range):<br>Fetal pillow (inflated): 4 (4 to 4)<br>Non-inflated fetal pillow: 4 (4 to 4)<br>p=.43[b]		LOW
**Incision to delivery time (seconds) - RCTs (Better indicated by lower values)**											
1	Randomised trials (Lassey 2020)	No serious risk of bias	No serious inconsistency	No serious indirectness	Very serious[a]	None	30	30	Median (interquartile range):<br>Fetal pillow (inflated): 31 (24 to 37)<br>Non-inflated fetal pillow: 54 (41 to 72),<br>p<.01[b]		LOW

CI: confidence interval; RD = risk difference; MD: mean difference; OR: odds ratio; RCT: randomised controlled trial; RR: risk ratio.

[a] The imprecision ratings were undertaken by using the optimum information size so that for dichotomous outcomes if the total event rate was ≥300, then the certainty was not downgraded, if the event rate was 150-299, then the certainty was downgraded by one level and if the event rate was <150, then the certainty was downgraded by two levels; and for continuous outcomes if the total n≥400, then the certainty was not downgraded; if the total n=200–399, then the certainty was downgraded by one level; and if the total n<200, then the certainty was downgraded by two levels.
[b] Data are only reported as medians and interquartile ranges and the p value appears to be obtained using the Wilcoxon rank sum test.

6. GRADE summary of findings table for Fetal Pillow® vs vaginal disimpaction

Quality assessment							No of patients		Effect		Certainty
**No of studies**	**Design**	**Risk of bias**	**Inconsistency**	**Indirectness**	**Imprecision[b]**	**Publication bias**	**Fetal pillow**	**Vaginal push up**	**Relative risk (95% CI)**	**Absolute**	
**Uterine incision extension: Incision extension on lower segment - NRS**											
1	Non-randomised studies (Safa 2016)	Very serious[a]	No serious inconsistency	No serious indirectness	Very serious[b]	None	18/91 (19.8%)	24/69 (34.8%)	RR 0.57 (0.34 to 0.96)	150 fewer per 1000 (from 14 fewer to 230 fewer)	VERY LOW
**Maternal operative blood loss (ml) - NRS (Better indicated by lower values)**											
1	Non-randomised studies (Safa 2016)	Very serious[a]	No serious inconsistency	No serious indirectness	Very serious[b]	None	91	69	Not applicable	MD 130 lower (185.61 to 74.39 lower)	VERY LOW
**Apgar score <7 at 5 minutes - NRS**											
1	Non-randomised studies (Safa 2016)	Very serious[a]	No serious inconsistency	No serious indirectness	Very serious[b]	None	3/91 (3.3%)	4/69 (5.8%)	RR 0.57 (0.13 to 2.46)	25 fewer per 1000 (from 50 fewer to 85 more)	VERY LOW
**Maternal blood transfusion - NRS**											
1	Non-randomised studies (Safa 2016)	Very serious[a]	No serious inconsistency	No serious indirectness	Very serious[b]	None	3/91 (3.3%)	2/69 (2.9%)	RR 1.14 (0.2 to 6.62)	4 more per 1000 (from 23 fewer to 163 more)	VERY LOW
**Maternal hospital stay (hours) - NRS (Better indicated by lower values)**											
1	Non-randomised studies (Safa 2016)	Very serious[a]	No serious inconsistency	No serious indirectness	Very serious[b]	None	91	69	Not applicable	MD 19.9 lower (27.56 to 12.24 lower)	VERY LOW
**NICU admission - NRS**											
1	Non-randomised studies (Safa 2016)	Very serious[a]	No serious inconsistency	No serious indirectness	Very serious[b]	None	14/91 (15.4%)	17/69 (24.6%)	RR 0.62 (0.33 to 1.18)	94 fewer per 1000 (from 165 fewer to 44 more)	VERY LOW
**Cord arterial pH - NRS (Better indicated by higher values)**											
1	Non-randomised studies (Safa 2016)	Very serious[a]	No serious inconsistency	No serious indirectness	Very serious[b]	None	91	69	Not applicable	MD 0.05 higher (0.03 to 0.07 higher)	VERY LOW

CI: confidence interval; RD = risk difference; MD: mean difference; NRS: non-randomised study; RR: risk ratio.

[a] Risk of bias assessed using ROBINS-I for non-randomised studies and the overall risk of bias of this study was serious because of serious risk of bias due to lack of adjustment for confounding in the analyses and moderate risk of bias because of limited details on vaginal push up methods and no mention of pre-registered protocol or statistical analysis plan.
[b] The imprecision ratings were undertaken by using the optimum information size so that for dichotomous outcomes if the total event rate was ≥300, then the certainty was not downgraded, if the event rate was 150-299, then the certainty was downgraded by one level and if the event rate was <150, then the certainty was downgraded by two levels; and for continuous outcomes if the total n≥400, then the certainty was not downgraded; if the total n=200–399, then the certainty was downgraded by one level; and if the total n<200, then the certainty was downgraded by two levels.

7. GRADE summary of findings table for Fetal Pillow® vs Patwardhan method

Quality assessment							No of patients		Effect		Certainty
**No of studies**	**Design**	**Risk of bias**	**Inconsistency**	**Indirectness**	**Imprecision**[b]	**Publication bias**	**Fetal pillow**	**Modified Patwardhan method**	**Relative risk (95% CI)**	**Absolute**	
**Uterine incision extension: Incision extension on lower segment - RCTs**											
1	Randomised trials (Dutta 2019)	Very serious[a]	No serious inconsistency	No serious indirectness	Very serious[b]	Yes*	2/25 (8%)	6/25 (24%)	RR 0.33 (0.07 to 1.5)	161 fewer per 1000 (from 223 fewer to 120 more)	VERY LOW
**Operative time (minutes) - RCTs - 30 to 40 minutes**											
1	Randomised trials (Dutta 2019)	Very serious[a]	No serious inconsistency	No serious indirectness	Very serious[b]	Yes*	20/25 (80%)	16/25 (64%)	RR 1.25 (0.88 to 1.78)	160 more per 1000 (from 77 fewer to 499 more)	VERY LOW
**Operative time (minutes) - RCTs - 40 to 50 minutes**											
1	Randomised trials (Dutta 2019)	Very serious[a]	No serious inconsistency	No serious indirectness	Very serious[b]	Yes*	3/25 (12%)	7/25 (28%)	RR 0.43 (0.12 to 1.47)	160 fewer per 1000 (from 246 fewer to 132 more)	VERY LOW
**Operative time (minutes) - RCTs - 50 to 60 minutes**											
1	Randomised trials (Dutta 2019)	Very serious[a]	No serious inconsistency	No serious indirectness	Very serious[b]	Yes*	2/25 (8%)	1/25 (4%)	RR 2.00 (0.19 to 20.67)	40 more per 1000 (from 32 fewer to 787 more)	VERY LOW
**Operative time (minutes) - RCTs - >1 hour**											
1	Randomised trials (Dutta 2019)	Very serious[a]	No serious inconsistency	No serious indirectness	Very serious[b]	Yes*	0/25 (0%)	1/25 (4%)	Peto OR 0.14 (0 to 6.82)	34 fewer per 1000 (from 40 fewer to 233 more)	VERY LOW
**Blood transfusion - RCTs**											
1	Randomised trials (Dutta 2019)	Very serious[a]	No serious inconsistency	No serious indirectness	Very serious[b]	Yes*	0/25 (0%)	4/25 (16%)	Peto OR 0.12 (0.02 to 0.9)	141 fewer per 1000 (from 16 fewer to 157 fewer)	VERY LOW
**Incision-to-delivery interval (minutes) - RCTs - 0 to 2 minutes**											
1	Randomised trials (Dutta 2019)	Very serious[a]	No serious inconsistency	No serious indirectness	Very serious[b]	Yes*	13/25 (52%)	2/25 (8%)	RR 6.50 (1.63 to 25.88)	440 more per 1000 (from 50 more to 1000 more)	VERY LOW
**Incision-to-delivery interval (minutes) - RCTs - 2 to 4 minutes**											
1	Randomised trials (Dutta 2019)	Very serious[a]	No serious inconsistency	No serious indirectness	Very serious[b]	Yes*	12/25 (48%)	11/25 (44%)	RR 1.09 (0.60 to 1.99)	40 more per 1000 (from 176 fewer to 436 more)	VERY LOW
**Incision-to-delivery interval (minutes) - RCTs - 4 to 6 minutes**											
1	Randomised trials (Dutta 2019)	Very serious[a]	No serious inconsistency	No serious indirectness	Very serious[b]	Yes*	0/25 (0%)	12/25 (48%)	Peto OR 0.08 (0.02 to 0.27)	442 fewer per 1000 (from 350 fewer to 470 fewer)	VERY LOW
**NICU admission - RCTs**											

Quality assessment							No of patients		Effect		Certainty
**No of studies**	**Design**	**Risk of bias**	**Inconsistency**	**Indirectness**	**Imprecision**[b]	**Publication bias**	**Fetal pillow**	**Modified Patwardhan method**	**Relative risk (95% CI)**	**Absolute**	
1	Randomised trials (Dutta 2019)	Very serious[a]	No serious inconsistency	No serious indirectness	Very serious[b]	Yes*	3/25 (12%)	15/25 (60%)	RR 0.2 (0.07 to 0.61)	480 fewer per 1000 (from 234 fewer to 558 fewer)	VERY LOW
**Neonatal death (defined as death within the first 28 days of life) – RCTs**											
1	Randomised trials (Dutta 2019)	Very serious[a]	No serious inconsistency	No serious indirectness	Very serious[b]	Yes*	0/25 (0%)	1/25 (4%)	Peto OR 0.14 (0.00 to 6.82)	34 fewer per 1000 (from 40 fewer to 233 more)	VERY LOW

CI: confidence interval; RD = risk difference; OR: odds ratio; RCT: randomised controlled trial; RR: risk ratio.

[a] Risk of bias assessed using Cochrane RoB2 for randomised trials and the overall risk of bias of this study had some concerns because of risk of bias in selection of the reported result (little detail regarding the outcomes reported in the methods section), and high risk of bias due to being published in a potential predatory journal according to Beall’s List of Potential Predatory Journals and Publishers.
[b] The imprecision ratings were undertaken by using the optimum information size so that for dichotomous outcomes if the total event rate was ≥300, then the certainty was not downgraded, if the event rate was 150-299, then the certainty was downgraded by one level and if the event rate was <150, then the certainty was downgraded by two levels; and for continuous outcomes if the total n≥400, then the certainty was not downgraded; if the total n=200–399, then the certainty was downgraded by one level; and if the total n<200, then the certainty was downgraded by two level.
* One study was published in a potential predatory journal according to Beall’s List of Potential Predatory Journals and Publishers.
