# Supplementary material for: Management of impacted fetal head at cesarean birth: A systematic review and meta‐analysis
Source: Acta Obstet Gynecol Scand. 2024 May 24;103(9):1702–13. doi: 10.1111/aogs.14873 (PMC11324922; doi:10.1111/aogs.14873)
Supplement: Supplementary file 8 — Table S5. [file AOGS-103-1702-s002.pdf]

Table S5: Assessment of data published in potentially predatory journals or at risk of retraction

First author, year	Medline	Pubmed	Embase	DOAJ	Beall's List of Predatory Journals and Publishers	First or last author of the article with another article that has been retracted
Bansiwal 2017	No	No	No	No	Yes – see 1 below	No
Bastani 2012	Yes	Yes	NA	No	No	No
Beeresh 2016	No	No	No	No	Yes – see 2 below	No
Bhattacharya 2020	No	No	No	No	Most likely – see 3 below	No
Bhoi 2019	No	No	Yes	No	No	
Chooi 2022	Yes	Yes	NA	No	No	No
Dutta 2019	No	No	No	No	Yes– see 4 below	No
Fasubaa 2002	Yes	Yes	NA	Yes (journal)	No	No
Frass 2011	Yes	Yes	NA	No	No	No
Hanley 2020	Yes	Yes	NA	No	No	No
Javed 2022	No	No	Yes	No	No	No
Keepanasseril 2019	Yes	Yes	NA	Yes (journal)		No
Lal 2018	No	No	No	No	Yes – see 5 below	No
Lassey 2020	Yes	Yes	NA	No	No	No
Lenz 2019	Yes	Yes	NA	Yes		No
Nooh 2017	Yes	Yes	NA	Yes	No	No
Rakholia 2019	No	No	No	No	Most likely – see 6 below	No
Sacre 2021	Yes	Yes	NA	No	No	No
Safa 2016	Yes	Yes	NA	No	No	No
Saha 2014	Yes	Yes	NA	Yes	No	
Saleh 2014	No	No	No	No	Yes – see 7 below	No
Seal 2014	Yes	Yes	NA	Yes	No	Yes – see 8 below
Tahir 2020	Yes	Yes	NA	No	No	No
Veisi 2012	Yes	Yes	NA	No	No	No

1. Bansiwal 2017: Yes, searching as a standalone journal comes up in recent update to Beall's as potentially predatory
2. Beeresh 2016: Yes, "Media Academy"
3. Bhattacharya 2020: Most likely, as publisher is "iMedPub Ltd". Listed on Beall's list is "IMED Research Publications".
4. Dutta 2019: Yes, "IOSR journals"
5. Lal 2018: Yes, listed on Beall's list under journal name
6. Rakholia 2019: Most likely – publisher name not identified but searching as a stand-alone journal finds several variants of "International Journal of Advanced Research"; the journal claims to be indexed on PubMed but it cannot be found there
7. Saleh 2014: Yes, "Scientific Research Publishing (SCIRP)"
8. Seal 2014 – Yes, same authors with similar data to Seal et al 2016 (https://doi.org/10.1002/ijgo.14924)
